# Rising rural body-mass index is the main driver of the global obesity epidemic in adults

**DOI:** 10.1038/s41586-019-1171-x

**Published:** 2019-05-08

**Authors:** NCD Risk Factor Collaboration (NCD-RisC), Honor Bixby, James Bentham, Bin Zhou, Mariachiara Di Cesare, Christopher J. Paciorek, James E. Bennett, Cristina Taddei, Gretchen A. Stevens, Andrea Rodriguez-Martinez, Rodrigo M. Carrillo-Larco, Young-Ho Khang, Maroje Sorić, Edward W. Gregg, J. Jaime Miranda, Zulfiqar A. Bhutta, Stefan Savin, Marisa K. Sophiea, Maria L. C. Iurilli, Bethlehem D. Solomon, Melanie J. Cowan, Leanne M. Riley, Goodarz Danaei, Pascal Bovet, Adela Chirita-Emandi, Ian R. Hambleton, Alison J. Hayes, Nayu Ikeda, Andre P. Kengne, Avula Laxmaiah, Yanping Li, Stephen T. McGarvey, Aya Mostafa, Martin Neovius, Gregor Starc, Ahmad A. Zainuddin, Leandra Abarca-Gómez, Ziad A. Abdeen, Shynar Abdrakhmanova, Suhaila Abdul Ghaffar, Zargar Abdul Hamid, Jamila Abubakar Garba, Niveen M. Abu-Rmeileh, Benjamin Acosta-Cazares, Robert J. Adams, Wichai Aekplakorn, Kaosar Afsana, Imelda A. Agdeppa, Carlos A. Aguilar-Salinas, Charles Agyemang, Mohamad Hasnan Ahmad, Noor Ani Ahmad, Naser Ahmadi, Alireza Ahmadvand, Wolfgang Ahrens, Kamel Ajlouni, Fadia AlBuhairan, Shahla AlDhukair, Hazzaa M. Al-Hazzaa, Mohamed M. Ali, Osman Ali, Ala’a Alkerwi, Amani Rashed Al-Othman, Rajaa Al-Raddadi, Mar Alvarez-Pedrerol, Eman Aly, Deepak N. Amarapurkar, Philippe Amouyel, Antoinette Amuzu, Lars Bo Andersen, Sigmund A. Anderssen, Lars H. Ängquist, Ranjit Mohan Anjana, Alireza Ansari-Moghaddam, Hajer Aounallah-Skhiri, Joana Araújo, Inger Ariansen, Tahir Aris, Raphael E. Arku, Nimmathota Arlappa, Krishna K. Aryal, Thor Aspelund, Felix K. Assah, Maria Cecília F. Assunção, May Soe Aung, Juha Auvinen, Mária Avdicová, Ana Azevedo, Fereidoun Azizi, Mehrdad Azmin, Bontha V. Babu, Azli Baharudin, Suhad Bahijri, Jennifer L. Baker, Nagalla Balakrishna, Mohamed Bamoshmoosh, Maciej Banach, Piotr Bandosz, José R. Banegas, Carlo M. Barbagallo, Alberto Barceló, Amina Barkat, Aluisio J. D. Barros, Mauro V. G. Barros, Iqbal Bata, Anwar M. Batieha, Rosangela L. Batista, Zhamilya Battakova, Assembekov Batyrbek, Louise A. Baur, Robert Beaglehole, Silvia Bel-Serrat, Habiba Ben Romdhane, Judith Benedics, Mikhail Benet, Salim Berkinbayev, Antonio Bernabe-Ortiz, Gailute Bernotiene, Heloisa Bettiol, Aroor Bhagyalaxmi, Sumit Bharadwaj, Santosh K. Bhargava, Hongsheng Bi, Yufang Bi, Anna Biehl, Elysée Claude Bika Lele, Mukharram Bikbov, Bihungum Bista, Dusko J. Bjelica, Peter Bjerregaard, Espen Bjertness, Marius B. Bjertness, Cecilia Björkelund, Anneke Blokstra, Simona Bo, Martin Bobak, Lynne M. Boddy, Bernhard O. Boehm, Heiner Boeing, Jose G. Boggia, Carlos P. Boissonnet, Marialaura Bonaccio, Vanina Bongard, Matthias Bopp, Rossana Borchini, Herman Borghs, Lien Braeckevelt, Lutgart Braeckman, Marjolijn C. E. Bragt, Imperia Brajkovich, Francesco Branca, Juergen Breckenkamp, João Breda, Hermann Brenner, Lizzy M. Brewster, Garry R. Brian, Lacramioara Brinduse, Graziella Bruno, H. Bas Bueno-de-Mesquita, Anna Bugge, Marta Buoncristiano, Genc Burazeri, Con Burns, Antonio Cabrera de León, Joseph Cacciottolo, Hui Cai, Tilema Cama, Christine Cameron, José Camolas, Gamze Can, Günay Can, Ana Paula C. Cândido, Felicia Cañete, Mario V. Capanzana, Eduardo Capuano, Vincenzo Capuano, Viviane C. Cardoso, Axel C. Carlsson, Esteban Carmuega, Maria J. Carvalho, Felipe F. Casanueva, Juan-Pablo Casas, Carmelo A. Caserta, Ertugrul Celikcan, Laura Censi, Juraci A. Cesar, Snehalatha Chamukuttan, Angelique W. Chan, Queenie Chan, Himanshu K. Chaturvedi, Nishi Chaturvedi, Norsyamlina Che Abdul Rahim, Chien-Jen Chen, Fangfang Chen, Huashuai Chen, Shuohua Chen, Zhengming Chen, Ching-Yu Cheng, Yiling J. Cheng, Angela Chetrit, Ekaterina Chikova-Iscener, Arnaud Chiolero, Shu-Ti Chiou, María-Dolores Chirlaque, Belong Cho, Yumi Cho, Kaare Christensen, Diego G. Christofaro, Jerzy Chudek, Renata Cifkova, Michelle Cilia, Eliza Cinteza, Frank Claessens, Janine Clarke, Els Clays, Hans Concin, Susana C. Confortin, Cyrus Cooper, Tara C. Coppinger, Simona Costanzo, Dominique Cottel, Chris Cowell, Cora L. Craig, Amelia C. Crampin, Ana B. Crujeiras, Juan J. Cruz, Alexandra Cucu, Liufu Cui, Jean Dallongeville, Albertino Damasceno, Camilla T. Damsgaard, Rachel Dankner, Thomas M. Dantoft, Graziella D’Arrigo, Parasmani Dasgupta, Saeed Dastgiri, Luc Dauchet, Kairat Davletov, Guy De Backer, Dirk De Bacquer, Amalia De Curtis, Giovanni de Gaetano, Stefaan De Henauw, Paula Duarte de Oliveira, Karin De Ridder, Susanne R. de Rooij, Delphine De Smedt, Mohan Deepa, Alexander D. Deev, Abbas Dehghan, Hélène Delisle, Francis Delpeuch, Elaine Dennison, Valérie Deschamps, Klodian Dhana, Meghnath Dhimal, Augusto F. Di Castelnuovo, Juvenal Soares Dias-da-Costa, Alejandro Diaz, Zivka Dika, Shirin Djalalinia, Ha T. P. Do, Annette J. Dobson, Maria Benedetta Donati, Chiara Donfrancesco, Silvana P. Donoso, Angela Döring, Maria Dorobantu, Ahmad Reza Dorosty, Eleonora d’Orsi, Kouamelan Doua, Wojciech Drygas, Jia Li Duan, Charmaine A. Duante, Rosemary B. Duda, Vesselka Duleva, Virginija Dulskiene, Samuel C. Dumith, Vilnis Dzerve, Elzbieta Dziankowska-Zaborszczyk, Ricky Eddie, Eruke E. Egbagbe, Robert Eggertsen, Gabriele Eiben, Ulf Ekelund, Jalila El Ati, Denise Eldemire-Shearer, Marie Eliasen, Paul Elliott, Reina Engle-Stone, Rajiv T. Erasmus, Cihangir Erem, Louise Eriksen, Johan G. Eriksson, Jorge Escobedo-de la Peña, Alun Evans, David Faeh, Caroline H. Fall, Victoria Farrugia Sant’Angelo, Farshad Farzadfar, Mohammad R. Fattahi, Francisco J. Felix-Redondo, Trevor S. Ferguson, Romulo A. Fernandes, Daniel Fernández-Bergés, Daniel Ferrante, Marika Ferrari, Catterina Ferreccio, Eldridge Ferrer, Jean Ferrieres, Anna Fijalkowska, Günther Fink, Krista Fischer, Eric Monterubio Flores, Bernhard Föger, Leng Huat Foo, Ann-Sofie Forslund, Maria Forsner, Heba M. Fouad, Damian K. Francis, Maria do Carmo Franco, Oscar H. Franco, Guillermo Frontera, Flavio D. Fuchs, Sandra C. Fuchs, Yuki Fujita, Takuro Furusawa, Zbigniew Gaciong, Mihai Gafencu, Daniela Galeone, Fabio Galvano, Jingli Gao, Manoli Garcia-de-la-Hera, Dickman Gareta, Sarah P. Garnett, Jean-Michel Gaspoz, Magda Gasull, Louise Gates, Andrea Gazzinelli, Harald Geiger, Johanna M. Geleijnse, Ali Ghanbari, Erfan Ghasemi, Anoosheh Ghasemian, Oana-Florentina Gheorghe-Fronea, Simona Giampaoli, Francesco Gianfagna, Tiffany K. Gill, Jonathan Giovannelli, Glen Gironella, Aleksander Giwercman, Justyna Godos, Sibel Gogen, Rebecca A. Goldsmith, David Goltzman, Helen Gonçalves, Angel R. Gonzalez, David A. Gonzalez-Chica, Marcela Gonzalez-Gross, Margot González-Leon, Juan P. González-Rivas, María-Elena González-Villalpando, Frederic Gottrand, Antonio Pedro Graça, Sidsel Graff-Iversen, Dušan Grafnetter, Aneta Grajda, Maria G. Grammatikopoulou, Ronald D. Gregor, Tomasz Grodzicki, Anders Grøntved, Giuseppe Grosso, Gabriella Gruden, Dongfeng Gu, Emanuela Gualdi-Russo, Elias F. Gudmundsson, Vilmundur Gudnason, Ramiro Guerrero, Idris Guessous, Andre L. Guimaraes, Martin C. Gulliford, Johanna Gunnlaugsdottir, Marc Gunter, Xiuhua Guo, Yin Guo, Prakash C. Gupta, Rajeev Gupta, Oye Gureje, Beata Gurzkowska, Laura Gutierrez, Felix Gutzwiller, Farzad Hadaegh, Charalambos A. Hadjigeorgiou, Rosa Haghshenas, Jytte Halkjær, Rebecca Hardy, Rachakulla Hari Kumar, Maria Hassapidou, Jun Hata, Teresa Haugsgjerd, Jiang He, Yuna He, Regina Heidinger-Felso, Mirjam Heinen, Tatjana Hejgaard, Marleen Elisabeth Hendriks, Ana Henriques, Leticia Hernandez Cadena, Sauli Herrala, Victor M. Herrera, Isabelle Herter-Aeberli, Ramin Heshmat, Allan G. Hill, Sai Yin Ho, Suzanne C. Ho, Michael Hobbs, Albert Hofman, Wilma M. Hopman, Andrea R. V. R. Horimoto, Claudia M. Hormiga, Bernardo L. Horta, Leila Houti, Christina Howitt, Thein Thein Htay, Aung Soe Htet, Maung Maung Than Htike, Yonghua Hu, José María Huerta, Ilpo Tapani Huhtaniemi, Constanta Huidumac Petrescu, Martijn Huisman, Abdullatif Husseini, Chinh Nguyen Huu, Inge Huybrechts, Nahla Hwalla, Jolanda Hyska, Licia Iacoviello, Jesús M. Ibarluzea, Mohsen M. Ibrahim, Norazizah Ibrahim Wong, M. Arfan Ikram, Vilma E. Irazola, Takafumi Ishida, Muhammad Islam, Aziz al-Safi Ismail, Vanja Ivkovic, Masanori Iwasaki, Tuija Jääskeläinen, Rod T. Jackson, Jeremy M. Jacobs, Hashem Jaddou, Tazeen Jafar, Kenneth James, Kazi M. Jamil, Konrad Jamrozik, Imre Janszky, Edward Janus, Juel Jarani, Marjo-Riitta Jarvelin, Grazyna Jasienska, Ana Jelakovic, Bojan Jelakovic, Garry Jennings, Seung-lyeal Jeong, Chao Qiang Jiang, Ramon O. Jimenez, Michel Joffres, Mattias Johansson, Jari J. Jokelainen, Jost B. Jonas, Torben Jørgensen, Pradeep Joshi, Dragana P. Jovic, Jacek Józwiak, Anne Juolevi, Gregor Jurak, Vesna Juresa, Rudolf Kaaks, Anthony Kafatos, Eero O. Kajantie, Ofra Kalter-Leibovici, Nor Azmi Kamaruddin, Yves Kameli, Efthymios Kapantais, Khem B. Karki, Amir Kasaeian, Marzieh Katibeh, Joanne Katz, Peter T. Katzmarzyk, Jussi Kauhanen, Prabhdeep Kaur, Maryam Kavousi, Gyulli Kazakbaeva, Ulrich Keil, Lital Keinan-Boker, Sirkka Keinänen-Kiukaanniemi, Roya Kelishadi, Cecily Kelleher, Han C. G. Kemper, Alina Kerimkulova, Mathilde Kersting, Timothy Key, Yousef Saleh Khader, Davood Khalili, Mohammad Khateeb, Kay-Tee Khaw, Bahareh Kheiri, Alireza Khosravi, Ilse M. S. L. Khouw, Stefan Kiechl, Ursula Kiechl-Kohlendorfer, Japhet Killewo, Jeongseon Kim, Yeon-Yong Kim, Jeannette Klimont, Jurate Klumbiene, Michael Knoflach, Bhawesh Koirala, Elin Kolle, Patrick Kolsteren, Jürgen König, Raija Korpelainen, Paul Korrovits, Magdalena Korzycka, Seppo Koskinen, Katsuyasu Kouda, Viktoria A. Kovacs, Sudhir Kowlessur, Slawomir Koziel, Wolfgang Kratzer, Susi Kriemler, Peter Lund Kristensen, Steinar Krokstad, Daan Kromhout, Herculina S. Kruger, Ruzena Kubinova, Renata Kuciene, Diana Kuh, Urho M. Kujala, Enisa Kujundzic, Zbigniew Kulaga, R. Krishna Kumar, Marie Kunešová, Pawel Kurjata, Yadlapalli S. Kusuma, Kari Kuulasmaa, Catherine Kyobutungi, Quang Ngoc La, Fatima Zahra Laamiri, Tiina Laatikainen, Carl Lachat, Youcef Laid, Tai Hing Lam, Maja Lang Morovic, Vera Lanska, Georg Lappas, Bagher Larijani, Tint Swe Latt, Lars E. Laugsand, Laura Lauria, Maria Lazo-Porras, Khanh Le Nguyen Bao, Agnès Le Port, Tuyen D. Le, Jeannette Lee, Jeonghee Lee, Paul H. Lee, Terho Lehtimäki, Daniel Lemogoum, Naomi S. Levitt, Christa L. Lilly, Wei-Yen Lim, M. Fernanda Lima-Costa, Hsien-Ho Lin, Xu Lin, Lars Lind, Allan Linneberg, Lauren Lissner, Mieczyslaw Litwin, Jing Liu, Helle-Mai Loit, Luis Lopes, Tania Lopez, Esther López-García, Roberto Lorbeer, Paulo A. Lotufo, José Eugenio Lozano, Dalia Luksiene, Annamari Lundqvist, Robert Lundqvist, Nuno Lunet, Per Lytsy, Guansheng Ma, Jun Ma, George L. L. Machado-Coelho, Aristides M. Machado-Rodrigues, Suka Machi, Stefania Maggi, Dianna J. Magliano, Emmanuella Magriplis, Bernard Maire, Marjeta Majer, Marcia Makdisse, Fatemeh Malekzadeh, Reza Malekzadeh, Rahul Malhotra, Sofia Malyutina, Lynell V. Maniego, Yannis Manios, Jim I. Mann, Enzo Manzato, Paula Margozzini, Anastasia Markaki, Oonagh Markey, Eliza Markidou Ioannidou, Larissa Pruner Marques, Pedro Marques-Vidal, Jaume Marrugat, Rosemarie Martin, Yves Martin-Prevel, Reynaldo Martorell, Eva Martos, Stefano Marventano, Shariq R. Masoodi, Ellisiv B. Mathiesen, Prashant Mathur, Alicia Matijasevich, Tandi E. Matsha, Artur Mazur, Jean Claude N. Mbanya, Shelly R. McFarlane, Martin McKee, Stela McLachlan, Rachael M. McLean, Scott B. McLean, Breige A. McNulty, Safiah Md Yusof, Sounnia Mediene-Benchekor, Jurate Medzioniene, Parinaz Mehdipour, Aline Meirhaeghe, Jørgen Meisfjord, Christa Meisinger, Ana Maria B. Menezes, Geetha R. Menon, Gert B. M. Mensink, Alibek Mereke, Indrapal I. Meshram, Andres Metspalu, Haakon E. Meyer, Jie Mi, Kim F. Michaelsen, Nathalie Michels, Kairit Mikkel, Jody C. Miller, Cláudia S. Minderico, Juan Francisco Miquel, Daphne Mirkopoulou, Erkin Mirrakhimov, Marjeta Misigoj-Durakovic, Antonio Mistretta, Veronica Mocanu, Pietro A. Modesti, Sahar Saeeidi Moghaddam, Bahram Mohajer, Mostafa K. Mohamed, Kazem Mohammad, Noushin Mohammadifard, Viswanathan Mohan, Salim Mohanna, Muhammad Fadhli Mohd Yusoff, Farnam Mohebi, Marie Moitry, Drude Molbo, Line T. Møllehave, Niels C. Møller, Dénes Molnár, Amirabbas Momenan, Charles K. Mondo, Eric A. Monterrubio, Kotsedi Daniel K. Monyeki, Jin Soo Moon, Leila B. Moreira, Alain Morejon, Luis A. Moreno, Karen Morgan, Suzanne Morin, Erik Lykke Mortensen, George Moschonis, Malgorzata Mossakowska, Jorge Mota, Anabela Mota-Pinto, Mohammad Esmaeel Motlagh, Jorge Motta, Kelias P. Msyamboza, Thet Thet Mu, Magdalena Muc, Boban Mugoša, Maria Lorenza Muiesan, Parvina Mukhtorova, Martina Müller-Nurasyid, Neil Murphy, Jaakko Mursu, Elaine M. Murtagh, Sanja Music Milanovic, Vera Musil, Iraj Nabipour, Shohreh Naderimagham, Gabriele Nagel, Balkish M. Naidu, Harunobu Nakamura, Jana Námešná, Ei Ei K. Nang, Vinay B. Nangia, Martin Nankap, Sameer Narake, Paola Nardone, Matthias Nauck, Eva Maria Navarrete-Muñoz, William A. Neal, Keiu Nelis, Liis Nelis, Ilona Nenko, Flavio Nervi, Chung T. Nguyen, Nguyen D. Nguyen, Quang Ngoc Nguyen, Ramfis E. Nieto-Martínez, Guang Ning, Toshiharu Ninomiya, Sania Nishtar, Marianna Noale, Oscar A. Noboa, Teresa Norat, Sawada Norie, Davide Noto, Mohannad Al Nsour, Eha Nurk, Moffat Nyirenda, Galina Obreja, Angélica M. Ochoa-Avilés, Eiji Oda, Kyungwon Oh, Kumiko Ohara, Ryutaro Ohtsuka, Örn Olafsson, Maria Teresa Anselmo Olinto, Isabel O. Oliveira, Maciej Oltarzewski, Mohd Azahadi Omar, Altan Onat, Terence W. O’Neill, Sok King Ong, Lariane M. Ono, Pedro Ordunez, Dermot O’Reilly, Rui Ornelas, Ana P. Ortiz, Pedro J. Ortiz, Merete Osler, Clive Osmond, Sergej M. Ostojic, Afshin Ostovar, Johanna A. Otero, Kim Overvad, Ellis Owusu-Dabo, Fred Michel Paccaud, Cristina Padez, Ioannis Pagkalos, Elena Pahomova, Andrzej Pająk, Domenico Palli, Alberto Palloni, Luigi Palmieri, Wen-Harn Pan, Songhomitra Panda-Jonas, Arvind Pandey, Francesco Panza, Dimitrios Papandreou, Soon-Woo Park, Winsome R. Parnell, Mahboubeh Parsaeian, Ionela M. Pascanu, Nikhil D. Patel, Ivan Pecin, Mangesh S. Pednekar, Nasheeta Peer, Sergio Viana Peixoto, Markku Peltonen, Alexandre C. Pereira, Cynthia M. Pérez, Napoleon Perez-Farinos, Annette Peters, Astrid Petersmann, Janina Petkeviciene, Ausra Petrauskiene, Niloofar Peykari, Son Thai Pham, Daniela Pierannunzio, Iris Pigeot, Hynek Pikhart, Aida Pilav, Lorenza Pilotto, Francesco Pistelli, Freda Pitakaka, Aleksandra Piwonska, Pedro Plans-Rubió, Bee Koon Poh, Hermann Pohlabeln, Raluca M. Pop, Stevo R. Popovic, Miquel Porta, Marileen L. P. Portegies, Georg Posch, Dimitrios Poulimeneas, Hamed Pouraram, Akram Pourshams, Hossein Poustchi, Rajendra Pradeepa, Alison J. Price, Jacqueline F. Price, Jardena J. Puder, Iveta Pudule, Soile E. Puhakka, Maria Puiu, Margus Punab, Radwan F. Qasrawi, Mostafa Qorbani, Tran Quoc Bao, Madhari S. Radhika, Ivana Radic, Ricardas Radisauskas, Mahfuzar Rahman, Mahmudur Rahman, Olli Raitakari, Manu Raj, Hemalatha Rajkumar, Sherali Rakhmatulloev, Sudha Ramachandra Rao, Ambady Ramachandran, Jacqueline Ramke, Elisabete Ramos, Rafel Ramos, Lekhraj Rampal, Sanjay Rampal, Kodavanti Mallikharjuna Rao, Ramon A. Rascon-Pacheco, Mette Rasmussen, Josep Redon, Paul Ferdinand M. Reganit, Valéria Regecová, Luis Revilla, Lourdes Ribas-Barba, Robespierre Ribeiro, Elio Riboli, Fernando Rigo, Natascia Rinaldo, Tobias F. Rinke de Wit, Ana Rito, Raphael M. Ritti-Dias, Juan A. Rivera, Cynthia Robitaille, Daniela Rodrigues, Fernando Rodríguez-Artalejo, María del Cristo Rodriguez-Perez, Laura A. Rodríguez-Villamizar, Rosalba Rojas-Martinez, Nipa Rojroongwasinkul, Dora Romaguera, Annika Rosengren, Ian Rouse, Joel G. R. Roy, Adolfo Rubinstein, Frank J. Rühli, Jean-Bernard Ruidavets, Emma Ruiz Moreno, Blanca Sandra Ruiz-Betancourt, Paola Russo, Petra Rust, Marcin Rutkowski, Charumathi Sabanayagam, Harshpal S. Sachdev, Saeid Safiri, Olfa Saidi, Benoit Salanave, Eduardo Salazar-Martinez, Diego Salmerón, Veikko Salomaa, Jukka T. Salonen, Massimo Salvetti, Jose Sánchez-Abanto, Sandjaja, Susana Sans, Loreto Santa-Marina, Diana A. Santos, Ina S. Santos, Osvaldo Santos, Rute Santos, Sara Santos Sanz, Jouko L. Saramies, Luis B. Sardinha, Nizal Sarrafzadegan, Kai-Uwe Saum, Savvas Savva, Mathilde Savy, Marcia Scazufca, Angelika Schaffrath Rosario, Herman Schargrodsky, Anja Schienkiewitz, Karin Schindler, Sabine Schipf, Carsten O. Schmidt, Ida Maria Schmidt, Ben Schöttker, Constance Schultsz, Aletta E. Schutte, Sylvain Sebert, Aye Aye Sein, Rusidah Selamat, Vedrana Sember, Abhijit Sen, Idowu O. Senbanjo, Sadaf G. Sepanlou, Victor Sequera, Luis Serra-Majem, Jennifer Servais, Svetlana A. Shalnova, Sanjib K. Sharma, Jonathan E. Shaw, Lela Shengelia, Kenji Shibuya, Hana Shimizu-Furusawa, Dong Wook Shin, Youchan Shin, Alfonso Siani, Rosalynn Siantar, Abla M. Sibai, Antonio M. Silva, Diego Augusto Santos Silva, Mary Simon, Judith Simons, Leon A. Simons, Khairil Si-Ramlee, Agneta Sjöberg, Michael Sjöström, Jolanta Slowikowska-Hilczer, Przemyslaw Slusarczyk, Liam Smeeth, Marieke B. Snijder, Hung-Kwan So, Eugène Sobngwi, Stefan Söderberg, Moesijanti Y. E. Soekatri, Agustinus Soemantri, Vincenzo Solfrizzi, Emily Sonestedt, Yi Song, Thorkild I. A. Sørensen, Charles Sossa Jérome, Aïcha Soumaré, Angela Spinelli, Igor Spiroski, Jan A. Staessen, Hanspeter Stamm, Maria G. Stathopoulou, Kaspar Staub, Bill Stavreski, Jostein Steene-Johannessen, Peter Stehle, Aryeh D. Stein, George S. Stergiou, Jochanan Stessman, Doris Stöckl, Tanja Stocks, Jakub Stokwiszewski, Gareth Stratton, Karien Stronks, Maria Wany Strufaldi, Lela Sturua, Ramón Suárez-Medina, Chien-An Sun, Johan Sundström, Yn-Tz Sung, Jordi Sunyer, Paibul Suriyawongpaisal, Boyd A. Swinburn, Rody G. Sy, René Charles Sylva, Lucjan Szponar, E. Shyong Tai, Mari-Liis Tammesoo, Abdonas Tamosiunas, Eng Joo Tan, Xun Tang, Frank Tanser, Yong Tao, Mohammed Rasoul Tarawneh, Jakob Tarp, Carolina B. Tarqui-Mamani, Radka Taxová Braunerová, Anne Taylor, Félicité Tchibindat, William R. Tebar, Grethe Tell, Tania Tello, Holger Theobald, Xenophon Theodoridis, Lutgarde Thijs, Betina H. Thuesen, Lubica Tichá, Erik J. Timmermans, Anne Tjonneland, Hanna K. Tolonen, Janne S. Tolstrup, Murat Topbas, Roman Topór-Madry, María José Tormo, Michael J. Tornaritis, Maties Torrent, Stefania Toselli, Pierre Traissac, Dimitrios Trichopoulos, Antonia Trichopoulou, Oanh T. H. Trinh, Atul Trivedi, Yu-Hsiang Tsao, Lechaba Tshepo, Maria Tsigga, Shoichiro Tsugane, Marshall K. Tulloch-Reid, Fikru Tullu, Tomi-Pekka Tuomainen, Jaakko Tuomilehto, Maria L. Turley, Per Tynelius, Themistoklis Tzotzas, Christophe Tzourio, Peter Ueda, Eunice E. Ugel, Flora A. M. Ukoli, Hanno Ulmer, Belgin Unal, Hannu M. T. Uusitalo, Justina Vaitkeviciute, Gonzalo Valdivia, Susana Vale, Damaskini Valvi, Yvonne T. van der Schouw, Koen Van Herck, Hoang Van Minh, Lenie van Rossem, Natasja M. Van Schoor, Irene G. M. van Valkengoed, Dirk Vanderschueren, Diego Vanuzzo, Gregorio Varela-Moreiras, Patricia Varona-Pérez, Lars Vatten, Tomas Vega, Toomas Veidebaum, Gustavo Velasquez-Melendez, Biruta Velika, Giovanni Veronesi, W. M. Monique Verschuren, Cesar G. Victora, Giovanni Viegi, Lucie Viet, Paolo Vineis, Jesus Vioque, Jyrki K. Virtanen, Marjolein Visser, Sophie Visvikis-Siest, Bharathi Viswanathan, Tiina Vlasoff, Peter Vollenweider, Henry Völzke, Ari Voutilainen, Sari Voutilainen, Martine Vrijheid, Tanja G. M. Vrijkotte, Alisha N. Wade, Aline Wagner, Thomas Waldhör, Janette Walton, Wan Mohamad Wan Bebakar, Wan Nazaimoon Wan Mohamud, Rildo S. Wanderley, Ming-Dong Wang, Qian Wang, Xiangjun Wang, Ya Xing Wang, Ying-Wei Wang, S. Goya Wannamethee, Nicholas Wareham, Adelheid Weber, Deepa Weerasekera, Daniel Weghuber, Wenbin Wei, Peter H. Whincup, Kurt Widhalm, Indah S. Widyahening, Andrzej Wiecek, Alet H. Wijga, Rainford J. Wilks, Johann Willeit, Peter Willeit, Tom Wilsgaard, Bogdan Wojtyniak, Jyh Eiin Wong, Tien Yin Wong, Roy A. Wong-McClure, Jean Woo, Mark Woodward, Frederick C. Wu, Jianfeng Wu, Shouling Wu, Haiquan Xu, Liang Xu, Uruwan Yamborisut, Weili Yan, Ling Yang, Xiaoguang Yang, Yang Yang, Nazan Yardim, Mehdi Yaseri, Xingwang Ye, Panayiotis K. Yiallouros, Agneta Yngve, Moein Yoosefi, Akihiro Yoshihara, Qi Sheng You, San-Lin You, Novie O. Younger-Coleman, Ahmad Faudzi Yusoff, Luciana Zaccagni, Vassilis Zafiropulos, Farhad Zamani, Sabina Zambon, Antonis Zampelas, Hana Zamrazilová, Maria Elisa Zapata, Ko Ko Zaw, Tomasz Zdrojewski, Tajana Zeljkovic Vrkic, Yi Zeng, Dong Zhao, Wenhua Zhao, Wei Zheng, Yingfeng Zheng, Bekbolat Zholdin, Maigeng Zhou, Dan Zhu, Baurzhan Zhussupov, Esther Zimmermann, Julio Zuñiga Cisneros, Majid Ezzati

**Affiliations:** 1 Imperial College London, London, UK; 2 University of Kent, Canterbury, UK; 3 Middlesex University, London, UK; 4 University of California Berkeley, Berkeley, CA USA; 5 World Health Organization, Geneva, Switzerland; 6 Seoul National University, Seoul, South Korea; 7 University of Zagreb, Zagreb, Croatia; 8 US Centers for Disease Control and Prevention, Atlanta, GA USA; 9 Universidad Peruana Cayetano Heredia, Lima, Peru; 10 Aga Khan University, Karachi, Pakistan; 11 The Hospital for Sick Children, Toronto, Ontario Canada; 12 Harvard T. H. Chan School of Public Health, Boston, MA USA; 13 Ministry of Health, Victoria, Seychelles; 14 University of Lausanne, Lausanne, Switzerland; 15 Victor Babeş University of Medicine and Pharmacy Timisoara, Timisoara, Romania; 16 The University of the West Indies, Cave Hill, Barbados; 17 University of Sydney, Sydney, New South Wales Australia; 18 National Institutes of Biomedical Innovation, Health and Nutrition, Tokyo, Japan; 19 South African Medical Research Council, Cape Town, South Africa; 20 ICMR-National Institute of Nutrition, Hyderabad, India; 21 Brown University, Providence, RI USA; 22 Ain Shams University, Cairo, Egypt; 23 Karolinska Institutet, Stockholm, Sweden; 24 University of Ljubljana, Ljubljana, Slovenia; 25 Ministry of Health Malaysia, Kuala Lumpur, Malaysia; 26 Caja Costarricense de Seguro Social, San José, Costa Rica; 27 Al-Quds University, East Jerusalem, Palestine; 28 National Center of Public Healthcare, Nur-Sultan, Kazakhstan; 29 Center for Diabetes and Endocrine Care, Srinagar, India; 30 Usmanu Danfodiyo University Teaching Hospital, Sokoto, Nigeria; 31 Birzeit University, Birzeit, Palestine; 32 Instituto Mexicano del Seguro Social, Mexico City, Mexico; 33 Flinders University, Adelaide, South Australia Australia; 34 Mahidol University, Nakhon Pathom, Thailand; 35 BRAC University, Dhaka, Bangladesh; 36 Food and Nutrition Research Institute, Taguig, The Philippines; 37 Instituto Nacional de Ciencias Médicas y Nutrición, Mexico City, Mexico; 38 University of Amsterdam, Amsterdam, The Netherlands; 39 Tehran University of Medical Sciences, Tehran, Iran; 40 Non-Communicable Diseases Research Center, Tehran, Iran; 41 University of Bremen, Bremen, Germany; 42 The National Center for Diabetes, Endocrinology and Genetics, Amman, Jordan; 43 Aldara Hospital and Medical Center, Riyadh, Saudi Arabia; 44 King Abdullah International Medical Research Center, Riyadh, Saudi Arabia; 45 King Saud University, Riyadh, Saudi Arabia; 46 Universiti Malaysia Sabah, Kota Kinabalu, Malaysia; 47 Luxembourg Institute of Health, Strassen, Luxembourg; 48 Kuwait Institute for Scientific Research, Safat, Kuwait; 49 King Abdulaziz University, Jeddah, Saudi Arabia; 50 ISGlobal Centre for Research in Environmental Epidemiology, Barcelona, Spain; 51 World Health Organization Regional Office for the Eastern Mediterranean, Cairo, Egypt; 52 Bombay Hospital and Medical Research Centre, Mumbai, India; 53 University of Lille, Lille, France; 54 Lille University Hospital, Lille, France; 55 London School of Hygiene & Tropical Medicine, London, UK; 56 Western Norway University of Applied Sciences, Sogndal, Norway; 57 Norwegian School of Sport Sciences, Oslo, Norway; 58 University of Copenhagen, Copenhagen, Denmark; 59 Madras Diabetes Research Foundation, Chennai, India; 60 Zahedan University of Medical Sciences, Zahedan, Iran; 61 National Institute of Public Health, Tunis, Tunisia; 62 Institute of Public Health of the University of Porto, Porto, Portugal; 63 Norwegian Institute of Public Health, Oslo, Norway; 64 University of Massachusetts, Amherst, MA USA; 65 Abt Associates, Kathmandu, Nepal; 66 University of Iceland, Reykjavik, Iceland; 67 University of Yaoundé 1, Yaoundé, Cameroon; 68 Federal University of Pelotas, Pelotas, Brazil; 69 University of Medicine 1, Yangon, Myanmar; 70 University of Oulu, Oulu, Finland; 71 Regional Authority of Public Health, Banska Bystrica, Slovakia; 72 University of Porto Medical School, Porto, Portugal; 73 Shahid Beheshti University of Medical Sciences, Tehran, Iran; 74 Indian Council of Medical Research, New Delhi, India; 75 Bispebjerg and Frederiksberg Hospital, Copenhagen, Denmark; 76 University of Science and Technology, Sana’a, Yemen; 77 Medical University of Lodz, Lodz, Poland; 78 Medical University of Gdansk, Gdansk, Poland; 79 Universidad Autónoma de Madrid, Madrid, Spain; 80 University of Palermo, Palermo, Italy; 81 Pan American Health Organization, Washington, DC USA; 82 Mohammed V University de Rabat, Rabat, Morocco; 83 University of Pernambuco, Recife, Brazil; 84 Dalhousie University, Halifax, Nova Scotia Canada; 85 Jordan University of Science and Technology, Irbid, Jordan; 86 Federal University of Maranhao, Sao Luis, Brazil; 87 Kazakh National Medical University, Almaty, Kazakhstan; 88 University of Auckland, Auckland, New Zealand; 89 University College Dublin, Dublin, Ireland; 90 University Tunis El Manar, Tunis, Tunisia; 91 Federal Ministry of Labour, Social Affairs, Health and Consumer Protection, Vienna, Austria; 92 Cafam University Foundation, Bogota, Colombia; 93 Lithuanian University of Health Sciences, Kaunas, Lithuania; 94 University of São Paulo, São Paulo, Brazil; 95 B. J. Medical College, Ahmedabad, India; 96 Chirayu Medical College, New Delhi, India; 97 Sunder Lal Jain Hospital, Delhi, India; 98 Shandong University of Traditional Chinese Medicine, Shandong, China; 99 Shanghai Jiao-Tong University School of Medicine, Shanghai, China; 100 Institute of Medical Research and Medicinal Plant Studies, Yaoundé, Cameroon; 101 Ufa Eye Research Institute, Ufa, Russia; 102 Nepal Health Research Council, Kathmandu, Nepal; 103 University of Montenegro, Niksic, Montenegro; 104 University of Greenland, Nuuk, Greenland; 105 University of Southern Denmark, Odense, Denmark; 106 University of Oslo, Oslo, Norway; 107 University of Gothenburg, Gothenburg, Sweden; 108 National Institute for Public Health and the Environment, Bilthoven, The Netherlands; 109 University of Turin, Turin, Italy; 110 University College London, London, UK; 111 Liverpool John Moores University, Liverpool, UK; 112 Nanyang Technological University, Singapore, Singapore; 113 German Institute of Human Nutrition, Potsdam, Germany; 114 Universidad de la República, Montevideo, Uruguay; 115 CEMIC, Buenos Aires, Argentina; 116 IRCCS Neuromed, Pozzilli, Italy; 117 Toulouse University School of Medicine, Toulouse, France; 118 University of Zurich, Zurich, Switzerland; 119 University Hospital of Varese, Varese, Italy; 120 University Hospital KU Leuven, Leuven, Belgium; 121 Flemish Agency for Care and Health, Brussels, Belgium; 122 Ghent University, Ghent, Belgium; 123 FrieslandCampina, Amersfoort, The Netherlands; 124 Universidad Central de Venezuela, Caracas, Venezuela; 125 Bielefeld University, Bielefeld, Germany; 126 World Health Organization Regional Office for Europe, Copenhagen, Denmark; 127 German Cancer Research Center, Heidelberg, Germany; 128 The Fred Hollows Foundation, Auckland, New Zealand; 129 University of Medicine and Pharmacy Bucharest, Bucharest, Romania; 130 University College Copenhagen, Copenhagen, Denmark; 131 Institute of Public Health, Tirana, Albania; 132 Cork Institute of Technology, Cork, Ireland; 133 Universidad de La Laguna, Tenerife, Spain; 134 University of Malta, Pietà, Malta; 135 Vanderbilt University, Nashville, TN USA; 136 Ministry of Health, Tongatapu, Tonga; 137 Canadian Fitness and Lifestyle Research Institute, Ottawa, Ontario Canada; 138 Hospital Santa Maria, Lisbon, Portugal; 139 Karadeniz Technical University, Trabzon, Turkey; 140 Istanbul University, Istanbul, Turkey; 141 Universidade Federal de Juiz de Fora, Juiz de Fora, Brazil; 142 Ministry of Public Health, Asunción, Paraguay; 143 Cardiologia di Mercato S. Severino Hospital, Mercato San Severino, Italy; 144 Karolinska Institutet, Huddinge, Sweden; 145 Centro de Estudios sobre Nutrición Infantil, Buenos Aires, Argentina; 146 University of Porto, Porto, Portugal; 147 Santiago de Compostela University, Santiago de Compostela, Spain; 148 Associazione Calabrese di Epatologia, Reggio Calabria, Italy; 149 Ministry of Health, Ankara, Turkey; 150 Food and Agriculture Organization of the United Nations, Rome, Italy; 151 Federal University of Rio Grande, Rio Grande, Brazil; 152 India Diabetes Research Foundation, Chennai, India; 153 Duke-NUS Medical School, Singapore, Singapore; 154 National Institute of Medical Statistics, New Delhi, India; 155 Academia Sinica, Taipei, Taiwan; 156 Capital Institute of Pediatrics, Beijing, China; 157 Duke University, Durham, NC USA; 158 Kailuan General Hospital, Tangshan, China; 159 University of Oxford, Oxford, UK; 160 The Gertner Institute for Epidemiology and Health Policy Research, Ramat Gan, Israel; 161 National Centre of Public Health and Analyses, Sofia, Bulgaria; 162 University of Bern, Lausanne, Switzerland; 163 Ministry of Health and Welfare, Taipei, Taiwan; 164 Murcia Health Council, Murcia, Spain; 165 Seoul National University College of Medicine, Seoul, South Korea; 166 Korea Centers for Disease Control and Prevention, Cheongju-si, South Korea; 167 Universidade Estadual Paulista, Presidente Prudente, Brazil; 168 Medical University of Silesia, Katowice, Poland; 169 Charles University in Prague, Prague, Czech Republic; 170 Thomayer Hospital, Prague, Czech Republic; 171 Primary Health Care, Floriana, Malta; 172 Carol Davila University of Medicine and Pharmacy, Bucharest, Romania; 173 Katholieke Universiteit Leuven, Leuven, Belgium; 174 Statistics Canada, Ottawa, Ontario, Canada; 175 Agency for Preventive and Social Medicine, Bregenz, Austria; 176 Universidade Federal de Santa Catarina, Florianópolis, Brazil; 177 University of Southampton, Southampton, UK; 178 Institut Pasteur de Lille, Lille, France; 179 Malawi Epidemiology and Intervention Research Unit, Lilongwe, Malawi; 180 CIBEROBN, Madrid, Spain; 181 National Institute of Public Health, Bucharest, Romania; 182 Eduardo Mondlane University, Maputo, Mozambique; 183 National Council of Research, Reggio Calabria, Italy; 184 Indian Statistical Institute, Kolkata, India; 185 Tabriz Health Services Management Centre, Tabriz, Iran; 186 Sciensano, Brussels, Belgium; 187 Academic Medical Center of University of Amsterdam, Amsterdam, The Netherlands; 188 National Research Centre for Preventive Medicine, Moscow, Russia; 189 Erasmus Medical Center Rotterdam, Rotterdam, The Netherlands; 190 University of Montreal, Montreal, Québec, Canada; 191 Institut de Recherche pour le Développement, Montpellier, France; 192 French Public Health Agency, St Maurice, France; 193 Mediterranea Cardiocentro, Naples, Italy; 194 Universidade do Vale do Rio dos Sinos, São Leopoldo, Brazil; 195 National Council of Scientific and Technical Research, Tandil, Argentina; 196 Ministry of Health and Medical Education, Tehran, Iran; 197 National Institute of Nutrition, Hanoi, Vietnam; 198 University of Queensland, Brisbane, Queensland Australia; 199 Istituto Superiore di Sanità, Rome, Italy; 200 Universidad de Cuenca, Cuenca, Ecuador; 201 Helmholtz Zentrum München, Munich, Germany; 202 Ministère de la Santé et de la Lutte Contre le Sida, Abidjan, Côte d’Ivoire; 203 The Cardinal Wyszynski Institute of Cardiology, Warsaw, Poland; 204 Beijing Center for Disease Prevention and Control, Beijing, China; 205 BIDMC, Boston, MA USA; 206 University of Latvia, Riga, Latvia; 207 Ministry of Health and Medical Services, Gizo, Solomon Islands; 208 University of Benin, Benin City, Nigeria; 209 University of Skövde, Skövde, Sweden; 210 National Institute of Nutrition and Food Technology, Tunis, Tunisia; 211 The University of the West Indies, Kingston, Jamaica; 212 University of California Davis, Davis, CA USA; 213 University of Stellenbosch, Cape Town, South Africa; 214 National Institute for Health and Welfare, Helsinki, Finland; 215 Queen’s University of Belfast, Belfast, UK; 216 Shiraz University of Medical Sciences, Shiraz, Iran; 217 Centro de Salud Villanueva Norte, Badajoz, Spain; 218 Hospital Don Benito-Villanueva de la Serena, Badajoz, Spain; 219 Ministry of Health, Buenos Aires, Argentina; 220 Council for Agricultural Research and Economics, Rome, Italy; 221 Pontificia Universidad Católica de Chile, Santiago, Chile; 222 Institute of Mother and Child, Warsaw, Poland; 223 University of Basel, Basel, Switzerland; 224 Swiss TPH, Basel, Switzerland; 225 University of Tartu, Tartu, Estonia; 226 Instituto Nacional de Salud Pública, Cuernavaca, Mexico; 227 Universiti Sains Malaysia, Kelantan, Malaysia; 228 Umeå University, Umeå, Sweden; 229 Georgia College and State University, Milledgeville, GA USA; 230 Federal University of São Paulo, São Paulo, Brazil; 231 Hospital Universitario Son Espases, Palma, Spain; 232 Hospital de Clinicas de Porto Alegre, Porto Alegre, Brazil; 233 Universidade Federal do Rio Grande do Sul, Porto Alegre, Brazil; 234 Kindai University, Osaka-Sayama, Japan; 235 Kyoto University, Kyoto, Japan; 236 Medical University of Warsaw, Warsaw, Poland; 237 Ministry of Health, Rome, Italy; 238 University of Catania, Catania, Italy; 239 CIBER en Epidemiología y Salud Pública, Alicante, Spain; 240 Africa Health Research Institute, Mtubatuba, South Africa; 241 Geneva University Hospitals, Geneva, Switzerland; 242 CIBER en Epidemiología y Salud Pública, Barcelona, Spain; 243 Australian Bureau of Statistics, Canberra, Australian Capital Territory Australia; 244 Universidade Federal de Minas Gerais, Belo Horizonte, Brazil; 245 Wageningen University, Wageningen, The Netherlands; 246 University of Insubria, Varese, Italy; 247 University of Adelaide, Adelaide, South Australia Australia; 248 Lund University, Lund, Sweden; 249 Ministry of Health, Jerusalem, Israel; 250 McGill University, Montreal, Québec Canada; 251 Universidad Autonoma de Santo Domingo, Santo Domingo, Dominican Republic; 252 Universidad Politécnica de Madrid, Madrid, Spain; 253 The Andes Clinic of Cardio-Metabolic Studies, Merida, Venezuela; 254 Instituto Nacional de Higiene, Epidemiología y Microbiología, Havana, Cuba; 255 Ministry of Health, Lisbon, Portugal; 256 Institute for Clinical and Experimental Medicine, Prague, Czech Republic; 257 Children’s Memorial Health Institute, Warsaw, Poland; 258 Aristotle University of Thessaloniki, Thessaloniki, Greece; 259 Jagiellonian University Medical College, Kraków, Poland; 260 National Center of Cardiovascular Diseases, Beijing, China; 261 University of Ferrara, Ferrara, Italy; 262 Icelandic Heart Association, Kopavogur, Iceland; 263 Universidad Icesi, Cali, Colombia; 264 State University of Montes Claros, Montes Claros, Brazil; 265 King’s College London, London, UK; 266 International Agency for Research on Cancer, Lyon, France; 267 Capital Medical University, Beijing, China; 268 Healis-Sekhsaria Institute for Public Health, Navi Mumbai, India; 269 Eternal Heart Care Centre and Research Institute, Jaipur, India; 270 University of Ibadan, Ibadan, Nigeria; 271 Institute for Clinical Effectiveness and Health Policy, Buenos Aires, Argentina; 272 Research and Education Institute of Child Health, Nicosia, Cyprus; 273 Danish Cancer Society Research Centre, Copenhagen, Denmark; 274 Alexander Technological Educational Institute, Thessaloniki, Greece; 275 Kyushu University, Fukuoka, Japan; 276 University of Bergen, Bergen, Norway; 277 Tulane University, New Orleans, LA USA; 278 Chinese Center for Disease Control and Prevention, Beijing, China; 279 University of Pécs, Pécs, Hungary; 280 Danish Health Authority, Copenhagen, Denmark; 281 Joep Lange Institute, Amsterdam, The Netherlands; 282 Oulu University Hospital, Oulu, Finland; 283 Universidad Autónoma de Bucaramanga, Bucaramanga, Colombia; 284 ETH Zurich, Zurich, Switzerland; 285 Chronic Diseases Research Center, Tehran, Iran; 286 University of Hong Kong, Hong Kong, China; 287 The Chinese University of Hong Kong, Hong Kong, China; 288 University of Western Australia, Perth, Western Australia Australia; 289 Kingston Health Sciences Centre, Kingston, Ontario, Canada; 290 Heart Institute, São Paulo, Brazil; 291 Fundación Oftalmológica de Santander, Santander, Colombia; 292 University Oran 1, Oran, Algeria; 293 Independent Public Health Specialist, Nay Pyi Taw, Myanmar; 294 Ministry of Health and Sports, Nay Pyi Taw, Myanmar; 295 Peking University, Beijing, China; 296 CIBER en Epidemiología y Salud Pública, Murcia, Spain; 297 Amsterdam UMC of University of Amsterdam, Amsterdam, The Netherlands; 298 Vrije Universiteit Amsterdam, Amsterdam, The Netherlands; 299 American University of Beirut, Beirut, Lebanon; 300 CIBER en Epidemiología y Salud Pública, San Sebastian, Spain; 301 Cairo University, Cairo, Egypt; 302 The University of Tokyo, Tokyo, Japan; 303 University Hospital Centre Zagreb, Zagreb, Croatia; 304 Niigata University, Niigata, Japan; 305 Hadassah University Medical Center, Jerusalem, Israel; 306 Norwegian University of Science and Technology, Trondheim, Norway; 307 The University of Melbourne, Melbourne, Victoria, Australia; 308 Sports University of Tirana, Tirana, Albania; 309 University of Zagreb School of Medicine, Zagreb, Croatia; 310 Heart Foundation, Melbourne, Victoria, Australia; 311 National Health Insurance Service, Wonju, South Korea; 312 Guangzhou 12th Hospital, Guangzhou, China; 313 Universidad Eugenio Maria de Hostos, Santo Domingo, Dominican Republic; 314 Simon Fraser University, Burnaby, British Columbia Canada; 315 Ruprecht-Karls-University of Heidelberg, Heidelberg, Germany; 316 World Health Organization Country Office, Delhi, India; 317 Institute of Public Health of Serbia, Belgrade, Serbia; 318 University of Opole, Opole, Poland; 319 University of Crete, Heraklion, Greece; 320 Universiti Kebangsaan Malaysia, Kuala Lumpur, Malaysia; 321 Hellenic Medical Association for Obesity, Athens, Greece; 322 Maharajgunj Medical Campus, Kathmandu, Nepal; 323 Aarhus University, Aarhus, Denmark; 324 Johns Hopkins Bloomberg School of Public Health, Baltimore, MD USA; 325 Pennington Biomedical Research Center, Baton Rouge, LA USA; 326 University of Eastern Finland, Kuopio, Finland; 327 National Institute of Epidemiology, Chennai, India; 328 University of Münster, Münster, Germany; 329 Research Institute for Primordial Prevention of Non-communicable Disease, Isfahan, Iran; 330 Amsterdam Public Health Research Institute, Amsterdam, The Netherlands; 331 Kyrgyz State Medical Academy, Bishkek, Kyrgyzstan; 332 Research Institute of Child Nutrition, Dortmund, Germany; 333 University of Cambridge, Cambridge, UK; 334 Hypertension Research Center, Isfahan, Iran; 335 Medical University of Innsbruck, Innsbruck, Austria; 336 Muhimbili University of Health and Allied Sciences, Dar es Salaam, Tanzania; 337 National Cancer Center, Goyang-si, South Korea; 338 Statistics Austria, Vienna, Austria; 339 B. P. Koirala Institute of Health Sciences, Dharan, Nepal; 340 University of Vienna, Vienna, Austria; 341 Oulu Deaconess Institute Foundation, Oulu, Finland; 342 Tartu University Clinics, Tartu, Estonia; 343 Kansai Medical University, Osaka-Sayama, Japan; 344 National Institute of Pharmacy and Nutrition, Budapest, Hungary; 345 Ministry of Health and Quality of Life, Port Louis, Mauritius; 346 Polish Academy of Sciences Anthropology Unit, Wroclaw, Poland; 347 University Hospital Ulm, Ulm, Germany; 348 University of Groningen, Groningen, The Netherlands; 349 North-West University, Potchefstroom, South Africa; 350 National Institute of Public Health, Prague, Czech Republic; 351 University of Jyväskylä, Jyväskylä, Finland; 352 Institute of Public Health of Montenegro, Podgorica, Montenegro; 353 Amrita Institute of Medical Sciences, Cochin, India; 354 Institute of Endocrinology, Prague, Czech Republic; 355 All India Institute of Medical Sciences, New Delhi, India; 356 African Population and Health Research Center, Nairobi, Kenya; 357 Hanoi University of Public Health, Hanoi, Vietnam; 358 Higher Institute of Nursing Professions and Technical Health, Rabat, Morocco; 359 National Institute of Public Health of Algeria, Algiers, Algeria; 360 Croatian National Institute of Public Health, Zagreb, Croatia; 361 Sahlgrenska Academy, Gothenburg, Sweden; 362 Endocrinology and Metabolism Research Center, Tehran, Iran; 363 University of Public Health, Yangon, Myanmar; 364 International Food Policy Research Institute, Dakar, Senegal; 365 National University of Singapore, Singapore, Singapore; 366 Hong Kong Polytechnic University, Hong Kong, China; 367 Tampere University Hospital, Tampere, Finland; 368 University of Douala, Douala, Cameroon; 369 University of Cape Town, Cape Town, South Africa; 370 West Virginia University, Morgantown, WV USA; 371 Oswaldo Cruz Foundation Rene Rachou Research Institute, Belo Horizonte, Brazil; 372 National Taiwan University, Taipei, Taiwan; 373 University of Chinese Academy of Sciences, Shanghai, China; 374 Uppsala University, Uppsala, Sweden; 375 Capital Medical University Beijing An Zhen Hospital, Beijing, China; 376 National Institute for Health Development, Tallinn, Estonia; 377 Universidad San Martín de Porres, Lima, Peru; 378 University Medicine of Greifswald, Greifswald, Germany; 379 Consejería de Sanidad Junta de Castilla y León, Valladolid, Spain; 380 Norrbotten County Council, Luleå, Sweden; 381 University of Uppsala, Uppsala, Sweden; 382 Universidade Federal de Ouro Preto, Ouro Preto, Brazil; 383 University of Coimbra, Coimbra, Portugal; 384 The Jikei University School of Medicine, Tokyo, Japan; 385 National Research Council, Padua, Italy; 386 Baker Heart and Diabetes Institute, Melbourne, Victoria, Australia; 387 Agricultural University of Athens, Athens, Greece; 388 Hospital Israelita Albert Einstein, São Paulo, Brazil; 389 Institute of Internal and Preventive Medicine, Novosibirsk, Russia; 390 Harokopio University, Athens, Greece; 391 University of Otago, Dunedin, New Zealand; 392 University of Padua, Padua, Italy; 393 Technological Educational Institute of Crete, Heraklion, Greece; 394 Loughborough University, Loughborough, UK; 395 Ministry of Health, Nicosia, Cyprus; 396 Lausanne University Hospital, Lausanne, Switzerland; 397 CIBERCV, Barcelona, Spain; 398 Mary Immaculate College, Limerick, Ireland; 399 Emory University, Atlanta, GA USA; 400 Hungarian Society of Sports Medicine, Budapest, Hungary; 401 Sher-i-Kashmir Institute of Medical Sciences, Srinagar, India; 402 UiT The Arctic University of Norway, Tromsø, Norway; 403 National Centre for Disease Informatics and Research, New Delhi, India; 404 Cape Peninsula University of Technology, Cape Town, South Africa; 405 University of Rzeszow, Rzeszow, Poland; 406 University of Edinburgh, Edinburgh, UK; 407 International Medical University, Shah Alam, Malaysia; 408 Institut National de la Santé et de la Recherche Médicale, Lille, France; 409 Robert Koch Institute, Berlin, Germany; 410 Lusófona University, Lisbon, Portugal; 411 Democritus University, Alexandroupolis, Greece; 412 Grigore T. Popa University of Medicine and Pharmacy, Iasi, Romania; 413 Università degli Studi di Firenze, Florence, Italy; 414 Isfahan Cardiovascular Research Center, Isfahan, Iran; 415 Strasbourg University Hospital, Strasbourg, France; 416 University of Strasbourg, Strasbourg, France; 417 Mulago Hospital, Kampala, Uganda; 418 Instituto Nacional de Salud Pública, Mexico City, Mexico; 419 University of Limpopo, Sovenga, South Africa; 420 Seoul National University Children’s Hospital, Seoul, South Korea; 421 University Medical Science, Havana, Cuba; 422 Universidad de Zaragoza, Zaragoza, Spain; 423 RCSI, Dublin, Ireland; 424 La Trobe University, Melbourne, Victoria Australia; 425 International Institute of Molecular and Cell Biology, Warsaw, Poland; 426 Ahvaz Jundishapur University of Medical Sciences, Ahvaz, Iran; 427 Gorgas Memorial Institute of Public Health, Panama City, Panama; 428 World Health Organization Country Office, Lilongwe, Malawi; 429 Department of Public Health, Nay Pyi Taw, Myanmar; 430 University of Brescia, Brescia, Italy; 431 Ministry of Health and Social Protection, Dushanbe, Tajikistan; 432 Bushehr University of Medical Sciences, Bushehr, Iran; 433 Ulm University, Ulm, Germany; 434 Kobe University, Kobe, Japan; 435 Suraj Eye Institute, Nagpur, India; 436 UNICEF, Yaoundé, Cameroon; 437 National Institute of Hygiene and Epidemiology, Hanoi, Vietnam; 438 University of Pharmacy and Medicine, Ho Chi Minh City, Vietnam; 439 Hanoi Medical University, Hanoi, Vietnam; 440 Miami Veterans Affairs Healthcare System, Miami, FL USA; 441 Heartfile, Islamabad, Pakistan; 442 National Cancer Center, Tokyo, Japan; 443 Eastern Mediterranean Public Health Network, Amman, Jordan; 444 State University of Medicine and Pharmacy, Chisinau, Moldova; 445 Tachikawa General Hospital, Nagaoka, Japan; 446 Japan Wildlife Research Center, Tokyo, Japan; 447 University of Vale do Rio dos Sinos, São Leopoldo, Brazil; 448 National Food and Nutrition Institute, Warsaw, Poland; 449 University of Manchester, Manchester, UK; 450 Ministry of Health, Bandar Seri Begawan, Brunei Darussalam; 451 University of Madeira, Funchal, Portugal; 452 University of Puerto Rico, San Juan, Puerto Rico; 453 Research Center for Prevention and Health, Glostrup, Denmark; 454 MRC Lifecourse Epidemiology Unit, Southampton, UK; 455 University of Novi Sad, Novi Sad, Serbia; 456 Kwame Nkrumah University of Science and Technology, Kumasi, Ghana; 457 Institute for Social and Preventive Medicine, Lausanne, Switzerland; 458 Cancer Prevention and Research Institute, Florence, Italy; 459 University of Wisconsin-Madison, Madison, WI USA; 460 IRCCS Casa Sollievo della Sofferenza, Bari, Italy; 461 Zayed University, Abu Dhabi, United Arab Emirates; 462 Catholic University of Daegu, Daegu, South Korea; 463 University of Medicine, Pharmacy, Science and Technology of Târgu Mureş, Târgu Mureş, Romania; 464 Jivandeep Hospital, Anand, India; 465 South African Medical Research Council, Durban, South Africa; 466 Spanish Food Safety and Nutrition Agency, Madrid, Spain; 467 Vietnam National Heart Institute, Hanoi, Vietnam; 468 Leibniz Institute for Prevention Research and Epidemiology - BIPS, Bremen, Germany; 469 University of Sarajevo, Sarajevo, Bosnia and Herzegovina; 470 Cardiovascular Prevention Centre Udine, Udine, Italy; 471 University Hospital of Pisa, Pisa, Italy; 472 Ministry of Health and Medical Services, Honiara, Solomon Islands; 473 Public Health Agency of Catalonia, Barcelona, Spain; 474 Institut Hospital del Mar d’Investigacions Mèdiques, Barcelona, Spain; 475 Digestive Oncology Research Center, Tehran, Iran; 476 Digestive Disease Research Institute, Tehran, Iran; 477 Centre for Disease Prevention and Control, Riga, Latvia; 478 Alborz University of Medical Sciences, Karaj, Iran; 479 Ministry of Health, Hanoi, Vietnam; 480 BRAC, Dhaka, Bangladesh; 481 Institute of Epidemiology Disease Control and Research, Dhaka, Bangladesh; 482 University of Turku, Turku, Finland; 483 Institut Universitari d’Investigació en Atenció Primària Jordi Gol, Girona, Spain; 484 Universiti Putra Malaysia, Serdang, Malaysia; 485 University of Malaya, Kuala Lumpur, Malaysia; 486 National Institute of Public Health, Copenhagen, Denmark; 487 University of Valencia, Valencia, Spain; 488 University of the Philippines, Manila, The Philippines; 489 Slovak Academy of Sciences, Bratislava, Slovakia; 490 Nutrition Research Foundation, Barcelona, Spain; 491 Minas Gerais State Secretariat for Health, Belo Horizonte, Brazil; 492 Health Center San Agustín, Palma, Spain; 493 PharmAccess Foundation, Amsterdam, The Netherlands; 494 National Institute of Health Doutor Ricardo Jorge, Lisbon, Portugal; 495 Universidade Nove de Julho, São Paulo, Brazil; 496 Public Health Agency of Canada, Ottawa, Ontario Canada; 497 Canarian Health Service, Tenerife, Spain; 498 Universidad Industrial de Santander, Santander, Colombia; 499 Sahlgrenska University Hospital, Gothenburg, Sweden; 500 Fiji National University, Suva, Fiji; 501 Spanish Nutrition Foundation, Madrid, Spain; 502 Institute of Food Sciences of the National Research Council, Avellino, Italy; 503 Singapore Eye Research Institute, Singapore, Singapore; 504 Sitaram Bhartia Institute of Science and Research, New Delhi, India; 505 Maragheh University of Medical Sciences, Maragheh, Iran; 506 University of Helsinki, Helsinki, Finland; 507 National Institute of Health, Lima, Peru; 508 Ministry of Health, Jakarta, Indonesia; 509 Catalan Department of Health, Barcelona, Spain; 510 Biodonostia Health Research Institute, San Sebastian, Spain; 511 Universidade de Lisboa, Lisbon, Portugal; 512 South Karelia Social and Health Care District, Lappeenranta, Finland; 513 Cardiovascular Research Institute, Isfahan, Iran; 514 University of São Paulo Clinics Hospital, São Paulo, Brazil; 515 Hospital Italiano de Buenos Aires, Buenos Aires, Argentina; 516 Medical University of Vienna, Vienna, Austria; 517 Rigshospitalet, Copenhagen, Denmark; 518 Lagos State University College of Medicine, Lagos, Nigeria; 519 University of Las Palmas de Gran Canaria, Las Palmas de Gran Canaria, Spain; 520 National Center for Disease Control and Public Health, Tbilisi, Georgia; 521 National Center for Global Health and Medicine, Tokyo, Japan; 522 Samsung Medical Center, Seoul, South Korea; 523 St Vincent’s Hospital, Sydney, New South Wales Australia; 524 University of New South Wales, Sydney, New South Wales Australia; 525 Health Polytechnic Jakarta II Institute, Jakarta, Indonesia; 526 Diponegoro University, Semarang, Indonesia; 527 University of Bari, Bari, Italy; 528 Institut Régional de Santé Publique, Ouidah, Benin; 529 University of Bordeaux, Bordeaux, France; 530 Institute of Public Health, Skopje, Macedonia; 531 University of Leuven, Leuven, Belgium; 532 Lamprecht und Stamm Sozialforschung und Beratung AG, Zurich, Switzerland; 533 INSERM, Nancy, France; 534 Bonn University, Bonn, Germany; 535 Sotiria Hospital, Sotiria, Greece; 536 National Institute of Public Health-National Institute of Hygiene, Warsaw, Poland; 537 Swansea University, Swansea, UK; 538 Fu Jen Catholic University, Taipei, Taiwan; 539 National Statistic Office of Cabo Verde, Praia, Cabo Verde; 540 University of KwaZulu-Natal, Mtubatuba, South Africa; 541 Ministry of Health, Amman, Jordan; 542 Comenius University, Bratislava, Slovakia; 543 Health Service of Murcia, Murcia, Spain; 544 IB-SALUT Area de Salut de Menorca, Maó, Spain; 545 University of Bologna, Bologna, Italy; 546 Hellenic Health Foundation, Athens, Greece; 547 Government Medical College, Bhavnagar, India; 548 Sefako Makgatho Health Science University, Ga-Rankuwa, South Africa; 549 Addis Ababa University, Addis Ababa, Ethiopia; 550 Dasman Diabetes Institute, Kuwait City, Kuwait; 551 Ministry of Health, Wellington, New Zealand; 552 Universidad Centro-Occidental Lisandro Alvarado, Barquisimeto, Venezuela; 553 Meharry Medical College, Nashville, TN USA; 554 Dokuz Eylul University, Izmir, Turkey; 555 University of Tampere Tays Eye Center, Tampere, Finland; 556 Polytechnic Institute of Porto, Porto, Portugal; 557 Utrecht University, Utrecht, The Netherlands; 558 University Medical Center Utrecht, Utrecht, The Netherlands; 559 National Research Council, Pisa, Italy; 560 Universidad Miguel Hernandez, Alicante, Spain; 561 Ministry of Health, Mont Fleuri, Seychelles; 562 North Karelian Center for Public Health, Joensuu, Finland; 563 University of the Witwatersrand, Johannesburg, South Africa; 564 Institute for Medical Research, Kuala Lumpur, Malaysia; 565 Xinjiang Medical University, Urumqi, China; 566 Shanghai Educational Development Co. Ltd, Shanghai, China; 567 Paracelsus Medical University, Salzburg, Austria; 568 St George’s, University of London, London, UK; 569 Universitas Indonesia, Jakarta, Indonesia; 570 Institute of Food and Nutrition Development of Ministry of Agriculture, Beijing, China; 571 Children’s Hospital of Fudan University, Shanghai, China; 572 University of Cyprus, Nicosia, Cyprus; 573 Iran University of Medical Sciences, Tehran, Iran; 574 West Kazakhstan State Medical University, Aktobe, Kazakhstan; 575 Inner Mongolia Medical University, Hohhot, China

**Keywords:** Risk factors, Epidemiology

## Abstract

Body-mass index (BMI) has increased steadily in most countries in parallel with a rise in the proportion of the population who live in cities^1,2^. This has led to a widely reported view that urbanization is one of the most important drivers of the global rise in obesity^3–6^. Here we use 2,009 population-based studies, with measurements of height and weight in more than 112 million adults, to report national, regional and global trends in mean BMI segregated by place of residence (a rural or urban area) from 1985 to 2017. We show that, contrary to the dominant paradigm, more than 55% of the global rise in mean BMI from 1985 to 2017—and more than 80% in some low- and middle-income regions—was due to increases in BMI in rural areas. This large contribution stems from the fact that, with the exception of women in sub-Saharan Africa, BMI is increasing at the same rate or faster in rural areas than in cities in low- and middle-income regions. These trends have in turn resulted in a closing—and in some countries reversal—of the gap in BMI between urban and rural areas in low- and middle-income countries, especially for women. In high-income and industrialized countries, we noted a persistently higher rural BMI, especially for women. There is an urgent need for an integrated approach to rural nutrition that enhances financial and physical access to healthy foods, to avoid replacing the rural undernutrition disadvantage in poor countries with a more general malnutrition disadvantage that entails excessive consumption of low-quality calories.

## Abstract (web summary)

Contrary to the view that urbanization is a major driver of the global rise in obesity, the global increase in body-mass index is shown to be mostly due to increases in the body-mass indexes of rural populations.

## Main

Being underweight or overweight can lead to adverse health outcomes. BMI—a measure of underweight and overweight—is rising in most countries^2^. It is commonly stated that urbanization is one of the most important drivers of the worldwide rise in BMI because diet and lifestyle in cities lead to adiposity^3–6^. However, such statements are typically based on cross-sectional comparisons in one or a small number of countries. Only a few studies have analysed how BMI is changing over time in rural and urban areas. The majority have been in one country, over short durations, and/or in one sex and narrow age groups. The few studies that covered more than one country^7–12^ used at most a few dozen data sources and hence could not systematically estimate trends, and focused primarily on women of child-bearing age.

**Extended Data Fig. 1:**
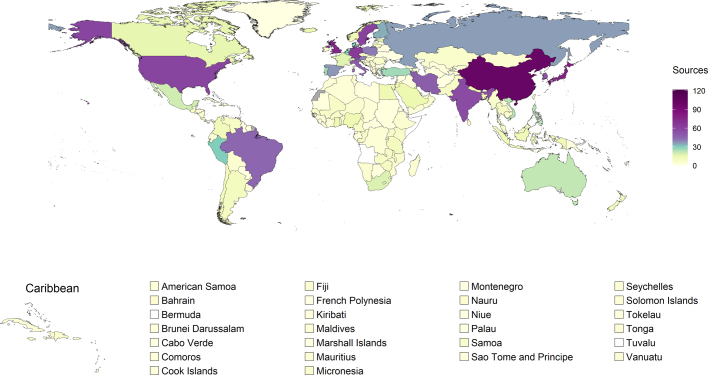
Number of data sources by country. The colour indicates the number of population-based data sources used in the analysis for each country. Countries and territories not included in the analysis are coloured in grey.

Data on how BMI in rural and urban populations is changing are needed to plan interventions that address underweight and overweight. Here, we report on mean BMI in rural and urban areas of 200 countries and territories from 1985 to 2017. We used 2,009 population-based studies of human anthropometry conducted in 190 countries (Extended Data Fig. 1), with measurements of height and weight in more than 112 million adults aged 18 years and older. We excluded data based on self-reported height and weight because they are subject to bias. For each sex, we used a Bayesian hierarchical model to estimate mean BMI by year, country and rural or urban place of residence. As described in the Methods, the estimated trends in population mean BMI represent a combination of (1) the change in the health of individuals due to change in their economic status and environment, and (2) the change in the composition of individuals that make up the population (and their economic status and environment).

**Extended Data Fig. 2:**
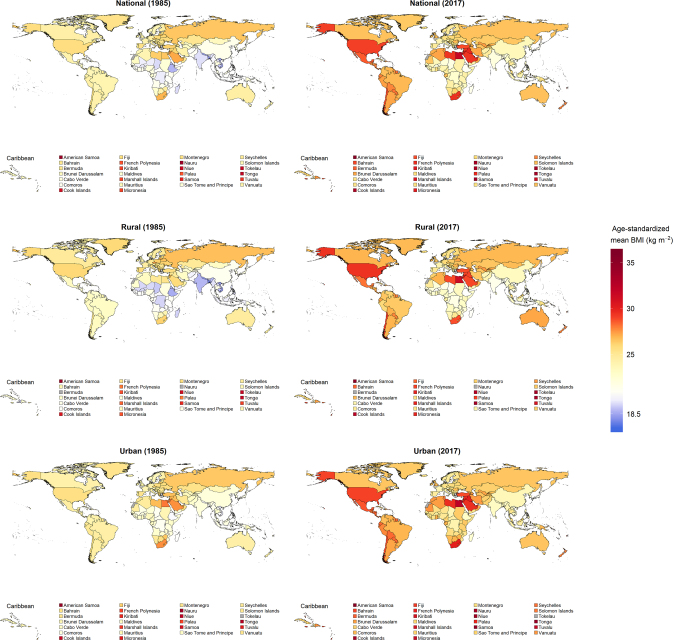
Age-standardized national, rural and urban mean BMI in women aged 18 years and older in 1985 and 2017 by country. The numerical values are provided in Supplementary Table 3 and can be downloaded from http://www.ncdrisc.org.

**Extended Data Fig. 3:**
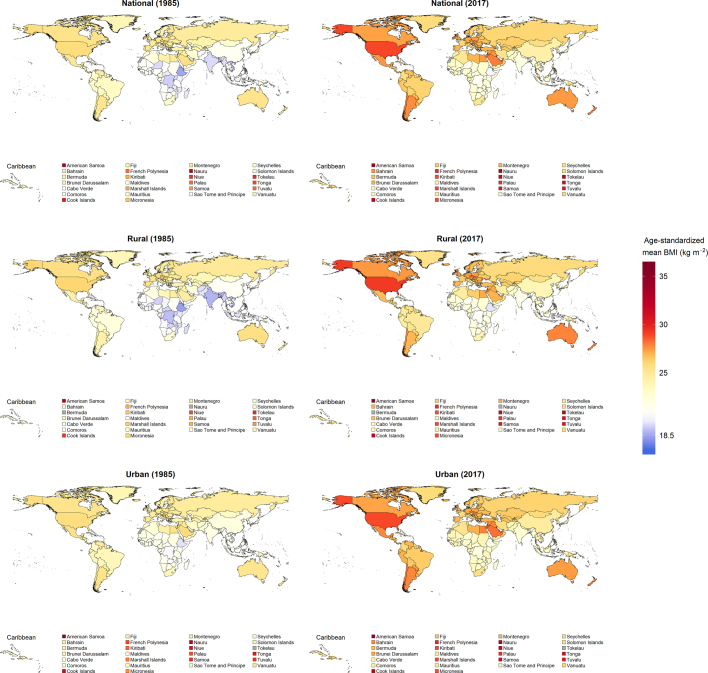
Age-standardized national, rural and urban mean BMI in men aged 18 years and older in 1985 and 2017 by country. The numerical values are provided in Supplementary Table 3 and can be downloaded from http://www.ncdrisc.org.

From 1985 to 2017, the proportion of the world’s population who lived in urban areas^1^ increased from 41% to 55%. Over the same period, global age-standardized mean BMI increased from 22.6 kg m^−2^ (95% credible interval 22.4–22.9) to 24.7 kg m^−2^ (24.5–24.9) in women, and from 22.2 kg m^−2^ (22.0–22.4) to 24.4 kg m^−2^ (24.2–24.5) in men. The increase in mean BMI was 2.09 kg m^−2^ (1.73–2.44) and 2.10 kg m^−2^ (1.79–2.41) among rural women and men, respectively, compared to 1.35 kg m^−2^ (1.05–1.65) and 1.59 kg m^−2^ (1.33–1.84) in urban women and men. Nationally, change in mean BMI ranged from small decreases among women in 12 countries in Europe and Asia Pacific, to a rise of >5 kg m^−2^ among women in Egypt and Honduras. The lowest observed sex-specific mean BMI over these 33 years was that of rural women in Bangladesh of 17.7 kg m^−2^ (16.3–19.2) and rural men in Ethiopia of 18.4 kg m^−2^ (17.0–19.9), both in 1985; the highest were 35.4 kg m^−2^ (33.7–37.1) for urban women and 34.6 kg m^−2^ (33.1–35.9) for rural men in American Samoa in 2017 (Extended Data Figs. 2, 3), representing a twofold difference.

**Fig. 1:**
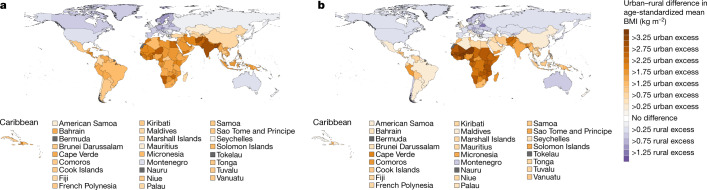
The difference between rural and urban age-standardized mean BMI in women. **a**, Difference in age-standardized mean BMI in 1985. **b**, Difference in age-standardized mean BMI in 2017. We did not estimate the difference between rural and urban areas for countries and territories in which the entire population live in areas classified as urban (Singapore, Hong Kong, Bermuda and Nauru) or rural (Tokelau)—shown in grey. See Extended Data Fig. 2 for mean BMI at the national level and in rural and urban populations in 1985 and 2017. See Extended Data Fig. 6 for comparisons of the results between women and men.

**Fig. 2:**
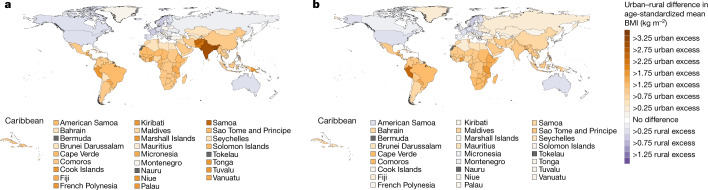
The difference between rural and urban age-standardized mean BMI in men. **a**, Difference in age-standardized mean BMI in 1985. **b**, Difference in age-standardized mean BMI in 2017. We did not estimate the difference between rural and urban areas for countries and territories in which the entire population live in areas classified as urban (Singapore, Hong Kong, Bermuda and Nauru) or rural (Tokelau)—shown in grey. See Extended Data Fig. 3 for mean BMI at the national level and in rural and urban populations in 1985 and 2017. See Extended Data Fig. 6 for comparison of results between women and men.

**Fig. 3:**
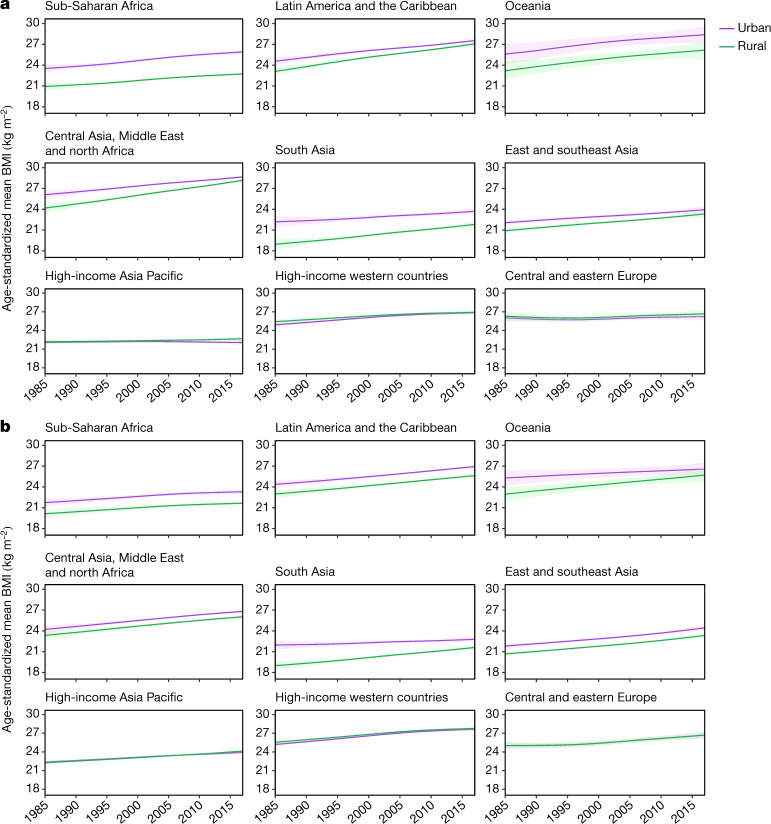
Trends in age-standardized mean BMI by rural and urban place of residence. **a**, Trends are shown for women in each region. **b**, Trends are shown for men in each region. The lines show the posterior mean estimates and the shaded areas show the 95% credible intervals.

**Extended Data Fig. 4:**
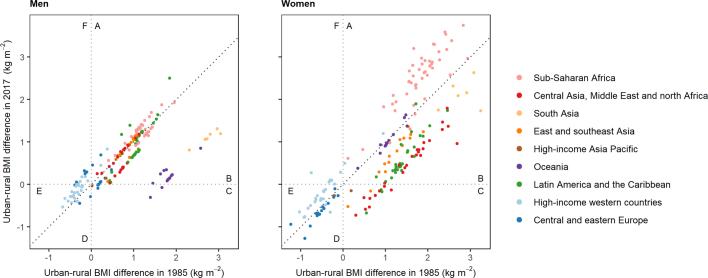
The difference between rural and urban age-standardized mean BMI in 1985 compared to 2017. Each point shows one country and colours indicate region. A positive number indicates a higher urban mean BMI and a negative number indicates a higher rural mean BMI. Different sections labelled A–F indicate the following categories of countries. A, countries with an urban excess BMI that increased from 1985 to 2017. B, countries with an urban excess BMI that decreased from 1985 to 2017. C, countries with an urban excess BMI in 1985 that changed to a rural excess BMI in 2017. D, countries with a rural excess BMI that increased from 1985 to 2017. E, countries with a rural excess BMI that decreased from 1985 to 2017. F, countries with a rural excess BMI in 1985 that changed to an urban excess BMI in 2017.

In 1985, urban men and women in every country in east, south and southeast Asia, Oceania, Latin America and the Caribbean and a region that comprises central Asia, the Middle East and north Africa had a higher mean BMI than their rural peers (Figs. 1, 2). The urban–rural gap was as large as 3.25 kg m^−2^ (2.57–3.96) in women and 3.05 kg m^−2^ (2.44–3.68) in men in India. Over time, the BMI gap between rural and urban women shrank in all of these regions by at least 40%, as BMI rose faster in rural areas than in cities (Fig. 3). In 14 countries in these regions, including Armenia, Chile, Jamaica, Jordan, Malaysia, Taiwan and Turkey, the ordering of rural and urban female BMI reversed over time and rural women had higher BMI than their urban peers in 2017 (Fig. 1 and Extended Data Fig. 4).

The mean BMI of rural men also increased more than the mean BMI of urban men in south Asia and Oceania, shrinking the urban–rural BMI gap by more than half (Figs. 2, 3). In east and southeast Asia, Latin America and the Caribbean, and central Asia, the Middle East and north Africa, men in both rural and urban areas experienced a similar BMI increase and, therefore, the urban excess BMI did not change substantially over time.

In contrast to emerging economies, excess BMI among urban women became larger in sub-Saharan Africa (Fig. 3): from 2.59 kg m^−2^ (2.21–2.98) in 1985 to 3.17 kg m^−2^ (2.93–3.42) in 2017 (posterior probability of the observed increase being a true increase >0.999). This occurred because female BMI rose faster in cities than in rural areas in sub-Saharan Africa. This led to women in sub-Saharan African countries, especially those in west Africa, having the largest urban excess BMI of any country in 2017—for example, more than 3.35 kg m^−2^ in Niger, Burkina Faso, Togo and Ghana (Fig. 1 and Extended Data Fig. 4). BMI increased at a similar rate in rural and urban men in sub-Saharan Africa, with the difference in 2017 (1.66 kg m^−2^; 1.37–1.94) being similar to 1985 (1.60 kg m^−2^; 1.13–2.07) (Fig. 2 and Extended Data Fig. 4).

BMI was previously lower in rural areas of low- and middle-income countries than in cities, both because rural residents had higher energy expenditure in their daily work—especially agriculture—and domestic activities, such as fuelwood and water collection^13,14^, and because lower incomes in rural areas restricted food consumption^15^. In middle-income countries, agriculture is increasingly mechanized, cars are used for rural transport as income increases and road infrastructure improves, service and administrative jobs have become more common in rural areas, and some household tasks are no longer needed—for example, because homes have a water connection and use commercial fuels^16^. Furthermore, higher incomes as a result of economic growth allow more spending on food and hence higher caloric intake, disproportionately more in rural areas, where a substantial share of income was previously spent on food. Additionally, the consumption of processed carbohydrates may have increased disproportionately in rural areas where such foods have become more readily available through national and transnational companies^9,17–21^. These changes, referred to as ‘urbanization of rural life’ by some researchers^6^, have contributed to a larger increase in rural BMI^22,23^.

In contrast to other regions, urbanization in sub-Saharan Africa preceded significant economic growth^24^. Subsistence farming remains common in Africa, and agriculture remains mostly manual; fuelwood—usually collected by women—is still the dominant fuel in rural Africa; and the use of cars for transportation is limited by poor infrastructure and poverty. In African cities, many people have service and office jobs, and mobility has become less energy-intensive owing to shorter travel distances and the use of cars and buses. Furthermore, urban markets where fresh produce is sold are increasingly replaced by commercially prepared and processed foods from transnational and local industries and street vendors^25–27^. These effects are exacerbated by limited time and space for cooking healthy meals and possibly perceptions of large weight as a sign of affluence^28,29^.

In contrast to low- and middle-income regions, urban women in high-income western and Asia Pacific regions, and in central and eastern Europe, had slightly lower mean BMI than their rural peers in 2017 (Fig. 3). The rural excess BMI for women in these regions changed little from 1985 to 2017. Nationally, the excess BMI of rural women was largest in central and eastern European countries (for example, around 1 kg m^−2^ or more in Belarus, Latvia and Czech Republic; Fig. 1 and Extended Data Fig. 4). Rural men in high-income western countries also had an excess BMI compared to urban men throughout the analysis period. The largest rural excess BMI for men in 2017 was seen in Sweden, Czech Republic, Ireland, Australia, Austria and the United States, which all had an excess BMI of 0.35 kg m^−2^ or larger. In the high-income Asia Pacific region and in central and eastern Europe, rural and urban men had almost identical BMI throughout these three decades (Fig. 2 and Extended Data Fig. 4).

The lower urban BMI in high-income and industrialized countries reflects a growing rural economic and social disadvantage, including lower education and income, lower availability and higher price of healthy and fresh foods^30,31^, less access to, and use of, public transport and walking than in cities^32,33^, and limited availability of facilities for sports and recreational activity^34^, which account for a significant share of overall physical activity in high-income and industrialized countries.

We also estimated how much of the overall rise in mean BMI since 1985 has been due to increases in BMI of rural and urban populations versus those attributable to urbanization (defined as an increase in the proportion of the population who live in urban areas), in each region and in the world as a whole. At the global level, 60% (56–64) of the rise in mean BMI from 1985 to 2017 in women and 57% (53–60) in men was due to increases in the BMI of rural populations; 28% (24–31) in women and 30% (27–32) in men due to the rise in BMI in urban populations; and 13% (11–15) and 14% (12–16) due to urbanization (Table 1). The contribution of the rise in rural BMI ranged from around 60% to 90% in the mostly rural regions of sub-Saharan Africa, east, south and southeast Asia and Oceania. The contribution of urbanization was small in all regions of the world, with maximum values of 19% (15–25) among women and 14% (10–21) among men in sub-Saharan Africa.

**Table 1:** Contributors to the rise in mean BMI from 1985 to 2017

			Rural component		Urban component		Urbanization component	
			Absolute contribution (kg m^−2^)	Percentage contribution (%)	Absolute contribution (kg m^−2^)	Percentage contribution (%)	Absolute contribution (kg m^−2^)	Percentage contribution (%)
**Emerging economies**								
	Central Asia, Middle East and north Africa	Men	1.30 (0.96–1.64)	48 (41–54)	1.33 (1.02–1.65)	49 (44–54)	0.09 (0.06–0.12)	3 (2–5)
		Women	1.96 (1.57–2.33)	59 (54–64)	1.31 (0.95–1.69)	39 (34–44)	0.06 (0.03–0.09)	2 (1–3)
	East and southeast Asia	Men	1.99 (1.62–2.37)	67 (63–71)	0.66 (0.53–0.80)	22 (20–24)	0.33 (0.26–0.39)	11 (9–14)
		Women	1.81 (1.36–2.26)	73 (67–80)	0.47 (0.32–0.64)	19 (16–22)	0.18 (0.10–0.26)	7 (4–11)
	Latin America and the Caribbean	Men	0.86 (0.63–1.09)	31 (26–37)	1.73 (1.31–2.16)	63 (58–67)	0.17 (0.13–0.20)	6 (5–8)
		Women	1.29 (1.07–1.51)	38 (34–43)	2.01 (1.56–2.49)	60 (55–63)	0.06 (0.03–0.10)	2 (1–3)
	Oceania	Men	2.24 (1.12–3.37)	90 (80–102)	0.24 (−0.03–0.51)	10 (−2–20)	0.00 (0.00–0.00)	0 (0–0)
		Women	2.41 (0.89–3.98)	81 (69–90)	0.53 (0.18–0.89)	19 (10–31)	0.00 (0.00–0.00)	0 (0–0)
	South Asia	Men	1.99 (1.42–2.54)	86 (79–94)	0.20 (0.00–0.40)	8 (0–15)	0.12 (0.09–0.15)	5 (3–8)
		Women	2.18 (1.46–2.87)	80 (73–87)	0.36 (0.13–0.60)	13 (6–19)	0.19 (0.16–0.23)	7 (5–11)
**Sub-Saharan Africa**								
	Sub-Saharan Africa	Men	1.14 (0.64–1.63)	64 (53–73)	0.39 (0.22–0.55)	22 (15–28)	0.23 (0.19–0.27)	14 (10–21)
		Women	1.37 (0.90–1.83)	57 (49–63)	0.58 (0.42–0.74)	24 (21–28)	0.45 (0.42–0.49)	19 (15–25)
**High-income and other industrialized regions**								
	Central and eastern Europe	Men	0.59 (0.35–0.82)	35 (26–44)	1.10 (0.70–1.50)	65 (57–73)	0.00 (−0.01–0.01)	0 (−1–1)
		Women	0.14 (−0.19–0.45)	NR	0.13 (−0.45–0.69)	NR	−0.02 (−0.03–0.00)	NR
	High-income Asia Pacific	Men	0.48 (0.37–0.59)	31 (25–37)	1.15 (0.84–1.46)	72 (68–75)	−0.04 (−0.08–0.00)	−2 (−6–0)
		Women	0.12 (−0.01–0.27)	NR	−0.02 (−0.38–0.36)	NR	−0.10 (−0.15 to −0.06)	NR
	High-income western countries	Men	0.58 (0.47–0.69)	24 (22–27)	1.80 (1.53–2.07)	76 (74–78)	−0.01 (−0.02–0.00)	0 (−1–0)
		Women	0.39 (0.24–0.54)	21 (15–26)	1.44 (1.09–1.79)	79 (74–84)	0.00 (−0.02–0.01)	0 (−1–1)
**World**								
	World	Men	1.24 (1.06–1.43)	57 (53–60)	0.65 (0.54–0.75)	30 (27–32)	0.30 (0.28–0.32)	14 (12–16)
		Women	1.22 (1.01–1.43)	60 (56–64)	0.56 (0.44–0.69)	28 (24–31)	0.25 (0.23–0.27)	13 (11–15)

Contributions of the rise in mean BMI in rural and urban populations and of urbanization to the rise in mean BMI from 1985 to 2017, by region. Urbanization is defined as an increase in the proportion of the population who live in urban areas. Percentage contributions were calculated as described in the Methods. The reported values are the means and 95% credible intervals. The three percentages sum to 100%. When one component causes an increase in BMI in a region and another does the opposite, the components can be negative or greater than 100%. Urban and rural mean BMI and the percentage of the population who live in urban areas in 1985 and 2017 for each region are provided in Extended Data Table 1. NR, percentage contribution was not reported, because the regional change in mean BMI (which appears in the denominator of the percentage contribution) was small (<0.5 kg m^−2^), leading to unstable estimates.

Our results show that, contrary to the prevailing view^3–6^, BMI is rising at the same rate or faster in rural areas compared to cities, particularly in low- and middle-income countries except among women in sub-Saharan Africa. These trends have resulted in a rural–urban convergence in BMI in most low- and middle-income countries, especially for women. This convergence mirrors the experience of high-income and industrialized countries, where we found a persistently higher BMI in rural areas. The rising rural BMI is the largest contributor to the BMI rise in low- and middle-income regions and in the world as a whole over the last 33 years, which challenges the current paradigm of urban living and urbanization as the key driver of the global epidemic of obesity.

In poor societies, urban areas historically had lower levels of undernutrition^35,36^, possibly because infrastructure such as roads and electricity facilitate food trade, transport and storage in cities, which can in turn reduce the impacts of agricultural shocks and seasonality. As economic growth and rural nutrition programmes reduce rural caloric deficiency, the rural undernutrition disadvantage may be replaced with a more general and complex malnutrition that entails excessive consumption of low-quality calories. To avoid such an unhealthy transition, the fragmented national and international responses to undernutrition and obesity should be integrated, and the narrow focus of international aid on undernutrition should be broadened, to enhance access to healthier foods in poor rural and urban communities.

### Reporting summary

Further information on research design is available in the Nature Research Reporting Summary linked to this paper.

## Methods

Our aim was to estimate trends in mean BMI from 1985 to 2017 by rural and urban place of residence for 200 countries and territories (Supplementary Table 2). To achieve this aim, we pooled cross-sectional population-based data on height and weight in adults aged 18 years and older. Therefore, by design, our results measure total change in BMI in each country’s rural and urban populations, which consists of (1) change in the BMI of individuals due to change in their economic status and environment, and (2) change in the composition of individuals that make up the population (and their economic status and environment). Change in population composition occurs naturally owing to fertility and mortality, as well as owing to migration. Therefore, our results should not be interpreted as solely a change in the BMI of individuals. Both components of change are relevant for policy formulation because policies should address the environment and nutrition of the contemporary population.

**Extended Data Fig. 5:**
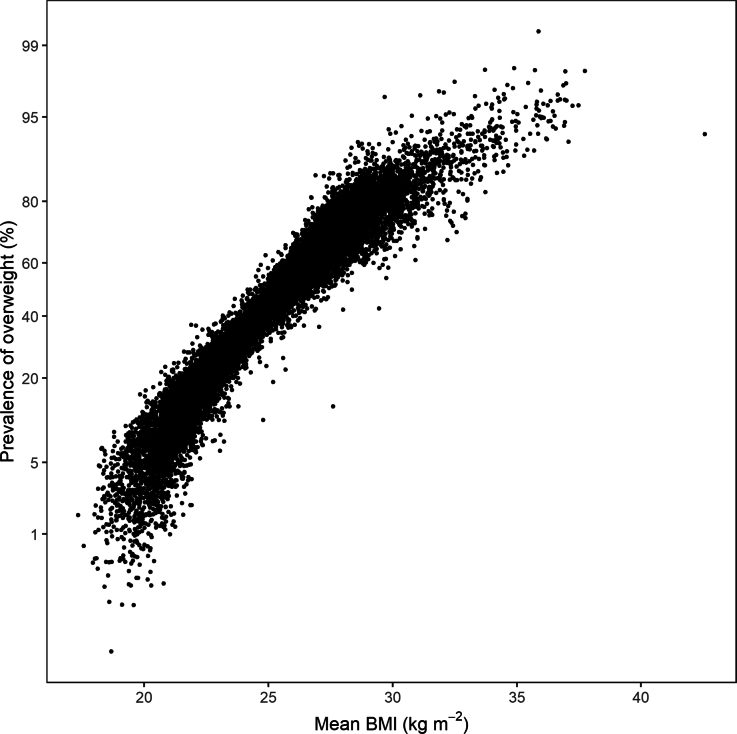
The relationship between mean BMI and prevalence of overweight. Overweight is defined as BMI ≥ 25 kg m^−2^. Prevalence is plotted on a probit scale, which changes in an approximately linear manner as the mean changes. Each point represents an age group- and sex-specific mean, stratified by place of residence as described in the Methods and with more than 25 participants, from data sources in the NCD-RisC database.

We used mean BMI as the primary outcome, rather than prevalence of overweight or obesity, because the relationship between BMI and disease risk is continuous, with each unit lower BMI being associated with a constant proportional reduction in disease risk until a BMI of around 21–23 kg m^−2^, which is below the cut-offs used to define overweight and obesity^37–39^. Therefore, the largest health benefits of weight management are achieved by lowering the population distribution of BMI. Mean BMI is the simplest summary statistic of the population distribution. Nonetheless, mean BMI and prevalence of overweight and obesity are closely associated (Extended Data Fig. 5).

### Data sources

We used a database on cardiometabolic risk factors collated by the Non-Communicable Disease Risk Factor Collaboration (NCD-RisC). NCD-RisC is a worldwide network of health researchers and practitioners, that systematically documents the worldwide trends and variations in risk factors for non-communicable diseases. The database was collated through multiple routes for identifying and accessing data. We accessed publicly available population-based measurement surveys—for example, Demographic and Health Surveys, Global School-based Student Health Surveys, the European Health Interview and Health Examination Surveys and those available via the Inter-University Consortium for Political and Social Research. We requested, through the World Health Organization (WHO) and its regional and country offices, help with identification and access to population-based surveys from ministries of health and other national health and statistical agencies. Requests were also sent by the World Heart Federation to its national partners. We made similar requests to the co-authors of an earlier pooled analysis of cardiometabolic risk factors^40–43^ and invited them to reanalyse data from their studies and join NCD-RisC. Finally, to identify major sources not accessed through the above routes, we searched and reviewed published studies as described previously^44^ and invited all eligible studies to join NCD-RisC.

Anonymized individual record data from sources included in NCD-RisC were reanalysed according to a common protocol. Within each survey, we included participants aged 18 years and older who were not pregnant. We dropped participants with implausible BMI levels (defined as BMI < 10 kg m^−2^ or BMI > 80 kg m^−2^) or with implausible height or weight values (defined as height < 100 cm, height > 250 cm, weight < 12 kg or weight > 300 kg; <0.2% of all subjects). We also dropped participants whose urban and rural status was unknown in surveys that had recorded place of residence (0.05% of all participants). We calculated mean BMI and its standard error by sex, age group (18 years, 19 years, 10-year age groups from 20–29 years to 70–79 years and 80+ years) and rural or urban place of residence. All analyses incorporated appropriate sample weights and complex survey design, when applicable, in calculating summary statistics. Countries typically use the rural and urban classification of communities designated by their statistical offices at any given time both for survey design and for reporting of population to the United Nations Population Division. The classification can change, for example as previously rural areas grow and industrialize and hence become, and are (re)designated as, de novo cities. To the extent that the reclassifications keep up with changes in the real status of each community, survey and population data reflect the status of each community at the time of measurement. For surveys without information on place of residence, we calculated age- and sex-stratified summary statistics for the entire sample, which represented the population-weighted sum of rural and urban means.

To ensure summaries were prepared according to the study protocol, computer code was provided to NCD-RisC members who requested assistance. All submitted data were checked by at least two independent reviewers. Questions and clarifications were discussed with NCD-RisC members and resolved before data were incorporated into the database.

Finally, we incorporated all nationally representative data from sources that were identified but not accessed through the above routes, by extracting summary statistics from published reports. Data were also extracted for nine WHO STEPwise approach to Surveillance (STEPS) surveys, one Countrywide Integrated Non-communicable Diseases Intervention (CINDI) survey, and five sites of the WHO Multinational MONItoring of trends and determinants in CArdiovascular disease (MONICA) project that were not deposited in the MONICA Data Centre. Data were extracted from published reports only when reported by sex and in age groups no wider than 20 years. We also used data from a previous global data pooling study^43^ when such data had not been accessed through the routes described.

All NCD-RisC members are asked periodically to review the list of sources from their country, to suggest additional sources not in the database, and to verify that the included data meet the inclusion criteria listed below and are not duplicates. The NCD-RisC database is continuously updated through this contact with NCD-RisC members. For this paper, we used data from the NCD-RisC database for years 1985 to 2017 and ages 18 years and older. A list of the data sources that we used in this analysis and their characteristics is provided in Supplementary Table 1.

### Data inclusion and exclusion

Data sources were included in the NCD-RisC database if: (1) measured data on height, weight, waist circumference or hip circumference were available; (2) study participants were 5 years of age and older; (3) data were collected using a probabilistic sampling method with a defined sampling frame; (4) data were from population samples at the national, sub-national (that is, covering one or more sub-national regions, more than three urban communities or more than five rural communities) or community level; and (5) data were from the countries and territories listed in Supplementary Table 2.

We excluded all data sources that were based solely on self-reported weight and height without a measurement component, because these data are subject to biases that vary by geography, time, age, sex and socioeconomic characteristics^45–47^. Owing to these variations, approaches to correcting self-reported data leave residual bias. We also excluded data sources on population subgroups whose anthropometric status may differ systematically from the general population, including: (1) studies that included or excluded people based on their health status or cardiovascular risk; (2) studies whose participants were only ethnic minorities; (3) specific educational, occupational, or socioeconomic subgroups, with the exception noted below; (4) those recruited through health facilities, with the exception noted below; and (5) women aged 15–19 years in surveys which sampled only ever-married women or measured height and weight only among mothers.

We used school-based data in countries, and in age–sex groups, with school enrolment of 70% or higher. We used data for which the sampling frame was health insurance schemes in countries in which at least 80% of the population were insured. Finally, we used data collected through general practice and primary care systems in high-income and central European countries with universal insurance, because contact with the primary care systems tends to be as good as or better than response rates for population-based surveys.

### Conversion of BMI prevalence metrics to mean BMI

In 2% of our data points—mostly extracted from published reports or from a previous pooling analysis^43^—mean BMI was not reported, but data were available for the prevalence of one or more BMI categories, for example, BMI ≥ 30 kg m^−2^. In order to use these data, we used previously validated conversion regressions^2^ to estimate the missing primary outcome from the available BMI prevalence metric(s). All sources of uncertainty in the conversion—including the sampling uncertainty of the original data, the uncertainty of the regression coefficients and random effects, and the regression residuals—were carried forward by using repeated draws from their joint posterior distribution, accounting for the correlations among the uncertainties of regression coefficients and random effects.

### Statistical analysis of BMI trends by rural and urban place of residence

We used a Bayesian hierarchical model to estimate mean BMI by country, year, sex, age and place of residence. The statistical model is described in detail in a statistical paper and related substantive papers^2,35,40–44,48–51^, and in the Supplementary Information. In summary, we organized countries into 21 regions (Supplementary Table 2), mostly based on geography and national income. The exception was high-income English-speaking countries (Australia, Canada, Ireland, New Zealand, the United Kingdom and the United States), grouped together in one region because BMI and other cardiometabolic risk factors have similar trends in these countries, which can be distinct from other countries in their geographical regions^2,49,50,52^. Regions were in turn organized into nine super-regions.

The model had a hierarchical structure in which estimates for each country and year were informed by their own data, if available, and by data from other years in the same country and from other countries, especially those in the same region with data for similar time periods. The extent to which estimates for each country-year were influenced by data from other years and other countries depended on whether the country had data, the sample size of the data, whether they were national, and the within-country and within-region variability of the available data. The model incorporated nonlinear time trends comprising linear terms and a second-order random walk, all modelled hierarchically. The age association of BMI was modelled using a cubic spline to allow nonlinear age patterns, which could vary across countries. The model accounted for the possibility that BMI in sub-national and community samples might differ systematically from nationally representative ones and have larger variation than in national studies. These features were implemented by including data-driven fixed-effect and random-effect terms for sub-national and community data. The fixed effects adjusted for systematic differences between sub-national or community studies and national studies. The random effects allowed national data to have larger influence on the estimates than sub-national or community data with similar sample sizes.

Here, we extended the model to make estimates for rural and urban populations following a previously published approach^35,51^. This model includes a parameter representing the urban–rural BMI difference, which is estimated empirically and allowed to vary by country and year. The model uses all of the data—those stratified by rural and urban place of residence as well as those reported for the entire population. If data for a country-year were not stratified by place of residence, the estimated urban–rural BMI difference was informed by stratified data from other years and countries, especially those in the same region with data from similar time periods.

We fitted the statistical model with the Markov chain Monte Carlo (MCMC) algorithm and following burn-in obtained 5,000 samples (or draws) from the posterior distribution of model parameters, which were in turn used to obtain the posterior distributions of our primary outcomes—mean urban BMI, mean rural BMI and mean urban–rural BMI difference. Posterior estimates were made in 1-year age groups for ages 18 and 19 and 5-year age groups for those aged 20 years and older. We generated age-standardized estimates by taking weighted means of age-specific estimates, using age weights from the WHO standard population. Regional and global rural and urban mean BMI estimates were calculated as population-weighted averages of rural and urban mean for the constituent country estimates by age group and sex. National mean BMI was calculated as population-weighted averages of the rural and urban means. All analyses were done separately by sex because geographical and temporal patterns of BMI differ between men and women^2^.

The reported credible intervals represent the 2.5th and the 97.5th percentiles of the posterior distributions. We report the posterior probability that the estimated urban–rural BMI difference is a true difference in the same direction as the posterior mean estimate. We also report the posterior probability that the estimated change in the rural–urban BMI difference over time represents a true increase or decrease.

### Validation of statistical model

We calculated the difference between the posterior estimates from the model and data from national studies. Median errors were very close to zero (0.03 kg m^−2^ for women and −0.02 kg m^−2^ for men) and median absolute errors were 0.32 kg m^−2^ for women and 0.26 kg m^−2^ for men, indicating that the estimates were unbiased and had small deviations relative to national studies. The differences were indistinguishable from zero at the 5% level of statistical significance.

We also tested how well our statistical model predicts missing data, known as external predictive validity or cross-validation, in two different tests. In the first test, we held out all data from 10% of countries with data (that is, created the appearance of countries with no data for which we actually had data). The countries for which the data were withheld were selected randomly from the following three groups: data rich (8 or more data sources for women and 7 or more data sources for men), data poor (1–3 data sources for women and 1–2 for men) and average data availability (4–7 data sources for women and 3–6 for men). All data-rich countries had at least one data source after 2000 and at least one source with data stratified on rural and urban place of residence. We fitted the model to the data from the remaining 90% of countries and made estimates of the held-out observations. In the second test, we assessed other patterns of missing data by holding out 10% of our data sources, again from a mix of data-rich, data-poor and average-data countries, as defined above. For a given country, we either held out a random one third of the country’s data or all of the country’s 2000–2017 data to determine, respectively, how well we filled in the gaps for countries with intermittent data and how well we estimated in countries without recent data. We fitted the model to the remaining 90% of the dataset and made estimates of the held-out observations. We repeated each test five times, holding out a different subset of data in each repetition. In both tests, we calculated the differences between the held-out data and the estimates. We also calculated the 95% credible intervals of the estimates; in a model with good external predictive validity, 95% of held-out values would be included in the 95% credible intervals.

Our statistical model performed very well in the external validation tests, that is, in estimating mean BMI when data were missing. The estimates of mean BMI were unbiased, as evidenced with median errors that were zero or close to zero globally (0.03 and −0.03 kg m^−2^ for women and –0.15 and 0.00 kg m^−2^ for men in the first and second tests, respectively), and less than ±0.20 kg m^−2^ in every subset of withheld data except 1985–1999 data in the first test for men, for which the median error was −0.24 kg m^−2^ (Extended Data Table 2). Most of the median errors were indistinguishable from zero at the 5% level of statistical significance. The 95% credible intervals of estimated mean BMI covered 94–98% of true data globally; coverage was >93% in all but one subset of withheld data. Median absolute errors ranged from 0.52 to 1.09 kg m^−2^ globally and were at most 1.29 kg m^−2^ in all subsets of withheld data. Median absolute errors were smaller in the second test, in which subsets of data sources from some countries were withheld, than in the first test, in which all data from some countries were withheld. Given that we had data for 190 out of 200 countries for women and 183 out of 200 countries for men, the second test is a better reflection of data availability in our analysis. For comparison, median absolute differences for mean BMI between pairs of nationally representative surveys done in the same country and in the same year was 0.46 kg m^−2^, indicating that our estimates perform almost as well as running two parallel surveys in the same country and year.

### Contributions of urbanization and rural and urban BMI change to changes in population mean BMI

We calculated the contributions of the following components to change in population mean BMI from 1985 to 2017: the contribution of change in BMI in rural areas, the contribution of change in BMI in urban areas, and the contribution of urbanization (that is, increase in the proportion of people living in urban areas). The first two parts were calculated by fixing the proportion of people living in rural and urban areas to 1985 levels and allowing BMI to change as it did in the respective population. The contribution of urbanization was calculated by fixing BMI in rural and urban areas to 2017 levels and allowing the proportion of people living in cities to change as it did. Percentage contributions were calculated using posterior draws, with reported credible intervals representing the 2.5th and the 97.5th percentiles of their posterior distributions. The change in mean BMI from 1985 to 2017 was then calculated as (contribution of change in rural BMI + contribution of change in urban BMI + contribution of change in the proportion of the population living in urban areas) = ((change in BMI_rural1985–2017_)(percentage living in rural areas_1985_) + (change in BMI_urban1985–2017_)(percentage living in urban areas_1985_) +(change in percentage living in urban areas_1985–2017_)(BMI_urban2017_ − BMI_rural2017_)).

### Strengths and limitations

Urbanization is regarded as one of the most important contributors to the global obesity epidemic, but this perspective is based on limited data. We present the first comparable estimates of mean BMI for rural and urban populations worldwide over three decades using, to our knowledge, the largest and most comprehensive global database of human anthropometry with information on urban or rural place of residence. We used population-based measurement data from almost all countries, with information on participants’ urban or rural place of residence for the majority of data sources. We maintained a high level of data quality through repeated checks of study characteristics against our inclusion and exclusion criteria, which were verified by NCD-RisC members, and did not use any self-reported data to avoid bias in height and weight. Data were analysed according to a common protocol to obtain mean BMI by age, sex and place of residence. We used a statistical model that used all available data, while giving more weight to national data than sub-national and community studies and took into account the epidemiological features of BMI by using nonlinear time trends and age associations. The model used information on the urban–rural difference in BMI where available and estimated this difference hierarchically and temporally in the absence of stratified data.

Despite our large-scale data collation effort, some countries and regions had fewer data sources, particularly the Caribbean, and Polynesia and Micronesia. There were also fewer data sources before 2000. This temporal and geographical sparsity of data led to wider uncertainty intervals for these countries, regions and years. Although health surveys commonly use the rural and urban classification of national statistical offices, cities and rural areas in different countries vary in their demographic characteristics (for example, population size or density), economic activity, administrative structures, infrastructure and environment. These differences appropriately exist because countries themselves differ in terms of their demography, geography and economy. For example, a country with a smaller population may use a lower threshold for urban designation than one with a larger population, because its cities are naturally smaller even if they serve the same functions. Official rural and urban classifications are used for resource allocation and planning for nutrition and health^53–58^, which makes them the appropriate unit for tracking outcomes. Nonetheless, understanding the causes of change in rural and urban areas can be enriched with use of more complex and multi-dimensional measures of urbanicity involving size, density, economic and commercial activities and infrastructures^59,60^. Finally, urbanization could arise from a variety of mechanisms: (1) natural increase due to excess births over deaths in cities compared to rural areas, (2) rural to urban migration (often related to opportunities for work and education) and (3) reclassification of previously rural areas as they grow and industrialize and hence become, and are (re)designated as, de novo cities. The contributions of these mechanisms to urbanization vary across countries. The use of time-varying rural versus urban classification of communities ensures that in any year, the rural and urban strata represent the actual status of each community. However, each of these mechanisms may have different implications for changes in nutrition and physical activity and, therefore, BMI.

## Online content

Any methods, additional references, Nature Research reporting summaries, source data, statements of data availability and associated accession codes are available at 10.1038/s41586-019-1171-x.

## Supplementary information

Supplementary Information This file contains the statistical model for estimating BMI trends by rural and urban place of residence, Supplementary Tables 1-3 and Supplementary References.

Reporting Summary

## Figures and Tables

**Extended Data Fig. 6:**
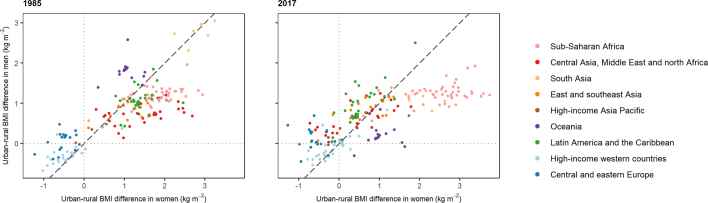
Comparison of the difference between rural and urban age-standardized mean BMI in women and men aged 18 years and older in 1985 and 2017. Each point shows one country and colours indicate region.

**Extended Data Table 1:**
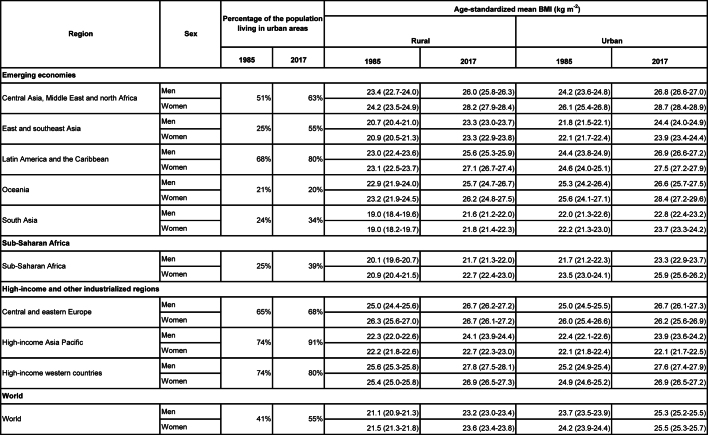
Mean BMI and percentage of the population by urban and rural place of residence

For each region, the table shows age-standardized mean BMI for urban and rural populations and the percentage of the population living in urban areas in 1985 and 2017.

See Supplementary Table 2 for a list of countries in each region. Numbers in parentheses show 95% credible intervals.

**Extended Data Table 2:**
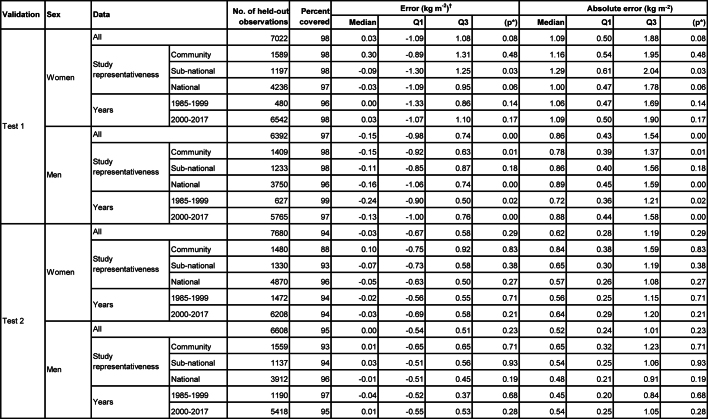
Results of model validation

Q1, first quartile; Q3, third quartile; p, p value.

†Estimated values minus held-out values.

*p values for model error comparisons were calculated using the non-parametric Wilcoxon signed-rank test for paired data. The p values are calculated assuming independence of the held-out observations. They should therefore be interpreted as an approximation because there is some dependence among the held-out observations, within each of the five repetitions for example.

## Data Availability

Estimates of mean BMI by country, year, sex and urban and rural place of residence are available from http://www.ncdrisc.org/. Input data from publicly available sources can also be downloaded from http://www.ncdrisc.org/. For other data sources, contact information for data providers can be obtained from http://www.ncdrisc.org/.

## Code availability

The computer code for the Bayesian hierarchical model used in this work is available at http://www.ncdrisc.org/.
