# Supplementary material for: Rising rural body-mass index is the main driver of the global obesity epidemic in adults
Source: Nature. 2019 May 8;569(7755):260–4. doi: 10.1038/s41586-019-1171-x (PMC6784868; doi:10.1038/s41586-019-1171-x)
Supplement: Supplementary file 1 — This file contains the statistical model for estimating BMI trends by rural and urban place of residence, Supplementary Tables 1-3 and Supplementary References. [file 41586_2019_1171_MOESM1_ESM.pdf]

https://doi.org/10.1038/s41586-019-1171-x

Rising rural body-mass index is the main driver of the global obesity epidemic in adults

NCD Risk Factor Collaboration (NCD-RisC)*

*A list of authors and their affiliations appears in the online version of the paper.

Supplementary Information. Statistical model for estimating BMI trends by rural and urban place of residence.

Statistical model

The Bayesian hierarchical model used for estimating mean body-mass index (BMI) trends by rural and urban place of residence was summarised in Methods. Here we provide a more detailed description of the model. Further details can be found in two statistical papers[1,2] and in the appendices of two empirical public health papers.[3,4]

1 Data

For each data source, we obtained data stratified by rural or urban place of residence, age and sex, including information on sample mean BMI, and whether the study sample was from the national population, from sub-national regions or from one or a small number of communities. For sources without information on place of residence, we obtained age-and-sex-stratified data.

Despite using the largest and most comprehensive database of human anthropometry, there were gaps in our data, because health and nutrition surveys are done at different frequencies across the world. For women, our analysis was based on 14,278 age-group-specific sample means (39% rural, 48% urban and 13% mixed) from 1,250 non-overlapping country-by-year combinations; for men, we had 12,958 sample means (37% rural, 47% urban and 16% mixed) from 1,157 non-overlapping country-by-year combinations. 10 countries (5.0%) had no data for women and 17 countries (8.5%) had no data for men. In addition, some data sources only had data in certain age groups.

60% of observations were from nationally representative studies, 17% from sub-national regions and 23% from studies in one or a few communities. Non-national data may have systematic differences from national data. In addition, BMI levels may vary across communities in the same country which increases the variance of data.

Our model was designed to use these disparate data sources to estimate mean BMI for each country × urban or rural place of residence × year × age combination. We fitted separate models for men and women because BMI levels, trends and urban-rural differences differ between them.[5,6]

2 Model

Each study contributed from 1 to 18 mean BMI values according to how many age groups were observed, and whether the study provided data stratified on urban and rural places of residence. The likelihood for an observation at urbanisation level s (urban-only, rural-only or mixed; referred to as stratum hereinafter) and age group h from study i, carried out in country j at time t is:

$$y_{s,h,i} \sim N\left(a_{j[i]} + b_{j[i]}t_i + u_{j[i],t_i} + \gamma_i(z_h) + \boldsymbol{X_i\beta} + e_i + I_{s,i}\left[p_{j[i]} + q_{j[i]}t_i + d_i\right], SD^2_{s,h,i}/n_{s,h,i} + \tau_i^2\right)$$

where the country-specific intercept and linear time slope from the jth country (j = 1 … J, where J = 200 which is the total number of countries in our analysis) are denoted a_j and b_j, respectively. We describe the hierarchical model used for the a's and b's in Section 2.1. Letting T = 33 be the total number of years from 1985 to 2017, the T-vector u_j captures smooth non-linear change over time in country j, as described in Section 2.2. The age effects of the hth age group (with mid-age z) in study i are denoted by γ_i; we describe the age model in Section 2.3. The matrix $\boldsymbol{X}$ contains terms describing whether studies were representative at the national, sub-national or community level. In addition, a random effect, e_i, is estimated for each study, described in Section 2.4.

The likelihood variance has two components. Sampling uncertainty in mean BMI is calculated for each study-stratum-age-group as $SD^2_{s,h,i}/n_{s,h,i}$, where $SD_{s,h,i}$ is the standard deviation of

BMI values observed in an age group and stratum of a particular study and $n_{s,h,i}$ is the corresponding sample size. Additional residual variability between age groups within a study is captured by the τ^2 terms, described in Section 2.5.

The difference in mean BMI between urban and rural residents is specified by time-varying country-level offset terms (p_j and q_j), as described in Section 2.6. We also include an additional study-specific random effect (d_i) for this difference, which accounts for how the urban-rural difference in each study differs from that of the country. I indicates whether the observation is from an urban, rural or mixed population, as also explained in Section 2.6.

2.1 Linear components of the country time trends

The model has a hierarchical structure: studies are nested in countries, which are nested in regions (indexed by k), which are nested in super-regions (indexed by l), which are, of course, all nested in the globe. This structure allows the model to share information across units to a greater degree when data are non-existent or weakly informative (e.g., have a small sample size or are not nationally representative), and to a lesser extent in data-rich countries and regions.[7]

The a and b terms are country-specific linear intercepts and time slopes with terms at each level of the hierarchy, denoted by the superscripts $c, r, s,$ and g, respectively:

$$a_j = a_j^c + a_{k[j]}^r + a_{l[k]}^s + a^g,$$

$$b_j = b_j^c + b_{k[j]}^r + b_{l[k]}^s + b^g,$$

$$a_j^x \sim N(0, \kappa_a^x),$$

$$b_j^x \sim N(0, \kappa_b^x) \text{ (where } x = \{c, r, s\})$$

The κ terms are each assigned a flat prior on the standard deviation scale.[8] We also assign flat priors to a^g and b^g.

2.2 Non-linear change

Mean BMI may change non-linearly over time.[5,6,9] We capture smooth non-linear change in time in urban and rural strata of country j using the vector u_j. Just as a_j and b_j are each defined as the sum of country, region, super-region, and global components, we define:

$$u_j = u_j^c + u_{k[j]}^r + u_{l[k]}^s + u^g$$

In order to allow the model to differentiate between the degrees of non-linearity that exist at the country, region, super-region, and global levels, we assign each of the u's four components a Gaussian autoregressive prior as in Breslow and Clayton[10] and Rue and Held.[11] In particular, the T-vectors u_j^c (j = 1 … J), u_k^r (k = 1 … K), u_l^s (l = 1 … L), and u^g each have a normal prior with mean zero and precision $\lambda_c P$, $\lambda_r P$, $\lambda_s P$, and $\lambda_g P$ respectively, where the scaled precision matrix P in the Gaussian autoregressive prior penalizes first and second differences:

$$P = \begin{bmatrix} 1 & 0 & 0 & \cdots & 0 \\ -2 & 1 & 0 & \cdots & 0 \\ 1 & -2 & 1 & \cdots & 0 \\ 0 & 1 & -2 & \cdots & 0 \\ 0 & 0 & 1 & \cdots & 0 \\ \vdots & \vdots & \vdots & \ddots & \vdots \\ 0 & 0 & 0 & \cdots & 1 \end{bmatrix} \begin{bmatrix} 1 & -2 & 1 & 0 & 0 & \cdots & 0 \\ 0 & 1 & -2 & 1 & 0 & \cdots & 0 \\ 0 & 0 & 1 & -2 & 1 & \cdots & 0 \\ \vdots & \vdots & \vdots & \vdots & \vdots & \ddots & \vdots \\ 0 & 0 & 0 & 0 & 0 & \cdots & 1 \end{bmatrix}$$

$$= \begin{bmatrix} 1 & -2 & 1 & 0 & 0 & \cdots & 0 \\ -2 & 5 & -4 & 1 & 0 & \cdots & 0 \\ 1 & -4 & 6 & -4 & 1 & \cdots & 0 \\ 0 & 1 & -4 & 6 & -4 & \cdots & 0 \\ 0 & 0 & 1 & -4 & 6 & \cdots & 0 \\ \vdots & \vdots & \vdots & \vdots & \vdots & \ddots & \vdots \\ 0 & 0 & 0 & 0 & 0 & \cdots & 1 \end{bmatrix}.$$

P is multiplied by the estimated precision parameters λ_c, λ_r, λ_s, and λ_g, thus up-weighting or down-weighting the strength of its penalties and ultimately determining the degree of smoothing at each level. For each of the four precision parameters, we use a truncated flat prior on the standard deviation scale $(1/\sqrt{\lambda})$ as recommended by Gelman.[8] We truncate these priors such that $\log\lambda \leq 20$ for each of the four λ's. This upper bound is enforced as a computational convenience: models with $\log\lambda > 20$ are treated as equivalent to a model with $\log\lambda = 20$ as they essentially have no extra-linear variability in time. In practice, this upper bound has little effect on the parameter estimates. Furthermore, we order the λ's a priori: $\lambda_c < \lambda_r < \lambda_s < \lambda_g$. This prior constraint conveys the natural expectation that, for example, the global BMI trend has less extra-linear variability than the trend of any given region.

The matrix P has rank $T - 2$, corresponding to a flat, improper prior on the mean and the slope of the u_j^c's, the u_k^r's, the u_l^s's and of u^g, and is not invertible.[12] Thus, we have a proper prior in a reduced-dimension space as discussed in Rue and Held,[11] with the prior expressed as:

$$P\left(u_j^c \middle| \lambda_c\right) \propto \lambda_c^{\frac{T-2}{2}} \exp\left\{-\frac{\lambda_c}{2} u_j^{c\prime} P u_j^c\right\}$$

Note that if u_j^c had a non-zero mean, this would introduce non-identifiability with respect to a_j^c. By the same token, b_j^c would not be identified if u_j had a non-zero time slope. Thus, in order to achieve identifiability of the a's, b's, and u's, we constrain the mean and slope of u^g and of each u^s, u^r, and u^c to be zero. Enforcing orthogonality between the linear and non-linear portions of the time trends means that each can be interpreted independently.

In cases where we have observations for at least two different time points, this improper prior will not lead to an improper posterior since the data will provide information about the mean

and slope. However, to enforce the desired orthogonality between the linear and non-linear portions of the model, we constrain the mean and slope of the u_j^c's, u_k^r's, u_l^s's, and of u^g to be zero, using the approach described by Rue and Held.[11]

For the few countries with no data, we take the Moore-Penrose pseudoinverse[13] of P, setting to infinity those eigenvalues that correspond to the non-identifiability. This effectively constrains the non-identified portions of the model to zero, as the corresponding variances are set to zero;[10] in this case the Rue and Held correction[11] is not needed. An intermediate case occurs when data are observed for only one time point in a country. In this case, the full conditional precision has rank $T-1$ because the mean but not the linear trend of u_j^c is identified by the data. We thus constrain the linear trend of u_j^c to zero by taking the generalized inverse of the full conditional precision. We then constrain the mean of u_j^c to zero using the one-dimensional version of the correction described in Rue and Held.[11]

2.3 Age model

The age association of BMI can be non-linear with a plateau or decline in older ages.[14-16] We therefore model age using cubic splines, treating the mid-age of each aggregate age group as a continuous variable. For age group h with a mid-point at z, in study i, the age effect is given by:

$$\gamma_i(z_h) = \gamma_{1i} z_h + \gamma_{2i} z_h^2 + \gamma_{3i} z_h^3 + \gamma_{4i}(z_h - 45)_+^3 + \gamma_{5i}(z_h - 60)_+^3$$

Spline knots are placed at ages 45 and 60 years, based on the age trajectory observed in the data. To reduce dependence among model parameters, we use a middle-aged group, specifically 50-year-olds, as the baseline age. The age effect coefficients ($\gamma_{p,i}$) for each age group mid-age (z_h) are given by:

$$\gamma_{p,i} = \psi_p + \phi_p \mu_i + c_{p,j[i]} \quad \text{(where } p = \{1,2,3,4,5\}\text{)}$$

BMI generally rises more steeply with age where BMI in the baseline age group is higher. In order to allow the shape of the curve to vary with BMI level, the spline coefficients for study i ($\gamma_{1,i},\ldots\gamma_{5,i}$) are allowed to depend on μ_i, the estimated BMI value for a 50-year-old for that study:

$$\mu_i = a_{j[i]} + b_{j[i]} t_i + \mu_{j[i],t_i} + \boldsymbol{X_i \beta} + e_i + I_{s,i}\left[p_{j[i]} + q_{j[i]} t_i + d_i\right]$$

The age association may also vary across countries beyond what can be explained by differences in country means. We therefore include country-specific random spline coefficients ($c_{p,j}$) in our specification of the γ's as well:

$$c_{p,j} \sim N(0, \sigma_p^2) \quad \text{(where, } p = \{1,2,3,4,5\}\text{)}$$

A flat improper prior is placed on each of the five σ's.

2.4 Study-level term and study-specific random effects

Individual studies may deviate from the true country-year mean. We use a study-level term to help account for potential systematic differences associated with data sources that are representative of sub-national and community populations. Our model thus includes time-varying offsets for sub-national and community data in the term $\boldsymbol{X_i \beta}$:

$$\boldsymbol{X_i \beta} = \beta_1 I\left\{X_{j[i],t[i]}^{cvrg} = \text{subnational}\right\} + \beta_2 I\left\{X_{j[i],t[i]}^{cvrg} = \text{subnational}\right\} t_i + \beta_3 I\left\{X_{j[i],t[i]}^{cvrg} = \text{community}\right\} + \beta_4 I\left\{X_{j[i],t[i]}^{cvrg} = \text{community}\right\} t_i$$

where $X_{j[i],t[i]}^{cvrg}$ is the indicator for whether the coverage of study i, in country j and year t, is sub-national or community.

Even after accounting for sampling variability, national studies may still not reflect the country's true mean BMI level with perfect accuracy, and sub-national and community studies have even larger variability. In study i, the study-specific random effect e_i allows all age groups from the same study to have an unusually high or an unusually low mean after conditioning on the other terms in the model. Each e_i is assigned a normal prior with variance depending on whether study i is representative at the national, sub-national or community level. Random effects from national studies are constrained to have smaller variance (ν_n) than random effects of sub-national studies (ν_s), which are in turn constrained to have smaller variance than community studies (ν_c). To make country-level predictions, we set e_i = 0, thus not including random effects due to imperfections in study design and to within-country variability of BMI means.

2.5 Residual age-by-study variability

The age patterns across communities within a given country may differ from their country's overall age pattern. This within-study variability cannot be captured by the e's, which are equal across age-specific observations in each study, so we include an additional variance component for each study, τ_i^2. We again assume that there is less residual variability in national studies than in sub-national and community-level studies, with $\tau_n^2 < \tau_s^2 < \tau_c^2$.

2.6 Urban and rural strata

To model mean BMI flexibly by urban and rural places of residence, the model incorporates country-specific intercept and slope offsets for the two strata using a centred indicator term ($I_{s,i}$):

$$I_{s,i}[p_{j[i]} + q_{j[i]}t_i + d_i] \quad (\text{where}, I_{s,i} = -1 + 2X_{s,i}^{urb})$$

with

$$X_{s,i}^{urb} = \begin{cases} 1, & \text{if stratum } s \text{ contains only urban individuals} \\ 0, & \text{if stratum } s \text{ contains only rural individuals} \\ X_{j[i],t[i]}^{urb} & \text{if stratum s contains a mixture of urban and rural individuals} \end{cases}$$

In other words, for data not stratified by place of residence, the model treats the unstratified mean BMI as equivalent to the weighted sum of the (unobserved) urban sample mean BMI and rural sample mean BMI, with the weights based on the proportion of the study country's population living in urban areas in the year of the survey ($X_{j[i],t[i]}^{urb}$).

The intercept (p) and slope (q) terms capture the country-to-country variation in the magnitude of the BMI difference between urban and rural populations and how the difference changes over time. These are specified with the same geographical hierarchy as the country-specific intercepts (a) and slopes (b):

$$p_j = p_j^c + p_{k[j]}^r + p_{l[k]}^s + p^g,$$

$$q_j = q_j^c + q_{k[j]}^r + q_{l[k]}^s + q^g,$$

$$p_j^x \sim N(0, \kappa_p^x),$$

$$q_j^x \sim N(0, \kappa_q^x) \qquad (\text{where}, x = \{c, r, s\})$$

The study random effect term d_i incorporates deviations from the country-level urban-rural difference in each study and is analogous to e_i.

2.7 Computation and inference

The model was fitted with the Markov chain Monte Carlo (MCMC) algorithm, with the sampler programmed using the statistical computing language R (the computer code is available at www.ncdrisc.org). For each sex, the model was run with 25 random starting values, and different seeds for the random number generation in the MCMC algorithm, for 55,000 iterations. Convergence of each model run (referred to as a 'chain' hereinafter) was monitored. Within each chain, post-burn-in iterations were thinned to 5,000 draws, which were then combined for all chains and further thinned to a final set of 5,000 draws that each contained the model parameter estimates.

The final set of model parameters was used to obtain 5,000 draws from the posterior distributions of the primary outcomes (mean urban BMI, mean rural BMI and mean urban-rural BMI difference), which allowed inferences to be made for each country-stratum-year-age unit, with parameters that corresponded to a national study; study-specific random effects were set to zero for this purpose. Posterior estimates were made in 1-year age groups for ages 18 and 19 years, 5-year age groups for those aged 20 to 84 years, and for the open-ended age group of 85 years and older.

Supplementary Table 1. Data sources used in the analysis.

Country	Data years	Survey/study name/citation	Level of representative-ness	Rural, urban or both	Age range as used for global analysis		Sample size as used for global analysis		Note
					Male	Female	Male	Female	
Afghanistan	2013	National Nutrition Survey	National	both		18-49		12987	*
Albania	2001	Shapo et al., Public Health Nutr 6:471-77, 2003	Community	urban	24+	24+	535	585	*
Albania	2008-2009	DHS	National	both	18-49	18-49	2534	6372	†
Algeria	2003	STEPS	Subnational	both	25-64	25-64	1612	2437	†
Algeria	2005	Transition and Health Impact in North Africa	National	both	35-70	35-70	2004	2739	*
Algeria	2007-2009	The ISOR (InSulino-resistance in ORan) study	Community	urban	30-64	30-64	378	409	*
Algeria	2017	STEPS	National	both	18-69	18-69	2991	3633	†
American Samoa	1990	McGarvey, Pac Health Dialog 8(1):157-62, 2001	National	both	25+	25+	359	484	*
American Samoa	1992	McGarvey, Pac Health Dialog 8(1):157-62, 2001	National	both	27+	27+	231	336	*
American Samoa	1994	McGarvey, Pac Health Dialog 8(1):157-62, 2001	National	both	29+	29+	164	245	*
American Samoa	2004	STEPS	National	both	25-64	25-64	949	1060	†
Argentina	1985-1986	INTERSALT	Community	urban	20-59	20-59	100	100	†
Argentina	1995-1998	de Sereday et al., Diabetes Metab 30:335-9, 2004	Subnational	urban	18-74	18-74	924	1246	*
Argentina	2003	CEDES-Programa VIGI+A-Banco Mundial	Community	urban	18-74	18-74	151	176	*
Argentina	2004-2005	CArdiovascular Risk factors Multiple Evaluation in Latin America (CARMELA)	Community	urban	25-64	25-64	733	742	*
Argentina	2005	Encuensta Nacional de Nutrición y Salud 2005	National	both		18-49		4803	*
Argentina	2006	Virasoro Survey	Community	urban	18-84	18-84	261	306	*
Argentina	2008-2011	The VELA Project	Community	rural	18+	18+	146	273	*
Argentina	2011	Primera Encuesta Alimentaria y Nutricional de la Ciudad Autónoma de Buenos Aires - EAN CABA	Community	urban	18+	18+	245	1288	*
Argentina	2011-2012	CESCAS Study	Community	urban	35-74	35-74	1584	2395	*
Argentina	2012-2013	Primer estudio sobre el estado nutricional y los hábitos alimentarios de la población adulta de Rosario	Community	urban	18-70	18-70	371	823	*
Armenia	1998	The health and nutritional status of children and women in Armenia	National	both		18-45		2397	*
Armenia	2000	DHS	National	both		18-49		5264	†
Armenia	2005	DHS	National	both	18-49	18-49	1025	5545	†
Armenia	2015-2016	DHS	National	both		18-49		5292	†
Armenia	2016	STEPS	National	both	18-69	18-69	604	1447	†
Australia	1983	Risk Factor Prevalence Study	National	urban	25-64	25-64	3731	3813	1*
Australia	1988-1989	Dubbo Study of Australian Elderly	Community	urban	59+	59+	877	1219	*
Australia	1988-1989	MONICA, Newcastle	Subnational	urban	35-64	35-64	672	671	†
Australia	1988-1989	MONICA, Newcastle	Community	urban	25-34	25-34	70	84	†
Australia	1989	Risk Factor Prevalence Study	National	urban	20-69	20-69	4497	4678	*
Australia	1992-1993	Australia Longitudinal Study of Ageing	Community	urban	65+	65+	814	746	*
Australia	1994	MONICA, Newcastle	Subnational	urban	35-64	35-64	637	688	†
Australia	1994	MONICA, Perth inner	Community	urban	25-64	25-64	363	349	†
Australia	1994	MONICA, Perth outer	Community	urban	25-64	25-64	373	387	†
Australia	1995	National Nutrition Survey	National	both	18+	18+	4864	5352	*
Australia	1996-1998	Western Australian AAA Screening Program	Community	urban	65-84		12194		*
Australia	1999-2000	The Australian Diabetes, Obesity and Lifestyle Study 1999-2000	National	both	25+	25+	4991	6070	*
Australia	1999-2003	North West Adelaide Health Study	Community	urban	18+	18+	1932	2122	*
Australia	2000	Perth children	Community	both	25	25	266	334	*
Australia	2004-2005	Janus et al., Med J Aust 187:147-52, 2007	Community	rural	25-74	25-74	383	423	*
Australia	2004-2005	The Australian Diabetes, Obesity and Lifestyle Study 2004-2005	National	both	30+	30+	2874	3472	*
Australia	2004-2006	North West Adelaide Health Study	Community	urban	20+	20+	1523	1679	*
Australia	2007-2008	National Health Survey	National	both	18+	18+	5279	5655	*
Australia	2008-2010	North West Adelaide Health Study	Community	urban	24+	24+	1168	1318	*
Australia	2011-2012	Australian Health Survey	National	both	18+	18+	9975	10849	*
Australia	2012	The Australian Diabetes, Obesity and Lifestyle Study 2012	National	both	37+	37+	2048	2530	*
Australia	2014-2015	National Health Survey	National	both	18+	18+	6653	7907	*
Austria	1983	The Austrian Conscription Database	National	both	18		28094		*
Austria	1984	The Austrian Conscription Database	National	both	18		28202		*
Austria	1985	The Austrian Conscription Database	National	both	18		28938		*
Austria	1986	CINDI	Community	both	25-64	25-64	657	715	*
Austria	1986	The Austrian Conscription Database	National	both	18		28923		*
Austria	1987	The Austrian Conscription Database	National	both	18		27534		*
Austria	1988	The Austrian Conscription Database	National	both	18		25185		*
Austria	1989	The Austrian Conscription Database	National	both	18		24628		*
Austria	1990	The Austrian Conscription Database	National	both	18		23826		*
Austria	1991	The Austrian Conscription Database	National	both	18		22453		*
Austria	1992	The Austrian Conscription Database	National	both	18		22127		*

Country	Data years	Survey/study name/citation	Level of representative-ness	Rural, urban or both	Age range as used for global analysis		Sample size as used for global analysis		Note
					Male	Female	Male	Female	
Austria	1992	Vorarlberg Health Monitoring and Promotion Programme	Subnational	both	18+	18+	14161	18835	*
Austria	1993	The Austrian Conscription Database	National	both	18		21953		*
Austria	1994	The Austrian Conscription Database	National	both	18		20846		*
Austria	1995	The Austrian Conscription Database	National	both	18		20097		*
Austria	1996	The Austrian Conscription Database	National	both	18		19710		*
Austria	1997	The Austrian Conscription Database	National	both	18		20526		*
Austria	1998	The Austrian Conscription Database	National	both	18		21262		*
Austria	1998	Vorarlberg Health Monitoring and Promotion Programme	Subnational	both	18+	18+	16153	20915	*
Austria	1999	The Austrian Conscription Database	National	both	18		21591		*
Austria	2000	The Austrian Conscription Database	National	both	18		21834		*
Austria	2001	The Austrian Conscription Database	National	both	18		21828		*
Austria	2002	The Austrian Conscription Database	National	both	18		20909		*
Austria	2003	The Austrian Conscription Database	National	both	18		20787		*
Austria	2004	The Austrian Conscription Database	National	both	18		20672		*
Austria	2004	Vorarlberg Health Monitoring and Promotion Programme	Subnational	both	18+	18+	20160	23893	*
Austria	2005	The Austrian Conscription Database	National	both	18		20499		*
Austria	2006	The Austrian Conscription Database	National	both	18		21658		*
Austria	2007	The Austrian Conscription Database	National	both	18		21405		*
Austria	2008	The Austrian Conscription Database	National	both	18		21513		*
Austria	2009	The Austrian Conscription Database	National	both	18		22359		*
Austria	2010	The Austrian Conscription Database	National	both	18		22180		*
Austria	2010-2012	Austrian Study on Nutritional Status 2012	National	both	18-80	18-80	156	235	*
Austria	2011	The Austrian Conscription Database	National	both	18		21763		*
Austria	2012	The Austrian Conscription Database	National	both	18		21126		*
Austria	2013	The Austrian Conscription Database	National	both	18		20610		*
Austria	2014	The Austrian Conscription Database	National	both	18		19907		*
Austria	2015	The Austrian Conscription Database	National	both	18		19245		*
Austria	2016	The Austrian Conscription Database	National	both	18		18095		*
Austria	2017	The Austrian Conscription Database	National	both	18		17285		*
Azerbaijan	1996	Health and Nutrition Survey	National	both	19-59	19-59	121	295	*
Azerbaijan	2001	Reproductive Health Survey (RHS)	National	both		18-44		1726	*
Azerbaijan	2006	DHS	National	both	18-59	18-49	2208	7044	†
Azerbaijan	2017	STEPS	National	both	18--69	18-69	1117	1577	†
Bahrain	1991-1992	al-Mannai et al., J R Soc Health 116:30-2, 37-40, 1996	Community	both	20+	20+	137	153	*
Bahrain	1995	Musaiger et al., Ann Hum Biol 28:346-50, 2001	Community	both	30+	30+	298	216	*
Bahrain	1998-1999	National Nutrition Survey	National	both	19+	19+	1120	1181	*
Bahrain	2001-2004	Global database on growth and malnutrition of school children and adolescents, WHO	National	both	18-19	18-20	100	102	*
Bahrain	2007	STEPS	National	both	20-64	20-64	854	858	*
Bangladesh	1992	Rahman et al., Hypertension 33:74-8, 1999	Community	rural	30+	30+	965	643	*
Bangladesh	1996-1997	DHS	National	both		20-49		3384	†
Bangladesh	1998	Zaman et al., J Health Popul Nutr 21:162-63, 2003	Community	rural	20+	20+	290	379	*
Bangladesh	1999-2000	Hussain et al., Eur J Public Health 17:291-96, 2007	Community	rural	20-59	20-59	2037	2720	*
Bangladesh	1999-2000	DHS	National	both		20-49		3887	†
Bangladesh	2000-2004	Nutritional Surveillance Project	National	rural		18-45		224251	*
Bangladesh	2002	STEPS	National	both	25-64	25-64	5619	5775	*
Bangladesh	2004	DHS	National	both		20-49		9165	†
Bangladesh	2007	DHS	National	both		20-49		9037	†
Bangladesh	2011	DHS	National	both	18+	20+	5250	16679	†
Bangladesh	2014	DHS	National	both		20-49		14963	†
Bangladesh	2015	An assessment of BRAC Health Nutrition and Population Programme and benchmark survey of Sustainable Development Go	National	rural	35+	18+	5432	16132	*
Barbados	1987-1992	Barbados Eye Study	National	both	40-84	40-84	1980	2627	*
Barbados	1991-1994	Cooper et al., Am J Public Health 87(2):160-68, 1997	Community	urban	25-100	25-100	329	482	*
Barbados	1997-2002	The Barbados Incidence Studies of Eye Diseases II	National	both	40-84	40-84	1004	1441	*
Barbados	1999-2000	The Survey on Health, Well-Being, and Aging in Latin America and the Caribbean (SABE)	Community	urban	60+	60+	559	866	2†
Barbados	2011-2013	Health of the Nation (HotN)	National	both	25+	25+	455	703	*
Belarus	2016	STEPS	National	both	18-69	18-69	2085	2894	†
Belgium	1984-1985	Belgian Interuniversity Research on Nutrition and Health	National	both	25-74	25-74	5897	5289	*
Belgium	1985-1986	INTERSALT, Ghent	Community	urban	20-59	20-59	100	100	†
Belgium	1985-1987	INTERSALT, Charleroi	Community	urban	20-59	20-59	82	75	†

Country	Data years	Survey/study name/citation	Level of representative-ness	Rural, urban or both	Age range as used for global analysis		Sample size as used for global analysis		Note
					Male	Female	Male	Female	
Belgium	1985-1987	MONICA, Charleroi	Community	urban	25-64	25-64	347	327	†
Belgium	1985-1987	MONICA, Ghent	Community	urban	25-64	25-64	549	459	†
Belgium	1985-1990	Flemish Study on Environment, Genes and Health Outcomes	Community	rural	20-90	20-90	656	692	*
Belgium	1987-1990	MONICA, Charleroi	Community	urban	25-64	25-64	325	301	†
Belgium	1988-1990	MONICA, Ghent	Community	urban	25-64	25-64	456	449	†
Belgium	1990-1992	MONICA, Ghent	Community	urban	25-64	25-64	507	475	†
Belgium	1990-1993	MONICA, Charleroi	Community	urban	25-64	25-64	337	332	†
Belgium	1991-1994	Flemish Study on Environment, Genes and Health Outcomes	Community	rural	26-88	26-88	393	416	*
Belgium	1992-1995	Flemish Study on Environment, Genes and Health Outcomes	Community	rural	27-89	27-89	298	312	*
Belgium	1994-1996	BIRNH Elderly: Belgian Interuniversity Research on Nutrition and Health in the Elderly	National	both	65-89	65-89	1142	953	*
Belgium	1996-1998	Flemish Study on Environment, Genes and Health Outcomes	Community	rural	18-84	18-84	354	347	*
Belgium	1998	Flemish Study on Environment, Genes and Health Outcomes	Community	rural	32-86	32-86	320	359	*
Belgium	1998-2000	Flemish Study on Environment, Genes and Health Outcomes	Community	rural	18-80	18-80	198	196	*
Belgium	1999-2001	Flemish Study on Environment, Genes and Health Outcomes	Community	rural	18-81	18-81	213	227	*
Belgium	2001	Flemish Study on Environment, Genes and Health Outcomes	Community	rural	18-78	18-78	230	208	*
Belgium	2002-2003	Flemish Study on Environment, Genes and Health Outcomes	Community	rural	18-81	18-81	169	181	*
Belgium	2002-2004	SPAH	Subnational	both	18-75	18-75	2595	2308	*
Belgium	2002-2005	Flemish Study on Environment, Genes and Health Outcomes	Community	rural	18-88	18-88	391	404	*
Belgium	2003	The European Male Ageing Study	Community	both	40+		433		*
Belgium	2005-2008	Flemish Study on Environment, Genes and Health Outcomes	Community	rural	18-89	18-89	449	456	*
Belgium	2008	The European Male Ageing Study	Community	both	40+		383		*
Belgium	2009-2013	Flemish Study on Environment, Genes and Health Outcomes	Community	rural	20-88	20-88	330	335	*
Belgium	2010-2015	Flemish Study on Environment, Genes and Health Outcomes	Community	rural	18-87	18-87	388	410	*
Belgium	2014-2015	Food Consumption Survey	National	urban	18-64	18-64	611	633	*
Belize	2004-2005	CAMDI	National	both	20+	20+	599	1018	*
Benin	1996	DHS	National	both		20-49		2137	†
Benin	2001	DHS	National	both		18-49		4787	†
Benin	2006	DHS	National	both		18-49		13293	†
Benin	2007	STEPS	Community	urban	25-64	25-64	955	1508	†
Benin	2008	STEPS	National	both	25-64	25-64	3430	3365	†
Benin	2011-2012	DHS	National	both		18-49		12833	†
Benin	2015	STEPS	National	both	18-69	18-69	2304	2543	†
Bhutan	2007	STEPS	Community	urban	25-74	25-74	1125	1322	†
Bhutan	2014	STEPS	National	both	18-69	18-69	1069	1674	†
Bolivia	1994	DHS	National	both		20-49		2128	†
Bolivia	1998	DHS	National	both		20-49		3939	†
Bolivia	2003	DHS	National	both		18-49		13974	†
Bolivia	2005-2007	Baya Botti et al., Nutr Hosp 24(3):304-11, 2009	National	both	18	18	137	131	*
Bolivia	2008	DHS	National	both		18-49		13497	†
Bosnia and Herzegovina	2002	Non-communicable disease risk factor survey, Federation of B&H	Subnational	both	25-64	25-64	1118	1613	*
Bosnia and Herzegovina	2012	Non-communicable disease risk factor survey, Federation of B&H	Subnational	both	18+	18+	1782	1971	*
Botswana	2007	STEPS	National	both	25-64	25-64	1243	2577	†
Botswana	2014	STEPS	National	both	18-69	18-69	1236	2500	†
Brazil	1989	Pesquisa Nacional sobre Saude e Nutricao	National	both	18+	18+	16783	17897	*
Brazil	1990-1991	Fornes et al., Rev Saude Publica 36:12-8, 2002	Community	urban	20+	20+	432	613	*
Brazil	1991-1993	EPIDOSO	Community	urban	65+	65+	269	473	*
Brazil	1992-1998	Moraes et al., Int J Cardiol 90:205-11, 2003	Community	urban	18+	18+	438	543	*
Brazil	1995	Health and Nutrition Survey of Rio de Janeiro	Community	urban	60+	60+	248	385	*
Brazil	1995-1996	Cohort study from Porto Alegre	Community	urban	18+	18+	489	596	*
Brazil	1996	DHS	National	both		20-49		2884	†
Brazil	1996-1997	The Bambui Cohort Study of Ageing	Community	urban	18+	18+	931	1335	*
Brazil	1997	PPV	Subnational	both	20+	20+	8063	9121	*
Brazil	1998	Belo Horizonte Heart Study	Community	urban	18-19	18-19	31	43	*
Brazil	1999-2000	Pelotas cross-sectional survey	Community	urban	20-69	20-69	839	1096	*
Brazil	1999-2000	The Survey on Health, Well-Being, and Aging in Latin America and the Caribbean (SABE)	Community	urban	60+	60+	732	1064	2†
Brazil	2000	The 1982 Pelotas (Brazil) Birth Cohort: 18 years follow-up	Community	urban	18		2228		*
Brazil	2001	de Freitas et al., Arq Bras Cardiol 88:191-99, 2007	Community	urban	18+	18+	310	331	*
Brazil	2001	The 1982 Pelotas (Brazil) Birth Cohort: 19 years follow-up	Community	urban		19		919	*
Brazil	2001-2003	Bustos et al., Nutr Metab Cardiovasc Dis 17:581-89, 2007	Community	both	22-28	22-28	992	1064	*

Country	Data years	Survey/study name/citation	Level of representative-ness	Rural, urban or both	Age range as used for global analysis		Sample size as used for global analysis		Note
					Male	Female	Male	Female	
Brazil	2002-2003	Pesquisa de Orcamentos Familiares	National	both	18+	18+	55681	56389	*
Brazil	2002-2004	Ribeira Preto Birth Cohort	Community	urban	22-25	22-25	1012	1082	*
Brazil	2003	PNAFS	Community	urban	20+	20+	1155	1941	*
Brazil	2003	Women health in Southern Brazil	Community	urban		20-60		986	*
Brazil	2003-2005	São Paulo Health and Ageing Study	Community	urban	65+	65+	783	1198	*
Brazil	2004	Caju and Virgem das Graças	Community	rural	18+	18+	291	286	*
Brazil	2004-2005	The 1982 Pelotas (Brazil) Birth Cohort: 23 years follow-up	Community	urban	23	23	2173	1935	*
Brazil	2004-2006	Hearts of Brazil	National	urban	18+	18+	550	626	*
Brazil	2006	Krause et al., J Aging Phys Act 17:387-97, 2009	Community	urban	60+	60+	93	1069	*
Brazil	2006	ATITUDE	Subnational	both	18-21	18-21	1314	1714	*
Brazil	2006	Pesquisa Nacional de Demografia e Saude 2006	National	both		18-49		13375	*
Brazil	2006-2007	SOFT study	Community	urban	18+	18+	739	1099	*
Brazil	2008	The Bambui Cohort Study of Ageing	Community	urban	71+	71+	248	456	*
Brazil	2008	Caju & Virgen das Gracas	Community	rural	18+	18+	273	287	*
Brazil	2008-2009	Pesquisa de Orcamentos Familiares	National	both	18+	18+	62035	65695	*
Brazil	2009-2010	EpiFloripa Cohort Study of Ageing - Wave 1	Community	urban	60+	60+	592	1047	*
Brazil	2010	San Pedro	Community	rural	18+	18+	153	214	*
Brazil	2010-2015	Baependi Heart Study	Community	urban	18+	18+	780	1125	*
Brazil	2011	ATITUDE	Subnational	both	18-19	18-19	687	839	*
Brazil	2011	Pregnancy in adolescence in municipalities of small size in the northeast of Brazil	Community	both	18-19	18-19	112	118	*
Brazil	2011-2012	The 1993 Pelotas (Brazil) Birth Cohort: 18 years follow-up	Community	urban	18-19	18-19	1953	1993	*
Brazil	2012-2013	The 1982 Pelotas (Brazil) Birth Cohort: 30 years follow-up	Community	urban	30	30	1753	1798	*
Brazil	2012-2013	Prevalence of Leptin Polymorphism Gln223Arg	Community	urban	18+	18+	282	523	*
Brazil	2013	Pesquisas Nacional de Saude	National	both	18+	18+	24918	32351	†
Brazil	2013-2014	EpiFloripa Cohort Study of Ageing - Wave 2	Community	urban	63+	63+	404	744	*
Brazil	2014	Brazilian Guide to the Physical Fitnees related to Health Assessment and Lifestyle Habits	Community	urban	18-19	18-19	73	45	*
Brazil	2014-2015	EpiFloripa Adults Cohort Study	Community	urban	25-65	25-65	353	476	*
Brazil	2015-2016	The 1993 Pelotas (Brazil) Birth Cohort: 22-23 years follow-up	Community	urban	21-23	21-23	1687	1872	*
Brazil	2016-2017	Study in Presidente Prudente	Community	urban	18+	18+	314	502	*
Brunei Darussalam	2010-2011	National Health And Nutritional Status Survey (NHANSS)	National	both	18-75	18-75	708	847	*
Brunei Darussalam	2015-2016	National Non-Communicable Diseases Survey (NNCDS)	National	both	18-69	18-69	814	1075	*
Bulgaria	2004	National Nutrition Survey	National	both	18+	18+	515	515	*
Burkina Faso	1992-1993	DHS	National	both		20-49		3190	†
Burkina Faso	1998-1999	DHS	National	both		20-49		3114	†
Burkina Faso	2002	Vulnérabilité Alimentaire et Sécurité Nutritionnelle dans la Gnagna (VASN-Gnagna)	Subnational	rural	18+	18+	733	2663	*
Burkina Faso	2003	DHS	National	both		18-49		9360	†
Burkina Faso	2004	Ouedraogo et al., Public Health Nutr 11:1280-87, 2008	Community	urban	35+	35+	956	1066	*
Burkina Faso	2010	DHS	National	both		18-49		6730	†
Burkina Faso	2013	STEPS	National	both	25-64	25-64	2223	2250	†
Burundi	2010	DHS	National	both		18-49		3471	†
Burundi	2016-2017	DHS	National	both		18-49		6651	†
Cabo Verde	2007	STEPS	National	both	25-64	25-64	658	1066	†
Cambodia	2000	DHS	National	both		18-49		5888	†
Cambodia	2005	DHS	National	both		18-49		6952	†
Cambodia	2008	Anthropometrics Survey	National	both		18-49		5955	*
Cambodia	2010	DHS	National	both		18-49		7650	†
Cambodia	2010	STEPS	National	both	25-64	25-64	1881	3344	†
Cambodia	2014	DHS	National	both		18-49		9686	†
Cameroon	1998	DHS	National	both		20-49		1429	†
Cameroon	1998-1999	ENHIP	Community	both	18+	18+	921	1237	*
Cameroon	2003	STEPS	Subnational	urban	18+	18+	3331	4991	†
Cameroon	2004	DHS	National	both		18-49		3924	†
Cameroon	2007	Cameroon Burden of Diabetes - Second Survey	Subnational	urban	18+	18+	3122	4123	*
Cameroon	2009	National Survey of Micronutrient Status and Consumption of Fortifiable Foods	National	both		18-49		788	*
Cameroon	2011	DHS	National	both		18-49		6281	†
Cameroon	2014-2015	Cardiovascular risk factors screening in urban and rural areas in the Far-North Region Cameroon	Subnational	both	20+	20+	520	369	*
Canada	1985-1986	INTERSALT, St Johns	Community	urban	20-59	20-59	100	100	†
Canada	1985-1988	MONICA, Halifax	Community	both	25-64	25-64	438	420	†
Canada	1986-1992	Canada Heart Health Survey	National	both	18-74	18-74	8789	8877	*

Country	Data years	Survey/study name/citation	Level of representative-ness	Rural, urban or both	Age range as used for global analysis		Sample size as used for global analysis		Note
					Male	Female	Male	Female	
Canada	1991-1992	Canadian Study of Health and Aging	Community	both	70+	70+	236	348	*
Canada	1993	Chen et al., Int J Obes Relat Metab Disord 22:771-77, 1998	Community	rural	18-74	18-74	803	988	*
Canada	1995	MONICA, Halifax	Community	both	25-64	25-64	274	287	†
Canada	1995-1997	Canadian Multicentre Osteoporosis Study (CaMos)	Subnational	both	25+	25+	2610	6332	*
Canada	1996	Canadian Study of Health and Aging	Community	both	70+	70+	236	348	*
Canada	1997	PEI Nutrition Survey	Subnational	both	18-74	18-74	1000	995	*
Canada	2005	CCHS	National	both	18+	18+	1684	2031	*
Canada	2005-2008	Canadian Multicentre Osteoporosis Study (CaMos)	Subnational	both	35+	35+	1486	3661	*
Canada	2007-2009	Canadian Health Measures Survey, Cycle 1	National	both	18-79	18-79	1744	1947	*
Canada	2008	CCHS	National	both	18+	18+	1689	1988	*
Canada	2009-2011	Canadian Health Measures Survey, Cycle 2	National	both	18-79	18-79	1785	2047	*
Canada	2012-2013	Canadian Health Measures Survey, Cycle 3	National	both	18-79	18-79	1666	1688	*
Canada	2014-2015	Canadian Health Measures Survey, Cycle 4	National	both	18-79	18-79	1683	1669	*
Central African Republic	1994-1995	DHS	National	both		20-49		1760	†
Central African Republic	2010	STEPS	Subnational	both	25-64	25-64	1846	1967	†
Chad	1996-1997	DHS	National	both		20-49		3262	†
Chad	2004	DHS	National	both		20-49		2618	†
Chad	2008	STEPS	Community	urban	25-64	25-64	995	845	†
Chad	2014-2015	DHS	National	both		18-49		8369	†
Chile	1989	INCLEN	Community	urban	35-65		199		*
Chile	1992-1993	Miquel et al., Gastroenterology 115:937-46, 1998	Community	urban	18+	18+	657	1031	*
Chile	1999-2000	The Survey on Health, Well-Being, and Aging in Latin America and the Caribbean (SABE)	Community	urban	60+	60+	410	806	2†
Chile	2000	Nervi et al., J Hepatol 45:299-305, 2006	Community	urban	18+	18+	335	624	*
Chile	2001-2003	Bustos et al., Nutr Metab Cardiovasc Dis 17:581-89, 2007	Community	both	22-28	22-28	436	562	*
Chile	2003	Encuesta Nacional de Salud	National	both	18+	18+	1545	1849	*
Chile	2004-2005	CArdiovascular Risk factors Multiple Evaluation in Latin America (CARMELA)	Community	urban	25-64	25-64	783	865	*
Chile	2005	Palomo et al., Rev Med Chil 135:904-12, 2007	Community	urban	18-74	18-74	339	668	*
Chile	2009-2010	Encuesta Nacional de Salud	National	both	18+	18+	1830	2773	*
Chile	2011-2012	CESCAS Study	Community	urban	35-74	35-74	922	1027	*
Chile	2016-2017	Encuesta Nacional de Salud	National	both	18+	18+	1878	3315	*
China	1982	China National Nutrition Survey	National	both	18+	18+	8920	8755	1*
China	1984-1985	Sino-MONICA Beijing	Subnational	both	25-64	25-64	813	857	†
China	1985	Chinese National Surveys on Students Constitution and Health	National	both	18	18	16847	16596	*
China	1986	INTERSALT, Beijing	Community	urban	20-59	20-59	100	100	†
China	1986	INTERSALT, Nanning	Community	urban	20-59	20-59	100	100	†
China	1986	INTERSALT, Tianjin	Community	urban	20-59	20-59	100	100	†
China	1986-1989	Wang et al., Zhonghua Liu Xing Bing Xue Za Zhi 26:394-9, 2005	Community	both	45-64		18244		*
China	1986-1989	Sino-MONICA Shanghai	Subnational	rural	25-64	25-64	675	753	*
China	1987	INCLEN	Community	urban	35-65		989		*
China	1988	Sino-MONICA Hebei	Subnational	both	25-64		800		*
China	1988	Sino-MONICA Heilongjiang	Subnational	urban	25-64	25-64	800	800	*
China	1988	Sino-MONICA Henan	Subnational	urban	25-64	25-64	345	427	*
China	1988	Sino-MONICA Neimenggu	Subnational	urban	25-64	25-64	396	400	*
China	1988	Sino-MONICA Sichuan	Subnational	both	25-64	25-64	312	334	*
China	1988	Sino-MONICA Shandong	Subnational	urban	25-64	25-64	211	225	*
China	1988-1989	Wang et al., Zhonghua Liu Xing Bing Xue Za Zhi 24:272-75, 2003	Community	both	25-64	25-64	873	731	*
China	1988-1989	Sino-MONICA Beijing	Subnational	both	25-64	25-64	701	862	*
China	1988-1989	Sino-MONICA Jilin	Subnational	urban	25-64	25-64	380	400	*
China	1988-1989	Sino-MONICA Jiangxi	Subnational	urban	25-64	25-64	379	386	*
China	1988-1989	Sino-MONICA Liaoning	Subnational	both	25-64	25-64	728	734	*
China	1988-1990	East Beijing Study 2	Community	urban	20-84	20-84	135	148	*
China	1989	The Tianjin Project	Community	urban	18-64	18-64	3894	3971	*
China	1989	China Health and Nutrition Study	National	both	18-45	18-45	2356	2549	3†
China	1989	Sino-MONICA Fujian	Subnational	urban	25-64	25-64	179	191	*
China	1989	Sino-MONICA Jiangsu	Subnational	rural	25-64	25-64	398	399	*
China	1990-1991	China Prospective Study	National	both	40-79		230676		*
China	1991	Hua et al., Zhonghua Nei Ke Za Zhi 36:18-20, 1997	Community	rural	60+	60+	288	335	*
China	1991	China National Hypertension Survey Epidemiology Follow-up Study	National	both	40+	40+	75696	79040	*
China	1991	China Health and Nutrition Study	National	both	18+	18+	4047	4470	3†

Country	Data years	Survey/study name/citation	Level of representative-ness	Rural, urban or both	Age range as used for global analysis		Sample size as used for global analysis		Note
					Male	Female	Male	Female	
China	1991	Sino-MONICA Shanghai	Subnational	rural	30-64	30-64	564	624	*
China	1991-1992	Fangshan Cohort Study	Community	urban	34-86	34-86	871	1736	*
China	1992	Huashan Study	Community	urban	35-75	35-75	892	965	*
China	1992	China National Nutrition Survey	National	both	18+	18+	23925	27306	*
China	1992	Sino-MONICA Sichuan	Subnational	both	25-64	25-64	608	526	*
China	1992-1993	Anzhen 02 Cohort Study	Community	urban	34-65	34-65	2032	2120	*
China	1993	Wang et al., Zhonghua Liu Xing Bing Xue Za Zhi 24:272-75, 2003	Community	both	25-64	25-64	822	617	*
China	1993	China Health and Nutrition Study	National	both	18+	18+	3864	4235	3†
China	1993	Sino-MONICA Anhui	Subnational	urban	25-64	25-64	193	195	*
China	1993	Sino-MONICA Beijing	Subnational	both	25-64	25-64	613	816	*
China	1993	Sino-MONICA Jiangsu	Subnational	urban	25-64	25-64	462	365	*
China	1993	Sino-MONICA Liaoning	Subnational	both	25-64	25-64	493	500	*
China	1995	Chinese National Surveys on Students Constitution and Health	National	both	18	18	8651	8454	*
China	1996	Wang et al., Zhonghua Liu Xing Bing Xue Za Zhi 24:272-75, 2003	Community	both	25-64	25-64	735	721	*
China	1996	The Tianjin Project	Community	urban	18-64	18-64	722	717	*
China	1996-2000	Shanghai Women's Health Study	Community	urban		40-70		74915	*
China	1996-2003	Wu et al., Osteoporos Int 15:751-59, 2004	Community	urban		18+		3418	*
China	1997	China Health and Nutrition Study	National	both	18+	18+	4164	4401	3†
China	1997	INTERMAP, Beijing	Community	rural	40-59	40-59	133	139	†
China	1997	INTERMAP, Guangxi	Community	rural	40-59	40-59	140	138	†
China	1997	INTERMAP, Shanxi	Community	rural	40-59	40-59	143	146	†
China	1998	Shanghai Diabetes Study	Community	urban	25+	25+	1264	1768	*
China	1998-2000	Jia et al., Obes Rev 3:157-65, 2002	Community	urban	20+	20+	1106	1670	*
China	1999	Chen et al., Zhonghua Yi Xue Za Zhi 85(40):2830-4, 2005	Subnational	both	35-85	35-85	13549	10315	*
China	1999	Wang et al., Zhonghua Liu Xing Bing Xue Za Zhi 24:272-75, 2003	Community	both	25-64	25-64	818	685	*
China	1999-2000	Xu et al., Public Health Nutr 8:47-51, 2005	Community	both	35+	35+	18194	18902	*
China	2000	China Health and Nutrition Study	National	both	18+	18+	4515	4934	3†
China	2000	Chinese National Surveys on Students Constitution and Health	National	both	18	18	9085	9162	*
China	2000-2001	The International Collaborative Study of Cardiovascular Disease in ASIA	National	both	35-74	35-74	7512	8006	*
China	2001	Shanghai Diabetes Study	Community	urban	25+	25+	1264	1768	*
China	2002	Ma et al., Zhonghua Liu Xing Bing Xue Za Zhi 25:1035-8, 2004	Subnational	both	18+	18+	7352	7352	*
China	2002	China National Nutrition and Health Survey	National	both	18+	18+	64630	75015	*
China	2002-2003	Fan et al., J Gastroenterol Hepatol 20:1825-32, 2005	Community	urban	18+	18-74	5502	7767	*
China	2002-2006	Shanghai Men's Health Study	Community	urban	40-74		61445		*
China	2004	Tian et al., Prev Med 48:59-63, 2009	Community	rural	18+	18+	1022669	1163313	*
China	2004	Beijing Child and Adolescent Metabolic Syndrome study	Community	both	18	18	248	258	*
China	2004	China Health and Nutrition Study	National	both	18+	18+	4345	4750	3†
China	2004-2005	Xinjiang Children and Adolescent Survey	Community	urban	18	18	115	123	*
China	2004-2006	Pang et al., Intern Med 47:893-97, 2008	Community	rural	35+	35+	22963	22962	*
China	2004-2006	Shanghai Women's Health Study	Community	urban		45-80		64545	*
China	2004-2008	China Kadoorie Biobank baseline survey	Subnational	both	35-74	35-74	205011	295708	†
China	2004-2008	Shanghai Men's Health Study	Community	urban	41-80		54800		*
China	2005	Ye et al., J Am Coll Cardiol 49:1798-805, 2007	Community	urban	50-70	50-70	743	906	*
China	2005	Chinese National Surveys on Students Constitution and Health	National	both	18	18	10117	10190	*
China	2005-2006	Zhou et al., World J Gastroenterol 13:6419-24, 2007	Community	urban	18-79	18-79	1101	2063	*
China	2006	Beijing Eye Study	Community	both	45+	45+	1394	1820	*
China	2006	China Health and Nutrition Study	National	both	18+	18+	4250	4781	3†
China	2007	Beijing Child and Adolescent Metabolic Syndrome study	Community	urban	18	18	20	18	*
China	2007-2008	China National Diabetes & Metabolic Disorders Study	National	both	20+	20+	18419	27820	*
China	2007-2010	SAGE	National	both	50+	50+	5759	6616	†
China	2007-2011	Shanghai Women's Health Study	Community	urban		47-83		52116	*
China	2008	China Health and Retirement Longitudinal Study (CHARLS), pilot survey	Subnational	both	45+	45+	923	950	†
China	2008-2010	Fangshan Family-based Ischemic Stroke Study in China (FISSIC) program	Community	rural	40+	40+	19478	36449	*
China	2008-2011	Shanghai Men's Health Study	Community	urban	43-84		51948		*
China	2009	China Health and Nutrition Study	National	both	18+	18+	4489	4936	3†
China	2010	China Noncommunicable Disease Surveillance	National	both	18+	18+	45066	53452	*
China	2010	Chinese National Surveys on Students Constitution and Health	National	both	18	18	8965	8923	*
China	2011	Beijing Children Eye Study	Community	both	18	18	615	588	*
China	2011	Beijing Eye Study	Community	both	50+	50+	1467	1895	*

Country	Data years	Survey/study name/citation	Level of representative-ness	Rural, urban or both	Age range as used for global analysis		Sample size as used for global analysis		Note
					Male	Female	Male	Female	
China	2011	China Health and Nutrition Study	National	both	18+	18+	5885	6646	3†
China	2011-2012	China Health and Retirement Longitudinal Study (CHARLS), baseline survey	National	both	45+	45+	6337	7003	†
China	2012	Beijing High School Eye Students Study	Community	both	18	18	352	370	*
China	2012	China Health and Retirement Longitudinal Study (CHARLS), wave 2 pilot survey	Subnational	both	45+	45+	777	856	†
China	2012	Shandong Children Study	Community	both	18	18	57	44	*
China	2012-2013	The Kailuan Study	Community	urban	18+	18+	80921	21385	*
China	2012-2015	Shanghai Men's Health Study	Community	urban	47-87		40921		*
China	2012-2015	Shanghai Women's Health Study	Community	urban		52-88		49592	*
China	2013	China Health and Retirement Longitudinal Study (CHARLS), wave 2 survey	National	both	45+	45+	5898	6582	*
China	2013	Gobi Desert Children Eye Study	Community	urban	18-21	18-21	28	25	*
China	2014	Shanghai Municipal Surveys on Students Constitution and Health	Community	both	18	18	404	395	*
China	2014-2015	The Kailuan Study	Community	urban	18+	18+	73161	18280	*
China	2016	Greater Beijing School Children Myopia Study	Subnational	both	18	18	628	638	*
China (Hong Kong SAR)	1985-1986	Shatin New Town Study	Community	urban	70+	70+	276	669	*
China (Hong Kong SAR)	1991	The Hong Kong study on health, health risk and quality of life in the Chinese elderly cohort	Community	both	70+	70+	943	944	*
China (Hong Kong SAR)	1995-1996	Hong Kong Cardiovascular Risk Factor Prevalence Study 1995-1996	National	both	25-74	25-74	1412	1478	*
China (Hong Kong SAR)	2005-2006	Hong Kong Growth Survey	National	both	18-19	18-19	920	951	*
Colombia	1986	INTERSALT	Community	rural	20-59	20-59	96	95	†
Colombia	1995	DHS	National	both		20-49		3068	†
Colombia	2000	DHS	National	both		20-49		2929	†
Colombia	2001	CINDI/CARMEN - Bucaramaga	Community	urban	18-74	18-74	627	1218	*
Colombia	2002	CINDI/CARMEN - Bogota	Community	urban	18-74	18-74	322	570	*
Colombia	2004-2005	CArdiovascular Risk factors Multiple Evaluation in Latin America (CARMELA)	Community	urban	25-64	25-64	738	812	*
Colombia	2005	DHS	National	both	18-64	18-64	25093	38617	†
Colombia	2005	Encuesta Nacional de Situacion Nutricional	National	both		18-49		2783	*
Colombia	2007	Encuesta Nacional de Salud	National	both	18-69	18-69	5462	7686	*
Colombia	2010	DHS	National	both	18-64	18-64	39544	51623	†
Colombia	2010	STEPS	Subnational	both	18-64	18-64	922	1239	*
Colombia	2015	STEPS	Subnational	both	18-64	18-64	867	1142	*
Comoros	1996	DHS	National	both		20-49		744	†
Comoros	2011	STEPS	National	both	25-64	25-64	1541	3505	†
Comoros	2012	DHS	National	both		18-49		4105	†
Congo	1986	Enquête Brazzaville 1986	Community	urban		18-50		935	*
Congo	1987	Maire et al., Rev Epidemiol Sante Publique 40:252-58, 1992	Community	rural		18-45		750	*
Congo	1987	Enquête Nationale Congo 1987	National	rural		18-49		1264	*
Congo	1991	Enquête Brazzaville 1991	Community	urban	18-90	18-90	1200	1806	*
Congo	1996	Enquête Brazzaville 1996	Community	urban	18-90	18-90	1346	1818	*
Congo	2004	STEPS	Community	urban	25-64	25-64	1013	956	†
Congo	2005	DHS	National	both		18-49		5390	†
Congo	2011-2012	DHS	National	both		18-49		4415	†
Cook Islands	2003	STEPS	National	both	25-64	25-64	925	958	†
Cook Islands	2013-2015	STEPS	National	both	18-64	18-64	456	469	†
Costa Rica	2004	CAMDI	Community	urban	20+	20+	304	624	*
Costa Rica	2004-2006	Costa Rican Longevity and Healthy Aging Study Pre-1945 Cohort Wave 1	National	both	60+	60+	1163	1346	†
Costa Rica	2006-2008	Costa Rican Longevity and Healthy Aging Study Pre-1945 Cohort Wave 2	National	both	62+	62+	944	1102	†
Costa Rica	2008-2009	Encuesta Nacional de Nutrición 2008-2009	National	both		45+		661	*
Costa Rica	2009-2010	Costa Rican Longevity and Healthy Aging Study Pre-1945 Cohort Wave 3	National	both	64+	64+	737	887	†
Costa Rica	2010	Costa Rican National Cardiovascular Risk Factors Survey, 2010	National	both	20+	20+	778	1958	*
Costa Rica	2010-2011	Costa Rican Longevity and Healthy Aging Study 1945-1955 Cohort Wave 1	National	both	54-66	54-66	1058	1676	†
Costa Rica	2012-2014	Costa Rican Longevity and Healthy Aging Study 1945-1955 Cohort Wave 2	National	both	56-68	56-68	867	1470	†
Cote d'Ivoire	1994	DHS	National	both		20-49		2682	†
Cote d'Ivoire	1998-1999	DHS	National	both		18-49		2278	†
Cote d'Ivoire	2005	STEPS	Subnational	both	18-64	18-64	1834	2255	†
Cote d'Ivoire	2011-2012	DHS	National	both		18-49		4023	†
Croatia	1997-1999	Budak A et al., Lijec Vjesn 125(1-2):32-5, 2003	National	both	25-100	25-100	1763	2684	*
Croatia	2003-2004	School Health Survey	National	both	18-19	18-19	71	21	*
Croatia	2005	Endemic Nephropathy and Arterial hypertension (ENAH)	Subnational	rural	18+	18+	264	367	*
Croatia	2006-2008	The Cardiovascular risk factors in school age – intervention model development	National	both	18-20	18-20	190	202	*
Croatia	2008	Endemic Nephropathy and Arterial hypertension (ENAH)	Subnational	rural	18+	18+	331	527	*

Country	Data years	Survey/study name/citation	Level of representative-ness	Rural, urban or both	Age range as used for global analysis		Sample size as used for global analysis		Note
					Male	Female	Male	Female	
Croatia	2010	Endemic Nephropathy and Arterial hypertension (ENAH)	Subnational	rural	18+	18+	252	393	*
Croatia	2015	Endemic Nephropathy and Arterial hypertension (ENAH) Follow-up Study	Subnational	rural	18+	18+	224	460	*
Cuba	1999-2000	The Survey on Health, Well-Being, and Aging in Latin America and the Caribbean (SABE)	Community	urban	60+	60+	630	1044	2†
Cuba	2001-2002	National Survey of Risk Factors	National	both	20-60	20-60	11426	11426	*
Cuba	2010	National Risk Factor Survey	National	both	18+	18+	3200	3732	*
Cuba	2011	Non-communicable disease risk factor in Cienfuegos	Community	urban	18-80	18-80	594	849	*
Cyprus	1999-2000	Countrywide Integrated Noncommunicable Diseases Intervention (CINDI) Programme	National	both	25-65	25-65	457	546	*
Cyprus	2007-2008	Asthma Study Cyprus	National	both	18	18	12	22	*
Czech Republic	1985	Czech-MONICA	National	both	25-64	25-64	1243	1303	†
Czech Republic	1988	Czech-MONICA	National	both	25-64	25-64	1357	1408	†
Czech Republic	1992	Czech-MONICA	National	both	25-64	25-64	1131	1207	†
Czech Republic	1997-1998	Czech post-MONICA	National	both	25-64	25-64	1526	1665	*
Czech Republic	2000-2001	Czech post-MONICA	National	both	25-64	25-64	1629	1691	*
Czech Republic	2001	6th nationwide anthropological survey of children and adolescents 2001	National	both	18-19	18-20	1050	1794	*
Czech Republic	2002-2005	Health, Alcohol and Psychosocial factors In Eastern Europe (HAPIEE)	Subnational	urban	45-69	45-69	3247	3888	*
Czech Republic	2006-2009	Czech post-MONICA	National	both	25-64	25-64	1717	1858	*
Czech Republic	2015-2018	Czech post-MONICA	National	both	25-65	25-65	1241	1366	*
Denmark	1982	The Danish Conscription Database	National	both	18-26		1184		1*
Denmark	1983	The Danish Conscription Database	National	both	18-26		761		1*
Denmark	1984	The Danish Conscription Database	National	both	18-26		378		1*
Denmark	1984-1985	The Epidemiology of Gallstones in a 70 Year-Old Danish Population	Community	both	70	70	202	172	*
Denmark	1985	INTERSALT	Community	urban	20-59	20-59	99	100	†
Denmark	1986-1987	MONICA, Glostrup	Community	urban	29-61	29-61	746	753	†
Denmark	1987	Nilsson et al., J Intern Med 237:479-86, 1995	Community	urban	51		439		*
Denmark	1991-1992	MONICA, Glostrup	Community	urban	29-61	29-61	808	816	†
Denmark	1992-1994	Obesity Research Group-Copenhagen City Heart Study 3	Subnational	both	33-73		922		*
Denmark	1993-1997	EPIC Aarhus	Community	urban	50-65	50-65	8430	8717	*
Denmark	1993-1997	EPIC Copenhagen	Community	urban	50-65	50-65	18729	21133	*
Denmark	1996-1997	Drivsholm et al., Diabet Med 18:126-32, 2001	Subnational	urban	60	60	325	370	*
Denmark	2002-2003	Odense Androgen Study	Community	urban	20-29		783		*
Denmark	2006	Danish Conscript Register	National	both	18-26		24901		*
Denmark	2006-2008	The Health2006 Cohort	Community	urban	18-71	18-71	1553	1916	*
Denmark	2007	Danish Conscript Register	National	both	18-26		26999		*
Denmark	2007-2008	The Danish Health Examination Survey 2007-2008	National	both	18+	18+	7349	10651	*
Denmark	2008	Danish Conscript Register	National	both	18-26		24153		*
Denmark	2009	Danish Conscript Register	National	both	18-26		26663		*
Denmark	2009-2010	The European Youth Heart Study	Community	both	18-28	18-28	305	332	*
Denmark	2010	Danish Conscript Register	National	both	18-26		30522		*
Denmark	2011	Danish Conscript Register	National	both	18-26		30480		*
Denmark	2011-2012	The Health2006 cohort - 5-year follow-up	Community	urban	24-76	24-76	1057	1249	*
Denmark	2012	Danish Conscript Register	National	both	18-26		29438		*
Denmark	2012-2015	The Danish study of Functional Disorders (DanFunD)	Subnational	urban	18-72	18-72	3451	4034	*
Denmark	2013	Danish Conscript Register	National	both	18-26		30379		*
Denmark	2014	Danish Conscript Register	National	both	18-26		32206		*
Denmark	2015	Danish Conscript Register	National	both	18-26		28751		*
Dominica	2007	STEPS	National	both	18-64	18-64	417	526	†
Dominican Republic	1991	DHS	National	both		20-49		1965	†
Dominican Republic	1993	Aono et al., J Epidemiol 7(4):238-43, 1997	National	both	20-70	20-70	767	1149	*
Dominican Republic	1996	DHS	National	both		18-49		6431	†
Dominican Republic	1996-1998	Estudio factores de riesgo cardiovascular y sindrome metabolico en la Republica Dominicana I (EFRICARD I)	National	both	18-75	18-75	2087	4095	*
Dominican Republic	2010-2011	Estudio factores de riesgo cardiovascular y sindrome metabolico en la Republica Dominicana II (EFRICARD II)	National	both	18-75	18-75	1641	3254	*
Dominican Republic	2013	DHS	National	both	18-59	18-49	9281	7906	†
DR Congo	2001	Multiple Indicator Cluster Survey Round 2	National	both		18-49		5398	*
DR Congo	2005	STEPS	Subnational	urban	18+	18+	677	1031	†
DR Congo	2007	DHS	National	both		18-49		3612	†
DR Congo	2013-2014	DHS	National	both		18-49		7034	†
Ecuador	2004	Encuesta Demografica y de Salud Materno e Infantil/Reproductive Health Survey	National	both		18-49		3757	†
Ecuador	2004-2005	CArdiovascular Risk factors Multiple Evaluation in Latin America (CARMELA)	Community	urban	25-64	25-64	813	814	*
Ecuador	2009	National Survey of Health, Wellbeing, and Aging	National	both	60+	60+	2341	2592	2†

Country	Data years	Survey/study name/citation	Level of representative-ness	Rural, urban or both	Age range as used for global analysis		Sample size as used for global analysis		Note
					Male	Female	Male	Female	
Ecuador	2011-2013	Encuesta Nacional de Salud y Nutrición	National	both	18-59	18-59	13188	17272	*
Egypt	1992	DHS	National	both		20-49		4654	†
Egypt	1995	DHS	National	both		20-49		6499	†
Egypt	2000	DHS	National	both		20-49		13602	†
Egypt	2002	National Survey of Smoking, Obesity, Blood Pressure and Blood Glucose	National	both	18+	18+	2913	3643	*
Egypt	2003	DHS	National	both		20-49		7930	†
Egypt	2003-2004	Marzouk et al., Gut 56(8):1105-10, 2007	Community	rural	25+	25+	322	456	*
Egypt	2005	DHS	National	both		20-49		16864	†
Egypt	2005	STEPS	National	both	18-65	18-65	4271	4033	†
Egypt	2007-2009	Mostafa et al., Gut 59(8):1135-40, 2010	Community	rural	35+	35+	642	843	*
Egypt	2008	DHS	National	both	18-59	20-49	6480	15242	†
Egypt	2011	STEPS	National	both	18-65	18-65	1659	2844	†
Egypt	2014	DHS	National	both		20-49		18891	†
Egypt	2015	DHS	National	both	18-59	18-59	6459	7623	†
Egypt	2017	STEPS	National	both	18-69	18-69	2092	3615	†
El Salvador	2002-2003	Ecuesta Nacional de Salud Familiar	National	both		18-49		3748	†
El Salvador	2004	CAMDI	Community	urban	20+	20+	396	811	*
El Salvador	2008	Ecuesta Nacional de Salud Familiar	National	both		18-49		6282	†
Equatorial Guinea	2011	DHS	National	both		18-49		1074	*
Eritrea	1995	DHS	National	both		18-49		1621	*
Eritrea	2002	DHS	National	both		18-49		3223	*
Eritrea	2004	STEPS	National	both	18-64	18-64	1044	1027	†
Eritrea	2010	STEPS	National	both	25-74	25-74	1712	4285	†
Estonia	1984-1986	Abina et al., Blood Press 12:111-21, 2003	Community	urban	20-54	30-54	2477	851	*
Estonia	1992-1994	Abina et al., Blood Press 12:111-21, 2003	Community	urban	20-54	20-54	921	678	*
Estonia	1997	Pomerleau et al., Public Health Nutr 3:3-10, 2000	National	both	19-64	19-64	525	628	*
Estonia	1999-2001	Abina et al., Blood Press 12:111-21, 2003	Community	urban	20-54	20-54	635	692	*
Estonia	2002	Estonian Biobank	National	both	18+	18+	89	217	*
Estonia	2003	Estonian Biobank	National	both	18+	18+	2695	5688	*
Estonia	2003	The European Male Ageing Study	Community	both	40+		416		*
Estonia	2004	Estonian Biobank	National	both	18+	18+	281	496	*
Estonia	2007	Estonian Biobank	National	both	18+	18+	965	2129	*
Estonia	2008	Estonian Biobank	National	both	18+	18+	4991	10688	*
Estonia	2008	The European Male Ageing Study	Community	both	40+		305		*
Estonia	2009	Estonian Biobank	National	both	18+	18+	3752	6145	*
Estonia	2010	Estonian Biobank	National	both	18+	18+	3766	6504	*
Estonia	2011	Estonian Biobank	National	both	18+	18+	102	168	*
Estonia	2012	Estonian Biobank	National	both	18+	18+	82	126	*
Estonia	2013	Estonian Biobank	National	both	18+	18+	104	146	*
Estonia	2013-2015	National Dietary Survey (RTU) 2014	National	both	18-74	18-74	890	1768	*
Ethiopia	2000	DHS	National	both		18-49		11776	†
Ethiopia	2005	DHS	National	both		18-49		5247	†
Ethiopia	2006	STEPS	Subnational	urban	25-64	25-64	1642	2295	†
Ethiopia	2011	DHS	National	both	18-59	18-49	12624	12833	†
Ethiopia	2016	DHS	National	both	18-59	18-49	10912	12143	†
Fiji	2002	STEPS	National	both	18-64	18-64	2498	3605	†
Fiji	2005-2007	Pacific Obesity Prevention in Communities - Ma'alahi Youth Project	Subnational	urban	18-19	18-19	288	329	*
Fiji	2007-2008	Pacific Obesity Prevention in Communities - Ma'alahi Youth Project	Subnational	urban	18-22	18-22	398	458	*
Fiji	2009	Fiji Eye Health Survey 2009	National	both	40+	40+	582	776	*
Fiji	2011	STEPS	National	both	25-64	25-64	1123	1417	†
Finland	1983	Young Finns Study 1983	National	both	18-21	18-21	350	402	1*
Finland	1984-1989	Kuopio Ischaemic Heart Disease Risk factor Study	Subnational	both	42-61		2670		*
Finland	1985	INTERSALT, Turku	Community	urban	20-59	20-59	100	100	†
Finland	1985-1986	INTERSALT, Joensuu	Community	urban	20-59	20-59	100	100	†
Finland	1986	Young Finns Study 1986	National	both	18-24	18-24	467	567	*
Finland	1987	MONICA, North Karelia/Kuopio/Turku/Loimaa	Subnational	both	25-64	25-64	2896	3151	†
Finland	1989	Finnish cohort of the FINE study	Community	rural	70-89		446		*
Finland	1990-1992	Oulu 35 Study	Community	urban	56-57	56-57	231	326	*
Finland	1991-1993	Kuopio Ischaemic Heart Disease Risk factor Study	Subnational	both	46-64		1037		*

Country	Data years	Survey/study name/citation	Level of representative-ness	Rural, urban or both	Age range as used for global analysis		Sample size as used for global analysis		Note
					Male	Female	Male	Female	
Finland	1992	The National FINRISK Study	Subnational	both	25-64	25-64	2849	3201	*
Finland	1994	Finnish cohort of the FINE study	Community	rural	75-94		266		*
Finland	1996-1998	Oulu 35 Study	Community	urban	60-63	60-63	242	345	*
Finland	1996-1998	Savitaipale Study, Baseline	Community	rural	40-66	40-66	574	574	*
Finland	1997	The National FINRISK Study	National	both	25-74	25-74	4128	4131	*
Finland	1997	North Finland Birth Cohort 1966	Community	both	30-31	30-31	2770	149	*
Finland	1998-2001	Kuopio Ischaemic Heart Disease Risk factor Study	Subnational	both	53-73	53-73	854	914	*
Finland	2000	Finnish cohort of the FINE study	Community	rural	81-96		92		*
Finland	2000-2001	Health 2000 Survey	National	both	30+	30+	2591	3156	*
Finland	2001	Young Finns Study 2001	National	both	24-39	24-39	1004	1162	*
Finland	2001-2003	Oulu 45 Study	Community	urban	55-58	55-58	426	550	*
Finland	2001-2004	Helsinki Birth Cohort Study	Community	urban	56-69	56-69	927	1074	*
Finland	2002	The National FINRISK Study	National	both	25-74	25-74	3299	3826	*
Finland	2004-2005	FIN-D2D	Subnational	both	45-74	45-74	1364	1461	*
Finland	2005	Mantyselka et al., Rheumatology (Oxford) 47(8):1235-38, 2008	Community	rural	30-65	30-65	230	241	*
Finland	2005-2008	Kuopio Ischaemic Heart Disease Risk factor Study	Subnational	both	62-82	60-82	1241	630	*
Finland	2007	The National FINRISK Study	National	both	25-74	25-74	2934	3323	*
Finland	2007	Oulu 35 Study	Community	urban	71-73	71-73	182	271	*
Finland	2007	Young Finns Study 2007	National	both	30-45	30-45	976	1145	*
Finland	2007-2008	Savitaipale Study, Follow-up	Community	rural	51-75	51-75	430	483	*
Finland	2008	Control group for Finnish male former elite athletes	National	both	61+		206		*
Finland	2011	Young Finns Study 2011	National	both	34-49	34-49	870	1060	*
Finland	2011-2012	Health 2011 Survey	National	both	30+	30+	2021	2519	*
Finland	2012	The National FINRISK Study	National	both	25-74	25-74	2774	3052	*
Finland	2012	North Finland Birth Cohort 1966	Community	both	45-47	45-47	2541	3224	*
France	1985-1987	MONICA, Strasbourg	Subnational	both	35-64	35-64	664	713	†
France	1985-1987	MONICA, Strasbourg	Community	both	25-34	25-34	65	78	†
France	1985-1987	MONICA, Toulouse	Subnational	both	35-64	35-64	675	644	†
France	1986-1989	MONICA, Lille	Community	urban	25-64	25-64	878	732	†
France	1988-1991	MONICA, Toulouse	Subnational	both	35-64		586		†
France	1994-1996	MONICA, Toulouse	Subnational	both	35-64	35-64	608	566	†
France	1995-1997	MONICA, Lille	Community	urban	36-67	36-67	598	590	†
France	1995-1997	MONICA, Strasbourg	Subnational	both	35-64	35-64	526	523	†
France	1996-2003	Jaquet et al., Diabetologia 48(5):849-55, 2005	Community	urban	18-34	18-34	173	164	*
France	1999-2001	The Three City Study	Community	urban	65+	65+	2423	3778	*
France	2004-2006	National Monitoring of Arterial Risk in Lille (MONA LISA Lille)	Subnational	urban	35-75	35-75	783	795	*
France	2005-2007	National Monitoring of Arterial Risk in Bas-Rhin (MONA LISA Bas-Rhin)	Subnational	both	35-74	35-74	780	787	*
France	2005-2007	Monitoring national du risque artériel (MONA LISA Toulouse)	Subnational	both	35-74	35-74	829	796	*
France	2006-2007	Etude Nationale Nutrition Santé	National	both	18-74	18-74	876	1512	*
France	2006-2008	The Three City Study	Community	urban	72+	72+	768	1217	*
France	2011-2013	Enquête LIttorale Souffle Air Biologie EnvironnemenT (ELISABET) Dunkerque	Community	urban	40-64	40-64	761	812	2*
France	2011-2013	Enquête LIttorale Souffle Air Biologie EnvironnemenT (ELISABET) Lille	Community	urban	40-64	40-64	758	857	2*
French Polynesia	2010	STEPS	National	both	18-64	18-64	1458	1916	†
Gabon	2000	DHS	National	both		20-49		2082	†
Gabon	2009	STEPS	Subnational	urban	18-64	18-64	1009	1443	†
Gabon	2012	DHS	National	both		18-49		4365	†
Gambia	1996-1997	National Survey of Blindness and Low Vision	National	both	18+	18+	1733	2071	*
Gambia	2003	Siervo et al., Eur J Clin Nutr 60(4):455-63, 2006	Community	urban	18-50	18-50	50	50	*
Gambia	2010	STEPS	National	both	25-64	25-64	1610	1919	†
Gambia	2013	DHS	National	both		18-49		3605	†
Georgia	2010	STEPS	National	both	18-64	18-64	1842	4460	†
Georgia	2016	STEPS	National	both	18-69	18-69	1188	2784	†
Germany	1983-1987	MONICA, Rhein-Neckar Region	Community	urban	35-64	35-64	1489	1609	†
Germany	1984	The German Conscription Database	National	both	19		419719		1*
Germany	1984-1985	MONICA, Augsburg	Community	both	25-64	25-64	2005	1961	†
Germany	1984-1986	MONICA, Cottbus County	Community	urban	25-64	25-64	657	739	†
Germany	1985	The German Conscription Database	National	both	19		402487		*
Germany	1985-1986	INTERSALT, Cottbus	Community	urban	20-59	20-59	99	99	†
Germany	1985-1986	INTERSALT, Heidelberg	Community	urban	20-59	20-59	97	99	†

Country	Data years	Survey/study name/citation	Level of representative-ness	Rural, urban or both	Age range as used for global analysis		Sample size as used for global analysis		Note
					Male	Female	Male	Female	
Germany	1985-1986	CINDI	Subnational	both	25-64	25-64	1875	1990	*
Germany	1985-1986	INTERSALT, Bernried	Community	urban	20-59	20-59	99	98	†
Germany	1986	The German Conscription Database	National	both	19		382632		*
Germany	1987	The German Conscription Database	National	both	19		349083		*
Germany	1987-1988	MONICA, Erfurt	Community	urban	25-64	25-64	871	909	†
Germany	1988	The German Conscription Database	National	both	19		303265		*
Germany	1988	German Cardiovascular Prevention Study (GCP) - National Health Survey 1988	Subnational	both	25-69	25-69	2642	2678	*
Germany	1988	MONICA, Berlin-Lichtenberg	Community	urban	25-64	25-64	690	728	†
Germany	1988	MONICA, Bremen North/West	Community	urban	25-69	25-69	619	632	†
Germany	1988	MONICA, Bremen Center/South/East	Community	urban	25-69	25-69	499	582	†
Germany	1988	MONICA, Chemnitz	Community	urban	25-64	25-64	288	382	†
Germany	1988	MONICA, Zwickau	Community	urban	25-64	25-64	193	250	†
Germany	1988-1989	CINDI	Subnational	both	25-64	25-64	1361	1435	*
Germany	1988-1989	MONICA, Halle County	Subnational	urban	25-64	25-64	959	1201	†
Germany	1988-1989	MONICA, Rest of Karl-Marx-Stadt County	Subnational	urban	25-64	25-64	541	626	†
Germany	1989	The German Conscription Database	National	both	19		245740		*
Germany	1989-1990	MONICA, Cottbus County	Community	urban	25-64	25-64	539	529	†
Germany	1989-1990	MONICA, Augsburg	Community	both	25-64	25-64	1933	1944	†
Germany	1990	The German Conscription Database	National	both	19		206599		*
Germany	1990-1992	European Community Respiratory Health Survey, Hamburg	Community	urban	20-47	20-47	146	138	*
Germany	1990-1992	European Community Respiratory Health Survey, Erfurt	Community	urban	20-47	20-47	146	124	*
Germany	1991	The German Conscription Database	National	both	19		138195		*
Germany	1991-1992	MONICA, Bremen North/West	Community	urban	25-69	25-69	599	671	†
Germany	1991-1992	MONICA, Bremen Center/South/East	Community	urban	25-69	25-69	524	546	†
Germany	1991-1992	CINDI	Subnational	both	25-64	25-64	1326	1400	*
Germany	1991-1992	German Cardiovascular Prevention Study (GCP) - National Health Survey 1991	Subnational	both	25-69	25-69	2599	2670	*
Germany	1991-1992	First National Examination of life conditions, Environment and Health in East Germany 1991/92	Subnational	both	25-69	25-69	1042	1155	*
Germany	1991-1992	MONICA, Erfurt	Community	urban	25-64	25-64	587	572	†
Germany	1992	The German Conscription Database	National	both	19		220956		*
Germany	1993	The German Conscription Database	National	both	19		188655		*
Germany	1993-1994	MONICA, Chemnitz	Community	urban	25-64	25-64	408	424	†
Germany	1993-1994	MONICA, Zwickau	Community	urban	25-64	25-64	139	186	†
Germany	1994	The German Conscription Database	National	both	19		155426		*
Germany	1994-1995	MONICA, Augsburg	Community	both	25-64	25-64	1898	1968	†
Germany	1994-1998	EPIC Heidelberg	Community	urban	40-64	35-64	11680	13458	*
Germany	1994-1998	EPIC Potsdam	Community	urban	40-64	35-64	10224	15995	*
Germany	1995	The German Conscription Database	National	both	19		185762		*
Germany	1996	The German Conscription Database	National	both	19		191260		*
Germany	1997	The German Conscription Database	National	both	19		148738		*
Germany	1997-1999	German National Health Interview and Examination Survey (GNHIES98)	National	both	18-79	18-79	3435	3608	*
Germany	1997-2001	Study of Health in Pomerania (SHIP-0) baseline study	Subnational	both	20-80	20-80	2111	2187	*
Germany	1998	The German Conscription Database	National	both	19		146528		*
Germany	1999	The German Conscription Database	National	both	19		292732		*
Germany	1999-2001	KORA S4 Study: Kooperative Research in the Region of Augsburg Survey 4	Community	both	24-75	24-75	2076	2148	*
Germany	2000-2001	European Community Respiratory Health Survey, Hamburg	Community	urban	30-57	30-57	146	138	*
Germany	2000-2001	European Community Respiratory Health Survey, Erfurt	Community	urban	30-57	30-57	146	124	*
Germany	2000-2002	Epidemiological study of the chances of prevention, early recognition and optimal treatment of chronic diseases in an elderly	Subnational	both	50-75	50-75	4242	5091	*
Germany	2000-2003	Heinz Nixdorf RECALL Study	Community	urban	45-74	45-74	2375	2393	*
Germany	2002	Echinoccoccus Multilocularis and Internal Diseases in Leutkirch	Community	urban	18-65	18-65	1047	1128	*
Germany	2002-2006	Study of Health in Pomerania (SHIP-1) 5-year follow-up	Subnational	both	25-85	25-85	1583	1707	*
Germany	2006-2008	KORA F4 Study: Kooperative Research in the Region of Augsburg Follow-Up of Survey 4	Community	both	31-81	31-81	1480	1583	*
Germany	2008	The German Conscription Database	National	both	19		98926		*
Germany	2008-2011	Epidemiological study of the chances of prevention, early recognition and optimal treatment of chronic diseases in an elderly	Subnational	both	58-84	58-84	1449	1581	*
Germany	2008-2011	German Health Interview and Examination Survey for adults 2008-11 (DEGS1)	National	both	18-79	18-79	3389	3650	*
Germany	2008-2012	Study of Health in Pomerania, second cohort (SHIP-TREND)	Subnational	both	20-79	20-79	2099	2232	*
Germany	2009	The German Conscription Database	National	both	19		111455		*
Germany	2010	The German Conscription Database	National	both	19		101911		*
Ghana	1993	DHS	National	both		20-49		1650	†
Ghana	1997	Amoah et al., Ethn Dis 13(2 Suppl 2):S97-101, 2003	Community	both	25+	25+	1857	2875	*

Country	Data years	Survey/study name/citation	Level of representative-ness	Rural, urban or both	Age range as used for global analysis		Sample size as used for global analysis		Note
					Male	Female	Male	Female	
Ghana	1998	DHS	National	both		20-49		1979	†
Ghana	2001	Addo et al., Ethn Dis 16(4):894-99, 2006	Community	rural	18+	18+	89	206	*
Ghana	2001-2002	Cappuccio et al., Hypertension 43(5):1017-22, 2004	Community	both	35-84	35-84	194	338	*
Ghana	2002	Amoah et al., Ethn Dis 13(2 Suppl 2):S97-101, 2003	Community	both	25+	25+	1859	2947	*
Ghana	2003	Women's Health Study of Accra	Community	urban		18+		1184	*
Ghana	2003	DHS	National	both		18-49		4310	†
Ghana	2006	STEPS	Community	urban	25+	25+	841	1635	†
Ghana	2007-2008	SAGE	National	both	50+	50+	2192	1987	†
Ghana	2008	DHS	National	both		18-49		3882	†
Ghana	2008-2010	Women's Health Study of Accra	Community	urban		18+		2677	*
Ghana	2012-2014	Research on Obesity and Diabetes among African Migrants (RODAM), control group	Subnational	both	25+	25+	849	1710	*
Ghana	2014	DHS	National	both	18-59	18-49	3876	3933	†
Greece	1991	Seven Countries Study	Subnational	both	70-89		177		*
Greece	1991-1999	EPIC	National	both	19-86	19-86	11578	16477	*
Greece	1997	The Didima Study	Community	rural	18+	18+	265	373	*
Greece	2000-2001	Karalis et al., BMC Public Health 7:351, 2007	Community	rural	18+	18+	73	87	*
Greece	2003	National Epidemiological Survey	National	both	18-19	18-19	987	1150	*
Greece	2010-2012	ADONUT	National	both	18-19	18-19	1531	1641	*
Greece	2013-2015	Hellenic National Nutrition and Health Survey (HNNHS)	National	both	18+	18+	1514	2175	*
Greece	2016	SKG-Elderly	Community	urban	60+	60+	51	63	*
Greenland	2005-2010	Population Health Survey in Greenland	National	both	18+	18+	1336	1714	*
Grenada	2011	STEPS	National	both	25-64	25-64	438	637	†
Guatemala	1995	DHS	National	both		20-49		4547	†
Guatemala	1998-1999	DHS	National	both		20-49		2172	†
Guatemala	2001-2002	CAMDI	Community	urban	20+	20+	293	638	*
Guatemala	2002	Reproducive Health Survey	National	both	18-59	18-49	1922	6579	*
Guatemala	2003-2005	The Institute of Nutrition of Central America and Panama Nutrition Supplementation Trial Cohort	Community	both	25-41	25-41	249	267	*
Guatemala	2008-2009	Encuesta Nacional de Salud Materno Infantil	National	both	18-59	18-49	5807	13668	*
Guatemala	2014-2015	DHS	National	both		18-49		20785	†
Guinea	1999	DHS	National	both		20-49		2984	†
Guinea	2005	DHS	National	both		18-49		3091	†
Guinea	2009	STEPS	Subnational	both	18-64	18-64	1044	1119	†
Guinea	2012	DHS	National	both		18-49		3624	†
Guinea Bissau	2010	Multiple Indicator Cluster Survey	National	both		18-49		6653	*
Guyana	2009	DHS	National	both	18-49	18-49	2954	4009	†
Haiti	1994-1995	DHS	National	both		20-49		1788	†
Haiti	2000	DHS	National	both		18-49		7781	†
Haiti	2005-2006	DHS	National	both		18-49		4192	†
Haiti	2012	DHS	National	both		18-49		7565	†
Haiti	2016-2017	DHS	National	both		18-49		7668	†
Honduras	1996	Honduras National Micronutrient Survey	National	both		20-40		722	*
Honduras	2003-2004	CAMDI	Community	urban	20+	20+	428	764	*
Honduras	2005-2006	DHS	National	both		18-49		15463	†
Honduras	2011-2012	DHS	National	both		18-49		18078	†
Hungary	1985	INTERSALT	Community	rural	20-59	20-59	100	100	†
Hungary	1985-1988	First Hungarian Representative Nutrition Survey	National	both	18+	18+	3079	8916	*
Hungary	1987-1988	MONICA, Budapest	Community	urban	35-64	35-64	1413	1594	†
Hungary	1987-1988	MONICA, Pecs	Community	urban	35-64	35-64	1573	1510	†
Hungary	2003	The European Male Ageing Study	Community	both	40+		428		*
Hungary	2008	The European Male Ageing Study	Community	both	40+		349		*
Iceland	1985-1986	INTERSALT	Community	urban	20-59	20-59	100	100	†
Iceland	1985-1987	The Reykjavik Study (Men)	Subnational	urban	51-79		2584		*
Iceland	1987-1991	The Reykjavik Study (Women)	Subnational	urban		52-82		2993	*
Iceland	1988-1989	MONICA, Arnes County	Community	rural	25-64	25-64	385	435	†
Iceland	1988-1989	MONICA, Reykjavik	Subnational	urban	25-64	25-64	414	443	†
Iceland	1991-1994	The Reykjavik Study (Men)	Subnational	urban	70-86		797		*
Iceland	1993-1994	MONICA, Arnes County	Community	rural	25-64	25-64	422	484	†
Iceland	1993-1994	MONICA, Reykjavik	Subnational	urban	25-64	25-64	441	448	†
Iceland	1994-1996	The Reykjavik Study (Women)	Subnational	urban		69-88		1101	*

Country	Data years	Survey/study name/citation	Level of representative-ness	Rural, urban or both	Age range as used for global analysis		Sample size as used for global analysis		Note
					Male	Female	Male	Female	
Iceland	2001-2003	The Reykjavik Study for the young	Subnational	urban	47-62	47-62	626	705	*
Iceland	2002-2006	AGES-Reykjavik Study	Subnational	urban	66-96	66-96	2413	3272	*
Iceland	2005-2011	Risk Evaluation For INfarct Estimates (REFINE)	Subnational	urban	20-73	20-73	3402	3525	*
Iceland	2007-2011	AGES-Reykjavik Study - follow up visit	Subnational	urban	71-98	71-98	1389	1928	*
Iceland	2010-2012	Risk Evaluation For INfarct Estimates (REFINE) follow-up visit (REFINELO)	Subnational	urban	26-74	26-74	653	667	*
Iceland	2012-2013	Risk Evaluation For INfarct Estimates (REFINE) - follow-up visit (REFLOCT)	Subnational	urban	55-73	55-73	516	561	*
India	1986	INTERSALT	Community	urban	20-59	20-59	100	99	†
India	1988-1989	Ramachandran et al., Diabetes Res Clin Pract 58(1):55-60, 2002	Community	urban	20-74	20-74	455	437	*
India	1988-1990	NNMB survey	National	rural	20+	20+	4760	6607	*
India	1991-1994	Prabhakaran et al., Chronic Illn 3(1):8-19, 2007	Community	both	35-64	35-64	1930	2085	*
India	1991-1995	Reddy et al., Obes Rev 3(3):197-202, 2002	Community	both	35-64	35-64	2526	2926	*
India	1991-1997	Mumbai Cohort Study	Community	urban	35+	35+	88658	59515	*
India	1992-1994	Jaipur Heart Watch 1	Community	both	20-80	20-80	3331	1929	*
India	1993-1994	Khongsdier, Eur J Clin Nutr 56(6):484-89, 2002	Community	both	18-59		575		*
India	1995	Shobana et al., Diabetes Res Clin Pract 42(3):181-86, 1998	Community	urban	20-74	20-74	1061	1093	*
India	1995-1996	Kusuma et al., Ann Hum Biol 29(5):502-12, 2002	Community	both	18-84	18-84	747	737	*
India	1995-1996	Epidemiology of blood pressure across cross-cultural populations of Visakhapatnam district, Andhra Pradesh, India	Community	rural	19-76	19-76	209	228	*
India	1995-1997	Aravind Comprehensive Eye Survey	Community	rural	40+	40+	2308	2830	*
India	1995-1997	Kashmiri Adults	Subnational	both	40+	40+	2496	2587	*
India	1996	NNMB survey	National	rural	18+	18+	12747	18018	*
India	1996-1999	Chennai Urban Population Study	Community	urban	20+	20+	557	705	*
India	1997	Ramachandran et al., Diabetes Res Clin Pract 44(3):207-13, 1999	Community	rural	20-74	20-74	738	879	*
India	1998-1999	DHS	National	both		20-49		72536	†
India	1998-2001	Chennai Prospective Study	Community	urban	35+	35+	264848	235968	*
India	1999-2001	Jaipur Heart Watch 2	Community	urban	20-75	20-75	534	569	*
India	1999-2002	New Delhi Birth Cohort	Community	urban	26-33	26-33	886	638	*
India	2000	Ramachandran et al., Diabet Med 20(3):220-24, 2003	Subnational	urban	20-75	20-75	4640	5257	*
India	2001	NNMB survey	National	rural	18+	18+	11072	17317	*
India	2001-2004	Chennai Urban Rural Epidemiology Study	Community	urban	20+	20+	1094	1254	*
India	2002-2003	Jaipur Heart Watch 3	Community	urban	20-59	20-59	179	195	*
India	2002-2003	Blood Pressure epidemiology in tribal, rural and urban communities of Orissa with special reference to physical and social pa	Community	rural	18-80	18-80	200	186	*
India	2003-2005	India STEPS, Ballabgarh	Subnational	both	18-69	18-69	2486	2669	*
India	2003-2005	India STEPS, Chennai	Subnational	both	18-69	18-69	2515	2484	*
India	2003-2005	India STEPS, Delhi	Subnational	both	18-69	18-69	1163	1183	*
India	2003-2005	India STEPS, Dibrugarh	Subnational	both	18-69	18-69	2588	2531	*
India	2003-2005	India STEPS, Nagpur	Subnational	both	18-69	18-69	2394	2423	*
India	2003-2005	India STEPS, Trivandrum	Subnational	both	18-69	18-69	2286	2456	*
India	2004-2005	Jaipur Heart Watch 4	Community	urban	20-59	20-59	413	473	*
India	2005-2006	DHS	National	both	18-54	18-49	64044	102354	†
India	2005-2006	NNMB survey	National	rural	18+	18+	14036	18598	*
India	2005-2007	Prevalence of cardiovascular risk factors in rural Tamil Nadu	Community	rural	25-65	25-65	4927	5573	*
India	2005-2011	Bengali School Children	Community	urban	18-21	18-21	527	577	*
India	2006	Ramachandran et al., Diabetes Care 31(5):893-98, 2008	Community	both	20+	20+	3321	3745	*
India	2006-2007	Kusuma et al., Asia Pac J Public Health 21(4):497-507, 2009	Community	urban	18-74	18-74	182	192	*
India	2006-2008	Central India Eye and Medical Study	Community	rural	30+	30+	2190	2518	*
India	2006-2008	Kashmiri Young Adults	Subnational	both	20-40	20-40	2119	905	*
India	2006-2009	New Delhi Birth Cohort	Community	urban	33-38	33-38	650	445	*
India	2007-2008	Integrated Disease Surveillance Project Non-communicable Disease Risk Factors Survey, Andhra Pradesh	Subnational	both	18-64	18-64	2602	3312	*
India	2007-2008	Integrated Disease Surveillance Project Non-communicable Disease Risk Factors Survey, Kerala	Subnational	both	18-64	18-64	1615	3000	*
India	2007-2008	Integrated Disease Surveillance Project Non-communicable Disease Risk Factors Survey, Madhya Pradesh	Subnational	both	18-64	18-64	2651	2733	*
India	2007-2008	Integrated Disease Surveillance Project Non-communicable Disease Risk Factors Survey, Maharashtra	Subnational	both	18-64	18-64	2899	2823	*
India	2007-2008	Integrated Disease Surveillance Project Non-communicable Disease Risk Factors Survey, Mizoram	Subnational	both	18-64	18-64	2137	1990	*
India	2007-2008	Integrated Disease Surveillance Project Non-communicable Disease Risk Factors Survey, Tamil Nadu	Subnational	both	18-64	18-64	1969	2853	*
India	2007-2008	Integrated Disease Surveillance Project Non-communicable Disease Risk Factors Survey, Uttrakhand	Subnational	both	18-64	18-64	1954	2959	*
India	2007-2008	SAGE	National	both	50+	50+	3213	3147	†
India	2007-2009	Prevalence of NCD risk factor in people above 15 year in Rural area Nagpur using WHO STEP approach	Community	rural	18+	18+	1833	1685	*
India	2008-2010	ICMR India Diabetes Study	National	both	20+	20+	6953	6889	*
India	2009-2010	Baseline Survey for the assessment of prevalence of risk factors of NCDs in Gandhinagar District	Community	both	18-64	18-64	1440	1421	*
India	2011-2012	NNMB survey	National	rural	18+	18+	24079	32208	*

Country	Data years	Survey/study name/citation	Level of representative-ness	Rural, urban or both	Age range as used for global analysis		Sample size as used for global analysis		Note
					Male	Female	Male	Female	
India	2012-2013	District Level Household and Facility Survey (DLHS) 4	National	both	18+	18+	401424	472287	†
India	2014	Annual Health Survey: Clinical, Anthropometric and Bio-chemical	National	both	18+	18+	440705	479413	†
India	2015-2016	DHS	National	both	18-54	18-49	97581	582772	†
Indonesia	1983-1987	Strickland et al., Eur J Clin Nutr 48 Suppl 3: S98-108; discussion S-9, 1994	Community	both	18+	18+	447	564	*
Indonesia	1993-1994	Indonesian Family Life Surveys	National	both	18+	18+	5738	7057	†
Indonesia	1997-1998	Indonesian Family Life Surveys	National	both	18+	18+	7711	9480	†
Indonesia	2000-2001	Indonesian Family Life Surveys	National	both	18+	18+	10569	11438	†
Indonesia	2001	Ng et al., Bull World Health Organ 84(4):305-13, 2006	Community	both	18-74	18-74	1261	1234	*
Indonesia	2001	STEPS/SURKESNAS	National	both	18-64	18-64	4100	4775	*
Indonesia	2003	A genetic-ecological study of the risk foctors for lifestyle-related diseases in Oceanian populations, Study A	Community	rural	18-79	18-79	99	103	*
Indonesia	2003	A genetic-ecological study of the risk foctors for lifestyle-related diseases in Oceanian populations, Study B	Community	rural	18-79	18-79	100	140	*
Indonesia	2006	Jakarta Non Communicable Disease Risk Factor Surveillance	Community	urban	25-64	25-64	641	950	*
Indonesia	2007-2008	Indonesian Family Life Surveys	National	both	18+	18+	12715	14219	†
Indonesia	2013	Population Health Basic Health Research 2013 (Riskesdas 2013): ages 5-19 years	National	both	18-19	18-19	15350	14095	*
Indonesia	2013	Population Health Basic Health Research 2013 (Riskesdas 2013): ages 20 years and older	National	both	20+	20+	299439	328085	*
Indonesia	2014-2015	Indonesian Family Life Surveys	National	both	18+	18+	14126	15587	†
Iran	1990-1991	National Health Survey I	National	both	18	18	284	405	*
Iran	1997-1998	Khadivzadeh, East Mediterr Health J 8(4-5):612-18, 2002	Community	urban		18-49		1513	*
Iran	1999-2000	National Health Survey II	National	both	18+	18+	13702	16618	*
Iran	1999-2001	Tehran Lipid and Glucose Study	Community	urban	18+	18+	4555	6126	*
Iran	2000	ASADABADI Study	Community	urban	18+	18+	132	168	*
Iran	2001	Isfahan Healthy Heart Program, Arak	Community	both	19+	19+	3107	3204	*
Iran	2001	Isfahan Healthy Heart Program, Isfahan	Community	both	19+	19+	1992	2145	*
Iran	2001	Isfahan Healthy Heart Program, Najaf Abad	Community	both	19+	19+	978	987	*
Iran	2001	Isfahan Healthy Heart Program in Students, Arak	Community	both	18	18	19	6	*
Iran	2001	Isfahan Healthy Heart Program in Students, Isfahan	Community	both	18		7		*
Iran	2002-2005	Tehran Lipid and Glucose Study	Community	urban	18+	18+	2153	2766	*
Iran	2003-2004	Childhood and Adolescence Surveillance and Prevention of Adult Noncommunicable Disease (CASPIAN)	National	both	18	18	370	362	*
Iran	2003-2004	The Persian Gulf Healthy Heart Study	Subnational	urban	25-75	25-75	1736	1973	*
Iran	2004	Hajian-Tilaki et al., Obes Rev 8(1):3-10, 2007	Community	urban	20-70	20-70	1800	1800	*
Iran	2004-2008	Golestan Cohort Study Main Phase	Subnational	both	40-75	40-75	21229	28808	*
Iran	2005	Dastgiri et al., J Public Health Nutr 9: 996-1000, 2006	Subnational	urban	18-70	18-70	116	151	*
Iran	2005	STEPS	National	both	18-64	18-64	37998	37520	*
Iran	2005-2006	Rashidy-Pour, Obes Rev 10(1):2-6, 2009	Subnational	both	30-70	30-70	1695	2104	*
Iran	2005-2008	Tehran Lipid and Glucose Study	Community	urban	18+	18+	2531	3259	*
Iran	2006	STEPS	National	both	18-65	18-65	14130	14008	*
Iran	2007	Isfahan Healthy Heart Program, Arak	Community	both	19+	19+	2452	2383	*
Iran	2007	Isfahan Healthy Heart Program, Isfahan	Community	both	19+	19+	1464	1453	*
Iran	2007	Isfahan Healthy Heart Program, Najaf Abad	Community	both	19+	19+	748	795	*
Iran	2007	Isfahan Healthy Heart Program in Students, Arak	Community	both	18	18	26	8	*
Iran	2007	Isfahan Healthy Heart Program in Students, Isfahan	Community	both	18	18	9	6	*
Iran	2007	Isfahan Healthy Heart Program in Students, Najaf Abad	Community	both	18		3		*
Iran	2007	STEPS	National	both	18-64	18-64	2205	2193	*
Iran	2007	STEPS	National	both	18-64	18-64	13839	13805	*
Iran	2008	STEPS	National	both	18-64	18-64	13691	13601	*
Iran	2008-2009	Zahedan city study	Community	urban	18+	18+	1131	1072	*
Iran	2008-2010	Amol county study	Community	both	18+	18+	3207	2499	*
Iran	2008-2010	Tehran city study	Community	urban	18+	18+	392	500	*
Iran	2008-2011	Tehran Lipid and Glucose Study	Community	urban	20+	20+	4622	5884	*
Iran	2009	STEPS	National	both	18-64	18-64	13919	13716	*
Iran	2009-2010	Childhood and Adolescence Surveillance and Prevention of Adult Noncommunicable Disease (CASPIAN)	National	both	18	18	497	524	*
Iran	2009-2010	The Persian Gulf Healthy Heart Study	Subnational	urban	31-79	31-79	834	1016	*
Iran	2010-2011	The Yazd Eye Study	Subnational	both	40-80	40-80	875	1012	*
Iran	2010-2012	Golestan Cohort Study Second Phase	Subnational	both	43-82	43-82	5416	5980	*
Iran	2011	STEPS	National	both	18-69	18-69	4075	5738	*
Iran	2011-2012	Childhood and Adolescence Surveillance and Prevention of Adult Noncommunicable Disease (CASPIAN)	National	both	18	18	287	269	*
Iran	2012	National Integrated Micronutrient Survey (NIMS) 2012	National	both	18-60	18-60	6200	6518	*
Iran	2012-2014	Pars Cohort Study	Community	rural	40-90	40-90	4272	4987	*
Iran	2013-2014	Bushehr Elderly Health Program (BEH)	Community	urban	60+	60+	1437	1514	*

Country	Data years	Survey/study name/citation	Level of representative-ness	Rural, urban or both	Age range as used for global analysis		Sample size as used for global analysis		Note
					Male	Female	Male	Female	
Iran	2013-2014	Gilan Eye Study	Subnational	both	50+	50+	1059	1439	*
Iran	2013-2014	Isfahan Salt Study (ISS)	Community	urban	18-60	18-60	700	734	*
Iran	2014-2015	Childhood and Adolescence Surveillance and Prevention of Adult Noncommunicable Disease (CASPIAN)	National	both	18	18	329	386	*
Iran	2015	Iranian School Measurement Database	National	both	18	18	969	1815	*
Iran	2016	STEPS	National	both	18+	18+	14080	15036	*
Iraq	2006	STEPS	National	both	25-64	25-64	2251	2252	*
Iraq	2015	STEPS	National	both	18+	18+	1589	2312	†
Ireland	1997-1999	North/South Ireland Food Consumption Survey	National	both	18-64	18-64	613	698	*
Ireland	1998	Survey of Lifestyle, Attitudes and Nutritional in Ireland 1998	National	both	18+	18+	123	296	†
Ireland	2002	Survey of Lifestyle, Attitudes and Nutritional in Ireland 2002	National	both	18+	18+	164	216	†
Ireland	2006-2007	Survey of Lifestyle, Attitudes and Nutritional in Ireland 2006-2007	National	both	18+	18+	945	1225	†
Ireland	2008-2010	National Adult Nutrition Survey	National	both	18+	18+	657	695	*
Ireland	2009-2011	The Irish Longitudinal Study on Ageing	National	both	50+	50+	2690	3168	†
Israel	1985-1986	MONICA, Tel Aviv	Community	urban	35-64	35-64	653	685	†
Israel	1990-1991	The Jerusalem Longitudinal Cohort Study	Community	urban	69-70	69-70	245	199	*
Israel	1997-1998	The Jerusalem Longitudinal Cohort Study	Community	urban	76-77	76-77	422	429	*
Israel	1999-2001	Mabat First Israeli National Health and Nutrition Survey	National	both	25-64	25-64	1367	1404	*
Israel	1999-2005	The Israel Glucose Intolerance, Obesity and Hypertension Study	National	urban	58+	58+	514	527	*
Israel	2002-2007	Hadera District Study	Subnational	urban	25-78	25-78	548	538	*
Israel	2003-2004	Mabat Youth First Israeli National Health and Nutrition Survey in 7th-12th grade students	National	both	18	18	50	33	*
Israel	2005-2006	The Jerusalem Longitudinal Cohort Study	Community	urban	83-85	83-85	490	584	*
Israel	2005-2006	Mabat Zahav First National Health and Nutrition Survey in ages 65 and over	National	urban	65+	65+	743	819	*
Israel	2014-2015	Mabat Second Israeli National Health and Nutrition Survey	National	both	18-64	18-64	1061	1073	*
Israel	2014-2015	Mabat Zahav Second National Health and Nutrition Survey ages in 65 and over	National	both	65+	65+	307	318	*
Israel	2015-2016	Mabat Youth Second Israeli National Health and Nutrition Survey in 7th-12th grade students	National	both	18	18	46	33	*
Italy	1983-1996	Malattie cardiovascolari ATerosclerotiche Istituto Superiore di Sanità	Community	rural	18-77	18-77	3948	4488	*
Italy	1985	Finland, Italy, Netherlands, Elderly (Fine-Italy)	Community	rural	65-84		650		*
Italy	1985	INTERSALT, Naples	Community	urban	20-59	20-59	100	100	†
Italy	1985-1988	Pisa Epidemiological Study - first survey	Community	urban	18-90	18-90	1502	1755	*
Italy	1986	INTERSALT, Bassiano	Community	urban	20-59	20-59	99	100	†
Italy	1986	INTERSALT, Gubbio	Community	urban	20-59	20-59	99	100	†
Italy	1986	INTERSALT, Mirano	Community	urban	20-59	20-59	100	100	†
Italy	1986	MONICA, Friuli	Subnational	urban	25-64	25-64	921	918	†
Italy	1986-1987	MONICA, Brianza	Subnational	urban	25-64	25-64	814	832	†
Italy	1988-1991	Po River Delta Epidemiological Study - second survey	Community	rural	18-73	18-73	1142	1307	*
Italy	1989	MONICA, Friuli	Subnational	urban	25-64	25-64	902	900	†
Italy	1989	Ventimiglia Heart Study	Community	rural	18+	18+	486	594	*
Italy	1989-1990	MONICA, Brianza	Subnational	urban	25-64	25-64	787	786	†
Italy	1990	Bruneck Study	Community	rural	40-79	40-79	469	450	*
Italy	1991-1993	Pisa Epidemiological Study - second survey	Community	urban	18-97	18-97	1164	1440	*
Italy	1992-1993	Italian Longitudinal Study on Aging	National	both	65-84	65-84	1666	1455	*
Italy	1992-1998	Vobarno Study	Community	both	25-64	35-64	265	309	*
Italy	1993-1994	MONICA, Brianza	Subnational	urban	25-64	25-64	801	856	†
Italy	1993-1998	EPIC Florence	Community	urban	24-72	24-72	3498	9968	*
Italy	1994	MONICA, Friuli	Subnational	urban	25-64	25-64	882	888	†
Italy	1995	Bruneck Study	Community	rural	45-84	45-84	411	408	*
Italy	1995-1996	Italian Longitudinal Study on Aging	National	both	68-90	68-90	1011	808	*
Italy	1995-1999	PROgetto Veneto Anziani (PROVA)	Subnational	both	65+	65+	1187	1722	*
Italy	1997-1999	Lucca CUORE Study	Community	urban	18-84	18-84	897	1123	*
Italy	1998-1999	progetto VIP	Community	both	25-74	25-74	599	600	*
Italy	1998-2000	InCHIANTI study	Community	both	18+	18+	560	681	*
Italy	1998-2002	Osservatorio Epidemiologico Cardiovascolare	National	both	35-74	35-74	4870	4752	*
Italy	2000	Bruneck Study	Community	rural	50-89	50-89	331	361	*
Italy	2000-2001	Italian Longitudinal Study on Aging	National	both	73-93	73-93	557	473	*
Italy	2000-2003	PROgetto Veneto Anziani (PROVA)	Subnational	both	67+	67+	795	1331	*
Italy	2001-2003	The Study of Asti	Community	both	45-64	45-64	780	878	*
Italy	2002-2005	PROgetto Veneto Anziani (PROVA)	Subnational	both	68+	68+	621	1138	*
Italy	2003	The European Male Ageing Study	Community	both	40+		433		*
Italy	2004-2005	Italian Project on the Epidemiology of Alzheimer's disease	National	both	65-84	65-84	1569	1421	*

Country	Data years	Survey/study name/citation	Level of representative-ness	Rural, urban or both	Age range as used for global analysis		Sample size as used for global analysis		Note
					Male	Female	Male	Female	
Italy	2004-2005	Vobarno study	Community	rural	55-74	55-74	99	113	*
Italy	2004-2008	Cardiolab project	National	urban	40+	40+	19152	14782	*
Italy	2005	Bruneck Study	Community	rural	55-93	55-93	264	307	*
Italy	2005-2007	Moli-family Study	Subnational	both	18-35	18-35	98	124	*
Italy	2005-2010	Moli-sani Study	Subnational	both	35+	35+	11694	12614	*
Italy	2008	The European Male Ageing Study	Community	both	40+		346		*
Italy	2008-2009	progetto VIP	Community	both	25-74	25-74	596	596	*
Italy	2008-2012	Osservatorio Epidemiologico Cardiovascolare/Health Examination Survey	National	both	35-80	35-80	4367	4332	*
Italy	2009-2010	Grosso et al., J Epidemiol 24(4):327-33, 2014	Community	both	19+	19+	760	1129	*
Italy	2009-2011	Pisa Epidemiological Study - third survey	Community	urban	18+	18+	482	556	*
Italy	2010	Bruneck Study	Community	rural	60-98	60-98	225	259	*
Italy	2010-2012	CArdiovascular risk MEtabolic syndrome LIver and Autoimmunity diseases (CA.ME.LI.A)	Community	both	18-75	18-75	477	515	*
Italy	2011-2012	Vobarno study	Community	rural	49-62	49-62	107	143	*
Italy	2014-2016	Mediterranean healthy Eating, Aging and Lifestyles (MEAL) study	Subnational	urban	20+	20+	762	762	*
Italy	2015	Bruneck Study	Community	rural	65-98	65-98	171	169	*
Jamaica	1993	Zohoori et al., West Indian Med J 52(2):111-17, 2003	Community	urban	25-74	25-74	845	1245	*
Jamaica	1994-1995	Cooper et al., Am J Public Health 87(2):160-68, 1997	Community	urban	25-100	25-100	597	833	*
Jamaica	1998	Ragoobirsingh et al., Diabetes Obes Metab 6(1):23-27, 2004	National	both	18+	18+	552	945	*
Jamaica	2000-2001	Jamaica Health and Lifestyle Survey	National	both	18-74	18-74	607	1227	*
Jamaica	2006-2007	Jamaica Youth Risk and Resiliency Behaviour Survey 2006	National	both	18-19	18-19	150	188	*
Jamaica	2007-2008	Jamaica Health and Lifestyle Survey	National	both	18-74	18-74	821	1822	*
Jamaica	2012	Older Persons in Jamaica 2012	National	both	60+	60+	157	203	11*
Japan	1982	National Nutrition Survey	National	both	18+	18+	4755	6709	1*
Japan	1983	National Nutrition Survey	National	both	18+	18+	4579	6548	1*
Japan	1984	National Nutrition Survey	National	both	18+	18+	4537	6286	1*
Japan	1985	INTERSALT, Osaka	Community	urban	20-59	20-59	100	97	†
Japan	1985	INTERSALT, Tochigi	Community	urban	20-59	20-59	95	99	†
Japan	1985	INTERSALT, Toyama	Community	urban	20-59	20-59	100	100	†
Japan	1985	National Nutrition Survey	National	both	18+	18+	5383	6869	*
Japan	1985-1986	Akabane Study	Community	urban	40-69	40-69	812	1022	*
Japan	1986	National Nutrition Survey	National	both	18+	18+	5275	6741	*
Japan	1987	Konan Town Study	Community	rural	20-79	20-79	70	88	*
Japan	1987	National Nutrition Survey	National	both	18+	18+	4721	6486	*
Japan	1988	Konan Town Study	Community	rural	20-79	20-79	76	85	*
Japan	1988	National Nutrition Survey	National	both	18+	18+	5126	6397	*
Japan	1989	Aito Town Study	Community	rural	18-74	18-84	529	525	*
Japan	1989	Konan Town Study	Community	rural	20-79	20-79	58	63	*
Japan	1989	National Nutrition Survey	National	both	18+	18+	4325	5571	*
Japan	1990	Konan Town Study	Community	rural	20-79	20-79	27	51	*
Japan	1990	National Nutrition Survey	National	both	18+	18+	4583	5928	*
Japan	1990-1994	Japan Public Health Center-based prospective Study (JPHC Study), Cohort I	Subnational	both	40-59	40-59	8749	14481	*
Japan	1991	Konan Town Study	Community	rural	20-79	20-79	93	116	*
Japan	1991	Shigaraki Town Study	Community	rural	30-89	30-89	230	319	*
Japan	1991	National Nutrition Survey	National	both	18+	18+	4667	5860	*
Japan	1992	Konan Town Study	Community	rural	20-79	20-79	45	47	*
Japan	1992	Shigaraki Town Study	Community	rural	30-89	30-89	288	385	*
Japan	1992	National Nutrition Survey	National	both	18+	18+	4348	5458	*
Japan	1993	Konan Town Study	Community	rural	20-79	20-79	54	65	*
Japan	1993	Shigaraki Town Study	Community	rural	30-89	30-89	301	452	*
Japan	1993	National Nutrition Survey	National	both	18+	18+	4414	5519	*
Japan	1993-1994	Japan Public Health Center-based prospective Study (JPHC Study), Cohort II	Subnational	both	40-69	40-69	8534	16190	*
Japan	1994	Japanese Population-Based Osteoporosis Study	Subnational	both		18-79		3222	*
Japan	1994	Konan Town Study	Community	rural	20-79	20-79	43	59	*
Japan	1994	Shigaraki Town Study	Community	rural	30-89	30-89	251	336	*
Japan	1994	National Nutrition Survey	National	both	18+	18+	4303	5318	*
Japan	1995	Konan Town Study	Community	rural	20-79	20-79	45	61	*
Japan	1995	Shigaraki Town Study	Community	rural	30-89	30-89	300	470	*
Japan	1995	National Nutrition Survey	National	both	18+	18+	4326	5286	*
Japan	1996	Shigaraki Town Study	Community	rural	30-89	30-89	86	152	*

Country	Data years	Survey/study name/citation	Level of representative-ness	Rural, urban or both	Age range as used for global analysis		Sample size as used for global analysis		Note
					Male	Female	Male	Female	
Japan	1996	National Nutrition Survey	National	both	18+	18+	4303	5230	*
Japan	1996-1997	INTERMAP, AitoTown	Community	rural	40-59	40-59	130	129	†
Japan	1997	Shigaraki Town Study	Community	rural	30-89	30-89	61	100	*
Japan	1997	National Nutrition Survey	National	both	18+	18+	4173	5161	*
Japan	1997-1998	INTERMAP, Sapporo	Community	urban	40-59	40-59	149	148	†
Japan	1997-1998	INTERMAP, Toyama	Community	urban	40-59	40-59	149	150	†
Japan	1997-1998	INTERMAP, Wakayama	Community	urban	40-59	40-59	146	144	†
Japan	1997-2000	Sudo et al., J Orthop Sci 13(5):413-18, 2008	Community	rural	55+	45+	261	785	*
Japan	1998	Niigata Study	Community	urban	70	70	287	284	*
Japan	1998	National Nutrition Survey	National	both	18+	18+	4407	5327	*
Japan	1999	Niigata Study	Community	urban	71	71	245	216	*
Japan	1999	National Nutrition Survey	National	both	18+	18+	3610	4635	*
Japan	2000	Niigata Study	Community	urban	72	72	233	202	*
Japan	2000	National Nutrition Survey	National	both	18+	18+	3863	4612	*
Japan	2001	The Japan Association of Health Service Database	Subnational	both	20+	20+	1471868	1231378	*
Japan	2001	Niigata Study	Community	urban	73	73	235	201	*
Japan	2001	National Nutrition Survey	National	both	18+	18+	3708	4675	*
Japan	2002	Niigata Study	Community	urban	74	74	228	202	*
Japan	2002	National Nutrition Survey	National	both	18+	18+	3472	4279	*
Japan	2002-2003	The Hisayama Study	Community	rural	40+	40+	1414	1884	*
Japan	2003	National Health and Nutrition Survey	National	both	18+	18+	3432	4307	*
Japan	2003	Niigata Study	Community	urban	75	75	215	189	*
Japan	2004	National Health and Nutrition Survey	National	both	18+	18+	2816	3467	*
Japan	2004	Niigata Study	Community	urban	76	76	215	185	*
Japan	2005	National Health and Nutrition Survey	National	both	18+	18+	2592	3207	*
Japan	2005	Niigata Study	Community	urban	77	77	203	184	*
Japan	2006	National Health and Nutrition Survey	National	both	18+	18+	2964	3641	*
Japan	2006	Niigata Study	Community	urban	78	78	199	194	*
Japan	2007	National Health and Nutrition Survey	National	both	18+	18+	2990	3606	*
Japan	2007	Niigata Study	Community	urban	79	79	183	192	*
Japan	2008	Resident in Kanazawa City (age 40+)	Community	urban	40+	40+	6562	11944	*
Japan	2008	National Health and Nutrition Survey	National	both	18+	18+	3058	3739	*
Japan	2008	Niigata Study	Community	urban	80	80	174	180	*
Japan	2009	National Health and Nutrition Survey	National	both	18+	18+	2978	3685	*
Japan	2010	National Health and Nutrition Survey	National	both	18+	18+	2766	3406	*
Japan	2011	National Health and Nutrition Survey	National	both	18+	18+	2620	3178	*
Japan	2011	The Tokyo Health Service Association Database	Community	urban	20+	20+	82453	54028	*
Japan	2012	National Health and Nutrition Survey	National	both	18+	18+	9817	12182	*
Japan	2013	National Health and Nutrition Survey	National	both	18+	18+	2774	3250	*
Japan	2014	National Health and Nutrition Survey	National	both	18+	18+	2802	3268	*
Japan	2014-2015	Nagaoka Health Screening	Community	both	20-89	20-89	4938	4298	*
Japan	2015	National Health and Nutrition Survey	National	both	18+	18+	2518	3071	*
Jordan	1994-1996	Ajlouni et al., Int J Obes Relat Metab Disord 22(7):624-28, 1998	Subnational	both	25+	25+	1047	1787	*
Jordan	1997	DHS	National	both		20-49		3002	†
Jordan	2002	DHS	National	both		20-49		4839	†
Jordan	2004	Khader et al., Metab Syndr Relat Disord 6(2):113-20, 2008	Community	both	25+	25-59	394	548	*
Jordan	2004	Behavioural Risk Factor Surveillence Survey	National	both	18+	18+	236	472	*
Jordan	2007	Behavioural Risk Factor Surveillence Survey	National	both	18+	18+	330	433	*
Jordan	2007	DHS	National	both		20-49		4451	†
Jordan	2009	DHS	National	both		20-49		4054	†
Jordan	2009	Metablic abnomalities and vitamin D study	National	both	18+	18+	1144	3350	*
Jordan	2012	DHS	National	both		20-49		6357	†
Jordan	2016-2017	National Cardiovascular Diseases and Diabetes Study (NCDDS)	National	both	18+	18+	1187	2745	*
Kazakhstan	1985	Balakhmetova et al., Ter Arkh 63(1):17-20, 1991	Community	urban	20-54		2886		*
Kazakhstan	1995	DHS	National	both		18-49		3138	†
Kazakhstan	1999	DHS	National	both		18-49		2005	†
Kazakhstan	2015	Almaty STEPS	Subnational	both	18-69	18-69	383	1144	*
Kazakhstan	2015	Shymkent STEPS	Subnational	both	18-69	18-69	400	808	*
Kazakhstan	2015-2016	Aktobe STEPS	Subnational	both	18-69	18-69	348	1153	*

Country	Data years	Survey/study name/citation	Level of representative-ness	Rural, urban or both	Age range as used for global analysis		Sample size as used for global analysis		Note
					Male	Female	Male	Female	
Kenya	1985	INTERSALT	Community	rural	20-59	20-59	90	86	†
Kenya	1993	DHS	National	both		20-49		3113	†
Kenya	1998	DHS	National	both		20-49		3009	†
Kenya	2003	DHS	National	both		18-49		6227	†
Kenya	2008-2009	DHS	National	both		18-49		6795	†
Kenya	2014	DHS	National	both		18-49		11772	†
Kenya	2015	STEPS	National	both	18-69	18-69	1751	2514	†
Kiribati	2004	STEPS	National	both	18-64	18-64	729	895	†
Kiribati	2015-2016	STEPS	National	both	18-69	18-69	557	694	†
Kuwait	1993-1994	al-Isa, Ann Nutr Metab 41(5):307-14, 1997	Community	both	18+		1730		*
Kuwait	1995-1996	Abdella et al., Diabetes Res and Clin Pract 42(3):187-196, 1998	Subnational	both	20-84	20-84	1099	1892	*
Kuwait	1998	Abiaka et al., Biol Trace Elem Res 91(1):33-43, 2003	National	both	18-80	18-80	178	233	*
Kuwait	2001	Kuwait nutrition surveillance system	National	both	18	18	124	132	*
Kuwait	2002	Kuwait nutrition surveillance system	National	both	18	18	97	93	*
Kuwait	2003	Kuwait nutrition surveillance system	National	both	18	18	196	175	*
Kuwait	2004	Kuwait nutrition surveillance system	National	both	18	18	124	112	*
Kuwait	2005	Kuwait nutrition surveillance system	National	both	18	18	170	179	*
Kuwait	2006	Kuwait nutrition surveillance system	National	both	18	18	134	152	*
Kuwait	2006	STEPS	National	both	20-65	20-65	918	1298	†
Kuwait	2007	Kuwait nutrition surveillance system	National	both	18	18	138	117	*
Kuwait	2008	Kuwait nutrition surveillance system	National	both	18	18	84	107	*
Kuwait	2008-2009	National Nutrition Program for the State of Kuwait	National	urban	18+	18+	484	568	*
Kuwait	2008-2010	Gulf Cooperation Council World Health Survey	National	both	18+	18+	1598	1782	*
Kuwait	2009	Kuwait nutrition surveillance system	National	both	18	18	78	109	*
Kuwait	2014	STEPS	National	both	18-69	18-69	1382	2212	†
Kyrgyzstan	1993	Kyrgyzstan Multipurpose Poverty Surveys	National	both	18-60	18-60	2457	2457	*
Kyrgyzstan	1997	DHS	National	both		18-49		3120	†
Kyrgyzstan	2012	DHS	National	both		18-49		6489	†
Kyrgyzstan	2013	STEPS	National	both	25-64	25-64	942	1600	†
Lao PDR	2006	Multiple Indicator Cluster Survey 3	National	both		18-49		698	†
Lao PDR	2008	STEPS	Community	both	25-64	25-64	1568	2353	†
Lao PDR	2013	STEPS	National	both	18-64	18-64	984	1461	†
Latvia	1997	Nutrition and Lifestyle in the Baltic Republics, WHO, 1997	National	both	19-50	19-50	703	732	*
Latvia	2008-2009	Cardiovascular risk factor study	National	both	25-74	25-74	1362	2398	*
Lebanon	1997	Population and Housing Survey	National	both	18+	18+	529	778	*
Lebanon	2008-2009	STEPS	National	both	18+	18+	1319	1476	*
Lebanon	2017	STEPS	National	both	18-69	18-69	729	983	†
Lesotho	1993	National survey on iodine, vitamin A and iron status of women and children in Lesotho	National	both		20-65		792	*
Lesotho	2004-2005	DHS	National	both		18-49		2702	†
Lesotho	2009-2010	DHS	National	both	18-59	18-49	2704	3200	†
Lesotho	2012	STEPS	National	both	25-64	25-64	726	1442	†
Lesotho	2014	DHS	National	both	18-59	18-49	2439	2762	†
Liberia	2006-2007	DHS	National	both		18-49		5674	†
Liberia	2011	STEPS	National	both	25-64	25-64	998	1254	†
Liberia	2013	DHS	National	both	18-49	18-49	3675	4142	†
Libya	1998-1999	Kadiki et al., Diabetes Metab 27(6):647-54, 2001	Community	both	18+	18+	228	398	*
Libya	2009	STEPS	National	both	25-64	25-64	1678	1564	†
Lithuania	1986-1987	MONICA, Kaunas	Community	urban	35-64	35-64	894	868	†
Lithuania	1987	Countrywide Integrated Noncommunicable Diseases Intervention (CINDI) Programme	Subnational	rural	25-64	25-64	1220	1434	*
Lithuania	1992-1993	MONICA, Kaunas	Community	urban	35-64	35-64	610	621	†
Lithuania	1992-1993	Countrywide Integrated Noncommunicable Diseases Intervention (CINDI) Programme	Subnational	rural	25-64	25-64	617	798	*
Lithuania	1997	Pomerleau, 2000	National	both	19+	19+	966	1130	*
Lithuania	1998-1999	Countrywide Integrated Noncommunicable Diseases Intervention (CINDI) Programme	Subnational	rural	25-64	25-64	816	1021	*
Lithuania	2001-2002	MONICA4	Community	urban	35-64	35-64	625	776	*
Lithuania	2002	Pomerleau et al., Public Health Nutr 3:3-10, 2000	National	both	24-70	24-70	977	928	*
Lithuania	2006-2007	Countrywide Integrated Noncommunicable Diseases Intervention (CINDI) Programme	Subnational	rural	25-64	25-64	718	972	*
Lithuania	2006-2008	Health, Alcohol and Psychosocial factors In Eastern Europe (HAPIEE)	Community	urban	45-75	45-75	3231	3874	*
Luxembourg	2007-2009	Observation of cardiovascular risk factors in Luxembourg (ORISCAV-LUX)	National	both	18-69	18-69	696	735	*
Macedonia (TFYR)	1999	Multiple Indicator Cluster Survey	National	both		18-45		1029	*

Country	Data years	Survey/study name/citation	Level of representative-ness	Rural, urban or both	Age range as used for global analysis		Sample size as used for global analysis		Note
					Male	Female	Male	Female	
Madagascar	1997	Mauny et al., Ann Trop Med Parasitol 97(6):645-54, 2003	Community	both	18+	18+	248	283	*
Madagascar	1997	DHS	National	both		20-49		2253	†
Madagascar	2003-2004	DHS	National	both		18-49		6366	†
Madagascar	2005	STEPS	Subnational	both	25-64	25-64	2596	2494	†
Madagascar	2008-2009	DHS	National	both		18-49		6723	†
Malawi	1992	DHS	National	both		20-49		2102	†
Malawi	1996	Chilima et al., Eur J Clin Nutr 52(9):643-9, 1998	Community	rural	55-94	55-94	86	185	*
Malawi	2000	DHS	National	both		18-49		9973	†
Malawi	2004	DHS	National	both		18-49		8563	†
Malawi	2009	STEPS	National	both	25-64	25-64	1666	3189	†
Malawi	2010	DHS	National	both		18-49		6006	†
Malawi	2013-2017	NCD Survey Malawi Epidemiology and Intervention Research Unit	Community	both	18+	18+	11651	17798	*
Malawi	2015-2016	DHS	National	both		18-49		6451	†
Malawi	2017	STEPS	National	both	18-70	18-70	1478	2534	†
Malaysia	1996	National Health and Morbidity Survey (NHMS)	National	both	18+	18+	14520	16244	*
Malaysia	2002-2003	Malaysian Adult Nutrition Survey	National	both	18-59	18-59	3302	3395	*
Malaysia	2004	Rampal et al., Public Health 122:11-8, 2008	National	both	18+	18+	6254	8550	*
Malaysia	2005	STEPS	National	both	25-64	25-64	1286	1286	*
Malaysia	2006	National Health and Morbidity Survey (NHMS)	National	both	18+	18+	15025	17824	*
Malaysia	2008	Metabolic Syndrome Study in Malaysia	National	both	18+	18+	1522	2814	*
Malaysia	2011	National Health and Morbidity Survey (NHMS)	National	both	18+	18+	8033	8780	*
Malaysia	2014	Malaysian Adult Nutrition Survey	National	both	18-59	18-59	1356	1533	*
Malaysia	2015	National Health and Morbidity Survey (NHMS)	National	both	18+	18+	8916	9581	*
Maldives	2001	Multiple Indicator Cluster Survey	National	both		18-50		1145	*
Maldives	2004	STEPS	Subnational	urban	25-64	25-64	933	1086	*
Maldives	2009	DHS	National	both		20-49		5139	†
Maldives	2011	STEPS	Subnational	urban	18-64	18-64	588	1000	†
Mali	1995-1996	DHS	National	both		20-49		3789	†
Mali	1997	Torheim et al., Eur J Clin Nutr 58(4):594-604, 2004	Subnational	rural	18-44	18-44	237	337	*
Mali	1999	Torheim et al., Public Health Nutr 8(4):387-94, 2005	Subnational	rural		18-44		191	*
Mali	2001	DHS	National	both		18-49		9281	†
Mali	2006	DHS	National	both		18-49		10726	†
Mali	2007	STEPS	Subnational	both	18-64	18-64	931	1315	†
Mali	2012-2013	DHS	National	both		18-49		4156	†
Mali	2013	Santé Nutritionnelle à Assise Comunautaire dans la région de Kayes (SNACK)	Subnational	rural		20-68		4595	*
Malta	1986	INTERSALT	Community	rural	20-59	20-59	100	100	†
Marshall Islands	2002	STEPS	National	both	18-64	18-64	686	1058	†
Mauritania	2000-2001	DHS	National	both		18-49		2635	*
Mauritania	2006	STEPS	Community	urban	18-64	18-64	1065	1218	†
Mauritius	1987	Mauritius non-communicable disease survey	National	both	25-74	25-74	2345	2652	*
Mauritius	1992	Mauritius non-communicable disease survey	National	both	25-74	25-74	2985	3477	*
Mauritius	1998	Mauritius non-communicable disease survey	National	both	25-74	25-74	2566	3248	*
Mauritius	2009	Mauritius non-communicable disease survey	National	both	19+	19+	2860	3391	*
Mexico	1988-1989	Encuesta Nacional de Nutrición	National	both		18-49		12043	*
Mexico	1992-1993	Encuesta Nacional de Enfermedades Cronicas	National	urban	20-69	20-69	6040	8298	*
Mexico	1996	Sanchez-Castillo et al., Eur J Clin Nutr 55(10):833-40, 2001	Community	rural	18+	18+	104	149	*
Mexico	1998-1999	Encuesta Nacional de Nutrición	National	both		18-49		13884	*
Mexico	1998-2004	Mexico City Prospective Study	Community	urban	35-84	35-84	51768	105313	*
Mexico	1999-2000	The Survey on Health, Well-Being, and Aging in Latin America and the Caribbean (SABE)	Community	urban	60+	60+	359	548	2†
Mexico	2000	Encuesta Nacional de Salud	National	both	18+	18+	14692	30262	*
Mexico	2001	The Mexican Health and Aging Study	National	both	50+	50+	1030	1224	†
Mexico	2002	Encuesta Nacional Sobre Niveles de vida de los Hogares	National	both	18+	18+	7332	9086	†
Mexico	2003	The Mexican Health and Aging Study	National	both	50+	50+	893	1162	†
Mexico	2004-2005	CArdiovascular Risk factors Multiple Evaluation in Latin America (CARMELA)	Community	urban	25-64	25-64	833	894	*
Mexico	2005	Encuesta Nacional Sobre Niveles de vida de los Hogares	National	both	18+	18+	7551	9086	†
Mexico	2006	Encuesta Nacional de Salud y Nutrición	National	both	18+	18+	14471	21429	*
Mexico	2006	PREVENIMSS National Coverage Surveys	National	urban	20+	20+	8715	11315	*
Mexico	2009-2010	SAGE	National	both	50+	50+	796	1236	†
Mexico	2009-2012	Encuesta Nacional Sobre Niveles de vida de los Hogares	National	both	18+	18+	4393	4108	†

Country	Data years	Survey/study name/citation	Level of representative-ness	Rural, urban or both	Age range as used for global analysis		Sample size as used for global analysis		Note
					Male	Female	Male	Female	
Mexico	2010	PREVENIMSS National Coverage Surveys	National	urban	20+	20+	6238	6003	*
Mexico	2011-2012	Encuesta Nacional de Salud y Nutrición	National	both	18+	18+	17511	23418	†
Mexico	2012	The Mexican Health and Aging Study	National	both	50+	50+	786	1106	†
Mexico	2016	Encuesta Nacional de Salud y Nutrición	National	both	18+	18+	3073	5759	†
Micronesia (Federated States of)	2002	STEPS	Subnational	both	25-64	25-64	591	893	†
Micronesia (Federated States of)	2006	STEPS	Subnational	both	18-64	18-64	825	1420	†
Micronesia (Federated States of)	2008	STEPS	Subnational	both	25-64	25-64	875	1266	†
Micronesia (Federated States of)	2009	STEPS, Kosrae	Subnational	both	18-64	18-64	208	413	†
Micronesia (Federated States of)	2009	STEPS, Yap	Subnational	both	18-64	18-64	391	518	†
Moldova	2005	DHS	National	both		18-49		6268	†
Moldova	2013	STEPS	National	both	18-69	18-69	1712	2777	†
Mongolia	1999	National Nutrition Survey	National	both	35-65	35-65	907	1317	*
Mongolia	2004	National Nutrition Survey	National	both	18-74	18-74	248	360	*
Mongolia	2005	STEPS	National	both	18-64	18-64	1513	1590	†
Mongolia	2009	STEPS	National	both	18-64	18-64	2079	2965	†
Mongolia	2013	STEPS	National	both	18-64	18-64	2317	2807	†
Montenegro	2016	Anthropometric parameters as an indicator of obesity at adolescents in Montenegro	National	both	18	18	139	158	*
Morocco	1992	DHS	National	both		20-49		2804	†
Morocco	2000	National Survey 2000	National	both	20+	20+	755	1047	*
Morocco	2003-2004	DHS	National	both		18-49		13944	†
Morocco	2017	STEPS	National	both	18-100	18-100	1871	3390	†
Mozambique	1997	DHS	National	both		20-49		2824	†
Mozambique	2003	DHS	National	both		18-49		9241	†
Mozambique	2005	STEPS	National	both	25-64	25-64	1276	1689	†
Mozambique	2011	DHS	National	both		18-49		10396	†
Mozambique	2014-2015	STEPS	National	both	18-64	18-64	1049	1565	*
Myanmar	2003-2004	STEPS	Subnational	both	25-74	25-74	1990	2449	*
Myanmar	2009	STEPS	National	both	18-64	18-64	2732	4277	*
Myanmar	2011	Underweight prevalence among young adults from rural areas, Salin Township, Magwe Region	Community	rural	18-35	18-35	127	207	*
Myanmar	2014	STEPS	National	both	25-64	25-64	2947	5444	*
Myanmar	2014	STEPS, Yangon	Subnational	both	25-74	25-74	745	740	*
Myanmar	2015-2016	DHS	National	both		18-49		11081	†
Namibia	1992	DHS	National	both		20-49		2062	†
Namibia	2005	STEPS	National	both	24-64	24-64	1390	1778	†
Namibia	2006-2007	DHS	National	both		18-49		7698	†
Namibia	2009	Okambilimbili Survey	Community	urban	18+	18+	752	949	*
Namibia	2013	DHS	National	both		18-64		4632	†
Nauru	1982	Trends in the prevalence and incidence of non-insulin-dependent diabetes mellitus and impaired glucose tolerance	National	both	20+	20+	701	773	1*
Nauru	1987	Trends in the prevalence and incidence of non-insulin-dependent diabetes mellitus and impaired glucose tolerance	National	both	20+	20+	555	667	*
Nauru	1994	Trends in the prevalence and incidence of non-insulin-dependent diabetes mellitus and impaired glucose tolerance	National	both	25+	25+	647	731	*
Nauru	2004	STEPS	National	both	18-64	18-64	1011	1078	†
Nauru	2006	STEPS	National	both	18-65	18-65	244	230	*
Nepal	1996	DHS	National	both		20-49		3068	†
Nepal	1997	Ohno et al., Asia Pac J Public Health 18(3):20-9, 2006	Community	rural	18-75	18-75	36	41	*
Nepal	2001	DHS	National	both		20-49		7216	†
Nepal	2003	STEPS	Subnational	both	25-64	25-64	1010	996	*
Nepal	2005	STEPS	Subnational	both	18-64	18-64	3431	3790	*
Nepal	2006	DHS	National	both		18-49		8677	†
Nepal	2006-2011	Early detection and management of Kidney disease, Hypertension, Diabetes and Cardiovascular disease (KHDC Nepal), Tara	Community	rural	18+	18+	1175	2350	*
Nepal	2006-2011	Early detection and management of Kidney disease, Hypertension, Diabetes and Cardiovascular disease (KHDC Nepal), Dam	Community	urban	18+	18+	1095	1576	*
Nepal	2006-2011	Early detection and management of Kidney disease, Hypertension, Diabetes and Cardiovascular disease (KHDC Nepal), Dha	Community	urban	18+	18+	4130	6126	*
Nepal	2007-2008	STEPS	National	both	18-64	18-64	1743	2210	*
Nepal	2011	DHS	National	both		18-49		5076	†
Nepal	2013	STEPS	National	both	18-69	18-69	1275	2703	†
Nepal	2015	Community based intervention for prevention and control of non-communicable diseases risk factors (CIPCON) baseline surv	Community	rural	18-69	18-69	546	772	*
Nepal	2015	Community based intervention for prevention and control of non-communicable diseases risk factors (CIPCON) baseline surv	Community	rural	18-69	18-69	538	703	*
Nepal	2016	DHS	National	both	18-49	18-49	3445	5382	†
Netherlands	1985	INTERSALT	Community	urban	20-59	20-59	100	99	†
Netherlands	1985	Zutphen Elderly Study	Community	urban	64-85		886		*

Country	Data years	Survey/study name/citation	Level of representative-ness	Rural, urban or both	Age range as used for global analysis		Sample size as used for global analysis		Note
					Male	Female	Male	Female	
Netherlands	1989-1993	The Rotterdam Study, first subcohort	Community	urban	55+	55+	2807	4103	*
Netherlands	1990	Zutphen Elderly Study	Community	urban	69-90		552		*
Netherlands	1992-1993	The Longitudinal Aging Study Amsterdam (LASA)	Subnational	both	55-85	55-85	1266	1308	4*
Netherlands	1993-1995	The Rotterdam Study, first subcohort	Community	urban	56+	56+	2214	3105	*
Netherlands	1993-1997	EPIC Bilthoven	Community	urban	20-59	20-59	9941	12021	*
Netherlands	1993-1997	EPIC Utrecht	Community	both		49-70		17335	*
Netherlands	1995-1996	The Longitudinal Aging Study Amsterdam (LASA)	Subnational	both	58-88	58-88	715	765	4*
Netherlands	1997-1999	The Rotterdam Study, first subcohort	Community	urban	61+	61+	1718	2361	*
Netherlands	1998-1999	The Longitudinal Aging Study Amsterdam (LASA)	Subnational	both	61-91	61-91	604	742	4*
Netherlands	1998-2001	Regenboog Project	National	both	18-89	18-89	2581	2490	*
Netherlands	2000-2001	The Rotterdam Study, second subcohort	Community	urban	55+	55+	1210	1468	*
Netherlands	2001-2002	The Longitudinal Aging Study Amsterdam (LASA)	Subnational	both	64-94	64-94	576	689	4*
Netherlands	2001-2003	Surinamese in the Netherlands: Study on Ethnicity and Health (SUNSET)	Community	urban	35-60	35-60	251	257	*
Netherlands	2002-2003	The Longitudinal Aging Study Amsterdam (LASA)	Subnational	both	54-65	54-65	431	482	4*
Netherlands	2002-2004	The Rotterdam Study, first subcohort	Community	urban	64+	64+	1206	1708	*
Netherlands	2003-2007	Doetinchem Cohort Study (4th measurement)	Subnational	urban	36-74	36-74	2125	2352	*
Netherlands	2004-2005	The Rotterdam Study, second subcohort	Community	urban	58+	58+	964	1244	*
Netherlands	2005-2006	The Longitudinal Aging Study Amsterdam (LASA)	Subnational	both	57-97	57-97	788	958	4*
Netherlands	2006-2008	The Rotterdam Study, third subcohort	Community	urban	45+	45+	1547	2029	*
Netherlands	2008-2009	The Longitudinal Aging Study Amsterdam (LASA)	Subnational	both	60-100	60-100	641	788	4*
Netherlands	2009-2010	Measuring the Netherlands (NL de Maat)	Subnational	both	30-70	30-70	1781	2014	*
Netherlands	2009-2011	The Rotterdam Study, first subcohort	Community	urban	72+	72+	690	1006	*
Netherlands	2011-2012	The Rotterdam Study, second subcohort	Community	urban	65+	65+	735	934	*
Netherlands	2011-2012	The Longitudinal Aging Study Amsterdam (LASA)	Subnational	both	63-104	63-104	529	652	4*
Netherlands	2011-2015	Healthy Life in an Urban Setting (HELIUS)	Community	urban	18-71	18-71	2088	2473	*
Netherlands	2012-2014	The Rotterdam Study, third subcohort	Community	urban	52+	52+	1256	1639	*
New Zealand	1989	The Life in New Zealand Survey	National	both	18+	18+	1345	1485	*
New Zealand	1990-1993	Williams, N Z Med J 113(1114):308-11, 2000	Community	both	18-21	18-21	932	859	*
New Zealand	1993-1994	MONICA, Auckland	Community	urban	35-64	35-64	723	674	†
New Zealand	1996-1997	National Nutrition Survey	National	both	18+	18+	1777	2423	*
New Zealand	2002-2003	New Zealand Health Survey	National	both	18+	18+	4438	6550	*
New Zealand	2006-2007	New Zealand Health Survey	National	both	18+	18+	4864	6271	*
New Zealand	2008-2009	New Zealand Adult Nutrition Survey	National	both	18+	18+	1754	2215	*
New Zealand	2011-2012	New Zealand Health Survey	National	both	18+	18+	4396	5948	*
New Zealand	2012-2013	New Zealand Health Survey	National	both	18+	18+	4967	6602	*
New Zealand	2013-2014	New Zealand Health Survey	National	both	18+	18+	5396	6807	*
New Zealand	2014-2015	New Zealand Health Survey	National	both	18+	18+	5486	6826	*
New Zealand	2015-2016	New Zealand Health Survey	National	both	18+	18+	5538	6932	*
Nicaragua	1997-1998	DHS	National	both		18-49		10320	†
Nicaragua	2001	DHS	National	both		18-49		10195	†
Nicaragua	2003-2004	CAMDI	Community	urban	20+	20+	773	916	*
Nicaragua	2003-2005	Sistema Integrado de Vigilancia de Intervenciones Nutricionales (SIVIN)	National	both		18-50		1115	*
Nicaragua	2006-2007	Encuesta Nicaraguense de Demografia y Salud	National	both		18-49		11765	*
Nicaragua	2011-2012	Encuesta Nicaraguense de Demografia y Salud	National	both		18-49		12790	*
Niger	1992	DHS	National	both		20-49		2993	†
Niger	1998	DHS	National	both		20-49		2958	†
Niger	2006	DHS	National	both		18-49		3590	†
Niger	2007	STEPS	National	both	18-64	18-64	1356	1131	†
Niger	2012	DHS	National	both		18-49		3935	†
Nigeria	1990	Non-communicable Diseases National Survey	National	both	18+	18+	5236	5327	*
Nigeria	1991-1994	Cooper et al., Am J Public Health 87(2):160-68, 1997	Community	both	20-100	20-100	910	1080	*
Nigeria	1999	DHS	National	both		20-49		2004	†
Nigeria	1999-2009	Prostate cancer dietary risk factors study	Subnational	both	35+		626		*
Nigeria	2003	DHS	National	both		18-49		5664	†
Nigeria	2006	Senbanjo et al., West Afr J Med 30(6):425-31, 2011	Community	urban	18-19	18-19	10	12	*
Nigeria	2007	Ibadan Study of Ageing	Subnational	both	60+	60+	642	914	*
Nigeria	2008	DHS	National	both		18-49		25418	†
Nigeria	2008	Ibadan Study of Ageing	Subnational	both	61+	61+	453	656	*
Nigeria	2009	Ibadan Study of Ageing	Subnational	both	62+	62+	420	619	*

Country	Data years	Survey/study name/citation	Level of representative-ness	Rural, urban or both	Age range as used for global analysis		Sample size as used for global analysis		Note
					Male	Female	Male	Female	
Nigeria	2009	Community Health Plan - Kwara Central Survey	Community	rural	18+	18+	1247	1418	*
Nigeria	2011	Community Health Plan - Kwara Central Survey	Community	rural	18+	18+	396	494	*
Nigeria	2013	DHS	National	both		18-49		29348	†
Nigeria	2013	Community Health Plan - Kwara Central Survey	Community	rural	18+	18+	358	421	*
Niue	2011	STEPS	National	both	18-100	18-100	376	454	†
Norway	1984-1986	HUNT1 Study	Subnational	rural	20+	20+	36517	37811	*
Norway	1986-1987	The Tromsø Study: Tromsø 3	Community	both	20-61	20-56	10295	9710	*
Norway	1994-1995	The Tromsø Study: Tromsø 4	Community	both	25+	25+	12516	13539	*
Norway	1995-1997	HUNT2 study	Subnational	rural	20+	20+	30285	33599	*
Norway	1995-1997	Young-HUNT1 Study	Subnational	rural	18-21	18-21	652	703	*
Norway	1997-1999	The Hordaland Health Study (HUSK)	Community	urban	70-74	70-74	1465	1839	*
Norway	1997-1999	The Hordaland Health Study (HUSK)	Subnational	both	40-47	40-47	10180	11928	*
Norway	2000-2001	Young-HUNT2 Study	Subnational	rural	18-21	18-21	413	543	*
Norway	2000-2003	the Oslo cohort (HUBRO), the Oppland and Hedmark cohort (OPPHED), and the Troms and Finnmark cohort (TROFINN)	Subnational	both	30-76	30-76	16567	19926	*
Norway	2001-2002	The Tromsø Study: Tromsø 5, Tromsø Study Panel	Community	both	30-89	30-89	2523	3579	*
Norway	2006-2008	HUNT3 Study	Subnational	rural	20+	20+	22860	27553	*
Norway	2006-2008	Young-HUNT3 Study	Subnational	rural	18-21	18-21	503	592	*
Norway	2007-2008	The Tromsø Study: Tromsø 6	Community	both	30-87	30-87	6048	6889	*
Occupied Palestinian Territory	1996	Stene et al., Eur J Clin Nutr 55(9):805-11, 2001	Community	rural	30-65	30-65	208	269	*
Occupied Palestinian Territory	1996-1998	Rural Kobar and urban Old Ramallah	Community	both	18-64	18-64	388	900	*
Occupied Palestinian Territory	1999-2000	The First National Health and Nutrition Survey	National	both	18-64	18-64	1319	1272	*
Occupied Palestinian Territory	2010	STEPS	National	both	18-64	18-64	2324	3830	†
Oman	1991	Oman National Health Survey	National	both	20+	20+	2128	2958	*
Oman	2000	Oman National Health Survey	National	both	20+	20+	3069	3331	*
Oman	2001	Al-Lawati et al., Diabetes Care 26(6):1781-85, 2003	Community	urban	20+	20+	755	756	*
Oman	2006	STEPS	Community	urban	20-59	20-59	540	732	*
Oman	2008	Gulf Cooperation Council World Health Survey	National	both	18+	18+	2389	2112	*
Pakistan	1990-1994	MHS	Community	urban	18+	18+	432	478	*
Pakistan	1990-1994	National Health Survey Of Pakistan 1990-1994	National	both	18+	18+	3789	4346	*
Pakistan	1999	Shah et al., Trop Med Int Health 9(4):526-32, 2004	Community	both	18+	18+	1391	2754	*
Pakistan	2004-2005	COBRA-1	Community	urban	40+	40+	1500	1635	*
Pakistan	2005	STEPS	National	both	25-65	25-65	787	1071	*
Pakistan	2011	National Nutrition Survey	National	both	18-49	18-49	7312	30383	*
Pakistan	2012-2013	DHS	National	both		20-49		3968	†
Pakistan	2014	STEPS	Subnational	both	18-69	18-69	2964	3674	†
Palau	2011-2013	STEPS	National	both	25-64	25-64	1031	1124	†
Panama	2003	Second Living Standards Survey	National	both	18-75	18-75	6844	7100	*
Panama	2010-2011	Prevalencia de factores de riesgo asociados a enfermedad cardiovascular 2010-2011	Subnational	both	18+	18+	1067	2469	*
Papua New Guinea	1985-1986	INTERSALT	Community	rural	20-59	20-59	88	74	†
Papua New Guinea	2007	STEPS	National	both	18-64	18-64	1281	1317	†
Paraguay	2011	Primera Encuesta Nacional de Factores de Riesgo de Enfermedades No Transmisibles en Poblacion General	National	both	18-75	18-75	877	1510	*
Peru	1991-1992	DHS	National	both		20-49		4887	†
Peru	1996	DHS	National	both		20-49		10125	†
Peru	2000	DHS	National	both		18-49		22078	†
Peru	2004-2005	CArdiovascular Risk factors Multiple Evaluation in Latin America (CARMELA)	Community	urban	25-64	25-64	769	876	*
Peru	2004-2005	Encuesta Nacional de Indicadores Nutricionales, Bioquímicos, Socioeconómicos y Culturales Relacionados con las Enfermec	National	both	20+	20+	2087	2095	*
Peru	2004-2006	DHS	National	both		18-49		5030	†
Peru	2005	Prevalencia de Factores de Riesgo de Enfermedades No Transmisibles (FRENT) study, Huancayo	Community	urban	18+	18+	198	530	*
Peru	2006	Prevalencia de Factores de Riesgo de Enfermedades No Transmisibles (FRENT) study, Lima	Community	urban	18+	18+	616	1052	*
Peru	2007-2008	Monitoreo de Indicadores Nutricionales en la ENAHO 2007-2008	National	both	18+	18+	9691	11038	†
Peru	2007-2008	DHS	National	both		18-49		18357	†
Peru	2007-2008	PERU MIGRANT Study	Community	both	30+	30+	464	522	*
Peru	2009	DHS	National	both		18-49		20118	†
Peru	2009-2011	Monitoreo de Indicadores Nutricionales en la ENAHO 2009-2010	National	both	18+	18+	17629	21366	†
Peru	2009-2012	CRONICAS Cohort Study	Subnational	both	35+	35+	1557	1660	*
Peru	2010	DHS	National	both		18-49		19529	†
Peru	2010-2013	CRONICAS Cohort Study	Subnational	both	35+	35+	1379	1468	*
Peru	2011	DHS	National	both		18-49		19429	†
Peru	2011-2012	Monitoreo de Indicadores Nutricionales en la ENAHO 2011	National	both	18+	18+	4877	5872	†

Country	Data years	Survey/study name/citation	Level of representative-ness	Rural, urban or both	Age range as used for global analysis		Sample size as used for global analysis		Note
					Male	Female	Male	Female	
Peru	2012	DHS	National	both		18-49		20859	†
Peru	2012-2013	PERU MIGRANT Study	Community	both	35+	35+	339	427	*
Peru	2013	DHS	National	both	18+	18+	2731	21171	†
Peru	2013	Clinical functional and sociofamilial profiles of the elderly from a community in a district of Lima, Peru	Community	urban	60+	60+	185	309	*
Peru	2013-2014	CRONICAS Cohort Study	Subnational	both	36+	36+	1292	1361	*
Peru	2014	DHS	National	both	18+	18+	11795	25900	†
Peru	2014	Launching a salt substitute to reduce blood pressure at the population level: a cluster randomized stepped wedge trial in Peru	Subnational	both	18+	18+	1149	1166	*
Peru	2015	DHS	National	both	18+	18+	13773	35241	†
Peru	2015-2016	PERU MIGRANT Study	Community	both	38+	38+	323	413	*
Peru	2016	DHS	National	both	18+	18+	13109	33055	†
Peru	2016-2017	Sceening of type II diabetes mellitus	Community	urban	30-70	30-70	798	809	*
Peru	2017	DHS	National	both	18+	18+	13467	34408	†
Philippines	1984-1985	Cebu Longitudinal Health and Nutrition Survey Baseline 16-Month Follow-up	Community	both		18-50		2113	†
Philippines	1984-1985	Cebu Longitudinal Health and Nutrition Survey Baseline 18-Month Follow-up	Community	both		18-50		2066	†
Philippines	1985-1986	Cebu Longitudinal Health and Nutrition Survey Baseline 20-Month Follow-up	Community	both		18-50		2037	†
Philippines	1985-1986	Cebu Longitudinal Health and Nutrition Survey Baseline 22-Month Follow-up	Community	both		18-50		2006	†
Philippines	1985-1986	Cebu Longitudinal Health and Nutrition Survey Baseline 24-Month Follow-up	Community	both		18-50		2015	†
Philippines	1988	INCLEN	Community	rural	35-65		274		*
Philippines	1991-1992	Cebu Longitudinal Health and Nutrition Survey 1991 Mother Follow-up	Community	both		22-55		2195	†
Philippines	1993	4th National Nutrition Survey	National	both	20-70	20-70	4383	4754	*
Philippines	1993	National Safe Motherhood Survey	National	both		18-49		7181	*
Philippines	1994-1995	Cebu Longitudinal Health and Nutrition Survey 1994-1995 Mother Follow-up	Community	both		18-59		2552	†
Philippines	1998	5th National Nutrition Survey Philippine	National	both	20-60	20-60	1323	1340	*
Philippines	1998-1999	Cebu Longitudinal Health and Nutrition Survey 1998-1999 Mother Follow-up	Community	both		18-59		1911	†
Philippines	2002	Cebu Longitudinal Health and Nutrition Survey 2002 Child Follow-up	Community	both	18-19	18-19	1084	897	†
Philippines	2002	Cebu Longitudinal Health and Nutrition Survey 2002 Mother Follow-up	Community	both		32-66		2080	†
Philippines	2003	6th National Nutrition Survey	National	both	20+	20+	6311	6949	*
Philippines	2005	Cebu Longitudinal Health and Nutrition Survey 2005 Child Follow-up	Community	both	20-22	20-22	1006	831	†
Philippines	2005	Cebu Longitudinal Health and Nutrition Survey 2005 Mother Follow-up	Community	both		35-69		2001	†
Philippines	2007	Cebu Longitudinal Health and Nutrition Survey 2007 Child Follow-up	Community	both	23-24	23-24	937	751	†
Philippines	2007	Cebu Longitudinal Health and Nutrition Survey 2007 Mother Follow-up	Community	both		38-71		1925	†
Philippines	2008	7th National Nutrition Survey	National	both	20+	20+	37687	38952	*
Philippines	2009	Cebu Longitudinal Health and Nutrition Survey 2009 Child Follow-up	Community	both	24-26	24-26	864	718	†
Philippines	2013-2014	8th National Nutrition Survey	National	both	18+	18+	35507	40834	*
Philippines	2015	2015 Updating of Nutritional Status of Filipino Children and Other Population Groups	National	both	18+	18+	43151	48510	*
Poland	1986	Poland Conscripts 10% Sample Cohort	National	both	18-19		29380		*
Poland	1986	INTERSALT, Krakow	Community	urban	20-59	20-59	100	100	†
Poland	1986	INTERSALT, Warsaw	Community	urban	20-59	20-59	100	100	†
Poland	1987-1988	MONICA, Tarnobrzeg Voivodship	Community	rural	35-64	35-64	616	672	†
Poland	1988-1989	MONICA, Warsaw	Community	urban	35-64	35-64	705	713	†
Poland	1989-1990	Polish Program CINDI (CINDI Lodz 1989-1990)	Community	urban	25-64	25-64	831	957	*
Poland	1992-1993	MONICA, Tarnobrzeg Voivodship	Community	rural	35-64	35-64	618	692	†
Poland	1993	MONICA, Warsaw	Community	urban	35-64	35-64	751	763	†
Poland	1995	Poland Conscripts 10% Sample Cohort	National	both	18-19		31007		*
Poland	1995-1996	Polish Program CINDI (CINDI Lodz 1995)	Community	urban	18-64	18-64	964	1411	*
Poland	2000	The health status, risk factors of chronic diseases and health behaviors of residents of Torun (CINDI Torun 2000)	Community	urban	18-83	18-83	931	1017	*
Poland	2000	Binkowska-Bury et al., Neuro Endocrinol Lett 34(8):814-20, 2013	Subnational	both	19-20		3003		*
Poland	2000-2001	Household Food Consumption and Anthropometric Survey	National	both	18+	18+	1287	1613	*
Poland	2001	Binkowska-Bury et al., Neuro Endocrinol Lett 34(8):814-20, 2013	Subnational	both	19-20		3420		*
Poland	2001	Poland Conscripts 10% Sample Cohort	National	both	18-19		31211		*
Poland	2001-2002	The health status, risk factors of chronic diseases and health behaviors of residents of Lodz (CINDI Lodz 2001)	Community	urban	18-64	18-64	1000	840	*
Poland	2002	The health status, risk factors of chronic diseases and health behaviors of residents of Lodz - seniors (CINDI Lodz 2002)	Community	urban	65+	65+	285	532	*
Poland	2002	Binkowska-Bury et al., Neuro Endocrinol Lett 34(8):814-20, 2013	Subnational	both	19-20		3544		*
Poland	2002	NATPOL	National	both	18+	18+	1018	1301	*
Poland	2002-2005	Health, Alcohol and Psychosocial factors In Eastern Europe (HAPIEE)	Community	urban	45-69	45-69	4454	4720	*
Poland	2003	Binkowska-Bury et al., Neuro Endocrinol Lett 34(8):814-20, 2013	Subnational	both	19-20		3633		*
Poland	2003	The European Male Ageing Study	Community	both	40+		406		*
Poland	2003-2005	National Multicenter Health Survey in Poland. Project WOBASZ	National	both	20-74	20-74	6245	6910	*
Poland	2003-2013	Mogielica Human Ecology Study	Community	rural	21+	21+	353	896	*

Country	Data years	Survey/study name/citation	Level of representative-ness	Rural, urban or both	Age range as used for global analysis		Sample size as used for global analysis		Note
					Male	Female	Male	Female	
Poland	2004	Binkowska-Bury et al., Neuro Endocrinol Lett 34(8):814-20, 2013	Subnational	both	19-20		3538		*
Poland	2004	LIPIDOGRAM2004 Study - National epidemiological study of lipid disorders and selected risk factors of cardiovascular dise	National	both	30+	30+	6673	9920	*
Poland	2005	Binkowska-Bury et al., Neuro Endocrinol Lett 34(8):814-20, 2013	Subnational	both	19-20		3308		*
Poland	2006	The health, risk factors for chronic diseases, attitudes and behaviors of health residents of Torun (CINDI Torun 2006)	Community	urban	18-65	18-65	749	1115	*
Poland	2006	Binkowska-Bury et al., Neuro Endocrinol Lett 34(8):814-20, 2013	Subnational	both	19-20		3701		*
Poland	2006	LIPIDOGRAM2006 Study - National epidemiological study of lipid disorders and selected risk factors of cardiovascular dise	National	both	32+	32+	6441	10640	*
Poland	2006-2007	National Multicenter Health Survey in Poland. Project WOBASZ Senior	National	both	75+	75+	541	533	*
Poland	2007	Binkowska-Bury et al., Neuro Endocrinol Lett 34(8):814-20, 2013	Subnational	both	19-20		3612		*
Poland	2007-2009	Elaboration of the reference range of arterial blood pressure for the population of children and adolescents in Poland - PL008	National	both	18	18	440	597	*
Poland	2007-2011	Medical, psychological and socioeconomic aspects of aging in Poland	National	both	55+	55+	2750	2582	*
Poland	2008	Binkowska-Bury et al., Neuro Endocrinol Lett 34(8):814-20, 2013	Subnational	both	19-20		3435		*
Poland	2008	The European Male Ageing Study	Community	both	40+		310		*
Poland	2009	Binkowska-Bury et al., Neuro Endocrinol Lett 34(8):814-20, 2013	Subnational	both	19-20		3405		*
Poland	2010	Binkowska-Bury et al., Neuro Endocrinol Lett 34(8):814-20, 2013	Subnational	both	19-20		3317		*
Poland	2011	NATPOL	National	both	18-79	18-79	1158	1235	*
Poland	2013-2014	National Multicenter Health Survey in Poland. Project WOBASZ II	National	both	20+	20+	2626	3198	*
Poland	2015-2016	LIPIDOGRAM2015 & LIPIDOGEN2015 Study - National epidemiological study of lipid disorders and selected risk factors c	National	both	18+	18+	5034	8690	*
Portugal	1985	Body Mass Index of Portuguese Conscripts	National	both	18-20		29151		*
Portugal	1986	Body Mass Index of Portuguese Conscripts	National	both	18-20		69318		*
Portugal	1986	INTERSALT	Community	rural	20-59	20-59	99	99	†
Portugal	1987	Body Mass Index of Portuguese Conscripts	National	both	18-20		66930		*
Portugal	1988	Body Mass Index of Portuguese Conscripts	National	both	18-20		66627		*
Portugal	1989	Body Mass Index of Portuguese Conscripts	National	both	18-20		67703		*
Portugal	1990	Body Mass Index of Portuguese Conscripts	National	both	18-20		42572		*
Portugal	1991	Body Mass Index of Portuguese Conscripts	National	both	18-20		16697		*
Portugal	1992	Body Mass Index of Portuguese Conscripts	National	both	18-20		51388		*
Portugal	1993	Body Mass Index of Portuguese Conscripts	National	both	18-20		58904		*
Portugal	1994	Body Mass Index of Portuguese Conscripts	National	both	18-20		54076		*
Portugal	1995	Body Mass Index of Portuguese Conscripts	National	both	18-20		67509		*
Portugal	1996	Body Mass Index of Portuguese Conscripts	National	both	18-21		104839		*
Portugal	1997	Body Mass Index of Portuguese Conscripts	National	both	18-21		59770		*
Portugal	1998	Body Mass Index of Portuguese Conscripts	National	both	18-21		40082		*
Portugal	1999	Body Mass Index of Portuguese Conscripts	National	both	18-21		52572		*
Portugal	1999-2003	EPIPorto Study	Community	urban	18+	18+	932	1507	*
Portugal	2000	Body Mass Index of Portuguese Conscripts	National	both	18-21		51440		*
Portugal	2003-2005	Estudo de Prevalência da Obesidade e Consumos Alimentares em Portugal	National	both	18-64	18-64	3144	3570	*
Portugal	2004	Growth of adolescents in Gouveia	Community	rural	18-19	18-19	2	5	*
Portugal	2007	Growth of adolescents in Tondela	Community	rural		18-19		2	*
Portugal	2007-2009	Portuguese National Survey of Physical Activity and Physical Fitness	National	both	18+	18+	3233	5593	*
Portugal	2008	Azorean Physical Activity and Health Study II	Subnational	both	18	18	63	81	*
Portugal	2010-2012	Exercise for Elderly	Community	urban	60-84	60-84	48	104	*
Portugal	2011-2013	EPITeen - Epidemiological Health Investigation of Teenagers in Porto	Community	urban	20-23	20-23	854	895	*
Portugal	2011-2014	Longitudinal Analysis of Biomarkers and Environmental Determinants of Physical activity (LABMED Study)	Subnational	urban	18	18	37	18	*
Puerto Rico	2002-2003	Puerto Rican Elderly: Health Conditions	National	both	60+	60+	1914	2850	5*
Puerto Rico	2005-2007	Perez et al., Ethn Dis 18(4):434-41, 2008	Community	urban	18-84	18-84	275	529	*
Puerto Rico	2006-2007	Puerto Rican Elderly: Health Conditions	National	both	60+	60+	1056	1669	5*
Puerto Rico	2010-2013	HPV Infection in a Population-Based Sample of Puerto Rican Women	Subnational	both		18-64		557	*
Qatar	2006	World Health Survey	National	both	18+	18+	1859	2018	*
Qatar	2012	STEPS	National	both	18-64	18-64	1034	1353	†
Romania	1986-1987	MONICA, Bucharest	Community	urban	35-64	35-64	702	873	†
Romania	1997	Somatometria	National	both	18-75	18-75	3142	4063	*
Romania	2009-2011	Study on children in Dolj County, South Romania	Subnational	both	18-21	18-21	87	38	*
Romania	2011-2012	Study for the Evaluation of Prevalence of Hypertension and cArdiovascular Risk among the Adult Population of Romania - S	National	both	18-80	18-80	1023	927	*
Romania	2014	Timis County Study	Community	urban	18-19	18-19	8	5	*
Romania	2015-2016	Study for the Evaluation of Prevalence of Hypertension and cArdiovascular Risk among the Adult Population of Romania - S	National	both	18-80	18-80	936	1034	*
Russian Federation	1984-1986	MONICA, Moscow (control)	Community	urban	35-64	35-64	774	642	†
Russian Federation	1984-1986	MONICA, Moscow, Leninsky district	Community	urban	35-64	35-64	553	622	†
Russian Federation	1984-1986	MONICA, Moscow, Cheremushkinsky district	Community	urban	35-64	35-64	580	579	†
Russian Federation	1985	MONICA, Novosibirsk (intervention)	Community	urban	25-64	25-64	797	818	†

Country	Data years	Survey/study name/citation	Level of representative-ness	Rural, urban or both	Age range as used for global analysis		Sample size as used for global analysis		Note
					Male	Female	Male	Female	
Russian Federation	1985-1986	MONICA, Novosibirsk, Kirowsky district	Community	urban	25-64	25-64	758	774	†
Russian Federation	1985-1986	MONICA, Novosibirsk, Leninsky district	Community	urban	25-64	25-64	624	624	†
Russian Federation	1986	INTERSALT	Community	urban	20-59	20-59	97	97	†
Russian Federation	1988	MONICA, Novosibirsk (intervention)	Community	urban	25-64	25-64	837	852	†
Russian Federation	1988-1989	MONICA, Moscow (control)	Community	urban	35-64	35-64	620	581	†
Russian Federation	1988-1989	MONICA, Moscow, Leninsky district	Community	urban	35-64	35-64	597	612	†
Russian Federation	1988-1989	MONICA, Novosibirsk, Kirowsky district	Community	urban	25-64	25-64	871	705	†
Russian Federation	1992	CINDI	Community	rural	25-64	25-64	377	453	*
Russian Federation	1992	Russian Karelia Survey in Pitkaranta	Community	both	25-64	25-64	380	455	*
Russian Federation	1992-1993	Russia Longitudinal Monitoring Survey-Higher School of Economics Round II	National	both	18+	18+	4361	5938	†
Russian Federation	1992-1995	MONICA, Moscow (control)	Community	urban	35-64	35-64	556	527	†
Russian Federation	1992-1995	MONICA, Moscow, Leninsky district	Community	urban	35-64	35-64	538	858	†
Russian Federation	1993	Russia Longitudinal Monitoring Survey-Higher School of Economics Round III	National	both	18+	18+	4519	6327	†
Russian Federation	1993-1994	Russia Longitudinal Monitoring Survey-Higher School of Economics Round IV	National	both	18+	18+	4035	5668	†
Russian Federation	1994	Russia Longitudinal Monitoring Survey-Higher School of Economics Round V	National	both	18+	18+	3574	4702	†
Russian Federation	1994-1995	MONICA, Novosibirsk (intervention)	Community	urban	25-64	25-64	820	860	†
Russian Federation	1995	MONICA, Novosibirsk, Kirowsky district	Community	urban	25-64	25-64	771	787	†
Russian Federation	1995	Russia Longitudinal Monitoring Survey-Higher School of Economics Round VI	National	both	18+	18+	3358	4452	†
Russian Federation	1996	Russia Longitudinal Monitoring Survey-Higher School of Economics Round VII	National	both	18+	18+	3317	4428	†
Russian Federation	1997	Russian Karelia Survey in Pitkaranta	Community	both	25-64	25-64	309	440	*
Russian Federation	1998-1999	Russia Longitudinal Monitoring Survey-Higher School of Economics Round VIII	National	both	18+	18+	3433	4553	†
Russian Federation	2000	Russia Longitudinal Monitoring Survey-Higher School of Economics Round IX	National	both	18+	18+	3521	4771	†
Russian Federation	2001	Russia Longitudinal Monitoring Survey-Higher School of Economics Round X	National	both	18+	18+	3881	5387	†
Russian Federation	2002	Russian Karelia Survey in Pitkaranta	Community	both	25-64	25-64	251	334	*
Russian Federation	2002	Russia Longitudinal Monitoring Survey-Higher School of Economics Round XI	National	both	18+	18+	4070	5542	†
Russian Federation	2002-2005	Health, Alcohol and Psychosocial factors In Eastern Europe (HAPIEE)	Community	urban	45-69	45-69	4208	5040	*
Russian Federation	2003	Russia Longitudinal Monitoring Survey-Higher School of Economics Round XII	National	both	18+	18+	4132	5631	†
Russian Federation	2004	Russia Longitudinal Monitoring Survey-Higher School of Economics Round XIII	National	both	18+	18+	4149	5645	†
Russian Federation	2005	Russia Longitudinal Monitoring Survey-Higher School of Economics Round XIV	National	both	18+	18+	4025	5498	†
Russian Federation	2007	Russian Karelia Survey in Pitkaranta	Community	both	25-64	25-64	176	276	*
Russian Federation	2007-2010	SAGE	National	both	50+	50+	1254	2251	†
Russian Federation	2015-2017	Ural Eye and Medical Study (UEMS)	Subnational	both	40+	40+	2580	3319	*
Rwanda	2000	DHS	National	both		18-49		7589	†
Rwanda	2005	DHS	National	both		18-49		4381	†
Rwanda	2010	DHS	National	both	18-59	18-49	5536	5559	†
Rwanda	2012	STEPS	National	both	18-64	18-64	2486	3997	†
Rwanda	2014-2015	DHS	National	both	18-59	18-49	5550	5433	†
Saint Kitts and Nevis	2007	STEPS	Subnational	both	25-64	25-64	510	852	†
Saint Lucia	1991-1994	Cooper et al., Am J Public Health 87(2):160-68, 1997	Community	urban	25-100	25-100	491	593	*
Saint Lucia	2012	STEPS	National	both	25-64	25-64	586	938	†
Samoa	1991	McGarvey, Pac Health Dialog 8(1):157-62, 2001	National	both	25+	25+	346	381	*
Samoa	1993	McGarvey, Pac Health Dialog 8(1):157-62, 2001	National	both	27+	27+	284	336	*
Samoa	1995	McGarvey, Pac Health Dialog 8(1):157-62, 2001	National	both	29+	29+	152	157	*
Samoa	2002	STEPS	National	both	25-64	25-64	1181	1334	†
Samoa	2010	Samoan Genome-Wide Association Study	National	both	24-65	24-65	1402	2061	*
Samoa	2013	STEPS	National	both	18-64	18-64	605	918	†
Sao Tome and Principe	2008-2009	DHS	National	both	18-59	18-49	1896	1961	†
Sao Tome and Principe	2009	STEPS	National	both	25-64	25-64	998	1286	†
Saudi Arabia	1985-1988	National Nutrition Survey	National	both	19-75	19-75	2311	3057	*
Saudi Arabia	1989-1994	National Nutrition Survey	National	both	18-40	18-40	2481	3294	*
Saudi Arabia	1990-1993	National Epidemiological Household Survey	National	both	18-60	18-60	4882	4509	*
Saudi Arabia	1990-1993	Saudi National Survey	National	both	30-70	30-70	1612	1648	*
Saudi Arabia	1992-1995	Saudi Health Information Survey	National	both	18-50	18-50	2812	5223	*
Saudi Arabia	1995	National Household Survey	National	both	20-70	20-70	7121	7074	*
Saudi Arabia	1995-2000	National Epidemiological Health Survey	National	both	30-70	30-70	8215	9008	*
Saudi Arabia	2004-2005	Al-Baghli et al., Saudi Med J 29(9):1319-25, 2008	Subnational	both	30+	30+	97254	97254	*
Saudi Arabia	2005	El Mouzan et al., Ann Saudi Med 30(3):203-8, 2010	National	both	18	18	417	471	*
Saudi Arabia	2005	STEPS	National	both	18-64	18-64	2245	2345	*
Saudi Arabia	2007	Gulf Cooperation Council World Health Survey	National	both	18+	18+	4854	3610	*

Country	Data years	Survey/study name/citation	Level of representative-ness	Rural, urban or both	Age range as used for global analysis		Sample size as used for global analysis		Note
					Male	Female	Male	Female	
Saudi Arabia	2009-2010	Arab Teens Lifestyle Study (ATLS)	Subnational	urban	18-19	18-19	349	244	*
Saudi Arabia	2011-2012	Jeeluna Study- National Assessment of the Health Needs of Adolescents in Saudi Arabia	National	both	18-19	18-19	1423	971	*
Saudi Arabia	2011-2013	Jeddah City Study	Community	urban	18+	18+	565	683	*
Saudi Arabia	2013	Saudi Health Information Survey	National	both	18+	18+	4694	4948	*
Senegal	1984	Maire et al., Rev Epidemiol Sante Publique 40:252-58, 1992	National	rural		18-45		1628	1*
Senegal	1986	Astagneau et al., J Hypertens 10(9):1095-101, 1992	Community	urban	18+	18+	651	707	*
Senegal	1986	Maire et al., Rev Epidemiol Sante Publique 40:252-58, 1992	Community	urban		18-45		616	*
Senegal	1992-1993	DHS	National	both		20-49		2713	†
Senegal	2003	Holdsworth et al., Int J Obes Relat Metab Disord 28(12):1561-68, 2004	Community	urban		20-49		301	*
Senegal	2005	DHS	National	both		18-49		3450	†
Senegal	2010-2011	DHS	National	both	18-59	18-49	3943	4704	†
Serbia	1988-1989	MONICA, Novi Sad	Community	urban	25-64	25-64	778	791	†
Serbia	1994-1995	MONICA, Novi Sad	Community	urban	25-64	25-64	600	670	†
Serbia	2000	Health Status, Health Needs and Utilization of Health Care of the Population of Serbia	National	both	18+	18+	4279	5375	*
Serbia	2006	The 2006 National Health Survey for the Population of Serbia	National	both	18+	18+	6766	7445	*
Serbia	2013	The National Health Survey of the Republic of Serbia, 2013	National	both	18+	18+	6262	7202	*
Serbia	2013-2014	Stay Fit for Lifelong Health; the Prevalence of Lifestyle Health Conditions in Serbian Population	National	urban	18-65		1366		*
Seychelles	1989	Seychelles Heart Survey I	National	both	25-64	25-64	513	568	*
Seychelles	1994	Seychelles Heart Survey II	National	both	25-64	25-64	497	557	*
Seychelles	2004	Seychelles Heart Survey III	National	both	25-64	25-64	568	687	*
Seychelles	2013-2014	Seychelles Heart Survey IV	National	both	25-64	25-64	531	699	*
Sierra Leone	2008	DHS	National	both		18-49		2964	†
Sierra Leone	2009	STEPS	National	both	25-64	25-64	2200	2319	†
Sierra Leone	2013	DHS	National	both	18-59	18-49	6126	6407	†
Singapore	1982-1985	Thyroid Heart Study	National	both	18+	18+	1030	990	1*
Singapore	1992	National Health Survey 1992	National	both	18-64	18-64	1743	1704	*
Singapore	1993-1995	NUH Heart Study	National	both	26-89	26-89	498	484	*
Singapore	1998	National Health Survey 1998	National	both	18-69	18-69	2284	2265	*
Singapore	2004	National Health Survey 2004	National	both	18-74	18-74	2059	2095	*
Singapore	2004-2007	Combined follow up of Singapore Cardiovascular Cohort study and Singapore Prospective study	National	both	24+	24+	2471	2686	*
Singapore	2009	Social Isolation, Health and Lifestyles Survey (SIHLS) 2009	National	both	60+	60+	2038	2382	*
Singapore	2009-2011	The Singapore Chinese Eye Study	Community	both	40-80	40-80	1652	1679	*
Singapore	2012-2013	Singapore Health Study 2012	National	both	18-79	18-79	956	1026	*
Slovakia	1993	Countrywide Integrated Noncommunicable Diseases Intervention (CINDI) Programme	National	both	18-64	18-64	762	1217	*
Slovakia	1998	Countrywide Integrated Noncommunicable Diseases Intervention (CINDI) Programme	National	both	18-64	18-64	857	1046	*
Slovakia	2003	Countrywide Integrated Noncommunicable Diseases Intervention (CINDI) Programme	National	both	18-64	18-64	622	867	*
Slovakia	2008	Countrywide Integrated Noncommunicable Diseases Intervention (CINDI) Programme	National	both	18-64	18-64	391	561	*
Slovakia	2011-2012	European Health Examination Survey	National	both	18-64	18-64	884	1080	*
Slovenia	1994	Analysis of Children's Development in Slovenia (ACDSi)	National	both	18	18	110	116	*
Slovenia	2004	Analysis of Children's Development in Slovenia (ACDSi)	National	both	18-19	18-19	229	172	*
Slovenia	2014	Analysis of Children's Development in Slovenia (ACDSi)	National	both	18-19	18-19	143	126	*
Slovenia	2014	the SLOFIT monitoring system	National	both	18-21	18-21	6460	6410	*
Solomon Islands	2004	A genetic-ecological study of the risk factors for lifestyle-related diseases in Oceanian populations	Community	both	18-79	18-79	197	204	*
Solomon Islands	2006	STEPS	Subnational	both	18-64	18-64	990	1307	†
Solomon Islands	2009-2010	Furusawa et al., N Z Med J 124(1333):17-28, 2011	Subnational	both	18+	18+	175	272	*
Solomon Islands	2015	STEPS	National	both	18-69	18-69	816	978	†
South Africa	1989	Temple et al., Ethn Dis 11(3):431-7, 2001	Community	both	18+	18+	457	614	*
South Africa	1990	Steyn et al., East Afr Med J 75(1):35-40, 1998	Community	urban	18-64	18-64	292	373	*
South Africa	1996	Temple et al., Ethn Dis 11(3):431-7, 2001	Community	both	18+	18+	302	406	*
South Africa	1998	DHS	National	both	18+	18+	4985	7105	†
South Africa	2002	The 1st South African National Youth Risk Behaviour Survey	National	both	18	18	475	420	*
South Africa	2002-2003	SASPI	Community	rural	35+	35+	80	275	*
South Africa	2003	DHS	National	both	18+	18+	2825	4121	†
South Africa	2003-2004	Africa Centre Biomeasure Survey	Community	rural	25-49	25-49	778	1693	*
South Africa	2004-2006	Li et al., Curationis 30(4):79-87, 2007	Community	both	18-40	18-40	334	270	*
South Africa	2007-2008	SAGE	National	both	50+	50+	1541	2058	†
South Africa	2008	National Income Dynamics Study Wave I	National	both	18+	18+	4811	7322	†
South Africa	2008	The 2nd South African National Youth Risk Behaviour Survey	National	both	18	18	526	431	*
South Africa	2008-2009	Cape Town Bellville South Cohort Study - Baseline evaluation I	Community	urban	18+	18+	220	715	*

Country	Data years	Survey/study name/citation	Level of representative-ness	Rural, urban or both	Age range as used for global analysis		Sample size as used for global analysis		Note
					Male	Female	Male	Female	
South Africa	2010	Africa Centre Biomeasure Survey	Community	rural	18+	18+	2271	5718	*
South Africa	2010-2011	National Income Dynamics Study Wave II	National	both	18+	18+	5331	7839	†
South Africa	2012	National Income Dynamics Study Wave III	National	both	18+	18+	6458	9628	†
South Africa	2012	South African National Health and Nutrition Examination Survey	National	both	18+	18+	2274	4320	*
South Africa	2014-2015	Health and Aging in Africa: A Longitudinal Study of an INDEPTH Community in South Africa (HAALSI)	Community	rural	40+	40+	2141	2502	†
South Korea	1986	INTERSALT	Community	urban	20-59	20-59	100	98	†
South Korea	1990	Korean National Blood Pressure Survey	National	both	30+	30+	9734	12620	*
South Korea	1992-1993	Park et al., Diabetes Res Clin Pract 34 Suppl:S65-72, 1996	Subnational	both	30-89	30+	1077	1392	*
South Korea	1997-1998	National Anthropometric Survey in Korean Children and Adolescents	National	both	18-19	18-19	2488	2443	*
South Korea	1998	Korea National Health and Nutrition Examination Survey	National	both	18+	18+	3741	4488	*
South Korea	1999	The South Korean Conscription Database	National	both	19		401721		*
South Korea	2000	The South Korean Conscription Database	National	both	19		402758		*
South Korea	2001	Kim et al., Br J Psychiatry 185:102-7, 2004	Community	both	65+	65+	300	432	*
South Korea	2001	The South Korean Conscription Database	National	both	19		398653		*
South Korea	2001	Korea National Health and Nutrition Examination Survey	National	both	18+	18+	2974	3763	*
South Korea	2002	The South Korean Conscription Database	National	both	19		367024		*
South Korea	2002-2003	Korean National Health Insurance	National	both	40+	40+	2979612	2470515	*
South Korea	2003	The South Korean Conscription Database	National	both	19		329626		*
South Korea	2004	The South Korean Conscription Database	National	both	19		323001		*
South Korea	2004-2005	Korean National Health Insurance	National	both	40+	40+	3586415	3244400	*
South Korea	2005	The South Korean Conscription Database	National	both	19		313378		*
South Korea	2005	Korea National Health and Nutrition Examination Survey	National	both	18+	18+	2392	3157	*
South Korea	2005	National Anthropometric Survey in Korean Children and Adolescents	National	both	18-19	18-19	3629	3273	*
South Korea	2006	The South Korean Conscription Database	National	both	19		302587		*
South Korea	2006-2007	Korean National Health Insurance	National	both	40+	40+	4325472	4348260	*
South Korea	2007	The South Korean Conscription Database	National	both	19		312795		*
South Korea	2007	Korea National Health and Nutrition Examination Survey	National	both	18+	18+	1276	1756	*
South Korea	2008	The South Korean Conscription Database	National	both	19		312919		*
South Korea	2008	Korea National Health and Nutrition Examination Survey	National	both	18+	18+	2875	3943	*
South Korea	2008-2009	Korean National Health Insurance	National	both	40+	40+	5480812	5733446	*
South Korea	2009	The South Korean Conscription Database	National	both	19		324818		*
South Korea	2009	Korea National Health and Nutrition Examination Survey	National	both	18+	18+	3313	4266	*
South Korea	2009	Korea National School Health Examination Survey (KNSHES)	National	both	18-20	18-20	6177	5182	*
South Korea	2010	The South Korean Conscription Database	National	both	19		347249		*
South Korea	2010	Korea National Health and Nutrition Examination Survey	National	both	18+	18+	2755	3585	*
South Korea	2010	Korea National School Health Examination Survey (KNSHES)	National	both	18-20	18-20	2130	954	*
South Korea	2010-2011	Korean National Health Insurance	National	both	40+	40+	6639239	7094118	*
South Korea	2011	The South Korean Conscription Database	National	both	19		364982		*
South Korea	2011	Korea National Health and Nutrition Examination Survey	National	both	18+	18+	2677	3561	*
South Korea	2011	Korea National School Health Examination Survey (KNSHES)	National	both	18-20	18-20	2597	1874	*
South Korea	2012	The South Korean Conscription Database	National	both	19		361009		*
South Korea	2012	Korea National Health and Nutrition Examination Survey	National	both	18+	18+	2529	3481	*
South Korea	2012	Korea National School Health Examination Survey (KNSHES)	National	both	18-20	18-20	1282	1066	*
South Korea	2012-2013	Korean National Health Insurance	National	both	40+	40+	7220230	7744568	*
South Korea	2013	The South Korean Conscription Database	National	both	19		363914		*
South Korea	2013	Korea National Health and Nutrition Examination Survey	National	both	18+	18+	2513	3309	*
South Korea	2013	Korea National School Health Examination Survey (KNSHES)	National	both	18-20	18-20	1267	1106	*
South Korea	2014	The South Korean Conscription Database	National	both	19		363597		*
South Korea	2014	Korea National Health and Nutrition Examination Survey	National	both	18+	18+	2409	3274	*
South Korea	2014	Korea National School Health Examination Survey (KNSHES)	National	both	18-20	18-20	1246	1201	*
South Korea	2014-2015	Korean National Health Insurance	National	both	40+	40+	7832947	8318037	*
South Korea	2015	The South Korean Conscription Database	National	both	19		350518		*
South Korea	2015	Korea National Health and Nutrition Examination Survey	National	both	18+	18+	2495	3168	*
South Korea	2015	Korea National School Health Examination Survey (KNSHES)	National	both	18-20	18-20	1538	1595	*
South Korea	2016	The South Korean Conscription Database	National	both	19		339410		*
South Korea	2016	Korea National Health and Nutrition Examination Survey	National	both	18+	18+	2677	3470	*
South Korea	2016	Korea National School Health Examination Survey (KNSHES)	National	both	18-20	18-20	1461	1482	*
South Korea	2017	The South Korean Conscription Database	National	both	19		323457		*
Spain	1985	INTERSALT, Manresa	Community	urban	20-59	20-59	100	100	†

Country	Data years	Survey/study name/citation	Level of representative-ness	Rural, urban or both	Age range as used for global analysis		Sample size as used for global analysis		Note
					Male	Female	Male	Female	
Spain	1986	INTERSALT, Torrejo	Community	urban	20-59	20-59	100	100	†
Spain	1986-1988	MONICA, Catalonia	Subnational	both	25-64	25-64	1251	1271	†
Spain	1989	Cardiovascular Risk Factors Study in Catalonia	Subnational	both	18+	18+	315	359	*
Spain	1989-1994	SEEDO	Subnational	both	25-60	25-60	2533	2855	*
Spain	1990	Banegas et al., Hypertension 32(6):998-1002, 1998	National	both	35-65	35-65	810	1203	*
Spain	1990-1992	MONICA, Catalonia	Subnational	both	25-64	25-64	1719	1191	†
Spain	1990-2000	SEEDO	Subnational	both	25-60	25-60	4707	5178	*
Spain	1991-1993	Encuesta de Factores de Riesgo Cardiovascular en la Región de Murcia (Cardiovascular Risk Factors Survey)	Subnational	both	18-69	18-69	1512	1562	*
Spain	1992	CINDI	Subnational	both	25-64	25-64	1194	1454	*
Spain	1992	ENCAT	Community	both	18-80	18-80	786	952	*
Spain	1994-1995	Encuesta de Nutrición y Salud Comunidad Valenciana 1994-95 (ENCV)	Subnational	urban	18+	18+	767	892	*
Spain	1994-1996	MONICA, Catalonia	Subnational	both	25-64	25-64	1800	1628	†
Spain	1996	Guía Study	Community	urban	30+	30+	305	384	*
Spain	1996-2002	Castells et al., J Epidemiol Community Health 60(4):316-21, 2006	Community	urban		50-69		26963	*
Spain	1997	Soriguer et al., Eur J Epidemiol 19(1):33-40, 2004	Community	rural	18-65	18-65	613	613	*
Spain	1998-2000	EnKID study	National	both	18-24	18-24	595	858	*
Spain	1999-2000	ENIB	Community	both	20-60	20-60	498	702	*
Spain	1999-2000	Factores de riesgo en las islas Baleares: Estudio CORSAIB	Subnational	both	35-74	35-74	811	865	*
Spain	2000-2001	Regidor et al., J Hum Hypertens 20(1):73-82, 2006	National	both	60+	60+	1318	2281	*
Spain	2000-2001	EUREYE Study	Subnational	both	65+	65+	273	324	*
Spain	2000-2005	CDC of the Canary Islands	Subnational	both	18-75	18-75	2878	3719	*
Spain	2001-2002	Catalan Health Interview Survey	Subnational	both	18-74	18-74	597	744	*
Spain	2001-2003	DIabetes, Nutrición y Obesidad en la población adulta de la Región de Murcia (DINO)	Subnational	both	20+	20+	715	828	*
Spain	2002-2003	ENCAT	Community	both	18-80	18-80	712	813	*
Spain	2003	The European Male Ageing Study	Community	both	40+		405		*
Spain	2003-2005	Registre Gironi del Cor (REGICOR)	Subnational	both	35-79	35-79	2934	3253	*
Spain	2004	Vioque J et al., Obesity 16:664-70, 2008	Community	urban	24+	24+	87	115	*
Spain	2004	Cardiovascular Risk Study in Castilla y León (RECCyL)	Subnational	both	18+	18+	1833	2027	*
Spain	2004-2006	PREVICTUS	National	both	60+	60+	3185	3629	*
Spain	2007-2009	Harmonizing Equation of Risk in Mediterraneon countries Extremadura (HERMEX)	Subnational	both	25-79	25-79	1298	1498	*
Spain	2008	The European Male Ageing Study	Community	both	40+		272		*
Spain	2008-2010	National Study of Nutrition and Cardiovascular Risk (ENRICA)	National	both	18+	18+	5756	6397	*
Spain	2009	Cardiovascular Risk Study in Castilla y León (RECCyL)	Subnational	both	20+	20+	1315	1590	*
Spain	2013	ANIBES Study	National	both	18-75	18-75	787	822	*
Spain	2014	Cardiovascular Risk Study in Castilla y León (RECCyL)	Subnational	both	20+	20+	1215	1475	*
Spain	2015	National Study of Nutrition and Cardiovascular Risk (ENRICA)	National	both	65+	65+	711	770	*
Sri Lanka	2003	Wijewardene et al., Ceylon Med J 50:62-70, 2005	Subnational	both	30-65	30-65	275	296	*
Sri Lanka	2003	Wijewardene et al., Ceylon Med J 50:62-70, 2005	Subnational	both	30-65	30-65	139	192	*
Sri Lanka	2003	Wijewardene et al., Ceylon Med J 50:62-70, 2005	Subnational	both	30-65	30-65	1891	2410	*
Sri Lanka	2003	Wijewardene et al., Ceylon Med J 50:62-70, 2005	Subnational	both	30-65	30-65	387	457	*
Sri Lanka	2006	STEPS	National	both	18-64	18-64	5682	5922	†
Sri Lanka	2006-2007	DHS	National	both		18-49		12539	*
Sri Lanka	2014	STEPS	National	both	18-69	18-69	1863	2893	†
Sudan (former)	2005	STEPS	Subnational	both	25-64	25-64	626	881	*
Sudan (former)	2016	STEPS	National	both	18-69	18-69	2661	4544	†
Suriname	2013-2015	The Healthy Life in Suriname Study (HELISUR)	Subnational	urban	18-70	18-70	424	722	*
Swaziland	2006-2007	DHS	National	both	18-49	18-49	3252	3970	†
Swaziland	2014	STEPS	National	both	18-69	18-69	986	1848	†
Sweden	1982	The Swedish Conscription Database	National	both	18		28487		1*
Sweden	1983	The Swedish Conscription Database	National	both	18		28020		1*
Sweden	1984	The Swedish Conscription Database	National	both	18		17397		1*
Sweden	1985	MONICA Gothenburg	Community	urban	25-64	25-64	666	702	†
Sweden	1985	The Swedish Conscription Database	National	both	18		9237		*
Sweden	1985-1989	Västerbotten Intervention Project	Subnational	both	25-64	25-64	1676	1554	*
Sweden	1985-1996	EPIC Umea	Subnational	both	24-72	24-72	12359	13217	*
Sweden	1986	MONICA Northern Sweden	Subnational	both	25-64	25-64	610	598	†
Sweden	1986	The Swedish Conscription Database	National	both	18		29739		*
Sweden	1987	The Swedish Conscription Database	National	both	18		33855		*
Sweden	1988	The Swedish Conscription Database	National	both	18		30456		*

Country	Data years	Survey/study name/citation	Level of representative-ness	Rural, urban or both	Age range as used for global analysis		Sample size as used for global analysis		Note
					Male	Female	Male	Female	
Sweden	1989	The Swedish Conscription Database	National	both	18		33678		*
Sweden	1990	MONICA Northern Sweden	Subnational	both	25-64	25-64	570	589	†
Sweden	1990	MONICA Gothenburg	Community	urban	25-64	25-64	775	775	†
Sweden	1990	The Swedish Conscription Database	National	both	18		35633		*
Sweden	1990-1992	Västerbotten Intervention Project	Subnational	both	25-64	25-64	7263	7804	*
Sweden	1991	The Swedish Conscription Database	National	both	18		35851		*
Sweden	1991-1995	Uppsala Longitudinal Study of Adult Men	Community	both	70		1215		*
Sweden	1991-1996	Malmö Diet and Cancer	Community	urban	45-73	45-73	12096	18293	*
Sweden	1992	The Swedish Conscription Database	National	both	18		33496		*
Sweden	1992-1993	Population Study of Women in Gothenburg	Community	urban		62-84		802	*
Sweden	1993	The Swedish Conscription Database	National	both	18		32442		*
Sweden	1993-1995	Västerbotten Intervention Project	Subnational	both	25-64	25-64	9804	10727	*
Sweden	1994	Helicobacter Pylori	Community	urban	56-65	56-65	170	217	*
Sweden	1994	MONICA Northern Sweden	Subnational	both	25-74	25-74	686	682	†
Sweden	1994	The Swedish Conscription Database	National	both	18		30614		*
Sweden	1994-1996	Kungsholmen Project	Community	urban	75+	75+	160	160	*
Sweden	1995	MONICA Gothenburg	Community	urban	25-64	25-64	745	867	†
Sweden	1995	The Swedish Conscription Database	National	both	18		30299		*
Sweden	1996	The Swedish Conscription Database	National	both	18		27042		*
Sweden	1996-1998	Västerbotten Intervention Project	Subnational	both	25-64	25-64	8327	8893	*
Sweden	1997	The Swedish Conscription Database	National	both	18		27230		*
Sweden	1997-2001	Uppsala Longitudinal Study of Adult Men	Community	both	77		783		*
Sweden	1998	The Swedish Conscription Database	National	both	18		34111		*
Sweden	1998-2001	The Kalixanda study	Community	both	20+	20+	508	483	*
Sweden	1999	MONICA Northern Sweden	Subnational	both	25-74	25-74	660	657	*
Sweden	1999	The Swedish Conscription Database	National	both	18		30143		*
Sweden	1999-2003	Västerbotten Intervention Project	Subnational	both	25-64	25-64	6354	6384	*
Sweden	2000	The Swedish Conscription Database	National	both	18		23619		*
Sweden	2000-2001	H70 Study	Community	urban	70	70	242	270	*
Sweden	2001	The Swedish Conscription Database	National	both	18		23717		*
Sweden	2001-2003	Uppsala Longitudinal Study of Adult Men	Community	both	82		511		*
Sweden	2001-2004	Swedish INTERGENE Cohort Study	Subnational	both	24-76	24-76	1694	1906	*
Sweden	2001-2004	Prospective Investigation of the Vasculature in Uppsala Seniors (PIVUS)	Community	both	70	70	507	509	*
Sweden	2002	The Swedish Conscription Database	National	both	18		20070		*
Sweden	2003	The European Male Ageing Study	Community	both	40+		396		*
Sweden	2003	The Swedish Conscription Database	National	both	18		25062		*
Sweden	2003-2004	Welin et al., BMC Public Health 8:403, 2008	Community	urban	50	50	595	655	*
Sweden	2003-2004	Welin et al., BMC Public Health 8:403, 2008	Community	urban	60		667		*
Sweden	2004	MONICA Northern Sweden	Subnational	both	26-75	26-75	722	706	*
Sweden	2004	The Swedish Conscription Database	National	both	18		23784		*
Sweden	2004-2005	European Youth Heart Study (EYHS) II	Subnational	urban	18-21	18-21	70	111	*
Sweden	2004-2005	Population Study of Women in Gothenburg	Community	urban		38-50		494	*
Sweden	2005	The Swedish Conscription Database	National	both	18		20818		*
Sweden	2005-2006	H70 Study	Community	urban	75	75	320	422	*
Sweden	2006-2009	Prospective Investigation of the Vasculature in Uppsala Seniors (PIVUS)	Community	both	75	75	407	419	*
Sweden	2008	The European Male Ageing Study	Community	both	40+		353		*
Sweden	2009	MONICA Northern Sweden	Subnational	both	25-74	25-74	629	622	*
Sweden	2011-2014	EpiHealth	National	both	45-75	45-75	4731	6054	*
Sweden	2014	MONICA Northern Sweden	Subnational	both	25-74	25-74	551	602	*
Switzerland	1984-1986	The Swiss MONICA Study Wave I	Subnational	both	25-74	25-74	1744	1689	†
Switzerland	1988-1989	The Swiss MONICA Study Wave II	Subnational	both	25-74	25-74	1778	1684	†
Switzerland	1992-1993	The Swiss MONICA Study Wave III	Subnational	both	25-74	25-74	1577	1672	†
Switzerland	2003-2006	Cohorte Lausannoise	Community	urban	35-75	35-75	3186	3536	*
Switzerland	2004	The Swiss Conscription Database	National	both	18-20		20491		*
Switzerland	2005	The Swiss Conscription Database	National	both	18-20		32131		*
Switzerland	2006	The Swiss Conscription Database	National	both	18-20		34530		*
Switzerland	2007	The Swiss Conscription Database	National	both	18-20		36194		*
Switzerland	2007-2012	Bus Santé Study	Subnational	urban	20-80	20-80	1884	1911	*
Switzerland	2008	The Swiss Conscription Database	National	both	18-20		34497		*

Country	Data years	Survey/study name/citation	Level of representative-ness	Rural, urban or both	Age range as used for global analysis		Sample size as used for global analysis		Note
					Male	Female	Male	Female	
Switzerland	2009	The Swiss Conscription Database	National	both	18-20		34896		*
Switzerland	2009-2012	Cohorte Lausannoise	Community	urban	40-81	40-81	2176	2494	*
Switzerland	2010	The Swiss Conscription Database	National	both	18-20		33815		*
Switzerland	2011	The Swiss Conscription Database	National	both	18-20		34495		*
Switzerland	2012	The Swiss Conscription Database	National	both	18-20		33590		*
Switzerland	2013	The Swiss Conscription Database	National	both	18-20		32882		*
Switzerland	2014	The Swiss Conscription Database	National	both	18-20		32687		*
Switzerland	2014-2017	Cohorte Lausannoise	Community	urban	45-87	45-87	2008	2473	*
Switzerland	2015	The Swiss Conscription Database	National	both	18-20		32604		*
Syrian Arab Republic	2002	National survey on non-communicable diseases and factors affecting their development	National	both	18-64	18-64	3155	4045	*
Taiwan	1985	INTERSALT	Community	rural	20-59	20-59	89	92	†
Taiwan	1989-1991	Chiu et al., J Gerontol A Biol Sci Med Sci 55(11):M684-90, 2000	Subnational	both	65+	65+	1322	1308	*
Taiwan	1993-1994	The Kinmen Neurological Disorders Survey	Community	urban	50+	50+	672	593	*
Taiwan	1993-1996	Nutrition and Health Survey in Taiwan 1993-1996	National	both	18+	18+	1506	1704	†
Taiwan	1999-2000	Nutrition and Health Survey in Taiwan 1999-2000	National	both	65+	65+	1271	1202	†
Taiwan	2004-2005	TCHS	Community	urban	40+	40+	1147	1212	*
Taiwan	2005-2008	Nutrition and Health Survey in Taiwan 2005-2008	National	both	19+	19+	1311	1355	†
Taiwan	2007	Taiwanese Survey on Hypertension, Hyperglycemia and Hyperlipidemia	National	both	20+	20+	2155	2490	*
Taiwan	2013-2016	Nutrition and Health Survey in Taiwan	National	both	18+	18+	1546	1622	†
Tajikistan	2003	Micronutrient Status Survey	National	both		18-49		1806	*
Tajikistan	2012	DHS	National	both		18-49		7724	†
Tajikistan	2016	STEPS	National	both	18-69	18-69	1091	1553	†
Tanzania	1991-1992	DHS	National	both		20-49		4039	†
Tanzania	1996	DHS	National	both		20-49		3512	†
Tanzania	1996-1997	Aspray et al., Trans R Soc Trop Med Hyg 94:637-44, 2000	Community	both	18+	18+	368	442	*
Tanzania	1998-1999	Bovet et al., Int J Epidemiol 31(1):240-7, 2002	Community	urban	25-64	25-64	3593	5646	*
Tanzania	2004-2005	DHS	National	both		18-49		7854	†
Tanzania	2010	DHS	National	both		18-49		7746	†
Tanzania	2011	STEPS	Subnational	both	25-64	25-64	1008	1517	†
Tanzania	2012	STEPS	National	both	25-64	25-64	2581	2827	†
Tanzania	2014	Dar es Salaam Urban Cohort Hypertension Study	Community	urban	40+	40+	965	1266	*
Tanzania	2015-2016	DHS	National	both		18-49		10376	†
Thailand	1987	INCLEN	Community	rural	35-65		244		*
Thailand	1989	INCLEN	Community	both	35-65		416		*
Thailand	1991	Thailand National Health Examination Survey I	National	both	18+	18+	5771	8140	*
Thailand	1995	The Fourth National Nutrition Survey of Thailand	National	both	20-60	20-60	1405	3631	*
Thailand	1997	Thailand National Health Examination Survey II	National	both	18-59	18-59	1183	2023	*
Thailand	2000	InterASIA	National	both	35+	35+	2092	3211	*
Thailand	2003-2004	The Fifth National Nutrition Survey of Thailand	National	both	19+	19+	1961	3366	*
Thailand	2004	Thailand National Health Examination Survey III	National	both	18+	18+	18442	19862	*
Thailand	2009	Thailand National Health Examination Survey IV	National	both	18+	18+	9262	10247	*
Timor-Leste	2009-2010	DHS	National	both		18-49		10009	†
Timor-Leste	2009-2010	Timor-Leste Eye Health Survey	Subnational	both	40+	40+	244	247	*
Timor-Leste	2014	STEPS	National	both	18-69	18-69	1048	1437	†
Timor-Leste	2016	DHS	National	both	18-59	18-49	3903	9826	†
Togo	1998	DHS	National	both		20-49		3114	†
Togo	2010	STEPS	National	both	18-64	18-64	1897	1961	†
Togo	2013-2014	DHS	National	both		18-49		3877	†
Togo	2014	Impact evaluation of a cash transfer program in North Togo	Subnational	rural		20-65		3588	*
Tokelau	2005	STEPS	National	both	18-64	18-64	241	267	†
Tokelau	2014	STEPS	National	both	18-64	18-64	261	276	†
Tonga	2004	STEPS	National	both	18-64	18-64	390	546	†
Tonga	2005-2007	Pacific Obesity Prevention in Communities - Ma'alahi Youth Project	Subnational	rural	18-19	18-19	105	137	*
Tonga	2007-2008	Pacific Obesity Prevention in Communities - Ma'alahi Youth Project	Subnational	rural	18-22	18-22	144	218	*
Tonga	2011	STEPS	National	both	18-64	18-64	878	1401	†
Trinidad and Tobago	1985	INTERSALT	Community	urban	20-59	20-59	84	92	†
Trinidad and Tobago	2001	Adult Survey	National	rural	25+	25+	198	267	*
Tunisia	1996-1997	Ariana Healthy Project 1997	Community	both	35-65	35-65	2645	2704	*
Tunisia	1996-1997	Tunisian National Nutrition Survey 1996-1997	National	both	18+	18+	1397	2674	*

Country	Data years	Survey/study name/citation	Level of representative-ness	Rural, urban or both	Age range as used for global analysis		Sample size as used for global analysis		Note
					Male	Female	Male	Female	
Tunisia	2005	Aounallah et al., Public Health 12(1):98, 2012	National	both	18-19	18-19	451	583	*
Tunisia	2005	Tunisian National Survey	National	both	35-71	35-71	3350	4442	*
Tunisia	2009-2010	ObeMaghreb	Subnational	urban	18-49	18-49	998	696	*
Turkey	1990	Turkish Adult Risk Factor Study	National	both	20+	20+	1331	1366	*
Turkey	1993	DHS	National	both		20-49		2294	†
Turkey	1995	Turkish Adult Risk Factor Study	National	both	25+	25+	855	879	*
Turkey	1998	DHS	National	both		20-49		2210	†
Turkey	1998	Turkish Adult Risk Factor Study	National	both	28+	28+	870	906	*
Turkey	1998-1999	Erem et al., Diabetes Res Clin Pract 54(3):203-8, 2001	Community	urban	20+	20+	1324	1322	*
Turkey	2000	MDHS	Subnational	urban		18-49		1420	*
Turkey	2000	Turkish Adult Risk Factor Study	National	both	30+	30+	883	936	*
Turkey	2000-2002	The Healthy Nutrition for Healthy Heart Study	National	both	25-84	25-84	4718	10631	*
Turkey	2001	Yumuk et al., Diabetes Res Clin Pract 70(2):151-58, 2005	Community	urban	20+	20+	1042	1789	*
Turkey	2001-2002	Turkish Adult Risk Factor Study	National	both	32+	32+	1098	1210	*
Turkey	2002	Onal et al., Blood Press 13(1):31-6, 2004	Subnational	urban	25+	25+	67	355	*
Turkey	2003	DHS	National	both		20-49		2934	†
Turkey	2003	Prevalence, awareness, treatment and control of hypertension in Turkey in 2003	National	both	18+	18+	1988	2847	*
Turkey	2003-2004	Turkish Adult Risk Factor Study	National	both	34+	34+	1104	1140	*
Turkey	2003-2005	Prevalence of prehypertension and associated risk factors among Turkish adults: Trabzon Hypertension Study	Subnational	both	20+	20+	2205	2601	*
Turkey	2004	Nationally Representative Cross-sectional Survey	National	both	20+	20+	2110	2154	*
Turkey	2005-2006	Turkish Adult Risk Factor Study	National	both	33+	33+	1029	1088	*
Turkey	2007	National Household survey	National	both	20-85	20-85	2263	1842	*
Turkey	2007-2008	Turkish Adult Risk Factor Study	National	both	35+	35+	1101	1133	*
Turkey	2009-2010	Turkish Adult Risk Factor Study	National	both	37+	37+	466	507	*
Turkey	2009-2012	Prevalence of diabetes and associated risk factors among adult population in Trabzon city	Subnational	both	20+	20+	1570	2124	*
Turkey	2011	Chronic Diseases and Risk Factors Survey in Turkey	National	both	18+	18+	7326	8205	*
Turkey	2012-2013	Turkish Adult Risk Factor Study	National	both	37+	37+	1012	1087	*
Turkey	2014-2015	Turkish Adult Risk Factor Study	National	both	44+	44+	437	484	*
Turkey	2017	STEPS	National	both	18+	18+	2230	3349	†
Turkmenistan	2000	DHS	National	both		18-49		2084	*
Turkmenistan	2013	STEPS	National	both	18-64	18-64	1879	2741	†
Uganda	1995	DHS	National	both		20-49		2831	†
Uganda	2000-2001	DHS	National	both		18-49		4983	†
Uganda	2006	DHS	National	both	18-54	18-49	2091	2139	†
Uganda	2011	DHS	National	both	18-54	18-49	1988	2119	†
Uganda	2011-2012	The Prevalence and Distribution of Non-communicable Diseases and Their Risk Factors in Kasese District, Uganda	Subnational	both	25-79	25-79	232	182	*
Uganda	2014	STEPS	National	both	18-69	18-69	1560	2120	†
Uganda	2016	DHS	National	both	18-54	18-54	4404	4594	†
Ukraine	2002	National Micronutrient Survey	National	both		18-50		816	*
United Arab Emirates	1989-1990	el Mugamer et al., J Trop Med Hyg 98(6):407-15, 1995	Community	both	20+	20+	123	199	*
United Arab Emirates	1999-2000	Emirates National Diabetes and Coronary Artery Disease Risk Factor Study	National	both	20-80	20-80	2822	3743	*
United Arab Emirates	2000-2001	Carter et al., J Health Popul Nutr 22(1):75-83, 2004	Community	both		20-79		521	*
United Arab Emirates	2009	Gulf Cooperation Council World Health Survey	National	both	18+	18+	603	645	*
United Kingdom	1982	MRC National Survey of Health and Development	National	both	36-37	36-37	1632	1648	1†
United Kingdom	1984-1986	Scottish Heart Health Survey	Subnational	both	40-59	40-59	4364	4465	†
United Kingdom	1985	INTERSALT, Birmingham	Community	urban	20-59	20-59	100	100	†
United Kingdom	1985	INTERSALT, Wales	Community	urban	20-59	20-59	100	99	†
United Kingdom	1985-1986	INTERSALT, Belfast	Community	urban	20-59	20-59	99	100	†
United Kingdom	1986-1987	Dietary and Nutritional Survey of British Adults 1986-1987	National	both	18-64	18-64	1100	1124	†
United Kingdom	1986-1987	MONICA, Belfast	Subnational	both	25-64	25-64	1155	1185	†
United Kingdom	1987-1988	Edinburgh Artery Study	Community	urban	54-75	54-75	808	783	*
United Kingdom	1989	MRC National Survey of Health and Development	National	both	42-44	42-44	1617	1608	†
United Kingdom	1991	National Child Development Study (1958 British Cohort Study)	National	both	33	33	5426	5605	†
United Kingdom	1991-1992	Health Survey for England	National	both	18+	18+	1668	1830	†
United Kingdom	1991-1992	MONICA, Belfast	Subnational	both	25-64	25-64	998	996	†
United Kingdom	1992	MONICA, Glasgow	Community	urban	25-64	25-64	696	775	†
United Kingdom	1992-1993	Whickham Survey	Community	urban	35+	35+	676	784	*
United Kingdom	1992-1994	Edinburgh Artery Study	Community	urban	60-81	60-81	580	582	*
United Kingdom	1993	Health Survey for England	National	both	18+	18+	7225	8059	†

Country	Data years	Survey/study name/citation	Level of representative-ness	Rural, urban or both	Age range as used for global analysis		Sample size as used for global analysis		Note
					Male	Female	Male	Female	
United Kingdom	1993-1997	EPIC Norfolk	Subnational	both	40-79	40-79	11574	13995	*
United Kingdom	1993-2000	EPIC Oxford	Subnational	both	20-98	20-98	10851	37605	*
United Kingdom	1994	Health Survey for England	National	both	18+	18+	6601	7670	†
United Kingdom	1994-1995	National Diet and Nutrition Survey (NDNS)	National	both	65+	65+	701	687	†
United Kingdom	1995	Health Survey for England	National	both	18+	18+	6525	7483	†
United Kingdom	1995	MONICA, Glasgow	Community	urban	25-64	25-64	855	958	†
United Kingdom	1995	Scottish Health Survey (SHeS)	Subnational	both	18-64	18-64	3207	3903	†
United Kingdom	1996	British Cohort Study 1970	National	both	26	26	81	78	†
United Kingdom	1996	Health Survey for England	National	both	18+	18+	6751	7793	†
United Kingdom	1997	Health Survey for England	National	both	18+	18+	3580	4103	†
United Kingdom	1997	National Diet and Nutrition Survey (NDNS)	National	both	18	18	49	53	†
United Kingdom	1997-1999	INTERMAP, West Bromwich	Community	urban	40-59	40-59	141	138	†
United Kingdom	1998	Health Survey for England	National	both	18+	18+	6380	7494	†
United Kingdom	1998	Scottish Health Survey (SHeS)	Subnational	both	18-74	18-74	3506	4444	†
United Kingdom	1998-1999	INTERMAP, Belfast	Community	urban	40-59	40-59	125	97	†
United Kingdom	1998-2000	The British Regional Heart Study	National	urban	60-79		4138		*
United Kingdom	1999	Health Survey for England	National	both	18+	18+	3104	3574	†
United Kingdom	1999	MRC National Survey of Health and Development	National	both	53-54	53-54	1452	1496	†
United Kingdom	1999-2001	British Women's Heart and Health Study	National	both		60-79		3678	12*
United Kingdom	1999-2001	Edinburgh Artery Study	Community	urban	66-87	66-87	373	404	*
United Kingdom	1999-2004	Hertfordshire Cohort Study	Subnational	both	59-73	59-73	1571	1416	*
United Kingdom	2000	Health Survey for England	National	both	18+	18+	3254	3828	†
United Kingdom	2000-2001	National Diet and Nutrition Survey 2000-2001	National	both	19-64	19-64	807	973	†
United Kingdom	2001	Health Survey for England	National	both	18+	18+	6071	7195	†
United Kingdom	2002	Health Survey for England	National	both	18+	18+	3703	4506	†
United Kingdom	2003	The European Male Ageing Study	Community	both	40+		394		*
United Kingdom	2003	Health Survey for England	National	both	18+	18+	5787	6854	†
United Kingdom	2003	Scottish Health Survey (SHeS)	Subnational	both	18+	18+	2922	3583	†
United Kingdom	2004	Health Survey for England	National	both	18+	18+	2365	3049	†
United Kingdom	2004-2005	English Longitudinal Study of Ageing Wave 2 2004-2005	National	both	52+	52+	3255	3961	†
United Kingdom	2005	Health Survey for England	National	both	18+	18+	3768	4422	†
United Kingdom	2006	Health Survey for England	National	both	18+	18+	5359	6298	†
United Kingdom	2006-2010	MRC National Survey of Health and Development	National	both	60-65	60-65	1061	1156	†
United Kingdom	2007	Health Survey for England	National	both	18+	18+	2632	3122	†
United Kingdom	2008	The European Male Ageing Study	Community	both	40+		301		*
United Kingdom	2008	Health Survey for England	National	both	18+	18+	5659	6767	†
United Kingdom	2008	Scottish Health Survey (SHeS)	Subnational	both	18+	18+	2392	2950	†
United Kingdom	2008-2009	English Longitudinal Study of Ageing Wave 4 2008-2009	National	both	50+	50+	3539	4296	†
United Kingdom	2008-2012	National Diet and Nutrition Survey (NDNS)	National	both	18+	18+	869	1120	†
United Kingdom	2009	Health Survey for England	National	both	18+	18+	1790	2054	†
United Kingdom	2009	Scottish Health Survey (SHeS)	Subnational	both	18+	18+	2757	3380	†
United Kingdom	2010	Health Survey for England	National	both	18+	18+	3046	3737	†
United Kingdom	2010	Scottish Health Survey (SHeS)	Subnational	both	18+	18+	2616	3257	†
United Kingdom	2011	Health Survey for England	National	both	18+	18+	3098	3769	†
United Kingdom	2011	Scottish Health Survey (SHeS)	Subnational	both	18+	18+	2688	3311	†
United Kingdom	2012	Health Survey for England	National	both	18+	18+	3033	3687	†
United Kingdom	2012	Scottish Health Survey (SHeS)	Subnational	both	18+	18+	1835	2175	†
United Kingdom	2012-2013	English Longitudinal Study of Ageing Wave 6 2012-2013	National	both	50+	50+	3257	4015	†
United Kingdom	2013	Health Survey for England	National	both	18+	18+	3246	3882	†
United Kingdom	2013	Scottish Health Survey (SHeS)	Subnational	both	18+	18+	1781	2237	†
United Kingdom	2013-2014	National Diet and Nutrition Survey (NDNS)	National	both	18+	18+	499	767	†
United Kingdom	2014	Health Survey for England	National	both	18+	18+	3057	3700	†
United Kingdom	2014	Scottish Health Survey (SHeS)	Subnational	both	18+	18+	1727	2156	†
United Kingdom	2015	Health Survey for England	National	both	18+	18+	3000	3634	†
United Kingdom	2015	MRC National Survey of Health and Development	National	both	69-70	69-70	1040	1082	†
United Kingdom	2015	Scottish Health Survey (SHeS)	Subnational	both	18+	18+	1840	2151	†
United Kingdom	2016	Health Survey for England	National	both	18+	18+	2851	3479	†
United Kingdom	2016	Scottish Health Survey (SHeS)	Subnational	both	18+	18+	1577	1944	†
United States of America	1985-1986	Coronary Artery Risk Development in Young Adults (CARDIA)	Subnational	urban	18-30	18-30	2321	2775	*

Country	Data years	Survey/study name/citation	Level of representative-ness	Rural, urban or both	Age range as used for global analysis		Sample size as used for global analysis		Note
					Male	Female	Male	Female	
United States of America	1985-1986	INTERSALT, Chicago	Community	urban	20-59	20-59	97	99	†
United States of America	1985-1986	MONICA, Stanford	Subnational	urban	25-64	25-64	713	848	6†
United States of America	1985-1987	The Minnesota Heart Survey	Community	both	25-75	25-75	5220	2421	*
United States of America	1986	INTERSALT, Goodman	Community	urban	20-59	20-59	192	192	†
United States of America	1987-1988	The Bogalusa Heart Study	Community	rural	18-25	18-25	39	28	*
United States of America	1987-1988	Coronary Artery Risk Development in Young Adults (CARDIA)	Subnational	urban	20-32	20-32	2082	2506	*
United States of America	1987-1989	Atherosclerosis Risk in Communities Study	Subnational	both	44-66	44-66	5041	6213	*
United States of America	1988-1994	US NHANES III	National	both	18+	18+	8271	9097	†
United States of America	1989-1990	Cardiovascular Health Study	Subnational	both	65+	65+	2458	3318	*
United States of America	1989-1990	MONICA, Stanford	Subnational	urban	25-64	25-64	720	842	6†
United States of America	1990-1991	Coronary Artery Risk Development in Young Adults (CARDIA)	Subnational	urban	23-35	23-35	1945	2382	*
United States of America	1990-1991	Cardiovascular Health Study	Subnational	both	65+	65+	2070	2707	*
United States of America	1990-1992	Atherosclerosis Risk in Communities Study	Subnational	both	46-70	46-70	4537	5624	*
United States of America	1991-1992	Cardiovascular Health Study	Subnational	both	65+	65+	1919	2563	*
United States of America	1992-1993	Coronary Artery Risk Development in Young Adults (CARDIA)	Subnational	urban	25-37	25-37	1823	2163	*
United States of America	1992-1993	Cardiovascular Health Study	Subnational	both	65+	65+	248	419	*
United States of America	1992-1994	The Bogalusa Heart Study	Community	rural	18-25	18-25	53	34	*
United States of America	1993-1994	Cardiovascular Health Study	Subnational	both	65+	65+	1751	2471	*
United States of America	1993-1995	Atherosclerosis Risk in Communities Study	Subnational	both	48-73	48-73	4000	5015	*
United States of America	1993-1998	Women's Health Initiative - Observational Study	National	both		49-81		92697	*
United States of America	1994-1995	Cardiovascular Health Study	Subnational	both	65+	65+	1617	2354	*
United States of America	1995-1996	Coronary Artery Risk Development in Young Adults (CARDIA)	Subnational	urban	28-40	28-40	1739	2145	*
United States of America	1995-1996	Cardiovascular Health Study	Subnational	both	65+	65+	1478	2194	*
United States of America	1996	National Longitudinal Study of Adolescent Health Wave II	National	both	18-21	18-21	486	440	7†
United States of America	1996-1997	Cardiovascular Health Study	Subnational	both	65+	65+	1356	2043	*
United States of America	1996-1997	INTERMAP, Baltimore	Community	urban	40-59	40-59	146	134	†
United States of America	1996-1997	INTERMAP, Jackson	Community	urban	40-59	40-59	132	134	†
United States of America	1996-1997	INTERMAP, Pittsburgh	Community	urban	40-59	40-59	132	128	†
United States of America	1996-1997	Study of Women's Health Across the Nation	Subnational	both		40-55		3200	8†
United States of America	1996-1998	Atherosclerosis Risk in Communities Study	Subnational	both	50-75	50-75	3550	4485	*
United States of America	1996-1998	INTERMAP, Minneapolis	Community	urban	40-59	40-59	130	130	†
United States of America	1997-1998	INTERMAP, CC	Community	urban	40-59	40-59	271	276	†
United States of America	1997-1998	INTERMAP, Chicago	Community	urban	40-59	40-59	156	159	†
United States of America	1997-1998	Cardiovascular Health Study	Subnational	both	65+	65+	1172	1801	*
United States of America	1997-1999	Study of Women's Health Across the Nation	Subnational	both		40-55		2761	8†
United States of America	1998-1999	Cardiovascular Health Study	Subnational	both	65+	65+	1092	1684	*
United States of America	1998-2000	Study of Women's Health Across the Nation	Subnational	both		40-55		2596	8†
United States of America	1999-2000	US NHANES 1999-2000	National	both	18+	18+	2301	2319	†
United States of America	1999-2001	Study of Women's Health Across the Nation	Subnational	both		40-56		2507	8†
United States of America	2000-2001	Coronary Artery Risk Development in Young Adults (CARDIA)	Subnational	urban	33-45	33-45	1570	1949	*
United States of America	2000-2002	Study of Women's Health Across the Nation	Subnational	both		40-57		2441	8†
United States of America	2001-2002	National Longitudinal Study of Adolescent Health Wave III	National	both	18-28	18-28	2139	2443	7†
United States of America	2001-2002	US NHANES 2001-2002	National	both	18+	18+	2505	2418	†
United States of America	2003-2004	US NHANES 2003-2004	National	both	18+	18+	2499	2442	†
United States of America	2005-2006	Coronary Artery Risk Development in Young Adults (CARDIA)	Subnational	urban	38-50	38-50	1528	2000	*
United States of America	2005-2006	Cardiovascular Health Study	Subnational	both	65+	65+	375	684	*
United States of America	2005-2006	US NHANES 2005-2006	National	both	18+	18+	2507	2371	†
United States of America	2005-2006	National Social Life Health and Aging Project	National	both	57-85	57-85	1355	1435	9†
United States of America	2007-2008	US NHANES 2007-2008	National	both	18+	18+	2902	2923	†
United States of America	2008-2009	National Longitudinal Study of Adolescent Health Wave IV	National	both	24-34	24-34	2045	2472	7†
United States of America	2009-2010	US NHANES 2009-2010	National	both	18+	18+	3050	3166	†
United States of America	2010-2011	Coronary Artery Risk Development in Young Adults (CARDIA)	Subnational	urban	43-55	43-55	1513	1976	*
United States of America	2010-2011	National Social Life Health and Aging Project	National	both	36-99	36-99	1452	1736	9†
United States of America	2011-2012	US NHANES 2011-2012	National	both	18+	18+	2737	2732	†
United States of America	2011-2013	Atherosclerosis Risk in Communities Study	Subnational	both	67-90	67-90	1787	2431	*
United States of America	2013-2014	US NHANES 2013-2014	National	both	18+	18+	2791	2991	†
United States of America	2015-2016	US NHANES 2015-2016	National	both	18+	18+	2718	2875	†
United States of America	2015-2016	National Social Life Health and Aging Project	Community	both	24-99	24-99	1995	2490	9†
Uruguay	1999-2000	The Survey on Health, Well-Being, and Aging in Latin America and the Caribbean (SABE)	Community	urban	60+	60+	492	828	2†

Country	Data years	Survey/study name/citation	Level of representative-ness	Rural, urban or both	Age range as used for global analysis		Sample size as used for global analysis		Note
					Male	Female	Male	Female	
Uruguay	2006	STEPS	National	both	25-64	25-64	261	641	†
Uruguay	2011-2012	CESCAS Study	Community	urban	35-74	35-74	650	927	*
Uruguay	2012-2016	Genotype, Phenotype and Environment of Hypertension in Uruguay (GEFA-HT-UY)	Community	urban	19+	19+	124	189	*
Uzbekistan	1996	DHS	National	both		18-49		3499	†
Uzbekistan	2002	DHS	National	both	18-59	18-49	2062	4559	†
Uzbekistan	2014	STEPS	National	both	18-64	18-64	1533	2164	†
Vanuatu	1996	Second National Nutrition Survey	National	both		18-50		1353	*
Vanuatu	1998	Vanuatu Non-communicable Disease Survey	National	both	20-60	20-60	533	730	*
Vanuatu	2005	STEPS	Subnational	both	18-60	18-60	583	701	†
Vanuatu	2011	STEPS	National	both	25-64	25-64	2251	2183	†
Venezuela	1999-2001	Florez et al., Diabetes Res Clin Pract 69(1):63-77, 2005	Subnational	both	18+	18+	1134	2599	*
Venezuela	2000	Diaz et al., Invest Clin 46(2):111-19, 2005	Community	urban	60+	60+	42	59	*
Venezuela	2004-2005	CArdiovascular Risk factors Multiple Evaluation in Latin America (CARMELA)	Community	urban	25-64	25-64	713	1123	*
Venezuela	2005-2006	Brajkovich et al., Rev Ven Endoc Metab 4(3):31-32, 2006	Community	urban	20-65	20-65	205	439	*
Venezuela	2007-2008	Venezuelan Study of Metabolic Syndrome, Obesity and Lifestyle (VEMSOLS)	Community	urban	20+	20+	107	230	*
Venezuela	2008-2009	Venezuelan Study of Metabolic Syndrome, Obesity and Lifestyle (VEMSOLS)	Community	rural	20+	20+	51	89	*
Venezuela	2010-2011	Venezuelan Study of Metabolic Syndrome, Obesity and Lifestyle (VEMSOLS)	Community	urban	20+	20+	66	193	*
Venezuela	2015-2017	Cardio-Metabolic Health Venezuelan Study (EVESCAM)	National	both	20+	20+	1056	2346	*
Viet Nam	1987-1989	General Nutrition Survey	National	both	18-70	18-70	13776	17271	*
Viet Nam	1992-1993	Living Standard Survey	National	both	18+	18+	5620	6497	*
Viet Nam	1997-1998	Living Standard Survey	National	both	18+	18+	7506	8759	*
Viet Nam	2000	National Nutrition Survey	National	both	20+	20+	8985	9464	*
Viet Nam	2001-2002	Viet Nam National Health Survey 2001-2002	National	both	18+	18+	42413	48739	*
Viet Nam	2001-2003	The National Epidemiological Survey on Hypertension and Its Risk Factors (North)	Subnational	both	25-74	25-74	2386	3604	*
Viet Nam	2003-2004	The Survey on Heart Failure and Its Risk Factors	Subnational	both	25-74	25-74	1853	2636	*
Viet Nam	2004	Cuong et al., Eur J Clin Nutr 61(5):673-81, 2007	Community	urban	20-60	20-60	717	771	*
Viet Nam	2004	The Hypertension Management Programme in Rural Communes (Hanoi)	Community	rural	25-74	25-74	855	1288	*
Viet Nam	2005	STEPS Bavi district	Subnational	rural	25-64	25-64	987	997	*
Viet Nam	2005	National Adult Overweight Survey	National	both	25-64	25-64	8474	8725	*
Viet Nam	2005	The Survey on Non-Communicable Disease Risk Factors	Subnational	both	25-74	25-74	1136	1220	*
Viet Nam	2005	Non-communicable disease risk factors in Ho Chi Minh City	Community	urban	25-64	25-64	908	1063	*
Viet Nam	2006	The Hypertension Management Programme in Rural Communes (Bavi)	Community	rural	25-74	25-74	395	643	*
Viet Nam	2006-2008	The National Epidemiological Survey on Hypertension and Its Risk Factors (South)	Subnational	both	25-74	25-74	1310	2078	*
Viet Nam	2007	The Hypertension Management Programme in Rural Communes (Phu Phuong)	Community	rural	25-74	25-74	364	616	*
Viet Nam	2008-2009	The Survey on Diabetes and Its Risk Factors	Subnational	both	25-74	25-74	751	1329	*
Viet Nam	2009	The Hypertension Management Programme in Rural Communes (Phu Cuong)	Community	rural	25-74	25-74	362	677	*
Viet Nam	2009	STEPS	National	both	25-64	25-64	6738	7805	†
Viet Nam	2009-2010	General Nutrition Survey	National	both	20+	20+	10810	11729	*
Viet Nam	2009-2010	Vietnam National Nutrition Survey 2009-2010	National	both	18+	18+	11529	12115	*
Viet Nam	2012	National Survey of Diabetes in Viet Nam	National	both	30-69	30-69	5319	5855	*
Viet Nam	2015	STEPS	National	both	18-69	18-69	1316	1721	†
Yemen	1997	DHS	National	both		18-49		5123	*
Yemen	2005-2006	Yemen Household Budget Survey 2005-2006	National	both	18+	18+	1555	1735	*
Yemen	2007-2009	Hypertension and Diabetes in Yemen (HYDY)	National	both	18-70	18-70	4690	4828	*
Yemen	2013	DHS	National	both		18-49		18900	†
Zambia	1992	DHS	National	both		20-49		2829	†
Zambia	1996	DHS	National	both		20-49		3485	†
Zambia	2001-2002	DHS	National	both		18-49		5800	†
Zambia	2003	Kelly et al., Am J Clin Nut 88(4):1010-17, 2008	Community	urban	18-74	18-84	132	217	*
Zambia	2007	DHS	National	both		18-49		5456	†
Zambia	2008	STEPS	Subnational	urban	25+	25+	626	1214	†
Zambia	2013-2014	DHS	National	both		18-49		12813	†
Zambia	2017	STEPS	National	both	18-69	18-69	1565	2439	†
Zimbabwe	1985-1986	INTERSALT	Community	urban	20-59	20-59	100	95	†
Zimbabwe	1991	Zinyowera et al., Cent Afr J Med 40(2):33-8, 1994	Community	both	18+	18+	775	734	*
Zimbabwe	1994	DHS	National	both		20-49		1776	†
Zimbabwe	1995	Mufunda et al., J Hum Hypertens 14(1):65-73, 2000	Community	urban	25+	25+	384	391	*
Zimbabwe	1999	DHS	National	both		18-49		4324	†
Zimbabwe	2005	STEPS	National	both	25-64	25-64	569	1808	*

Country	Data years	Survey/study name/citation	Level of representative-ness	Rural, urban or both	Age range as used for global analysis		Sample size as used for global analysis		Note
					Male	Female	Male	Female	
Zimbabwe	2005-2006	DHS	National	both		18-49		7017	†
Zimbabwe	2010-2011	DHS	National	both	18-54	18-49	6284	7216	†
Zimbabwe	2015	DHS	National	both	18-54	18-49	7087	8093	†

* Data should be requested from study investigators.

† Data available from www.ncdrisc.org.

1. National studies for the 3 years prior to 1985 were assigned to 1985 so that they can inform the estimates in countries with slightly earlier national data.
2. The bibliographic citation for this data source is: Pelaez, Martha, Alberto Palloni, Cecilia Albala, Juan C. Alfonso, Roberto Ham-Chande, Anselm Hennis, Maria Lucia Lebrao, Esther Lesn-Diaz, Edith Pantelides, and Omar Prats. SABE - SURVEY ON HEALTH, WELL-BEING, AND AGING IN LATIN AMERICA AND THE CARIBBEAN, 2000 [Computer file]. ICPSR version. Washington, D.C.: Pan American Health Organization/World Health Organization (PAHO/WHO) [producers], 2004. Ann Arbor, MI: Inter-university Consortium for Political and Social Research [distributor], 2005.
3. This research uses data from China Health and Nutrition Survey (CHNS). We thank the National Institute of Nutrition and Food Safety, China Center for Disease Control and Prevention, Carolina Population Center (5 R24 HD050924), the University of North Carolina at Chapel Hill, the NIH (R01-HD30880, DK056350, R24HD050924, and R01-HD38700) and the Fogarty International Center, NIH for financial support for the CHNS data collection and analysis files from 1989 to 2011 and future surveys, and the China-Japan Friendship Hospital, Ministry of Health for support for CHNS 2009.
4. The Longitudinal Aging Study Amsterdam is supported by a grant from the Netherlands Ministry of Health Welfare and Sports, Directorate of Long-Term Care.
5. The bibliographic citation for this data source is: Palloni, Alberto, Ana Luisa Davila, and Melba Sanchez-Ayendez. Puerto Rican Elderly: Health Conditions (PREHCO) Project, 2002-2003, 2006-2007. ICPSR34596-v1. Ann Arbor, MI: Inter-university Consortium for Political and Social Research[distributor], 2013-09-13. doi:10.3886/ICPSR34596.v1.
6. We thank Prof Stephen Fortmann for data from the Stanford Five-City Project.
7. This research uses data from Add Health, a program project designed by J. Richard Udry, Peter S. Bearman, and Kathleen Mullan Harris, and funded by a grant P01-HD31921 from the Eunice Kennedy Shriver National Institute of Child Health and Human Development, with cooperative funding from 17 other agencies. Special acknowledgment is due Ronald R. Rindfuss and Barbara Entwisle for assistance in the original design. Persons interested in obtaining data files from Add Health should contact Add Health, Carolina Population Center, 123 W. Franklin Street, Chapel Hill, NC 27516-2524 (addhealth@unc.edu). No direct support was received from grant P01-HD31921 for this analysis.
8. The bibliographic citation for this data source is: Sutton-Tyrrell, Kim, Faith Selzer, MaryFran Sowers, Robert Neer, Lynda Powell, Ellen Gold, Gail Greendale, Gerson Weiss, Karen Matthews, and Sonja McKinlay. Study of Women's Health Across the Nation (SWAN), 1996-1997: Baseline Dataset. ICPSR28762-v2. Ann Arbor, MI: Inter-university Consortium for Political and Social Research[distributor], 2014-02-04. http://doi.org/10.3886/ICPSR28762.v2.
9. The bibliographic citation for this data source is: Waite, Linda J., Kathleen Cagney, William Dale, Elbert Huang, Edward O. Laumann, Martha McClintock, Colm A. O'Muircheartaigh, L. Phillip Schumm, and Benjamin Cornwell. National Social Life, Health, and Aging Project (NSHAP): Wave 2 and Partner Data Collection. ICPSR34921-v1. Ann Arbor, MI: Inter-university Consortium for Political and Social Research [distributor], 2014-04-29. https://doi.org/10.3886/ICPSR34921.v1.
10. We thank Jelena Jakovljevic, Henrique Loyola, Harry Rutter, Joop van Raaij, Stephen Whiting and Ivo Rakovac for data from the COSI Project.
11. The Older Persons in Jamaica Study was funded by the National Health Fund, Jamaica.
12. The British Women’s Heart and Health Study is supported by the British Heart Foundation (PG/13/66/30442). British Women’s Heart and Health Study data are available to bona fide researchers for research purposes. Please refer to the BWHHS data sharing policy at http://www.ucl.ac.uk/british-womens-heart health-study.

Supplementary Table 2. List of analysis regions and super-regions, and countries in each region.

Super-region	Region
**Sub-Saharan Africa (48)**	**Central Africa (6)**: Angola, Central African Republic, Congo, DR Congo, Equatorial Guinea, Gabon
	**East Africa (17)**: Burundi, Comoros, Djibouti, Eritrea, Ethiopia, Kenya, Madagascar, Malawi, Mauritius, Mozambique, Rwanda, Seychelles, Somalia, Sudan (former), Tanzania, Uganda, Zambia
	**Southern Africa (6)**: Botswana, Lesotho, Namibia, South Africa, Swaziland, Zimbabwe
	**West Africa (19)**: Benin, Burkina Faso, Cabo Verde, Cameroon, Chad, Cote d'Ivoire, Gambia, Ghana, Guinea, Guinea Bissau, Liberia, Mali, Mauritania, Niger, Nigeria, Sao Tome and Principe, Senegal, Sierra Leone, Togo
**Central Asia, Middle East and north Africa (28)**	**Central Asia (9)**: Armenia, Azerbaijan, Georgia, Kazakhstan, Kyrgyzstan, Mongolia, Tajikistan, Turkmenistan, Uzbekistan
	**Middle East and north Africa (19)**: Algeria, Bahrain, Egypt, Iran, Iraq, Jordan, Kuwait, Lebanon, Libya, Morocco, Occupied Palestinian Territory, Oman, Qatar, Saudi Arabia, Syrian Arab Republic, Tunisia, Turkey, United Arab Emirates, Yemen
**South Asia (6)**	**South Asia (6)**: Afghanistan, Bangladesh, Bhutan, India, Nepal, Pakistan
**East and southeast Asia (16)**	**East Asia (4)**: China, China (Hong Kong SAR), North Korea, Taiwan
	**Southeast Asia (12)**: Brunei Darussalam, Cambodia, Indonesia, Lao PDR, Malaysia, Maldives, Myanmar, Philippines, Sri Lanka, Thailand, Timor-Leste, Viet Nam
**Oceania (17)**	**Polynesia and Micronesia (13)**: American Samoa, Cook Islands, French Polynesia, Kiribati, Marshall Islands, Micronesia (Federated States of), Nauru, Niue, Palau, Samoa, Tokelau, Tonga, Tuvalu
	**Melanesia (4):** Fiji, Papua New Guinea, Solomon Islands, Vanuatu
**High-income Asia Pacific (3)**	**High-income Asia Pacific (3)**: Japan, Singapore, South Korea
**Latin America and the Caribbean (35)**	**Andean Latin America (3)**: Bolivia, Ecuador, Peru
	**The Caribbean (18)**: Antigua and Barbuda, Bahamas, Barbados, Belize, Bermuda, Cuba, Dominica, Dominican Republic, Grenada, Guyana, Haiti, Jamaica, Puerto Rico, Saint Kitts and Nevis, Saint Lucia, Saint Vincent and the Grenadines, Suriname, Trinidad and Tobago
	**Central Latin America (9)**: Colombia, Costa Rica, El Salvador, Guatemala, Honduras, Mexico, Nicaragua, Panama, Venezuela
	**Southern Latin America (5)**: Argentina, Brazil, Chile, Paraguay, Uruguay
**High-income western (27)**	**High-income English-speaking countries* (6)**: Australia, Canada, Ireland, New Zealand, United Kingdom, United States of America
	**Northwestern Europe (12)**: Austria, Belgium, Denmark, Finland, Germany, Greenland, Iceland, Luxembourg, Netherlands, Norway, Sweden, Switzerland
	**Southwestern Europe (9)**: Andorra, Cyprus, France, Greece, Israel, Italy, Malta, Portugal, Spain
**Central and eastern Europe (20)**	**Central Europe (13)**: Albania, Bosnia and Herzegovina, Bulgaria, Croatia, Czech Republic, Hungary, Macedonia (TFYR), Montenegro, Poland, Romania, Serbia, Slovakia, Slovenia
	**Eastern Europe (7)**: Belarus, Estonia, Latvia, Lithuania, Moldova, Russian Federation, Ukraine

*Although high-income English-speaking countries are geographically separated, they exhibit remarkably similar trends in cardiometabolic risk factors and outcomes.[5,17-19] They were therefore grouped together so that the statistical model shares information amongst them more than it does with other countries that are geographically closer but epidemiologically more distinct.

We did not have data on population by age group for American Samoa, Bermuda, Greenland, and Tokelau. Country-specific estimates were made but were not used in calculation of regional and global means because the latter requires weighting by age-specific population.

Supplementary Table 3. Age-standardised national, rural and urban mean body-mass index (BMI) in 1985 and 2017.

Country	Sex	Age-standardised mean BMI in 1985 (kg/m^2)			Age-standardised mean BMI in 2017 (kg/m^2)		
		National	Rural	Urban	National	Rural	Urban
Afghanistan	Men	20.2 (17.8-22.7)	19.7 (17.2-22.2)	22.4 (20.0-25.0)	22.8 (20.3-25.3)	22.5 (20.0-25.0)	23.6 (21.0-26.1)
	Women	20.6 (18.4-22.8)	20.1 (17.8-22.4)	23.2 (20.9-25.4)	24.4 (23.3-25.4)	23.6 (22.5-24.8)	26.3 (25.1-27.4)
Albania	Men	25.2 (23.9-26.5)	25.0 (23.7-26.4)	25.4 (24.0-26.7)	27.0 (26.0-27.9)	26.9 (25.9-27.9)	27.0 (26.0-28.0)
	Women	26.0 (24.1-27.9)	26.1 (24.1-28.1)	25.9 (23.9-27.8)	26.0 (24.8-27.2)	26.2 (24.8-27.5)	25.9 (24.6-27.2)
Algeria	Men	22.1 (20.8-23.3)	21.8 (20.5-23.1)	22.3 (21.0-23.6)	25.1 (24.5-25.7)	24.8 (24.1-25.4)	25.2 (24.6-25.9)
	Women	24.0 (22.2-25.7)	23.3 (21.4-25.1)	24.8 (22.9-26.6)	27.4 (26.7-28.0)	27.0 (26.3-27.8)	27.5 (26.7-28.2)
American Samoa	Men	33.7 (32.7-34.7)	32.6 (31.7-33.5)	34.0 (32.9-35.1)	34.3 (33.0-35.6)	34.6 (33.1-35.9)	34.2 (32.9-35.6)
	Women	34.3 (33.1-35.6)	34.1 (33.0-35.2)	34.4 (33.0-35.8)	35.3 (33.7-36.9)	35.0 (33.1-36.9)	35.4 (33.7-37.1)
Andorra	Men	25.0 (22.5-27.4)	25.3 (22.8-27.7)	25.0 (22.5-27.4)	26.8 (24.4-29.2)	26.8 (24.3-29.2)	26.8 (24.3-29.3)
	Women	25.2 (22.0-28.4)	25.4 (22.2-28.7)	25.2 (22.0-28.4)	25.3 (22.1-28.6)	25.2 (21.9-28.5)	25.3 (22.1-28.6)
Angola	Men	20.5 (17.9-23.1)	20.2 (17.6-22.9)	21.4 (18.8-24.0)	22.6 (20.0-25.1)	22.0 (19.4-24.6)	23.2 (20.6-25.9)
	Women	21.3 (18.0-24.6)	20.9 (17.6-24.3)	22.7 (19.3-26.0)	24.4 (21.2-27.7)	23.3 (20.0-26.7)	25.7 (22.4-29.1)
Antigua and Barbuda	Men	23.6 (21.2-26.0)	23.2 (20.8-25.7)	24.2 (21.8-26.8)	26.2 (23.8-28.6)	26.0 (23.6-28.4)	26.7 (24.3-29.2)
	Women	24.5 (21.3-27.6)	24.0 (20.8-27.3)	25.4 (22.1-28.6)	28.1 (25.1-31.3)	28.0 (24.9-31.3)	28.5 (25.3-31.8)
Argentina	Men	25.2 (24.2-26.3)	24.3 (23.0-25.7)	25.4 (24.3-26.4)	27.8 (26.9-28.7)	26.9 (25.8-28.1)	27.9 (27.0-28.8)
	Women	24.3 (23.0-25.7)	23.3 (21.5-25.2)	24.5 (23.2-25.9)	27.3 (26.1-28.4)	27.0 (25.5-28.5)	27.3 (26.1-28.5)
Armenia	Men	23.4 (22.0-24.8)	22.8 (21.4-24.3)	23.7 (22.2-25.1)	25.7 (24.9-26.3)	25.3 (24.5-26.2)	25.8 (25.1-26.6)
	Women	25.4 (23.7-27.1)	25.2 (23.5-27.0)	25.5 (23.8-27.3)	27.1 (26.4-27.7)	27.5 (26.8-28.3)	26.8 (26.1-27.5)
Australia	Men	25.3 (24.8-25.8)	25.6 (24.9-26.3)	25.3 (24.8-25.8)	27.5 (27.0-28.1)	27.9 (27.4-28.5)	27.5 (27.0-28.1)
	Women	24.5 (23.9-25.1)	24.7 (23.8-25.6)	24.4 (23.8-25.0)	26.7 (26.0-27.3)	27.3 (26.6-27.9)	26.6 (26.0-27.3)
Austria	Men	24.4 (23.8-24.9)	24.7 (23.9-25.5)	24.2 (23.5-24.8)	26.7 (26.1-27.4)	27.0 (26.2-27.8)	26.6 (25.9-27.3)
	Women	23.7 (22.5-24.7)	24.3 (22.9-25.6)	23.3 (22.1-24.5)	24.0 (22.9-25.1)	24.5 (23.2-25.8)	23.7 (22.6-24.9)
Azerbaijan	Men	24.0 (22.8-25.2)	23.6 (22.3-24.9)	24.3 (23.1-25.6)	26.2 (25.6-26.8)	26.0 (25.4-26.6)	26.4 (25.8-27.0)
	Women	25.2 (23.5-26.8)	24.2 (22.5-25.9)	26.0 (24.3-27.7)	27.4 (26.7-28.1)	26.9 (26.1-27.7)	27.7 (26.9-28.5)
Bahamas	Men	24.1 (21.7-26.5)	23.3 (20.8-25.7)	24.3 (21.8-26.7)	26.7 (24.3-29.0)	26.1 (23.6-28.5)	26.8 (24.4-29.2)
	Women	25.1 (21.9-28.3)	24.0 (20.8-27.3)	25.4 (22.1-28.6)	28.4 (25.3-31.5)	28.0 (24.8-31.2)	28.5 (25.3-31.6)
Bahrain	Men	24.3 (23.2-25.4)	23.7 (22.4-25.1)	24.4 (23.3-25.5)	27.7 (26.6-28.8)	27.0 (25.7-28.3)	27.7 (26.7-28.9)
	Women	25.4 (23.9-26.8)	24.0 (22.1-25.8)	25.6 (24.1-27.1)	28.9 (27.5-30.4)	28.4 (26.6-30.2)	29.0 (27.6-30.5)
Bangladesh	Men	19.4 (18.3-20.5)	18.8 (17.7-20.0)	21.8 (20.6-23.1)	21.6 (21.0-22.2)	21.1 (20.5-21.7)	22.4 (21.7-23.2)
	Women	18.2 (16.9-19.7)	17.7 (16.3-19.2)	20.6 (19.2-22.1)	22.5 (21.9-23.1)	21.7 (21.1-22.4)	23.9 (23.2-24.6)
Barbados	Men	24.2 (23.3-25.1)	24.1 (23.0-25.1)	24.5 (23.4-25.6)	26.5 (25.5-27.4)	26.4 (25.4-27.5)	26.5 (25.5-27.5)
	Women	27.1 (25.9-28.3)	26.8 (25.5-28.0)	27.7 (26.3-29.0)	29.4 (28.2-30.6)	29.5 (28.2-30.7)	29.3 (28.0-30.5)
Belarus	Men	24.7 (23.2-26.3)	24.7 (23.0-26.4)	24.7 (23.1-26.4)	26.5 (25.8-27.2)	26.2 (25.4-26.9)	26.6 (25.9-27.4)
	Women	26.2 (23.8-28.4)	26.7 (24.2-29.1)	25.8 (23.4-28.1)	26.4 (25.6-27.2)	27.3 (26.5-28.2)	26.1 (25.2-26.9)
Belgium	Men	24.8 (24.3-25.2)	25.0 (24.4-25.5)	24.8 (24.3-25.2)	26.4 (25.7-27.1)	26.4 (25.6-27.2)	26.4 (25.7-27.1)
	Women	24.8 (24.2-25.3)	25.1 (24.4-25.7)	24.8 (24.2-25.3)	25.4 (24.5-26.3)	25.3 (24.2-26.4)	25.4 (24.5-26.4)
Belize	Men	24.9 (23.4-26.3)	24.4 (22.8-26.0)	25.4 (23.9-27.0)	27.6 (26.3-28.8)	27.3 (25.9-28.6)	27.9 (26.6-29.3)
	Women	26.8 (24.8-28.7)	26.2 (24.1-28.2)	27.5 (25.4-29.5)	30.5 (29.0-32.0)	30.4 (28.6-32.1)	30.8 (29.0-32.5)
Benin	Men	20.7 (19.4-22.1)	20.3 (18.9-21.7)	21.7 (20.3-23.1)	22.7 (22.0-23.3)	22.1 (21.3-22.8)	23.4 (22.7-24.1)
	Women	20.6 (19.1-22.0)	20.1 (18.5-21.6)	21.8 (20.3-23.3)	24.3 (23.5-25.0)	23.2 (22.5-24.0)	25.5 (24.7-26.3)
Bermuda	Men	24.3 (21.8-26.8)	na*	24.3 (21.8-26.8)	26.7 (24.3-29.2)	na*	26.7 (24.3-29.2)
	Women	25.4 (22.2-28.6)	na*	25.4 (22.2-28.6)	28.5 (25.3-31.6)	na*	28.5 (25.3-31.6)
Bhutan	Men	20.6 (19.1-22.1)	20.3 (18.7-21.8)	23.1 (21.5-24.6)	23.5 (22.8-24.3)	23.1 (22.2-23.9)	24.2 (23.3-25.2)
	Women	20.7 (18.4-22.9)	20.3 (18.0-22.6)	23.0 (20.8-25.3)	24.6 (23.7-25.5)	23.8 (22.7-24.9)	25.9 (24.7-27.0)

Country	Sex	Age-standardised mean BMI in 1985 (kg/m^2)			Age-standardised mean BMI in 2017 (kg/m^2)		
		National	Rural	Urban	National	Rural	Urban
Bolivia	Men	23.3 (21.0-25.5)	22.9 (20.6-25.2)	23.6 (21.3-25.9)	26.1 (23.9-28.4)	25.3 (23.1-27.5)	26.5 (24.2-28.8)
	Women	23.8 (22.3-25.3)	23.0 (21.4-24.5)	24.6 (23.1-26.1)	27.9 (26.5-29.3)	27.0 (25.6-28.5)	28.3 (26.8-29.7)
Bosnia and Herzegovina	Men	24.9 (23.5-26.2)	24.8 (23.4-26.2)	25.0 (23.6-26.4)	26.8 (25.7-27.8)	26.7 (25.7-27.8)	26.8 (25.7-27.9)
	Women	25.5 (23.5-27.4)	25.7 (23.7-27.7)	25.1 (23.1-27.0)	25.7 (24.4-27.1)	26.0 (24.6-27.5)	25.3 (23.9-26.8)
Botswana	Men	20.6 (19.2-22.0)	20.3 (18.8-21.7)	21.4 (20.0-22.9)	22.6 (21.9-23.4)	21.9 (20.9-22.8)	23.2 (22.3-24.0)
	Women	24.2 (22.2-26.2)	23.7 (21.5-25.8)	25.6 (23.5-27.7)	26.2 (25.3-27.1)	25.0 (23.8-26.2)	27.0 (26.0-28.2)
Brazil	Men	23.3 (22.5-24.0)	22.2 (21.4-23.0)	23.7 (22.9-24.5)	26.2 (25.7-26.7)	25.0 (24.3-25.6)	26.4 (25.9-26.9)
	Women	24.0 (23.2-25.0)	23.1 (22.1-24.1)	24.4 (23.5-25.4)	26.9 (26.2-27.5)	26.4 (25.7-27.2)	26.9 (26.3-27.6)
Brunei Darussalam	Men	24.0 (22.5-25.4)	23.7 (22.1-25.1)	24.2 (22.7-25.7)	27.1 (26.4-27.7)	26.6 (26.0-27.3)	27.2 (26.5-27.9)
	Women	23.6 (21.4-25.6)	22.9 (20.7-25.0)	24.0 (21.8-26.1)	27.4 (26.6-28.1)	27.0 (26.1-27.8)	27.5 (26.7-28.2)
Bulgaria	Men	25.0 (23.8-26.4)	24.9 (23.5-26.4)	25.1 (23.8-26.5)	27.0 (25.8-28.1)	27.0 (25.7-28.3)	27.0 (25.7-28.2)
	Women	25.2 (23.4-27.1)	25.5 (23.4-27.6)	25.0 (23.1-26.9)	25.6 (24.1-27.1)	26.0 (24.2-27.8)	25.5 (23.9-27.1)
Burkina Faso	Men	20.2 (18.8-21.5)	20.0 (18.6-21.4)	21.4 (19.9-22.9)	22.2 (21.4-23.0)	21.8 (21.0-22.6)	23.1 (22.1-24.1)
	Women	19.9 (18.7-21.2)	19.6 (18.4-20.9)	22.0 (20.7-23.3)	22.2 (21.4-23.1)	21.1 (20.2-22.0)	24.7 (23.7-25.7)
Burundi	Men	20.2 (17.7-22.6)	20.1 (17.6-22.5)	21.2 (18.7-23.6)	22.1 (19.8-24.5)	22.0 (19.6-24.3)	23.2 (20.8-25.6)
	Women	19.6 (17.5-21.7)	19.5 (17.4-21.6)	21.7 (19.5-23.8)	21.1 (20.3-22.0)	20.7 (19.8-21.6)	24.0 (23.0-24.9)
Cabo Verde	Men	21.4 (20.0-22.8)	20.9 (19.4-22.4)	22.3 (20.9-23.8)	23.6 (22.6-24.7)	22.7 (21.6-23.9)	24.1 (23.0-25.2)
	Women	22.2 (20.4-24.2)	21.8 (19.8-23.7)	23.3 (21.3-25.2)	25.6 (24.2-27.0)	24.2 (22.7-25.7)	26.3 (24.8-27.8)
Cambodia	Men	19.8 (18.4-21.2)	19.7 (18.3-21.1)	20.6 (19.1-22.1)	22.1 (21.2-23.1)	21.9 (21.0-22.9)	22.9 (21.7-24.1)
	Women	19.2 (17.6-20.9)	19.0 (17.4-20.7)	20.4 (18.7-22.1)	22.5 (21.7-23.3)	22.3 (21.4-23.1)	23.2 (22.3-24.1)
Cameroon	Men	21.6 (20.3-23.0)	21.2 (19.7-22.6)	22.5 (21.2-23.8)	23.5 (22.6-24.4)	22.9 (21.9-23.9)	24.1 (23.1-25.0)
	Women	22.0 (20.5-23.5)	21.1 (19.5-22.7)	23.5 (22.0-25.1)	24.8 (23.9-25.7)	23.1 (22.1-24.0)	26.2 (25.3-27.2)
Canada	Men	25.4 (24.8-26.0)	25.6 (24.9-26.3)	25.4 (24.7-26.0)	27.4 (26.8-28.0)	27.6 (26.8-28.3)	27.4 (26.8-27.9)
	Women	24.5 (23.7-25.2)	24.9 (24.0-25.8)	24.3 (23.5-25.1)	26.7 (26.0-27.4)	27.0 (26.0-28.0)	26.6 (25.9-27.3)
Central African Republic	Men	20.0 (18.4-21.6)	19.6 (17.8-21.3)	20.8 (19.0-22.5)	21.8 (20.5-23.1)	21.3 (19.9-22.7)	22.5 (21.0-24.0)
	Women	20.5 (19.0-22.0)	20.1 (18.6-21.7)	21.3 (19.7-22.9)	23.3 (21.7-24.9)	22.6 (20.8-24.3)	24.5 (22.8-26.2)
Chad	Men	20.4 (18.5-22.2)	20.1 (18.2-22.0)	21.4 (19.6-23.2)	22.2 (20.6-23.9)	22.0 (20.2-23.7)	23.1 (21.5-24.7)
	Women	19.6 (18.1-21.2)	19.3 (17.7-20.8)	21.1 (19.4-22.6)	21.8 (20.9-22.7)	21.2 (20.3-22.2)	23.9 (23.0-24.9)
Chile	Men	25.3 (24.2-26.3)	24.9 (23.7-26.1)	25.3 (24.2-26.4)	28.1 (27.5-28.7)	27.8 (27.2-28.5)	28.1 (27.5-28.7)
	Women	25.8 (24.2-27.4)	25.3 (23.5-27.1)	25.9 (24.2-27.5)	28.8 (28.1-29.5)	29.4 (28.6-30.2)	28.8 (28.0-29.5)
China	Men	21.1 (20.8-21.5)	20.9 (20.5-21.3)	22.0 (21.6-22.4)	24.4 (23.9-24.9)	23.9 (23.4-24.4)	24.7 (24.2-25.3)
	Women	21.4 (21.0-21.8)	21.2 (20.7-21.6)	22.2 (21.7-22.7)	23.6 (23.0-24.3)	23.4 (22.8-24.1)	23.7 (23.1-24.4)
China (Hong Kong SAR)	Men	22.4 (21.4-23.3)	21.5 (20.2-22.8)	22.4 (21.5-23.4)	24.8 (23.6-26.1)	na*	24.8 (23.6-26.1)
	Women	22.0 (20.8-23.2)	21.2 (19.4-23.0)	22.1 (20.8-23.3)	24.2 (22.2-26.2)	na*	24.2 (22.2-26.2)
Colombia	Men	22.8 (21.7-23.9)	22.0 (20.9-23.2)	23.2 (22.0-24.4)	25.7 (25.0-26.4)	24.8 (24.0-25.5)	26.0 (25.2-26.7)
	Women	23.8 (22.5-25.0)	23.3 (22.0-24.5)	24.0 (22.7-25.4)	26.7 (25.8-27.7)	26.6 (25.6-27.5)	26.7 (25.8-27.7)
Comoros	Men	21.3 (19.9-22.7)	21.0 (19.5-22.5)	22.1 (20.5-23.6)	23.3 (22.4-24.2)	22.9 (22.0-23.9)	24.2 (23.1-25.3)
	Women	21.3 (19.7-22.9)	21.0 (19.3-22.6)	22.2 (20.5-23.9)	25.5 (24.6-26.4)	25.0 (24.0-25.9)	26.9 (25.9-28.0)
Congo	Men	20.6 (19.3-21.9)	19.9 (18.3-21.5)	21.1 (19.8-22.4)	22.4 (20.9-23.8)	21.6 (19.9-23.3)	22.8 (21.3-24.2)
	Women	21.7 (20.8-22.5)	20.6 (19.8-21.5)	22.6 (21.5-23.7)	24.2 (23.1-25.2)	22.5 (21.4-23.6)	25.0 (23.9-26.1)
Cook Islands	Men	30.4 (29.0-31.8)	29.3 (27.7-31.0)	31.2 (29.7-32.7)	32.6 (31.7-33.5)	32.4 (31.2-33.6)	32.6 (31.6-33.6)
	Women	31.1 (29.2-32.9)	30.5 (28.3-32.6)	31.6 (29.6-33.5)	33.1 (32.0-34.2)	32.4 (30.8-34.0)	33.4 (32.2-34.6)
Costa Rica	Men	24.3 (22.9-25.7)	24.0 (22.5-25.4)	24.7 (23.3-26.1)	26.9 (26.1-27.7)	26.4 (25.5-27.2)	27.1 (26.2-27.9)
	Women	23.8 (21.9-25.6)	23.1 (21.2-25.1)	24.5 (22.6-26.4)	28.0 (27.1-28.9)	27.6 (26.7-28.6)	28.1 (27.2-29.0)

Country	Sex	Age-standardised mean BMI in 1985 (kg/m^2)			Age-standardised mean BMI in 2017 (kg/m^2)		
		National	Rural	Urban	National	Rural	Urban
Cote d'Ivoire	Men	21.5 (20.0-23.0)	21.1 (19.5-22.7)	22.1 (20.5-23.7)	23.4 (22.1-24.8)	22.9 (21.5-24.3)	23.8 (22.4-25.3)
	Women	21.5 (20.2-22.9)	20.7 (19.3-22.2)	22.9 (21.4-24.3)	24.2 (23.1-25.2)	22.7 (21.5-23.8)	25.4 (24.2-26.5)
Croatia	Men	25.4 (24.2-26.5)	25.1 (23.8-26.4)	25.6 (24.4-26.8)	27.6 (26.7-28.5)	27.4 (26.5-28.2)	27.7 (26.7-28.8)
	Women	25.0 (23.3-26.6)	25.3 (23.5-27.1)	24.7 (22.9-26.5)	26.2 (25.0-27.4)	26.7 (25.5-27.8)	25.9 (24.4-27.5)
Cuba	Men	22.8 (21.5-24.1)	22.0 (20.5-23.4)	23.1 (21.8-24.5)	25.3 (24.4-26.2)	24.7 (23.7-25.7)	25.5 (24.5-26.4)
	Women	23.5 (21.7-25.2)	22.7 (20.7-24.6)	23.8 (22.0-25.6)	26.3 (25.1-27.4)	26.2 (24.9-27.4)	26.3 (25.2-27.5)
Cyprus	Men	25.6 (24.4-26.9)	25.8 (24.5-27.2)	25.5 (24.3-26.8)	27.4 (26.1-28.7)	27.4 (26.0-28.8)	27.4 (26.2-28.8)
	Women	25.4 (23.8-27.2)	25.6 (23.7-27.6)	25.3 (23.6-27.1)	25.5 (23.7-27.3)	25.4 (23.4-27.4)	25.5 (23.7-27.4)
Czech Republic	Men	26.4 (25.9-26.9)	26.6 (25.9-27.3)	26.3 (25.8-26.9)	27.9 (27.3-28.5)	28.2 (27.6-28.9)	27.8 (27.2-28.4)
	Women	26.6 (26.0-27.2)	27.6 (26.6-28.5)	26.3 (25.7-27.0)	26.1 (25.5-26.8)	26.8 (26.1-27.6)	25.9 (25.2-26.6)
Denmark	Men	24.0 (23.5-24.4)	24.1 (23.4-24.9)	23.9 (23.5-24.4)	26.3 (25.7-26.8)	26.3 (25.6-27.0)	26.3 (25.7-26.8)
	Women	24.2 (23.3-25.2)	24.6 (23.2-25.9)	24.2 (23.2-25.1)	24.5 (23.5-25.6)	24.6 (23.5-25.8)	24.5 (23.5-25.6)
Djibouti	Men	21.0 (18.6-23.3)	20.1 (17.7-22.6)	21.2 (18.8-23.7)	23.0 (20.6-25.3)	22.0 (19.6-24.4)	23.3 (20.9-25.6)
	Women	22.0 (18.9-25.1)	20.8 (17.7-24.0)	22.4 (19.2-25.6)	24.9 (21.7-27.9)	23.0 (19.8-26.1)	25.4 (22.2-28.5)
Dominica	Men	22.4 (21.0-23.9)	21.9 (20.2-23.5)	22.8 (21.4-24.4)	25.0 (23.8-26.1)	24.5 (23.2-25.9)	25.2 (23.9-26.4)
	Women	25.4 (23.4-27.4)	24.7 (22.4-26.9)	26.1 (24.0-28.2)	29.1 (27.5-30.5)	28.7 (27.0-30.5)	29.2 (27.6-30.8)
Dominican Republic	Men	23.0 (21.9-24.0)	22.4 (21.2-23.6)	23.4 (22.3-24.5)	26.1 (25.4-26.8)	25.6 (24.8-26.3)	26.3 (25.5-27.0)
	Women	23.3 (22.3-24.4)	22.6 (21.4-23.8)	24.0 (22.8-25.1)	27.7 (26.8-28.5)	27.4 (26.5-28.3)	27.7 (26.9-28.6)
DR Congo	Men	19.4 (17.6-21.2)	19.1 (17.2-21.0)	20.2 (18.5-22.0)	21.3 (19.8-22.9)	20.8 (19.1-22.7)	22.0 (20.4-23.5)
	Women	20.0 (18.2-21.8)	19.5 (17.7-21.5)	21.2 (19.3-23.0)	22.7 (21.8-23.7)	21.7 (20.7-22.7)	24.1 (23.1-25.1)
Ecuador	Men	23.5 (22.0-24.9)	23.1 (21.5-24.6)	24.0 (22.4-25.5)	26.5 (25.7-27.3)	25.6 (24.8-26.4)	27.0 (26.2-27.8)
	Women	24.5 (22.6-26.4)	23.8 (21.8-25.8)	25.2 (23.2-27.2)	28.0 (27.0-29.0)	27.4 (26.4-28.4)	28.3 (27.3-29.4)
Egypt	Men	25.0 (23.7-26.1)	24.7 (23.4-25.9)	25.3 (24.1-26.6)	27.5 (27.1-28.0)	27.2 (26.7-27.7)	27.9 (27.4-28.4)
	Women	26.6 (25.4-27.7)	25.4 (24.1-26.6)	28.1 (26.9-29.3)	31.7 (31.3-32.2)	31.3 (30.8-31.9)	32.3 (31.7-32.8)
El Salvador	Men	24.3 (22.5-26.2)	23.8 (21.9-25.7)	24.9 (23.1-26.8)	27.1 (25.4-28.7)	26.3 (24.5-28.0)	27.4 (25.8-29.1)
	Women	23.9 (22.1-25.7)	23.2 (21.3-25.1)	24.8 (22.8-26.6)	28.5 (27.2-29.8)	28.0 (26.6-29.4)	28.7 (27.4-30.1)
Equatorial Guinea	Men	20.6 (18.0-23.2)	20.2 (17.6-22.9)	21.4 (18.8-24.1)	22.5 (19.9-25.0)	22.0 (19.3-24.6)	23.2 (20.6-25.8)
	Women	22.8 (20.4-25.0)	22.3 (19.9-24.6)	24.0 (21.5-26.4)	25.9 (24.6-27.2)	25.0 (23.5-26.5)	27.3 (25.7-28.9)
Eritrea	Men	19.0 (17.7-20.4)	18.9 (17.5-20.2)	20.0 (18.5-21.5)	20.5 (19.7-21.4)	20.2 (19.3-21.1)	21.6 (20.4-22.7)
	Women	19.1 (17.7-20.5)	18.8 (17.4-20.3)	20.5 (18.8-22.2)	21.1 (20.0-22.2)	20.5 (19.3-21.6)	23.1 (21.5-24.6)
Estonia	Men	24.8 (23.9-25.7)	25.0 (24.0-26.1)	24.7 (23.7-25.6)	26.6 (26.0-27.2)	26.6 (26.0-27.2)	26.6 (26.0-27.2)
	Women	25.3 (24.2-26.5)	25.8 (24.4-27.1)	25.1 (23.9-26.4)	25.7 (25.0-26.4)	26.2 (25.4-26.9)	25.5 (24.8-26.2)
Ethiopia	Men	18.5 (17.2-20.0)	18.4 (17.0-19.9)	19.8 (18.4-21.3)	20.2 (19.6-20.8)	19.8 (19.2-20.4)	21.5 (20.9-22.1)
	Women	18.9 (17.2-20.5)	18.7 (17.0-20.4)	20.2 (18.5-21.9)	21.0 (20.2-21.8)	20.4 (19.6-21.2)	23.1 (22.3-23.9)
Fiji	Men	24.1 (22.7-25.4)	23.4 (22.0-24.8)	25.1 (23.6-26.6)	26.5 (25.7-27.4)	26.4 (25.4-27.4)	26.7 (25.7-27.7)
	Women	25.8 (23.8-27.9)	25.5 (23.4-27.6)	26.4 (24.2-28.6)	28.8 (27.7-29.9)	28.3 (27.1-29.5)	29.2 (28.0-30.4)
Finland	Men	25.2 (24.7-25.7)	25.5 (25.0-26.0)	25.1 (24.6-25.6)	26.4 (25.8-27.0)	26.6 (26.0-27.3)	26.3 (25.7-26.9)
	Women	24.5 (23.8-25.2)	25.2 (24.3-26.0)	24.3 (23.6-25.0)	25.4 (24.6-26.1)	25.9 (25.0-26.7)	25.3 (24.5-26.1)
France	Men	24.9 (24.3-25.5)	25.4 (24.6-26.2)	24.7 (24.1-25.4)	26.0 (25.1-26.9)	26.3 (25.3-27.3)	25.9 (25.0-26.8)
	Women	24.2 (23.4-25.0)	24.6 (23.5-25.8)	24.0 (23.2-24.9)	24.0 (22.8-25.2)	24.2 (22.9-25.5)	24.0 (22.7-25.1)
French Polynesia	Men	28.2 (26.8-29.7)	27.1 (25.6-28.7)	29.0 (27.4-30.6)	29.7 (28.7-30.7)	29.6 (28.5-30.8)	29.8 (28.6-30.9)
	Women	28.7 (26.6-30.9)	28.1 (25.8-30.5)	29.1 (26.9-31.5)	29.6 (28.3-30.9)	29.1 (27.5-30.7)	30.0 (28.5-31.5)
Gabon	Men	21.7 (20.0-23.5)	20.9 (19.0-22.9)	22.2 (20.4-24.0)	24.0 (22.6-25.4)	22.9 (21.2-24.6)	24.2 (22.8-25.6)
	Women	22.5 (20.6-24.3)	21.3 (19.4-23.2)	23.2 (21.3-25.1)	26.8 (25.6-27.9)	24.4 (23.3-25.7)	27.1 (25.9-28.3)

Country	Sex	Age-standardised mean BMI in 1985 (kg/m²)			Age-standardised mean BMI in 2017 (kg/m²)		
		National	Rural	Urban	National	Rural	Urban
Gambia	Men	20.0 (18.8-21.2)	19.6 (18.3-20.9)	20.9 (19.6-22.2)	23.0 (22.0-24.0)	22.3 (21.2-23.4)	23.5 (22.4-24.6)
	Women	20.5 (19.1-22.0)	19.8 (18.2-21.4)	22.1 (20.5-23.7)	24.6 (23.7-25.5)	22.9 (21.9-23.9)	25.8 (24.8-26.8)
Georgia	Men	24.7 (23.3-26.2)	24.4 (22.8-25.9)	25.1 (23.5-26.6)	27.5 (26.8-28.1)	27.3 (26.6-28.0)	27.6 (26.9-28.3)
	Women	24.3 (22.2-26.4)	24.0 (21.8-26.1)	24.5 (22.4-26.7)	27.6 (26.9-28.3)	27.9 (27.1-28.8)	27.3 (26.5-28.2)
Germany	Men	25.3 (24.9-25.6)	25.6 (25.1-26.1)	25.1 (24.8-25.5)	27.3 (26.6-28.1)	27.5 (26.7-28.3)	27.3 (26.5-28.0)
	Women	24.6 (24.1-25.1)	25.2 (24.4-25.9)	24.4 (23.9-24.9)	25.6 (24.6-26.7)	26.0 (24.9-27.1)	25.5 (24.4-26.6)
Ghana	Men	20.8 (19.6-22.1)	20.2 (18.9-21.5)	22.1 (20.8-23.5)	22.8 (22.1-23.4)	21.7 (21.0-22.4)	23.6 (22.9-24.3)
	Women	21.5 (20.2-22.8)	20.6 (19.3-21.9)	23.2 (21.9-24.6)	25.6 (24.8-26.4)	23.8 (22.9-24.6)	27.1 (26.3-28.0)
Greece	Men	25.5 (24.6-26.5)	25.6 (24.6-26.7)	25.5 (24.5-26.5)	26.9 (26.2-27.6)	26.7 (25.9-27.5)	26.9 (26.2-27.6)
	Women	26.3 (24.9-27.6)	26.2 (24.7-27.8)	26.3 (24.8-27.7)	25.0 (24.2-25.9)	24.7 (23.6-25.6)	25.1 (24.3-26.0)
Greenland	Men	23.9 (22.6-25.2)	23.7 (22.4-25.0)	23.9 (22.6-25.2)	25.8 (24.8-26.8)	25.4 (24.4-26.5)	25.9 (24.8-26.9)
	Women	25.5 (23.6-27.4)	26.0 (24.0-28.0)	25.3 (23.4-27.3)	26.5 (25.1-27.8)	26.9 (25.4-28.3)	26.4 (25.1-27.8)
Grenada	Men	22.7 (21.2-24.2)	22.4 (20.7-24.0)	23.4 (21.8-25.0)	25.2 (24.2-26.2)	25.0 (23.9-26.0)	25.7 (24.6-26.9)
	Women	25.0 (22.9-27.1)	24.6 (22.4-26.8)	25.9 (23.6-28.1)	28.8 (27.6-30.1)	28.7 (27.3-30.0)	29.1 (27.6-30.6)
Guatemala	Men	23.1 (21.8-24.3)	22.5 (21.1-23.8)	24.0 (22.7-25.4)	25.7 (24.7-26.6)	24.8 (23.9-25.8)	26.4 (25.5-27.4)
	Women	23.0 (21.6-24.4)	22.3 (20.8-23.8)	24.1 (22.6-25.6)	27.4 (26.6-28.2)	26.8 (25.9-27.6)	27.9 (27.1-28.8)
Guinea	Men	20.5 (18.9-22.1)	20.2 (18.5-21.8)	21.5 (19.8-23.2)	22.4 (21.2-23.7)	21.9 (20.7-23.3)	23.1 (21.7-24.5)
	Women	20.9 (19.2-22.5)	20.3 (18.6-22.0)	22.5 (20.8-24.3)	23.5 (22.5-24.6)	22.4 (21.3-23.5)	25.5 (24.4-26.6)
Guinea Bissau	Men	20.7 (18.3-23.1)	20.4 (17.9-22.8)	21.7 (19.2-24.1)	22.8 (20.5-25.2)	22.2 (19.8-24.7)	23.4 (21.0-25.8)
	Women	21.1 (19.1-23.1)	20.6 (18.5-22.6)	22.7 (20.6-24.7)	24.2 (22.9-25.5)	22.8 (21.4-24.1)	25.6 (24.2-26.9)
Guyana	Men	22.5 (21.1-24.0)	22.3 (20.7-23.8)	23.1 (21.6-24.7)	25.1 (24.0-26.2)	24.9 (23.8-26.1)	25.5 (24.3-26.6)
	Women	23.9 (21.9-26.1)	23.6 (21.4-25.7)	24.8 (22.7-27.0)	27.6 (26.2-29.0)	27.5 (26.1-29.0)	27.8 (26.4-29.2)
Haiti	Men	23.5 (21.0-25.9)	23.2 (20.7-25.8)	24.3 (21.8-26.7)	26.5 (24.0-28.9)	26.0 (23.6-28.5)	26.7 (24.3-29.2)
	Women	20.0 (18.5-21.4)	19.4 (17.8-20.9)	21.8 (20.3-23.4)	24.6 (23.8-25.3)	23.5 (22.7-24.3)	25.2 (24.4-26.0)
Honduras	Men	23.5 (21.7-25.3)	23.1 (21.2-25.0)	24.2 (22.4-26.0)	26.2 (24.6-27.8)	25.5 (23.8-27.3)	26.6 (25.1-28.2)
	Women	22.1 (20.5-23.8)	21.4 (19.7-23.1)	23.4 (21.7-25.1)	27.7 (26.7-28.7)	26.9 (25.9-28.0)	28.3 (27.3-29.4)
Hungary	Men	25.7 (25.0-26.3)	25.6 (24.7-26.4)	25.7 (25.0-26.4)	27.9 (26.7-29.1)	27.9 (26.5-29.2)	27.9 (26.6-29.1)
	Women	25.4 (24.7-26.1)	25.6 (24.5-26.7)	25.3 (24.4-26.1)	25.9 (23.8-28.0)	26.2 (24.0-28.5)	25.8 (23.6-28.0)
Iceland	Men	24.8 (24.2-25.5)	25.2 (24.3-26.1)	24.8 (24.1-25.4)	27.1 (26.2-27.9)	27.3 (26.1-28.4)	27.1 (26.2-27.9)
	Women	24.3 (23.4-25.2)	24.9 (23.7-26.1)	24.3 (23.3-25.2)	25.5 (24.4-26.7)	25.9 (24.2-27.5)	25.5 (24.4-26.7)
India	Men	19.6 (19.0-20.3)	18.9 (18.2-19.6)	21.9 (21.2-22.7)	21.9 (21.4-22.3)	21.5 (21.0-21.9)	22.6 (22.2-23.1)
	Women	19.7 (18.9-20.6)	19.0 (18.1-19.9)	22.2 (21.3-23.1)	22.1 (21.6-22.6)	21.5 (21.0-22.0)	23.2 (22.7-23.8)
Indonesia	Men	20.1 (19.3-20.9)	19.8 (19.0-20.6)	20.9 (20.1-21.8)	22.5 (22.0-23.0)	21.9 (21.4-22.4)	23.1 (22.5-23.6)
	Women	20.5 (19.5-21.5)	20.0 (19.0-21.1)	21.9 (20.8-23.0)	24.0 (23.4-24.6)	23.3 (22.6-24.0)	24.5 (23.9-25.2)
Iran	Men	22.6 (21.6-23.6)	22.0 (21.0-23.0)	23.2 (22.2-24.2)	25.4 (25.0-25.8)	24.5 (24.0-24.9)	25.7 (25.3-26.2)
	Women	24.2 (22.7-25.6)	23.1 (21.6-24.6)	25.1 (23.7-26.6)	27.3 (26.8-27.8)	26.5 (25.9-27.1)	27.5 (27.0-28.1)
Iraq	Men	24.6 (23.2-25.9)	24.2 (22.7-25.6)	24.8 (23.4-26.2)	27.7 (27.0-28.4)	27.2 (26.4-28.1)	27.9 (27.2-28.6)
	Women	26.1 (24.3-28.0)	24.8 (22.8-26.9)	26.7 (24.8-28.6)	29.8 (29.0-30.6)	29.1 (28.1-30.0)	30.1 (29.2-30.9)
Ireland	Men	25.4 (24.4-26.4)	25.7 (24.6-26.8)	25.2 (24.2-26.2)	27.8 (27.0-28.6)	28.1 (27.2-29.0)	27.7 (26.9-28.5)
	Women	24.4 (23.0-25.8)	24.7 (23.1-26.2)	24.2 (22.7-25.7)	26.8 (25.7-27.8)	27.0 (25.9-28.1)	26.7 (25.6-27.7)
Israel	Men	24.6 (23.7-25.5)	24.3 (23.2-25.3)	24.6 (23.8-25.6)	26.4 (25.8-26.9)	25.6 (24.9-26.2)	26.4 (25.8-27.0)
	Women	24.9 (23.7-26.1)	24.0 (22.6-25.5)	25.0 (23.8-26.2)	25.6 (24.9-26.3)	24.5 (23.7-25.3)	25.7 (24.9-26.4)
Italy	Men	24.9 (24.5-25.4)	25.1 (24.5-25.7)	24.9 (24.4-25.4)	26.2 (25.5-26.9)	26.1 (25.4-26.8)	26.3 (25.6-27.0)
	Women	24.9 (24.2-25.5)	25.4 (24.6-26.2)	24.6 (24.0-25.3)	24.5 (23.6-25.3)	24.3 (23.3-25.2)	24.5 (23.6-25.4)

Country	Sex	Age-standardised mean BMI in 1985 (kg/m^2)			Age-standardised mean BMI in 2017 (kg/m^2)		
		National	Rural	Urban	National	Rural	Urban
Jamaica	Men	22.6 (21.5-23.7)	22.1 (20.8-23.3)	23.1 (22.0-24.3)	25.4 (24.6-26.3)	25.0 (24.1-25.9)	25.7 (24.8-26.6)
	Women	25.1 (23.5-26.5)	24.6 (23.0-26.2)	25.5 (24.0-27.0)	29.1 (28.0-30.1)	29.1 (27.9-30.3)	29.0 (27.9-30.1)
Japan	Men	22.3 (22.1-22.6)	22.3 (22.0-22.6)	22.3 (22.1-22.6)	23.6 (23.2-24.0)	23.6 (23.2-24.1)	23.6 (23.2-24.0)
	Women	22.0 (21.8-22.3)	22.2 (21.9-22.5)	22.0 (21.7-22.3)	21.6 (21.1-22.1)	21.9 (21.3-22.4)	21.6 (21.1-22.1)
Jordan	Men	25.3 (24.1-26.4)	25.0 (23.7-26.2)	25.4 (24.2-26.6)	28.3 (27.7-28.9)	27.9 (27.2-28.6)	28.4 (27.7-29.0)
	Women	27.7 (26.3-29.2)	27.0 (25.4-28.5)	28.1 (26.6-29.6)	29.9 (29.3-30.5)	30.0 (29.3-30.7)	29.9 (29.2-30.5)
Kazakhstan	Men	24.1 (23.0-25.3)	23.8 (22.4-25.1)	24.4 (23.3-25.6)	26.3 (25.6-27.1)	26.2 (25.4-27.0)	26.5 (25.7-27.3)
	Women	25.2 (23.7-26.7)	24.9 (23.3-26.5)	25.4 (23.8-27.0)	26.4 (25.5-27.3)	26.6 (25.6-27.5)	26.3 (25.3-27.2)
Kenya	Men	20.1 (19.0-21.3)	19.9 (18.8-21.1)	21.2 (19.9-22.5)	22.0 (21.3-22.7)	21.6 (20.9-22.3)	23.1 (22.3-23.9)
	Women	21.2 (20.1-22.3)	20.9 (19.7-22.0)	23.0 (21.7-24.2)	24.4 (23.7-25.1)	23.7 (22.9-24.4)	26.5 (25.7-27.3)
Kiribati	Men	27.2 (25.8-28.6)	26.6 (25.1-28.1)	28.4 (26.9-29.9)	28.8 (27.9-29.6)	28.7 (27.7-29.7)	28.9 (27.8-29.9)
	Women	29.0 (27.1-31.0)	28.7 (26.6-30.7)	29.7 (27.7-31.8)	30.3 (29.4-31.3)	29.9 (28.7-31.1)	30.9 (29.6-32.2)
Kuwait	Men	26.0 (24.9-27.1)	25.2 (23.8-26.6)	26.0 (24.9-27.1)	29.0 (28.3-29.7)	28.2 (27.1-29.2)	29.0 (28.4-29.7)
	Women	28.1 (26.5-29.7)	26.3 (24.3-28.2)	28.2 (26.6-29.7)	30.6 (29.8-31.4)	29.8 (28.2-31.3)	30.6 (29.8-31.4)
Kyrgyzstan	Men	23.5 (22.4-24.6)	23.2 (22.0-24.4)	23.9 (22.7-25.2)	26.0 (25.1-26.8)	25.8 (24.9-26.8)	26.2 (25.2-27.3)
	Women	23.0 (21.6-24.5)	22.8 (21.3-24.3)	23.4 (21.9-25.0)	27.2 (26.3-28.1)	27.4 (26.5-28.3)	26.8 (25.9-27.8)
Lao PDR	Men	20.4 (19.0-21.8)	20.3 (18.8-21.7)	20.9 (19.5-22.4)	22.8 (22.0-23.6)	22.5 (21.7-23.4)	23.2 (22.3-24.1)
	Women	19.8 (17.9-21.7)	19.6 (17.7-21.5)	21.2 (19.3-23.2)	23.2 (22.3-24.2)	22.8 (21.7-23.8)	23.9 (22.8-25.0)
Latvia	Men	24.9 (23.7-26.1)	25.2 (23.9-26.4)	24.8 (23.5-26.0)	26.8 (25.9-27.8)	26.9 (25.8-27.9)	26.8 (25.8-27.9)
	Women	25.4 (23.7-27.1)	25.9 (24.1-27.8)	25.1 (23.4-26.9)	25.8 (24.6-27.1)	26.5 (25.2-27.8)	25.5 (24.2-26.8)
Lebanon	Men	24.9 (23.7-26.0)	24.7 (23.4-25.9)	24.9 (23.8-26.1)	27.7 (27.1-28.3)	27.4 (26.7-28.2)	27.7 (27.1-28.3)
	Women	25.0 (23.5-26.5)	24.3 (22.7-26.0)	25.2 (23.6-26.7)	27.0 (26.3-27.7)	27.1 (26.2-28.1)	27.0 (26.2-27.7)
Lesotho	Men	20.8 (19.4-22.2)	20.7 (19.2-22.2)	21.8 (20.3-23.2)	22.6 (22.0-23.3)	22.3 (21.7-23.0)	23.5 (22.8-24.2)
	Women	25.5 (24.0-26.9)	25.3 (23.8-26.8)	27.0 (25.3-28.7)	26.9 (26.1-27.7)	26.4 (25.5-27.3)	28.1 (27.1-29.0)
Liberia	Men	21.5 (20.1-22.9)	21.1 (19.6-22.6)	22.2 (20.7-23.7)	23.1 (22.4-23.8)	22.6 (21.8-23.4)	23.6 (22.8-24.4)
	Women	21.6 (19.6-23.5)	20.8 (18.8-22.8)	22.7 (20.7-24.7)	25.0 (24.1-26.0)	23.8 (22.8-24.8)	26.3 (25.3-27.3)
Libya	Men	24.2 (22.9-25.6)	23.7 (22.2-25.2)	24.4 (23.0-25.8)	26.9 (25.9-27.9)	26.3 (25.1-27.5)	27.1 (26.1-28.1)
	Women	26.3 (24.5-28.2)	25.0 (22.9-27.0)	26.8 (24.9-28.7)	29.5 (28.2-30.7)	28.9 (27.2-30.5)	29.6 (28.3-31.0)
Lithuania	Men	25.6 (24.8-26.4)	25.7 (24.8-26.5)	25.5 (24.7-26.4)	27.0 (25.9-28.1)	26.9 (25.7-28.0)	27.1 (26.0-28.3)
	Women	26.7 (25.7-27.8)	27.1 (26.0-28.3)	26.5 (25.4-27.7)	26.0 (24.5-27.6)	26.5 (24.9-28.1)	25.8 (24.2-27.4)
Luxembourg	Men	24.8 (23.6-26.1)	25.2 (23.9-26.5)	24.8 (23.5-26.1)	26.9 (25.8-27.9)	27.1 (26.0-28.2)	26.9 (25.8-27.9)
	Women	24.5 (22.6-26.4)	24.9 (22.9-26.8)	24.4 (22.5-26.3)	25.4 (24.1-26.9)	25.6 (24.2-27.0)	25.4 (24.0-26.9)
Macedonia (TFYR)	Men	25.3 (23.0-27.7)	25.3 (22.9-27.6)	25.4 (23.0-27.8)	27.3 (24.9-29.7)	27.3 (24.8-29.7)	27.3 (24.8-29.7)
	Women	25.6 (23.8-27.5)	25.9 (23.9-27.9)	25.4 (23.4-27.3)	26.1 (24.1-28.0)	26.4 (24.3-28.5)	25.9 (23.8-27.9)
Madagascar	Men	19.9 (18.6-21.3)	19.7 (18.3-21.1)	20.8 (19.3-22.3)	21.9 (20.7-23.1)	21.4 (20.1-22.7)	22.7 (21.4-24.1)
	Women	19.7 (18.1-21.2)	19.5 (17.9-21.0)	20.4 (18.8-22.0)	21.5 (20.3-22.6)	20.8 (19.5-22.0)	22.6 (21.4-23.8)
Malawi	Men	20.1 (18.8-21.4)	20.0 (18.7-21.3)	20.9 (19.5-22.3)	22.0 (21.4-22.5)	21.8 (21.2-22.3)	22.9 (22.3-23.6)
	Women	20.8 (19.6-22.0)	20.7 (19.5-21.9)	22.3 (21.0-23.6)	23.5 (23.0-24.0)	23.1 (22.5-23.6)	25.9 (25.3-26.5)
Malaysia	Men	22.2 (21.1-23.2)	21.9 (20.8-22.9)	22.6 (21.5-23.6)	25.3 (24.8-25.8)	24.7 (24.2-25.3)	25.5 (24.9-26.0)
	Women	22.1 (20.7-23.5)	21.8 (20.4-23.2)	22.5 (21.0-23.9)	26.1 (25.5-26.7)	26.2 (25.6-26.9)	26.1 (25.4-26.7)
Maldives	Men	21.4 (19.9-22.9)	21.1 (19.5-22.7)	22.1 (20.6-23.5)	24.0 (22.9-25.2)	23.5 (22.2-24.9)	24.5 (23.4-25.7)
	Women	22.6 (20.9-24.3)	22.3 (20.6-24.1)	23.3 (21.5-25.1)	25.9 (24.8-27.1)	25.7 (24.5-27.0)	26.2 (25.0-27.4)
Mali	Men	20.7 (19.3-22.1)	20.4 (19.0-21.8)	21.8 (20.3-23.3)	22.9 (21.7-24.1)	22.4 (21.2-23.7)	23.6 (22.3-25.0)
	Women	20.0 (18.6-21.5)	19.6 (18.1-21.1)	21.7 (20.2-23.2)	23.6 (22.7-24.6)	22.3 (21.3-23.2)	25.6 (24.5-26.6)

Country	Sex	Age-standardised mean BMI in 1985 (kg/m^2)			Age-standardised mean BMI in 2017 (kg/m^2)		
		National	Rural	Urban	National	Rural	Urban
Malta	Men	25.5 (23.9-27.0)	25.8 (24.3-27.2)	25.5 (23.9-27.1)	27.2 (25.3-29.1)	27.2 (25.3-29.0)	27.2 (25.3-29.1)
	Women	25.5 (23.5-27.6)	25.8 (24.1-27.6)	25.5 (23.4-27.6)	25.5 (22.8-28.1)	25.4 (22.9-28.0)	25.5 (22.8-28.2)
Marshall Islands	Men	27.4 (26.0-28.8)	26.3 (24.7-27.8)	28.1 (26.6-29.5)	28.9 (27.6-30.3)	28.8 (27.4-30.3)	29.0 (27.5-30.4)
	Women	29.0 (27.2-30.9)	28.4 (26.4-30.6)	29.4 (27.4-31.3)	30.5 (28.8-32.3)	29.9 (27.9-31.9)	30.8 (28.9-32.6)
Mauritania	Men	21.2 (19.5-22.9)	20.7 (18.9-22.6)	22.0 (20.3-23.8)	23.3 (21.7-24.8)	22.6 (20.8-24.2)	23.8 (22.1-25.3)
	Women	23.3 (21.5-25.1)	22.6 (20.7-24.5)	24.6 (22.8-26.4)	26.6 (25.0-28.2)	24.9 (23.1-26.6)	27.7 (26.0-29.3)
Mauritius	Men	22.5 (21.9-23.1)	22.4 (21.7-23.0)	22.8 (22.1-23.4)	24.6 (23.7-25.5)	24.4 (23.4-25.3)	25.0 (24.0-25.9)
	Women	23.7 (22.9-24.4)	23.5 (22.7-24.3)	24.0 (23.2-24.7)	25.8 (24.6-27.0)	25.5 (24.2-26.7)	26.3 (25.0-27.5)
Mexico	Men	25.1 (24.1-26.1)	24.4 (23.3-25.6)	25.4 (24.4-26.4)	27.6 (27.1-28.2)	26.8 (26.3-27.3)	27.9 (27.3-28.4)
	Women	24.2 (23.2-25.2)	23.3 (22.2-24.4)	24.6 (23.6-25.6)	28.5 (27.9-29.1)	28.1 (27.5-28.8)	28.6 (28.0-29.2)
Micronesia (Federated States of)	Men	26.5 (25.1-27.8)	26.0 (24.6-27.5)	27.7 (26.2-29.1)	28.7 (27.7-29.8)	28.8 (27.7-29.9)	28.7 (27.5-29.8)
	Women	29.0 (27.1-31.0)	28.6 (26.7-30.7)	30.1 (28.1-32.2)	30.2 (28.9-31.5)	29.9 (28.5-31.2)	31.5 (30.0-33.0)
Moldova	Men	24.9 (23.5-26.4)	24.8 (23.3-26.4)	25.1 (23.5-26.6)	26.6 (25.8-27.4)	26.3 (25.5-27.2)	27.0 (26.2-27.9)
	Women	26.8 (24.9-28.7)	27.0 (25.1-29.0)	26.5 (24.6-28.4)	27.1 (26.2-28.0)	27.4 (26.4-28.4)	26.8 (25.8-27.8)
Mongolia	Men	22.9 (21.6-24.2)	22.6 (21.2-23.9)	23.2 (21.8-24.5)	25.8 (25.1-26.5)	25.6 (24.9-26.4)	25.8 (25.1-26.6)
	Women	23.5 (21.8-25.2)	23.1 (21.3-25.0)	23.8 (22.0-25.6)	26.6 (25.7-27.5)	26.8 (25.8-27.8)	26.5 (25.5-27.5)
Montenegro	Men	25.1 (23.1-27.1)	25.1 (23.0-27.1)	25.2 (23.1-27.2)	27.1 (25.2-29.0)	27.1 (25.1-29.1)	27.1 (25.1-29.0)
	Women	25.6 (22.9-28.3)	25.8 (23.1-28.6)	25.4 (22.6-28.2)	26.0 (23.3-28.8)	26.3 (23.5-29.2)	25.8 (23.1-28.6)
Morocco	Men	22.2 (21.0-23.5)	21.9 (20.5-23.2)	22.7 (21.4-24.0)	24.6 (24.0-25.2)	24.1 (23.4-24.7)	24.9 (24.2-25.6)
	Women	23.9 (22.7-25.1)	22.8 (21.6-24.1)	25.3 (24.0-26.6)	27.1 (26.4-27.8)	26.3 (25.5-27.0)	27.6 (26.9-28.4)
Mozambique	Men	19.9 (18.6-21.2)	19.7 (18.3-21.0)	21.0 (19.7-22.4)	22.1 (21.5-22.8)	21.6 (20.9-22.4)	23.2 (22.4-24.0)
	Women	20.6 (19.1-22.2)	20.3 (18.7-21.8)	22.2 (20.7-23.8)	23.3 (22.5-24.1)	22.4 (21.5-23.2)	25.3 (24.4-26.1)
Myanmar	Men	19.8 (18.5-21.1)	19.5 (18.2-20.8)	20.6 (19.2-21.9)	21.8 (21.2-22.5)	21.4 (20.8-22.1)	22.6 (21.9-23.3)
	Women	20.2 (18.4-22.1)	19.8 (18.0-21.7)	21.3 (19.4-23.2)	23.0 (22.4-23.6)	22.5 (21.9-23.1)	23.9 (23.2-24.5)
Namibia	Men	20.9 (19.6-22.3)	20.6 (19.1-22.1)	21.8 (20.4-23.3)	22.9 (21.9-24.0)	22.3 (21.1-23.5)	23.7 (22.5-24.8)
	Women	22.8 (21.5-24.1)	22.0 (20.6-23.3)	25.0 (23.6-26.3)	25.3 (24.4-26.2)	23.9 (22.9-24.8)	26.8 (25.8-27.8)
Nauru	Men	32.6 (32.0-33.2)	na*	32.6 (32.0-33.2)	32.9 (31.7-34.1)	na*	32.9 (31.7-34.1)
	Women	34.1 (33.4-34.8)	na*	34.1 (33.4-34.8)	33.6 (32.0-35.2)	na*	33.6 (32.0-35.2)
Nepal	Men	19.7 (18.5-20.9)	19.5 (18.3-20.8)	22.2 (20.9-23.5)	22.4 (21.9-22.9)	22.2 (21.7-22.7)	23.2 (22.7-23.8)
	Women	18.4 (17.0-19.9)	18.3 (16.8-19.7)	20.5 (19.0-22.0)	22.5 (21.9-23.1)	22.1 (21.5-22.7)	23.9 (23.3-24.5)
Netherlands	Men	24.1 (23.4-24.8)	24.3 (23.5-25.1)	24.0 (23.3-24.7)	25.9 (25.2-26.7)	26.0 (25.1-26.8)	25.9 (25.2-26.7)
	Women	24.3 (23.2-25.3)	24.5 (23.3-25.7)	24.2 (23.1-25.2)	25.0 (24.0-26.0)	24.9 (23.8-26.0)	25.0 (24.0-26.0)
New Zealand	Men	25.3 (24.6-26.0)	25.3 (24.5-26.2)	25.3 (24.5-26.0)	28.1 (27.6-28.5)	28.0 (27.5-28.5)	28.1 (27.6-28.5)
	Women	24.6 (23.7-25.5)	24.8 (23.7-25.9)	24.6 (23.7-25.5)	28.0 (27.5-28.5)	28.0 (27.4-28.6)	28.0 (27.5-28.5)
Nicaragua	Men	24.2 (22.4-26.0)	23.6 (21.8-25.6)	24.8 (23.0-26.5)	26.8 (25.1-28.4)	26.1 (24.3-27.8)	27.2 (25.6-28.9)
	Women	23.6 (22.0-25.1)	22.6 (21.0-24.3)	24.5 (22.9-26.1)	28.0 (27.0-29.0)	27.3 (26.3-28.4)	28.5 (27.5-29.5)
Niger	Men	19.6 (18.2-21.1)	19.5 (18.0-21.0)	20.8 (19.2-22.4)	21.4 (20.3-22.5)	21.2 (20.1-22.3)	22.4 (21.1-23.7)
	Women	20.0 (18.8-21.2)	19.6 (18.3-20.9)	22.4 (21.1-23.7)	22.3 (21.3-23.3)	21.6 (20.6-22.6)	25.3 (24.2-26.4)
Nigeria	Men	21.0 (20.2-21.8)	20.8 (19.9-21.6)	21.7 (20.8-22.5)	22.5 (21.6-23.3)	22.1 (21.2-23.0)	22.9 (21.9-23.8)
	Women	22.2 (21.2-23.2)	21.8 (20.7-22.9)	23.5 (22.4-24.5)	24.1 (23.2-24.9)	23.1 (22.3-24.0)	25.0 (24.1-25.9)
Niue	Men	29.4 (27.9-31.0)	28.8 (27.2-30.5)	30.7 (29.0-32.3)	31.6 (30.6-32.6)	31.5 (30.4-32.6)	31.6 (30.4-32.8)
	Women	31.5 (29.3-33.7)	31.2 (28.9-33.5)	32.2 (29.8-34.5)	33.2 (32.0-34.5)	32.8 (31.3-34.3)	33.8 (32.2-35.4)
North Korea	Men	22.0 (19.5-24.5)	21.5 (18.9-24.0)	22.4 (19.8-24.9)	24.5 (21.9-27.1)	24.1 (21.5-26.6)	24.7 (22.0-27.3)
	Women	22.3 (19.0-25.6)	21.8 (18.4-25.1)	22.6 (19.3-26.0)	24.2 (20.8-27.6)	24.0 (20.6-27.5)	24.3 (20.8-27.9)

Country	Sex	Age-standardised mean BMI in 1985 (kg/m^2)			Age-standardised mean BMI in 2017 (kg/m^2)		
		National	Rural	Urban	National	Rural	Urban
Norway	Men	24.5 (23.7-25.2)	24.7 (24.0-25.4)	24.4 (23.6-25.2)	26.7 (25.7-27.7)	26.8 (25.9-27.9)	26.7 (25.6-27.7)
	Women	23.7 (22.7-24.7)	24.3 (23.4-25.3)	23.4 (22.3-24.5)	25.2 (23.7-26.7)	25.8 (24.3-27.3)	25.0 (23.6-26.6)
Occupied Palestinian Territory	Men	25.0 (23.8-26.1)	24.6 (23.4-25.8)	25.2 (24.0-26.4)	27.9 (27.0-28.8)	27.4 (26.4-28.4)	28.1 (27.2-29.0)
	Women	26.7 (25.1-28.3)	25.8 (24.2-27.4)	27.3 (25.6-28.8)	29.8 (28.7-31.0)	29.5 (28.2-30.8)	29.9 (28.7-31.1)
Oman	Men	23.5 (22.6-24.3)	22.9 (21.9-23.9)	23.9 (23.0-24.8)	26.5 (25.6-27.4)	25.6 (24.6-26.7)	26.7 (25.8-27.7)
	Women	25.1 (24.0-26.3)	24.3 (23.0-25.7)	25.7 (24.5-26.9)	27.2 (26.0-28.5)	27.0 (25.6-28.4)	27.3 (26.0-28.6)
Pakistan	Men	20.5 (19.6-21.4)	19.8 (18.9-20.8)	22.2 (21.2-23.1)	23.4 (22.7-24.1)	23.1 (22.3-23.9)	23.9 (23.1-24.7)
	Women	21.0 (19.9-22.1)	20.2 (19.0-21.4)	22.8 (21.7-24.0)	24.8 (24.0-25.6)	23.9 (23.1-24.7)	26.2 (25.3-27.1)
Palau	Men	28.2 (26.7-29.8)	27.0 (25.3-28.8)	28.8 (27.2-30.5)	29.5 (28.5-30.6)	29.5 (28.1-30.9)	29.5 (28.5-30.6)
	Women	28.7 (26.4-30.9)	28.1 (25.6-30.5)	28.9 (26.6-31.3)	29.7 (28.4-31.0)	29.0 (27.2-30.9)	29.8 (28.5-31.1)
Panama	Men	23.8 (22.3-25.2)	23.1 (21.6-24.6)	24.3 (22.8-25.8)	26.4 (25.3-27.4)	25.6 (24.4-26.7)	26.8 (25.7-27.9)
	Women	23.9 (22.0-25.8)	23.1 (21.1-25.1)	24.6 (22.6-26.6)	27.9 (26.5-29.2)	27.3 (25.9-28.8)	28.1 (26.7-29.6)
Papua New Guinea	Men	22.6 (21.4-23.8)	22.4 (21.1-23.6)	24.1 (22.5-25.7)	25.4 (24.2-26.5)	25.3 (24.1-26.5)	25.7 (24.2-27.2)
	Women	22.2 (20.6-23.8)	22.0 (20.4-23.6)	23.5 (21.3-25.7)	25.7 (24.2-27.3)	25.6 (24.0-27.1)	26.9 (24.9-28.9)
Paraguay	Men	24.1 (22.6-25.5)	23.4 (21.8-24.9)	24.9 (23.4-26.4)	27.0 (26.0-27.9)	26.0 (25.1-27.0)	27.6 (26.6-28.5)
	Women	24.6 (22.5-26.8)	24.1 (21.9-26.3)	25.3 (23.1-27.5)	28.1 (27.1-29.3)	27.9 (26.7-29.1)	28.3 (27.1-29.5)
Peru	Men	23.2 (21.9-24.6)	22.0 (20.5-23.4)	23.8 (22.4-25.2)	26.5 (26.2-26.9)	24.5 (24.2-24.9)	27.0 (26.7-27.4)
	Women	24.5 (23.3-25.7)	23.3 (22.1-24.6)	25.1 (23.9-26.3)	27.5 (27.1-27.8)	26.0 (25.6-26.4)	27.9 (27.5-28.2)
Philippines	Men	21.1 (20.3-22.0)	20.7 (19.8-21.6)	21.7 (20.7-22.7)	23.0 (22.5-23.5)	22.5 (22.0-23.1)	23.6 (23.1-24.1)
	Women	20.1 (19.4-20.8)	19.5 (18.8-20.2)	20.9 (20.1-21.6)	23.5 (22.9-24.1)	23.0 (22.4-23.7)	24.1 (23.4-24.7)
Poland	Men	25.4 (24.8-25.9)	25.3 (24.6-26.0)	25.4 (24.8-26.0)	27.5 (27.0-28.0)	27.5 (27.0-28.0)	27.4 (26.9-28.0)
	Women	26.0 (25.1-26.9)	26.3 (25.2-27.3)	25.7 (24.8-26.7)	26.0 (25.4-26.5)	26.4 (25.8-27.0)	25.7 (25.1-26.3)
Portugal	Men	24.4 (23.9-24.9)	24.6 (24.1-25.1)	24.1 (23.6-24.6)	26.3 (25.4-27.2)	26.4 (25.5-27.3)	26.2 (25.3-27.1)
	Women	25.4 (24.0-26.7)	25.6 (24.2-27.0)	25.1 (23.6-26.6)	25.0 (23.9-26.2)	25.1 (23.9-26.4)	25.0 (23.8-26.1)
Puerto Rico	Men	25.8 (24.3-27.3)	24.8 (23.0-26.5)	25.9 (24.4-27.5)	28.4 (27.1-29.7)	27.6 (26.1-29.2)	28.4 (27.1-29.7)
	Women	25.4 (23.5-27.3)	24.3 (22.1-26.4)	25.7 (23.7-27.6)	28.8 (27.4-30.1)	28.3 (26.5-30.1)	28.8 (27.4-30.2)
Qatar	Men	25.7 (24.3-26.9)	25.0 (23.4-26.5)	25.7 (24.4-27.0)	28.7 (27.9-29.6)	27.9 (26.7-29.1)	28.7 (27.9-29.6)
	Women	26.8 (24.8-28.7)	25.2 (23.0-27.4)	27.0 (25.0-28.8)	30.4 (29.4-31.5)	29.6 (27.9-31.3)	30.4 (29.4-31.5)
Romania	Men	24.3 (23.2-25.3)	24.3 (23.1-25.4)	24.3 (23.2-25.3)	27.0 (26.4-27.6)	27.1 (26.5-27.8)	26.9 (26.3-27.5)
	Women	25.2 (23.9-26.5)	25.3 (23.7-26.9)	25.2 (23.8-26.5)	26.8 (26.2-27.5)	27.0 (26.2-27.8)	26.7 (26.0-27.5)
Russian Federation	Men	24.8 (24.3-25.3)	24.9 (24.3-25.6)	24.8 (24.3-25.3)	26.1 (25.3-26.9)	25.8 (25.0-26.7)	26.2 (25.4-27.0)
	Women	26.6 (26.0-27.2)	26.7 (26.0-27.5)	26.5 (25.9-27.1)	26.7 (25.7-27.6)	26.9 (25.8-27.9)	26.6 (25.6-27.6)
Rwanda	Men	19.4 (18.1-20.8)	19.4 (18.0-20.8)	20.2 (18.8-21.6)	21.4 (20.8-21.9)	21.1 (20.5-21.7)	22.1 (21.4-22.7)
	Women	20.3 (18.7-22.0)	20.3 (18.6-22.0)	21.5 (19.8-23.2)	23.0 (22.3-23.8)	22.4 (21.7-23.2)	24.5 (23.7-25.3)
Saint Kitts and Nevis	Men	25.6 (23.9-27.2)	25.3 (23.5-26.9)	26.2 (24.5-28.0)	28.5 (27.1-29.9)	28.3 (26.8-29.7)	28.9 (27.3-30.4)
	Women	26.8 (24.6-29.0)	26.4 (24.1-28.6)	27.7 (25.3-30.0)	30.8 (28.9-32.7)	30.7 (28.7-32.6)	31.1 (29.1-33.1)
Saint Lucia	Men	25.2 (23.7-26.7)	25.1 (23.4-26.6)	25.5 (24.0-27.0)	29.2 (28.2-30.1)	29.2 (28.2-30.1)	29.2 (28.0-30.5)
	Women	25.8 (23.9-27.7)	25.5 (23.4-27.6)	26.5 (24.6-28.4)	30.5 (29.3-31.7)	30.5 (29.2-31.7)	30.5 (29.0-32.1)
Saint Vincent and the Grenadines	Men	23.6 (21.1-26.0)	23.2 (20.7-25.7)	24.2 (21.7-26.7)	26.4 (24.0-28.7)	26.0 (23.6-28.4)	26.7 (24.3-29.1)
	Women	24.6 (21.4-27.7)	24.1 (20.8-27.2)	25.4 (22.1-28.5)	28.3 (25.2-31.4)	28.0 (24.8-31.3)	28.5 (25.3-31.7)
Samoa	Men	27.5 (26.6-28.4)	27.0 (26.0-27.9)	29.6 (28.5-30.7)	30.4 (29.7-31.2)	30.3 (29.5-31.1)	31.1 (30.1-32.1)
	Women	30.0 (28.8-31.2)	29.7 (28.5-31.0)	30.8 (29.5-32.1)	33.9 (32.9-34.9)	33.7 (32.6-34.8)	34.7 (33.4-36.0)
Sao Tome and Principe	Men	21.7 (20.3-23.0)	21.2 (19.7-22.6)	22.4 (21.0-23.9)	23.8 (22.9-24.7)	23.1 (22.1-24.0)	24.2 (23.2-25.2)
	Women	22.5 (20.5-24.3)	22.0 (20.0-23.9)	23.2 (21.2-25.1)	25.7 (24.5-26.8)	24.5 (23.2-25.8)	26.3 (25.0-27.5)

Country	Sex	Age-standardised mean BMI in 1985 (kg/m^2)			Age-standardised mean BMI in 2017 (kg/m^2)		
		National	Rural	Urban	National	Rural	Urban
Saudi Arabia	Men	25.6 (25.0-26.2)	24.8 (23.9-25.6)	25.9 (25.3-26.6)	28.0 (27.3-28.7)	27.0 (26.2-27.8)	28.2 (27.5-28.9)
	Women	27.2 (26.5-28.0)	25.9 (24.8-27.1)	27.7 (27.0-28.5)	29.5 (28.6-30.3)	28.9 (27.8-30.0)	29.6 (28.7-30.4)
Senegal	Men	20.0 (18.9-21.2)	19.6 (18.4-20.9)	20.7 (19.6-21.9)	21.7 (20.8-22.6)	21.3 (20.4-22.2)	22.3 (21.3-23.2)
	Women	21.9 (21.1-22.7)	21.2 (20.3-22.0)	23.2 (22.3-24.1)	23.7 (22.6-24.8)	22.4 (21.3-23.6)	25.3 (24.2-26.5)
Serbia	Men	25.2 (24.2-26.2)	25.1 (24.0-26.2)	25.3 (24.3-26.3)	27.0 (26.4-27.6)	27.0 (26.3-27.7)	27.1 (26.4-27.7)
	Women	25.7 (24.4-27.1)	26.0 (24.5-27.5)	25.5 (24.1-26.9)	25.5 (24.7-26.4)	25.9 (24.9-26.8)	25.3 (24.4-26.2)
Seychelles	Men	22.8 (22.0-23.6)	22.6 (21.7-23.4)	23.1 (22.3-24.0)	25.6 (24.8-26.3)	25.2 (24.4-26.0)	25.9 (25.0-26.7)
	Women	25.1 (24.1-26.0)	25.0 (24.0-26.0)	25.1 (24.0-26.2)	27.9 (27.0-28.9)	27.6 (26.6-28.6)	28.2 (27.1-29.3)
Sierra Leone	Men	20.3 (18.9-21.7)	20.0 (18.5-21.4)	21.1 (19.7-22.6)	22.0 (21.2-22.7)	21.6 (20.8-22.3)	22.5 (21.7-23.4)
	Women	21.5 (19.6-23.5)	20.9 (19.0-22.9)	22.8 (20.8-24.8)	23.6 (22.6-24.5)	22.5 (21.5-23.5)	25.1 (24.1-26.2)
Singapore	Men	22.8 (22.2-23.3)	na*	22.8 (22.2-23.3)	24.4 (23.6-25.1)	na*	24.4 (23.6-25.1)
	Women	23.5 (22.7-24.2)	na*	23.5 (22.7-24.2)	23.0 (22.1-24.0)	na*	23.0 (22.1-24.0)
Slovakia	Men	25.6 (24.6-26.5)	25.5 (24.4-26.6)	25.6 (24.6-26.7)	27.4 (26.6-28.2)	27.4 (26.5-28.3)	27.4 (26.5-28.3)
	Women	25.3 (24.1-26.6)	25.6 (24.1-27.1)	25.1 (23.7-26.5)	25.6 (24.6-26.6)	25.9 (24.6-27.1)	25.3 (24.2-26.5)
Slovenia	Men	25.1 (23.2-26.9)	25.0 (23.1-26.8)	25.2 (23.3-27.0)	26.8 (25.2-28.4)	26.8 (25.1-28.5)	26.9 (25.2-28.5)
	Women	26.0 (23.4-28.6)	26.1 (23.5-28.7)	25.9 (23.3-28.5)	27.0 (24.7-29.2)	27.0 (24.6-29.4)	26.9 (24.6-29.3)
Solomon Islands	Men	23.0 (21.5-24.5)	22.8 (21.3-24.3)	24.2 (22.6-25.9)	25.7 (25.0-26.4)	25.7 (24.9-26.5)	25.7 (24.7-26.8)
	Women	24.1 (21.9-26.3)	23.9 (21.6-26.1)	25.3 (22.9-27.7)	26.9 (26.0-27.8)	26.5 (25.6-27.5)	28.1 (26.8-29.4)
Somalia	Men	20.5 (18.1-22.9)	20.2 (17.7-22.6)	21.2 (18.8-23.7)	22.5 (20.1-24.9)	22.0 (19.6-24.4)	23.3 (20.9-25.8)
	Women	21.3 (18.2-24.5)	20.9 (17.7-24.0)	22.4 (19.3-25.6)	24.0 (20.9-27.0)	23.0 (19.9-26.1)	25.4 (22.3-28.6)
South Africa	Men	23.1 (22.1-24.0)	22.5 (21.5-23.5)	23.7 (22.7-24.7)	25.1 (24.4-25.7)	24.2 (23.5-24.8)	25.6 (24.9-26.2)
	Women	27.0 (25.8-28.3)	26.2 (24.8-27.6)	27.8 (26.5-29.1)	29.6 (28.8-30.3)	28.7 (27.9-29.5)	30.0 (29.2-30.8)
South Korea	Men	22.2 (21.5-22.9)	22.0 (21.2-22.8)	22.4 (21.7-23.1)	24.6 (24.3-24.9)	24.5 (24.1-24.8)	24.6 (24.3-24.9)
	Women	22.3 (21.4-23.2)	22.2 (21.2-23.2)	22.4 (21.5-23.2)	23.1 (22.7-23.5)	23.3 (22.8-23.7)	23.1 (22.7-23.5)
Spain	Men	25.3 (24.7-25.8)	25.6 (24.9-26.2)	25.2 (24.6-25.8)	27.0 (26.4-27.5)	27.0 (26.4-27.6)	26.9 (26.4-27.5)
	Women	25.5 (24.8-26.2)	25.9 (25.1-26.7)	25.4 (24.7-26.1)	24.8 (24.1-25.5)	25.1 (24.3-25.8)	24.7 (24.0-25.4)
Sri Lanka	Men	20.1 (18.9-21.4)	20.0 (18.7-21.2)	20.8 (19.4-22.2)	22.6 (21.8-23.3)	22.4 (21.7-23.1)	23.3 (22.3-24.3)
	Women	20.5 (18.8-22.2)	20.3 (18.5-22.0)	21.5 (19.6-23.5)	23.7 (22.9-24.6)	23.6 (22.7-24.5)	24.4 (23.0-25.8)
Sudan (former)	Men	21.0 (19.6-22.5)	20.8 (19.4-22.3)	21.9 (20.5-23.4)	22.9 (22.3-23.6)	22.5 (21.8-23.2)	23.8 (23.1-24.6)
	Women	22.4 (20.4-24.4)	22.0 (20.0-24.1)	23.9 (21.8-26.0)	24.4 (23.5-25.1)	23.4 (22.6-24.3)	26.4 (25.5-27.3)
Suriname	Men	23.2 (21.5-25.0)	22.6 (20.7-24.5)	23.5 (21.8-25.4)	25.6 (24.3-26.9)	25.2 (23.7-26.8)	25.8 (24.6-27.1)
	Women	24.9 (22.6-27.2)	24.0 (21.5-26.5)	25.4 (23.0-27.7)	28.3 (26.7-29.8)	28.0 (26.1-29.9)	28.4 (26.9-29.9)
Swaziland	Men	22.4 (21.0-23.9)	22.2 (20.7-23.7)	23.3 (21.8-24.8)	24.4 (23.6-25.2)	24.1 (23.3-25.0)	25.4 (24.5-26.4)
	Women	26.7 (24.7-28.6)	26.4 (24.4-28.5)	27.5 (25.4-29.5)	28.9 (27.9-29.8)	28.6 (27.6-29.6)	29.6 (28.4-30.8)
Sweden	Men	24.2 (23.8-24.6)	24.8 (24.3-25.2)	24.1 (23.7-24.5)	26.3 (25.7-27.0)	26.8 (26.1-27.5)	26.3 (25.6-26.9)
	Women	23.8 (23.1-24.6)	24.7 (23.8-25.6)	23.7 (22.9-24.4)	24.9 (24.0-25.7)	25.4 (24.4-26.4)	24.8 (23.9-25.6)
Switzerland	Men	24.8 (24.1-25.5)	25.1 (24.4-25.8)	24.6 (23.9-25.4)	26.5 (25.9-27.1)	26.7 (26.1-27.4)	26.4 (25.8-27.1)
	Women	23.4 (22.5-24.3)	23.8 (22.8-24.8)	23.2 (22.2-24.2)	23.9 (22.7-25.0)	24.1 (22.6-25.7)	23.8 (22.6-24.9)
Syrian Arab Republic	Men	24.6 (23.3-25.8)	24.2 (22.8-25.6)	25.0 (23.6-26.3)	27.4 (26.3-28.5)	27.0 (25.7-28.2)	27.7 (26.5-29.0)
	Women	26.1 (24.4-27.9)	25.3 (23.4-27.2)	27.0 (25.2-28.9)	29.4 (27.8-31.0)	29.0 (27.3-30.7)	29.7 (28.0-31.4)
Taiwan	Men	22.5 (21.7-23.3)	22.3 (21.4-23.1)	22.7 (21.8-23.5)	24.8 (24.1-25.5)	24.7 (24.1-25.4)	24.8 (24.1-25.5)
	Women	22.5 (21.4-23.5)	22.4 (21.2-23.6)	22.5 (21.3-23.6)	23.3 (22.5-24.1)	23.7 (22.9-24.6)	23.2 (22.4-24.1)
Tajikistan	Men	23.5 (21.9-25.0)	23.2 (21.5-24.9)	24.0 (22.3-25.7)	26.0 (25.3-26.7)	25.9 (25.1-26.7)	26.3 (25.2-27.3)
	Women	22.6 (20.6-24.5)	22.1 (20.1-24.1)	23.5 (21.3-25.5)	26.4 (25.6-27.1)	26.3 (25.4-27.1)	26.7 (25.8-27.6)

Country	Sex	Age-standardised mean BMI in 1985 (kg/m^2)			Age-standardised mean BMI in 2017 (kg/m^2)		
		National	Rural	Urban	National	Rural	Urban
Tanzania	Men	20.5 (19.2-21.8)	20.2 (18.9-21.6)	21.9 (20.6-23.2)	22.5 (21.7-23.2)	21.9 (21.0-22.7)	23.7 (22.8-24.6)
	Women	20.7 (19.6-21.9)	20.5 (19.3-21.6)	22.1 (20.9-23.3)	24.1 (23.5-24.7)	23.1 (22.4-23.8)	26.3 (25.6-27.0)
Thailand	Men	21.0 (20.3-21.7)	20.7 (19.9-21.4)	21.7 (20.9-22.5)	23.9 (23.0-24.8)	23.3 (22.4-24.2)	24.5 (23.6-25.3)
	Women	22.0 (20.9-23.0)	21.7 (20.6-22.7)	22.8 (21.7-23.8)	25.0 (23.8-26.1)	24.6 (23.4-25.8)	25.3 (24.1-26.4)
Timor-Leste	Men	18.8 (17.4-20.2)	18.7 (17.2-20.1)	19.3 (17.9-20.8)	21.0 (20.5-21.6)	20.8 (20.3-21.4)	21.5 (20.9-22.1)
	Women	18.5 (16.5-20.5)	18.2 (16.2-20.2)	19.7 (17.6-21.6)	21.2 (20.6-21.8)	20.9 (20.2-21.5)	21.9 (21.3-22.6)
Togo	Men	20.6 (19.2-22.1)	20.3 (18.8-21.8)	21.6 (20.0-23.1)	22.5 (21.6-23.5)	22.1 (21.0-23.1)	23.2 (22.1-24.4)
	Women	20.9 (19.3-22.5)	20.3 (18.6-22.0)	22.7 (21.0-24.4)	24.2 (23.4-25.0)	22.7 (21.9-23.6)	26.3 (25.3-27.2)
Tokelau	Men	29.6 (28.1-31.1)	29.6 (28.1-31.1)	na*	32.3 (31.4-33.2)	32.3 (31.4-33.2)	na*
	Women	31.7 (29.6-33.7)	31.7 (29.6-33.7)	na*	33.4 (32.4-34.4)	33.4 (32.4-34.4)	na*
Tonga	Men	28.8 (27.4-30.2)	28.4 (27.0-29.9)	30.3 (28.7-31.8)	30.9 (30.0-31.8)	30.8 (29.9-31.8)	31.1 (29.8-32.3)
	Women	32.0 (30.0-33.9)	31.7 (29.7-33.8)	32.8 (30.6-34.9)	33.6 (32.4-34.8)	33.3 (32.1-34.6)	34.3 (32.7-36.0)
Trinidad and Tobago	Men	24.3 (23.1-25.5)	24.2 (22.9-25.4)	25.3 (24.1-26.6)	26.9 (25.5-28.2)	26.8 (25.4-28.2)	27.6 (26.0-29.1)
	Women	25.4 (23.8-27.0)	25.2 (23.6-26.9)	26.9 (25.2-28.5)	28.7 (26.8-30.6)	28.6 (26.7-30.5)	29.3 (27.2-31.4)
Tunisia	Men	22.4 (21.3-23.5)	21.9 (20.7-23.0)	22.9 (21.8-24.0)	25.5 (24.6-26.5)	24.8 (23.8-25.8)	25.9 (24.9-26.9)
	Women	24.6 (23.2-26.1)	23.4 (21.8-24.9)	25.7 (24.2-27.2)	27.6 (26.4-28.9)	26.8 (25.4-28.1)	28.1 (26.8-29.3)
Turkey	Men	24.1 (23.3-24.8)	24.0 (23.1-24.8)	24.1 (23.3-24.9)	27.0 (26.6-27.4)	26.8 (26.3-27.3)	27.1 (26.6-27.5)
	Women	26.2 (25.3-27.1)	25.7 (24.7-26.7)	26.7 (25.7-27.7)	28.8 (28.3-29.3)	28.8 (28.1-29.4)	28.8 (28.2-29.3)
Turkmenistan	Men	24.0 (22.4-25.5)	23.6 (22.0-25.3)	24.4 (22.8-26.0)	26.5 (25.7-27.3)	26.3 (25.4-27.3)	26.7 (25.8-27.7)
	Women	23.0 (21.2-24.9)	22.6 (20.6-24.6)	23.6 (21.5-25.6)	26.6 (25.7-27.6)	26.6 (25.4-27.8)	26.6 (25.4-27.9)
Tuvalu	Men	28.3 (25.8-30.7)	27.6 (25.1-30.1)	29.5 (26.9-32.0)	30.3 (27.9-32.7)	30.2 (27.8-32.6)	30.4 (27.9-32.8)
	Women	29.5 (26.4-32.7)	29.2 (26.0-32.4)	30.2 (26.9-33.4)	31.2 (28.1-34.4)	30.7 (27.4-33.9)	31.6 (28.3-34.9)
Uganda	Men	19.5 (18.2-20.8)	19.4 (18.1-20.7)	20.6 (19.3-22.0)	21.5 (21.0-22.0)	21.3 (20.7-21.8)	22.7 (22.1-23.2)
	Women	20.7 (19.3-22.1)	20.5 (19.1-21.9)	22.6 (21.2-24.1)	23.3 (22.7-23.9)	22.8 (22.2-23.5)	25.7 (25.0-26.4)
Ukraine	Men	25.0 (22.5-27.4)	25.1 (22.6-27.5)	25.0 (22.5-27.4)	26.8 (24.3-29.2)	26.6 (24.1-29.0)	26.9 (24.4-29.3)
	Women	25.8 (23.9-27.9)	26.2 (24.0-28.4)	25.6 (23.6-27.7)	26.1 (24.2-28.0)	26.6 (24.5-28.7)	25.9 (23.9-27.8)
United Arab Emirates	Men	25.1 (24.0-26.2)	24.4 (23.2-25.7)	25.3 (24.1-26.4)	28.1 (27.1-29.0)	27.3 (26.3-28.3)	28.2 (27.2-29.2)
	Women	26.5 (25.1-27.9)	24.7 (23.0-26.3)	27.0 (25.6-28.4)	29.5 (28.2-30.7)	28.2 (26.9-29.5)	29.7 (28.4-31.0)
United Kingdom	Men	24.7 (24.4-25.1)	24.9 (24.5-25.4)	24.7 (24.3-25.1)	27.3 (26.9-27.6)	27.4 (27.0-27.7)	27.3 (26.9-27.6)
	Women	24.3 (23.8-24.8)	24.4 (23.8-24.9)	24.3 (23.8-24.8)	26.8 (26.4-27.2)	26.6 (26.2-27.0)	26.9 (26.5-27.3)
United States of America	Men	25.8 (25.3-26.3)	26.1 (25.5-26.7)	25.7 (25.2-26.2)	28.9 (28.5-29.4)	29.2 (28.7-29.7)	28.8 (28.4-29.3)
	Women	25.7 (25.1-26.3)	26.0 (25.2-26.8)	25.6 (25.0-26.2)	29.1 (28.6-29.6)	29.5 (28.9-30.1)	29.0 (28.5-29.6)
Uruguay	Men	24.4 (23.0-25.8)	23.4 (21.8-25.1)	24.5 (23.1-25.9)	27.2 (26.2-28.3)	26.3 (25.0-27.6)	27.3 (26.3-28.3)
	Women	24.6 (22.7-26.6)	23.5 (21.2-25.8)	24.7 (22.8-26.8)	27.4 (26.1-28.7)	27.1 (25.2-28.9)	27.4 (26.1-28.7)
Uzbekistan	Men	23.6 (22.2-24.9)	23.4 (22.0-24.7)	23.8 (22.4-25.2)	26.3 (25.6-27.1)	26.3 (25.5-27.1)	26.3 (25.6-27.1)
	Women	23.1 (21.5-24.7)	22.8 (21.1-24.5)	23.6 (21.9-25.3)	27.5 (26.6-28.4)	27.6 (26.6-28.6)	27.3 (26.3-28.3)
Vanuatu	Men	23.9 (22.6-25.2)	23.6 (22.3-25.0)	25.3 (23.7-26.8)	25.9 (24.9-26.8)	25.8 (24.8-26.8)	26.0 (24.7-27.3)
	Women	24.4 (22.7-26.1)	24.2 (22.4-25.9)	25.4 (23.3-27.5)	26.8 (25.5-28.0)	26.5 (25.2-27.8)	27.6 (25.9-29.3)
Venezuela	Men	24.7 (23.3-26.0)	23.7 (22.2-25.2)	24.9 (23.5-26.3)	26.8 (26.1-27.5)	25.7 (24.9-26.5)	26.9 (26.2-27.6)
	Women	24.0 (22.2-25.9)	22.6 (20.7-24.6)	24.3 (22.5-26.2)	26.9 (26.2-27.7)	26.1 (25.3-27.0)	27.0 (26.2-27.8)
Viet Nam	Men	19.0 (18.3-19.6)	18.8 (18.1-19.5)	19.8 (19.0-20.5)	21.8 (21.2-22.3)	21.4 (20.9-22.0)	22.4 (21.9-23.0)
	Women	18.5 (17.7-19.4)	18.2 (17.4-19.1)	19.8 (18.9-20.7)	21.8 (21.2-22.4)	21.4 (20.8-22.1)	22.5 (21.8-23.1)
Yemen	Men	21.3 (20.0-22.6)	21.2 (19.8-22.5)	22.0 (20.7-23.4)	24.0 (23.0-24.9)	23.6 (22.7-24.6)	24.5 (23.6-25.5)
	Women	20.5 (18.9-22.0)	20.0 (18.4-21.6)	22.5 (20.9-24.1)	24.3 (23.4-25.2)	23.7 (22.8-24.6)	25.5 (24.5-26.4)

Country	Sex	Age-standardised mean BMI in 1985 (kg/m^2)			Age-standardised mean BMI in 2017 (kg/m^2)		
		National	Rural	Urban	National	Rural	Urban
Zambia	Men	20.0 (18.6-21.4)	19.6 (18.1-21.1)	20.7 (19.2-22.0)	22.2 (21.6-22.9)	21.7 (21.0-22.4)	23.0 (22.3-23.7)
	Women	21.4 (20.3-22.6)	20.7 (19.5-21.9)	22.5 (21.3-23.7)	23.9 (23.3-24.5)	22.7 (22.1-23.3)	25.5 (24.9-26.2)
Zimbabwe	Men	21.2 (20.2-22.3)	20.9 (19.8-22.1)	22.1 (21.1-23.1)	22.3 (21.7-22.9)	21.9 (21.2-22.5)	23.3 (22.6-23.9)
	Women	24.6 (23.5-25.9)	24.0 (22.8-25.4)	26.5 (25.3-27.8)	25.3 (24.6-26.1)	24.5 (23.7-25.3)	27.1 (26.2-27.9)

*The entire population live in areas classified as urban (Bermuda, Hong Kong (from 1992 onwards), Nauru and Singapore) or rural (Tokelau).
Numbers in parentheses show 95% credible intervals.

References

1 Finucane, M. M., Paciorek, C. J., Danaei, G. & Ezzati, M. Bayesian estimation of population-level trends in measures of health status. *Stat. Sci.* **29**, 18-25 (2014).

2 Finucane, M. M., Paciorek, C. J., Stevens, G. A. & Ezzati, M. Semiparametric Bayesian density estimation with disparate data sources: a meta-analysis of global childhood undernutrition. *J. Am. Stat. Assoc.* **110**, 889-901 (2015).

3 Danaei, G. *et al.* National, regional, and global trends in systolic blood pressure since 1980: systematic analysis of health examination surveys and epidemiological studies with 786 country-years and 5·4 million participants. *Lancet* **377**, 568-577 (2011).

4 Stevens, G. A. *et al.* Global, regional, and national trends in haemoglobin concentration and prevalence of total and severe anaemia in children and pregnant and non-pregnant women for 1995–2011: a systematic analysis of population-representative data. *Lancet Glob Health* **1**, e16-e25 (2013) DOI: https://doi.org/10.1016/s2214-109x(13)70001-9.

5 NCD Risk Factor Collaboration (NCD-RisC). Worldwide trends in body-mass index, underweight, overweight, and obesity from 1975 to 2016: a pooled analysis of 2416 population-based measurement studies in 128.9 million children, adolescents, and adults. *Lancet* **390**, 2627-2642 (2017).

6 NCD Risk Factor Collaboration (NCD-RisC). Trends in adult body-mass index in 200 countries from 1975 to 2014: a pooled analysis of 1698 population-based measurement studies with 19.2 million participants. *Lancet* **387**, 1377-1396 (2016).

7 Gelman, A. & Jill, J. *Data analysis using regression and multilevel/hierarchical models*. (Cambridge University Press, 2007).

8 Gelman, A. Prior distributions for variance parameters in hierarchical models. *Bayesian Anal.* **1**, 515-533 (2006).

9 Finucane, M. M. *et al.* National, regional, and global trends in body-mass index since 1980: systematic analysis of health examination surveys and epidemiological studies with 960 country-years and 9·1 million participants. *Lancet* **377**, 557-567 (2011).

10 Breslow, N. E. & Clayton, D. G. Approximate inference in generalized linear mixed models. *J. Am. Stat. Assoc.* **88**, 9-25 (1993).

11 Rue, H. & Held, L. *Gaussian Markov random fields: theory and applications*. (Chapman & Hall, 2005).

12 Wood, S. N. *Generalized additive models: an introduction with R*. (CRC Press, 2006).

13 Harville, D. A. *Matrix algebra from a statistician's perspective*. (Springer-Verlag, 2008).

14 Kuchibhatla, M. N., Fillenbaum, G. G., Kraus, W. E., Cohen, H. J. & Blazer, D. G. Trajectory classes of body mass index in a representative elderly community sample. *J. Gerontol. A. Biol. Sci. Med. Sci.* **68**, 699-704 (2013).

15 Østbye, T., Malhotra, R. & Landerman, L. R. Body mass trajectories through adulthood: results from the National Longitudinal Survey of Youth 1979 Cohort (1981–2006). *Int. J. Epidemiol.* **40**, 240-250 (2011).

16 Wang, M. *et al.* Body mass index trajectories among middle-aged and elderly Canadians and associated health outcomes. *J. Environ. Public Health* **2016**, 7014857 (2016).

17 NCD Risk Factor Collaboration (NCD-RisC). A century of trends in adult human height. *eLife* **5**, e13410 (2016) DOI: https://doi.org/10.7554/eLife.13410.

18 NCD Risk Factor Collaboration (NCD-RisC). Worldwide trends in diabetes since 1980: a pooled analysis of 751 population-based studies with 4.4 million participants. *Lancet* **387**, 1513-1530 (2016).

19 NCD Risk Factor Collaboration (NCD-RisC). Worldwide trends in blood pressure from 1975 to 2015: a pooled analysis of 1479 population-based measurement studies with 19.1 million participants. *Lancet* **389**, 37-55 (2017).
