# Supplementary material for: Rising rural body-mass index is the main driver of the global obesity epidemic in adults
Source: Nature. 2019 May 8;569(7755):260–4. doi: 10.1038/s41586-019-1171-x (PMC6784868; doi:10.1038/s41586-019-1171-x)
Supplement: Supplementary file 2 — Reporting Summary [file 41586_2019_1171_MOESM2_ESM.pdf]

nature research

Corresponding author(s): Majid Ezzati

Reporting Summary

Nature Research wishes to improve the reproducibility of the work that we publish. This form provides structure for consistency and transparency in reporting. For further information on Nature Research policies, see Authors & Referees and the Editorial Policy Checklist.

Statistical parameters

When statistical analyses are reported, confirm that the following items are present in the relevant location (e.g. figure legend, table legend, main text, or Methods section).

n/a	Confirmed	
☒	☐	The exact sample size (n) for each experimental group/condition, given as a discrete number and unit of measurement
☒	☐	An indication of whether measurements were taken from distinct samples or whether the same sample was measured repeatedly
☒	☐	The statistical test(s) used AND whether they are one- or two-sided<br>*Only common tests should be described solely by name; describe more complex techniques in the Methods section.*
☒	☐	A description of all covariates tested
☒	☐	A description of any assumptions or corrections, such as tests of normality and adjustment for multiple comparisons
☐	☒	A full description of the statistics including central tendency (e.g. means) or other basic estimates (e.g. regression coefficient) AND variation (e.g. standard deviation) or associated estimates of uncertainty (e.g. confidence intervals)
☒	☐	For null hypothesis testing, the test statistic (e.g. F, t, r) with confidence intervals, effect sizes, degrees of freedom and P value noted<br>*Give P values as exact values whenever suitable.*
☐	☒	For Bayesian analysis, information on the choice of priors and Markov chain Monte Carlo settings
☒	☐	For hierarchical and complex designs, identification of the appropriate level for tests and full reporting of outcomes
☒	☐	Estimates of effect sizes (e.g. Cohen's d, Pearson's r), indicating how they were calculated
☐	☒	Clearly defined error bars<br>*State explicitly what error bars represent (e.g. SD, SE, CI)*

Our web collection on statistics for biologists may be useful.

Software and code

Policy information about availability of computer code

Data collection	Processing of secondary data was conducted using the statistical software R (version 3.5.1).
Data analysis	All analyses were conducted using the statistical software R (version 3.5.1). The code for national analysis of mean risk factor trends is available at www.ncdrisc.org. The code for analysis of trends in urban and rural subgroups is available from www.ncdrisc.org.

For manuscripts utilizing custom algorithms or software that are central to the research but not yet described in published literature, software must be made available to editors/reviewers upon request. We strongly encourage code deposition in a community repository (e.g. GitHub). See the Nature Research guidelines for submitting code & software for further information.

Data

Policy information about availability of data

All manuscripts must include a data availability statement. This statement should provide the following information, where applicable:

- Accession codes, unique identifiers, or web links for publicly available datasets
- A list of figures that have associated raw data
- A description of any restrictions on data availability

This is a data-pooling study that brings together almost 2000 disparate data sources and uses a Bayesian hierarchical model to estimate population risk factor trends. Estimates of mean BMI by country, year, sex and place of residence (urban and rural) are available from www.ncdrisc.org. Estimates of mean BMI by

country, year, sex and urban and rural place of residence are available from http://www.ncdrisc.org/. Input data from publicly available sources can also be downloaded from http://www.ncdrisc.org/. For other data sources, contact information for data providers can be obtained from http://www.ncdrisc.org/.

Field-specific reporting

Please select the best fit for your research. If you are not sure, read the appropriate sections before making your selection.

☐ Life sciences ☒ Behavioural & social sciences ☐ Ecological, evolutionary & environmental sciences

For a reference copy of the document with all sections, see nature.com/authors/policies/ReportingSummary-flat.pdf

Behavioural & social sciences study design

All studies must disclose on these points even when the disclosure is negative.

Study description	We pooled and re-analysed population-based data on height and weight in adults to estimate trends in mean BMI from 1985 to 2017 by urban and rural place of residence fro 200 countries and territories, using a Bayesian hierarchical model.
Research sample	We pooled data from 2,009 population-based studies of human anthropometry conducted in 190 countries, with measurement of height and weight in over 112 million adults aged 18 years and older. Studies were representative of a national, subnational or community population.
Sampling strategy	We included data collected using a probabilistic sampling method with a defined sampling frame. We therefore included studies with simple random and complex survey designs but excluded convenience samples.
Data collection	We used data on measured height and weight to calculate body-mass index. We excluded self-reported data.
Timing	We pooled data collected from 1985 to 2017. We also included national studies for the 3 years prior to 1985 (n=17), assigning them to 1985, so that they can inform the estimates in countries with slightly earlier national data.
Data exclusions	We excluded all data sources that were solely based on self-reported weight and height without a measurement component because these data are subject to biases that vary by geography, time, age, sex and socioeconomic characteristics. Due to these variations, approaches to correcting self-reported data leave residual bias. We also excluded data sources on population subgroups whose anthropometric status may differ systematically from the general population, including:<br>• studies that had included or excluded people based on their health status or cardiovascular risk;<br>• studies whose participants were only ethnic minorities;<br>• specific educational, occupational, or socioeconomic subgroups, with the exception noted below;<br>• those recruited through health facilities, with the exception noted below; and<br>• women aged 18-19 years in surveys which sampled only ever-married women or measured height and weight only among mothers.<br><br>Our exclusion criteria were established at the initiation of the study to ensure all data were representative.
Non-participation	Our inclusion/exclusion criteria were designed to ensure participants of the surveys included were representative of the general population from which each sample was drawn.
Randomization	Our study is descriptive, and we did not carry out experiments.

Reporting for specific materials, systems and methods

Materials & experimental systems

n/a	Involved in the study	
☒	☐	Unique biological materials
☒	☐	Antibodies
☒	☐	Eukaryotic cell lines
☒	☐	Palaeontology
☒	☐	Animals and other organisms
☒	☐	Human research participants

Methods

n/a	Involved in the study	
☒	☐	ChIP-seq
☒	☐	Flow cytometry
☒	☐	MRI-based neuroimaging
